# Flexible Modeling of Epidemics with an Empirical Bayes Framework

**DOI:** 10.1371/journal.pcbi.1004382

**Published:** 2015-08-28

**Authors:** Logan C. Brooks, David C. Farrow, Sangwon Hyun, Ryan J. Tibshirani, Roni Rosenfeld

**Affiliations:** 1 School of Computer Science, Carnegie Mellon University, Pittsburgh, Pennsylvania, United States of America; 2 Department of Statistics, Carnegie Mellon University, Pittsburgh, Pennsylvania, United States of America; University of New South Wales, AUSTRALIA

## Abstract

Seasonal influenza epidemics cause consistent, considerable, widespread loss annually in terms of economic burden, morbidity, and mortality. With access to accurate and reliable forecasts of a current or upcoming influenza epidemic’s behavior, policy makers can design and implement more effective countermeasures. This past year, the Centers for Disease Control and Prevention hosted the “Predict the Influenza Season Challenge”, with the task of predicting key epidemiological measures for the 2013–2014 U.S. influenza season with the help of digital surveillance data. We developed a framework for in-season forecasts of epidemics using a semiparametric Empirical Bayes framework, and applied it to predict the weekly percentage of outpatient doctors visits for influenza-like illness, and the season onset, duration, peak time, and peak height, with and without using Google Flu Trends data. Previous work on epidemic modeling has focused on developing mechanistic models of disease behavior and applying time series tools to explain historical data. However, tailoring these models to certain types of surveillance data can be challenging, and overly complex models with many parameters can compromise forecasting ability. Our approach instead produces possibilities for the epidemic curve of the season of interest using modified versions of data from previous seasons, allowing for reasonable variations in the timing, pace, and intensity of the seasonal epidemics, as well as noise in observations. Since the framework does not make strict domain-specific assumptions, it can easily be applied to some other diseases with seasonal epidemics. This method produces a complete posterior distribution over epidemic curves, rather than, for example, solely point predictions of forecasting targets. We report prospective influenza-like-illness forecasts made for the 2013–2014 U.S. influenza season, and compare the framework’s cross-validated prediction error on historical data to that of a variety of simpler baseline predictors.

## Author Summary

Influenza epidemics occur annually, and incur significant losses in terms of lost productivity, sickness, and death. Policy makers employ countermeasures, such as vaccination campaigns, to combat the occurrence and spread of infectious diseases, but epidemics exhibit a wide range of behavior, which makes designing and planning these efforts difficult. Accurate and reliable numerical forecasts of how an epidemic will behave, as well as advance notice of key events, could enable policy makers to further specialize countermeasures for a particular season. While a large amount of work already exists on modeling epidemics in past seasons, work on forecasting is relatively sparse. Specially tailored models for historical data may be overly strict and fail to produce behavior similar to the current season. We designed a framework for predicting epidemics without making strong assumptions about how the disease propagates by relying on slightly modified versions of past epidemics to form possibilities for the current season. We report forecasts generated for the 2013–2014 Centers for Disease Control and Prevention (CDC) “Predict the Influenza Season Challenge”, and assess its accuracy retrospectively.

## Introduction

Seasonal influenza epidemics occur each year and incur significant economic burden, morbidity, and mortality. The annual impact in the United States has been estimated at 611K lost undiscounted life-years, 3.1M hospitalized days, 31.4M outpatient visits, and $87.1B in economic burden [1]. Accurate and reliable forecasts offer many opportunities to improve preparedness and response to influenza epidemics. Long-term predictions could be used to help select a vaccine for the next season. Forecasts within a season can help policy makers to tailor vaccination campaigns and advisories, hospitals to prepare staff and beds, and individuals and organizations to plan for vaccination and potential sickness. Despite the notable impacts of the disease, though, many weaknesses of influenza surveillance and prediction systems in the past [2] remain today. Capabilities to observe and forecast the prevalence of influenza and similar diseases lag considerably, e.g., behind analogues in meteorology. During the 2013–2014 flu season, the Centers for Disease Control and Prevention (CDC) hosted the “Predict the Influenza Season Challenge” [3], which encouraged teams to forecast features of the current epidemic progression that would be useful to policy makers, and to take advantage of digital surveillance such as search engine and social network data. The competition established a closer relationship between forecasters and policy makers, and provided valuable assessment of the performance of true (prospective) within-season forecasts.

Existing work on modeling influenza epidemic curves generally falls into one of three categories:

**Compartmental models** estimate the number of people in various states related to a disease [4]. For example, the SIR model approximates dynamics between the proportions of the population susceptible to influenza, infected with the virus, and recovered from infection. Common assumptions include that any pair of individuals in a population are equally likely to interact, and that different strains of influenza behave identically. Careful construction of compartmental models incorporating additional states and exogenous variables can improve on the results of more basic alternatives, and have outperformed alternative models in other settings [5, 6].
**Agent-based models** generate synthetic populations based on census data and build complex schemes of interaction and disease behavior in synthetic humans [7–11]. It is common for these systems to be applied to the special case of a single, novel strain of influenza.
**Parametric statistical models** are tools from time series modeling that are less closely tied with mechanistic assumptions of how flu is transmitted. Simple approaches include linear autoregression, which estimates flu activity at some time with a linear function of the flu activity in the recent past. A referee identified beta regression [12, 13] as an alternative with observations constrained within the range of possible wILI values (0%–100%). More complex methods include generalized linear models (GLM), Box-Jenkins analysis [14, 15], seasonal autoregressive integrated moving-average models [16], and generalized autoregressive moving-average models [17].

Past forecasting efforts [18, 19] usually take a compartmental model [20, 21], agent-based model [22], or parametric statistical model [23–26], and condition on partial data to predict flu activity levels one to ten weeks in the future. Other methods include prediction markets [27], which combine expert predictions using a stock market-like system, and the method of analogues (*k* nearest neighbors) [28], which makes predictions of future flu activity levels using similar patterns from the past, without assuming a strict model. The forecasting targets and (sometimes qualitative) evaluation metrics selected vary widely between works [18, 19], making it difficult to compare results for different methods. The 2013–2014 CDC challenge provided a standardized set of forecasting targets, allowing for some qualitative and quantitative comparisons on a single season. The contest winner [29] used an SIRS compartmental model approach [20, 30, 31].

We take a nonmechanistic approach, generating possibilities for the current season’s epidemic curve using modified versions of past seasons’ curves, incorporating adjustments in the timing, pace, and intensity of the epidemic informed by variability in historical data, and accommodating noise in observations. Our method models the process generating the data nonparametrically, using a large family of smooth curves to produce fairly close fits to historical data, relying on a small set of transformations of these curves to construct a probability distribution for the underlying level of ILI this season. While the method of analogues is similar in this regard as a nonparametric method, our framework considers the entire season as a unit and models observational noise, which differs from the traditional perspective in nearest neighbor modeling. Our framework outputs a distribution over epidemic curves, which can be used to produce histograms, credible intervals, and point predictions of the season’s onset, peak week, peak, and duration, as well as individual wILI measurements; existing applications of the method of analogues generate separate point predictions for each wILI measurement.

## Materials and Methods

### Surveillance data

#### U.S. Outpatient Influenza-like Illness Surveillance Network (ILINet)

The Centers for Disease Control and Prevention (CDC) release several forms of surveillance data regarding the prevalence, type, and impact of influenza-like illness (ILI) in the United States [32, 33]. These data (as well as Google Flu Trends and our predictions) are in terms of ILI, because doctors do not generally diagnose influenza specifically, but rather as part of a broader syndromic category of ILI. Since ILI is generally not notifiable in the U.S., its activity is measured not with case counts, but with the percentage of doctor’s visits that are ILI-related during a given epidemiological week. The U.S. Outpatient Influenza-like Illness Surveillance Network (ILINet) is a group of over 2,900 outpatient healthcare providers that voluntarily provide information about the number of total visits and ILI-related visits that they receive. The CDC compiles ILINet reports, adjusts for effects of changes in participation, and weights data based on state population. The result, called percent weighted ILI (wILI), is released on a weekly basis, with about a weekly delay for reporting and processing, at a national level and for each of the ten Health & Human Services (HHS) regions, broken down by age group; data may be revised in later weeks. This data is available for every season since the 1997–1998 season. The CDC did not report wILI data for weeks 21–39 in the first six seasons of ILINet surveillance. Beginning with the 2003–2004 season, wILI data is reported for every week.

#### Google Flu Trends (GFT)

Google Flu Trends (GFT) is a system designed to estimate (“nowcast”) CDC ILINet data up to and including the current week using Google query data. GFT results are available in near real-time, with final estimates of ILI activity in a given week available soon after that week ends. Estimates are available for the nation as whole and the ten HHS regions, as well as smaller geographical units such as states. The original algorithm [34], launched in 2008, was updated in 2009 [35] and 2013 [36] to improve performance by regenerating its selection of queries using additional data, and by revising the method itself. Despite these modifications, GFT has recently drawn criticism [37, 38] on a number of issues, including its performance versus some simple alternatives. However, existing work at the start of the competition indicated that GFT was the most accurate of existing digital surveillance systems [39], and is helpful when used in combination with CDC ILINet data [17]. We used GFT results as a proxy for CDC ILINet data for a few weeks before our predictions were made, when CDC data was not yet released, or could be revised significantly later. S6 Fig illustrates the relationship between ILINet data, GFT data, and underlying phenomena.

### Empirical Bayes framework

The forecasting framework is composed of five major procedures:

1. Model past seasons’ epidemic curves as smoothed versions plus noise.
2. Construct prior for the current season’s epidemic curve by considering sets of transformations of past seasons’ curves.
3. Estimate what the wILI values in recent past will be after their final revisions, using non-final wILI and GFT.
4. Weight possibilities for current season’s epidemic curve using estimates of final revised wILI.
5. Calculate forecasting targets for each possibility, and report results.

The first two steps only need to be executed once, at the beginning of the current season. As additional data becomes available throughout the season, we generate forecasts using steps 3–5.

We perform predictions for each geographical unit—the U.S. as a whole or individual HHS regions—separately. Historically, surveillance has focused on influenza activity between epidemiological weeks 40 and 20, inclusive. We define seasons as epidemic weeks 21 to 39, the “preseason”, together with weeks 40 to 20. During the competition, data was available for 15 historical seasonal influenza epidemics. We excluded the 2009–2010 season from the data since it included nonseasonal behavior from the 2009 pandemic in the preseason. Additionally, there was partial data available for the 2013–2014 season.

#### Data model

We view wILI trajectories for a geographical unit *r* as the sum of some underlying ILI curve plus noise:

$$y_{i}^{r,\;s}=f^{r,\;s}\left(i\right)+\epsilon_{i}^{r,\;s},\;\epsilon_{i}^{r,\;s}∼N\left(0,\tau^{r,\;s}\right),\;\text{for}\;\text{each}\;\text{week}\;i,$$ (1)

where $y_{i}^{r,\;s}$ is the wILI value for the *i*th week of season *s*, *f* ^*r*, *s*^ is the underlying curve, and $\epsilon_{i}^{s}$ is (independent) normally distributed noise. We estimate the underlying ILI curve ${\hat{f}}^{r,\;s}$ from the wILI curve *y* ^*r*, *s*^ with quadratic trend filtering [40] for each historical season *s*. This method smooths out fluctuations in the wILI data, producing a new set of points that lie on a piecewise quadratic curve. We use the cv.trendfilter [41] method to select an appropriate amount of smoothness for each curve, then estimate the corresponding noise level ${\hat{\tau }}^{r,\;s}$:

$${\left({\hat{\tau }}^{r,\;s}\right)}^{2}=\underset{i}{\mathrm{avg}}{\left[y_{i}^{r,\;s}-{\hat{f}}^{r,\;s}\left(i\right)\right]}^{2}.$$

The quadratic trend filtering procedure produces one point for each available wILI observation, i.e., 33 or 34 for the first six seasons, and 52 or 53 for the rest. We fill in the curve on the rest of the real line by copying the first available wILI value at earlier times, copying the last measurement at later times, and using linear interpolation at non-integer values. These filled-in values are later used by the peak week and pacing transformations. Trend filtering seems better suited for epidemic data with than the more common smoothing spline fit because it is more “locally adaptive”, responding better to varying levels of smoothness in data [40], e.g., relatively sharp peaks mixed with smoother, flatter, less active regions. S7 Fig compares trend filtering, SIR, and smoothing spline fits for two fairly representative wILI trajectories. A referee identified Bayesian nonparametric covariance regression [42] as another alternative for fitting curves and noise models, which can incorporate heteroscedasticity and spatial relationships.

#### Prior

The key assumption of the framework is that the current season will resemble one of the past seasons, perhaps with a few changes. **Shape**: The general shape *f* ^*r*^ of the underlying curve is taken from one of the past seasons. We select each of the historical shapes with equal probability: $f^{r}∼\mathrm{Unif}\left\{{\hat{f}}^{r,\;s}\;:\;\text{historical}\;\text{season}\;s\right\}$. **Noise**: The standard deviation of the normally distributed noise at each week is assumed to take on values from the past years’ candidates with equal probability: $\sigma ∼\mathrm{Unif}\{{\hat{\tau }}^{r,\;s}\;:\;\text{historical}\;\text{season}\;s\}$. Alternative choices are discussed in S2 Text. **Peak height**: The distribution of underlying peak heights is drawn from a continuous uniform distribution: *θ* ∼ *U* [*θ*_*m*_, *θ*_*M*_]. We use an unbiased estimator [43] for *θ*_*m*_ and *θ*_*M*_ based on past seasons’ trend filtered curves. The resulting curve is $f_{2}^{r}(i)=b^{r}+\frac{\theta^{r}-b^{r}}{\max_{j}\;f^{r}(j)-b^{r}}(f^{r}(i)-b^{r})$, where *b* ^*r*^ is the current year’s CDC baseline wILI level (i.e., the onset threshold) for the selected geographical region *r*, e.g., 2% for the U.S. as a nation for the 2013–2014 flu season. **Peak week**: The distribution of underlying peak weeks is formed in a similar manner to the peak height distribution; we find unbiased estimators *μ*_*m*_, *μ*_*M*_ for uniform distribution bounds, but restrict the distribution to integral output: $\mu ∼\mathrm{Unif}\{i\in \left\{1..53\right\}\;:\;\mu_{m}\leq i\leq \mu_{M}\}$. The resulting curve is $f_{3}^{r}\left(i\right)=f_{2}^{r}\left(i-\mu^{r}+{arg max}_{j}\;f_{2}^{r}\left(j\right)\right)$. **Pacing**: We allow for variations in the “pace” of an epidemic by incorporating a time scale that stretches the curve about the peak week; the distribution of time scale factors is *ν* ∼ *U* [0.75, 1.25]. The resulting curve is $f_{4}^{r}\left(i\right)=f_{3}^{r}\;(\frac{i-{arg max}_{j}\;f_{3}^{r}\left(j\right)}{\nu }+{arg max}_{j}\;f_{3}^{r}\left(j\right))$.

To generate a possible curve for the current season, i.e., to sample from the prior, we independently sample a shape, noise level, peak height, peak week, and pacing parameter from the above distributions, then generate the corresponding wILI curve. We have also developed and are investigating an alternative “local” transformation prior [44] that does not use information from other historical curves when transforming a particular historical curve *f*, but instead reuses the noise level for *f* and makes smaller *changes* to the peak week and height of *f*, which are restricted to a smaller, predefined range; this is more appropriate for surveillance data with less regular seasonal behavior, such as dengue case counts in Brazil.

In total, we model the underlying curve *f* ^*r, s*_curr_^ for the current season as the curve generated by a randomly sampled parameter configuration ⟨*f* ^*r*^, *σ* ^*r*^, *ν* ^*r*^, *θ* ^*r*^, *μ* ^*r*^⟩, using the following equation:

$$f^{r,\;s_{\mathrm{curr}}}\left(i\right)=f_{4}^{r}\left(i\right)=b^{r}+\frac{\theta^{r}-b^{r}}{\max_{j}\;f^{r}\left(j\right)-b^{r}}\;[f^{r}\;(\frac{i-\mu^{r}}{\nu^{r}}+\underset{j}{arg max}f^{r}\left(j\right))-b^{r}].$$

**Fig 1:**
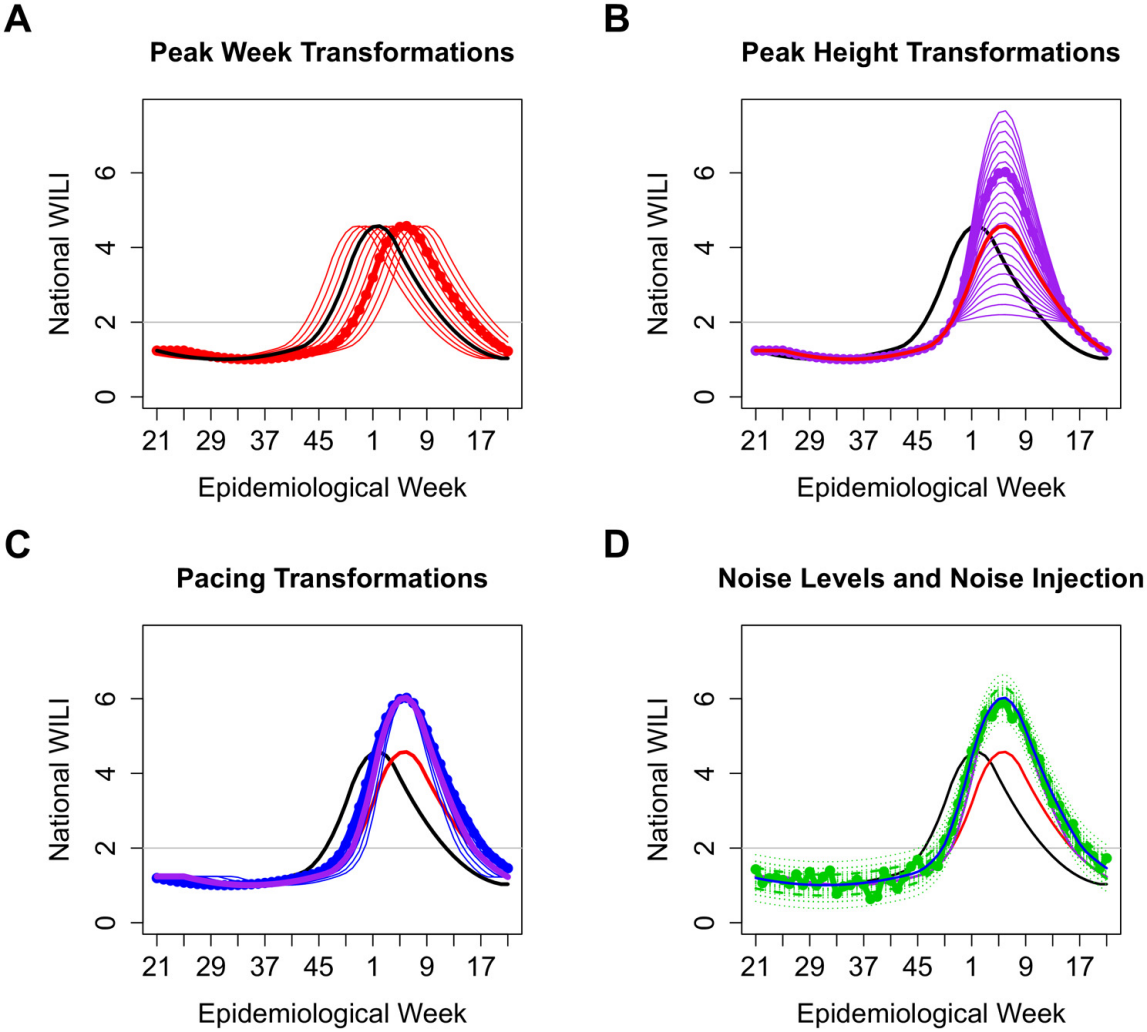
Examples of possible peak week, peak height, and pacing transformations, and different noise levels. Thick black, original curve; red, possible peak week transformations; thick red, a random peak week transformation; purple, possible peak height transformations; thick purple, a random peak height transformation; blue, possible pacing transformations; thick blue, a random pacing transformation; dotted green, 5th and 95th (pointwise) percentiles of noise distribution for possible noise levels; dashed green, percentiles for a random noise level; thick green, one possible trajectory for the selected transformations and noise level. (A) Peak week transformations. Peak weeks of historical smoothed curves occurred between weeks 51 and week 10 of the next year, so we limit transformations to give peak weeks roughly within this range. (B) Peak height transformations. Peak heights of historical smoothed curves were between 2% and 8%, so we limit transformations to give peak heights roughly within this range. (C) Pacing transformations. We stretch the curve by a factor between 75% and 125% about the peak week. (D) Noise levels. We randomly select one of 15 noise levels from the fitting procedure and add this level of Gaussian noise to the transformed curve.

Fig 1 illustrates the peak week, peak height, and pacing transformations, and different levels of noise that could be considered. The data model for the current season’s wILI values *y* ^*r, s*_curr_^ is the same as that for historical seasons, shown in Eq 1.

#### Sampling from the posterior

We use importance sampling [45] to obtain a large set of curves from the posterior weighted by how closely they match the epidemic curve so far, beginning with week 40. More concretely, we obtain a single weighted sample from the posterior by (i) sampling a historical smoothed curve *f*, noise level *σ*, and transformation parameters *ν*, *θ*, and *μ* from the prior; (ii) applying the peak height, peak week, and pacing transformations; (iii) assigning the curve an “importance weight” or “likelihood” based on how well it matches existing observations for the current seasons; and (iv) drawing noisy wILI observations around the curve for the rest of the season. We apply this procedure many times to obtain a collection of possible wILI trajectories and associated weights, forming a probability distribution over possible futures for the current season. The sampling algorithm is described in more detail in S1 Text. Our implementation is written in R [46] and Rcpp [47, 48], and takes roughly one second to produce a forecast for a given geographical unit.

#### Forecasting targets

For the CDC challenge, we were interested in four forecasting targets: the epidemic’s onset, peak week, peak, and duration. These features are already used to summarize epidemic curves and perform retrospective analysis, and the CDC selected them as forecasting targets for the competition, as accurate predictions of these milestones would assist policy makers in planning vaccination campaigns, resource allocation, and messages to the public. **Onset**: The first week that the wILI curve is above a specified CDC baseline wILI level, and remains there for at least the next two weeks. For example, the 2013–2014 national baseline wILI level was 2%, so the onset was the first in at least three consecutive weeks with wILI levels above 2%. **Peak Week**: The week in which the wILI curve attains its maximum value. **Peak**: The maximum observed wILI value in a season. **Duration**: Roughly, how many weeks the wILI level remained above the CDC baseline since the onset. We defined this more rigorously as the sum of the lengths of all periods of three or more consecutive weeks with wILI levels above the CDC baseline.

We generate distributions for each of these targets by repeatedly (i) sampling a possible wILI trajectory and associated weight from the posterior, (ii) calculating the four forecasting targets for that trajectory, and (iii) storing these four values along with the trajectory’s weight. We represent these forecasting target posterior distributions with histograms, and generate point estimates by taking the posterior mean for each target.

#### Incorporating non-final and digital data

At the time that forecasts were generated, GFT estimates were available for the current week and previous week, while ILINet wILI measurements were available only for times further in the past. We produced one set of forecasts using the latest ILINet data by itself, and another that incorporated GFT data. We considered two methods of including GFT data: (i) using GFT estimates only for the two weeks in which ILINet data was not yet available, and (ii) also using GFT estimates in place of recent ILINet values which may be revised significantly in the future. Since GFT attempts to minimize root mean squared error (RMSE) on the logit scale [34] (subject to some regularization [36]), we performed linear regression to reduce the RMSE on the linear scale that our framework works with.

## Results

### Predictions for the 2013–2014 season

**Fig 2:**
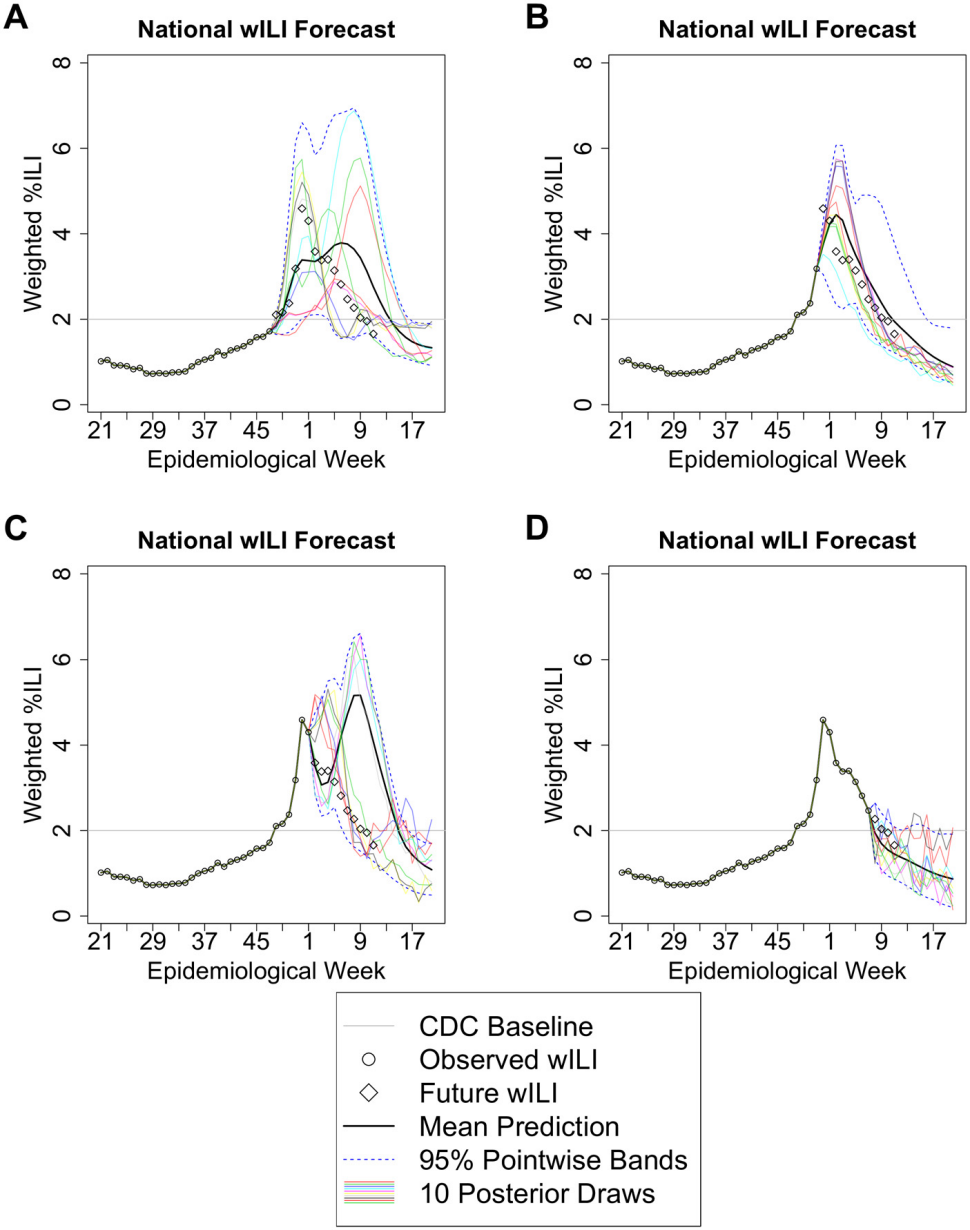
2013–2014 national forecast, retrospectively, using the final revisions of wILI values, using revised wILI data through epidemiological weeks (A) 47, (B) 51, (C) 1, and (D) 7.

For the CDC challenge, we generated biweekly forecasts from December 5 (epidemiological week 49) to March 27 (week 9), for the nation as a whole, and individually for each the 10 HHS regions. Included below is a summary of our framework’s forecasts throughout the season, based on revised wILI data and no GFT. Fig 2 shows 10 draws from the posterior representing likely wILI curves, as well as the posterior mean and 5th and 95th posterior percentiles for the wILI value for each week. S1 Fig contains these forecasts for the entire 2013–2014 season, along with histograms and point predictions for the onset, peak week, peak height, and duration.

#### Week 49 (December 5) forecast, using wILI data through week 47

During the week of the first forecast, all of the available wILI values are below the CDC onset threshold, as shown in Fig 2A. Predictions for the onset are concentrated near the actual value, and the error in the point prediction is fairly small (1.58 weeks). Much of this error can be attributed to the sudden jump in wILI at the onset, which corresponds to Thanksgiving week. The number of patients seen per reporting provider in ILINet drops noticeably every season on Thanksgiving week and around winter holidays; at these times, there is a systematic bias towards higher wILI values.

In the 2013–2014 season, the number of total visits dropped from 869362 on the week before Thanksgiving to 661282 on Thanksgiving week, and from 808701 on week 51 to 607611 on week 52. The number of ILI visits also dropped slightly on Thanksgiving week (from 14995 to 13909, not as significant as the drop in total visits), then increased continuously until it achieved a peak on epidemiological week 1 (and remained nearly the same on weeks 2 and 3). The forecasts for the overall wILI curves and the other three targets contain a large degree of uncertainty than the onset prediction, shown by wider histograms that more closely resemble the prior distribution. The peak of the epidemic could potentially occur early or late (90% credible interval: week 52–10), and be mild or strong (90% credible interval: peak wILI between 2.4% and 7.5%).

#### Week 1 (January 2) forecast, using wILI data through week 51

Fig 2B shows that, with data available up to the week before the sudden peak, the framework matches the observed wILI trajectory fairly closely with many of the posterior draws. The sudden peak can be explained as a combination of elevated ILI-related visits combined with a relative decrease in unrelated visits associated with winter holidays. The framework selects posterior curves with slightly later peaks of similar height, as well as seasons with much later peaks, which contain secondary peaks around the winter holidays. The onset has already been confirmed, so the corresponding histogram, shown in S1 Fig, is a point mass. Duration predictions narrow around the actual duration (90% credible interval shrinks by 3 weeks).

#### Week 3 (January 16) forecast, using wILI data through week 1

Fig 2C indicates that, after the sudden peak, the posterior for the 2013–2014 epidemic contained primarily transformed versions of the 2006–2007 curve, which featured a relatively large secondary peak around winter holidays, followed by a primary peak in early February. Subsequent forecasts, such as the week 9 forecast show in Fig 2D, continue to predict another, later, primary or secondary peak, until some time late in the season, in which forecasts match the falling tail of the epidemic curve. The prediction of a second peak is a significant mismatch from the observed single-peaked season. Our cross-validation analysis revealed that retrospective forecasts only produced this type of mismatch in one other season at this epiweek, with half the error. We also discuss some extensions in S2 Text that may help prevent these types of mismatches.

### Point prediction trends

**Fig 3:**
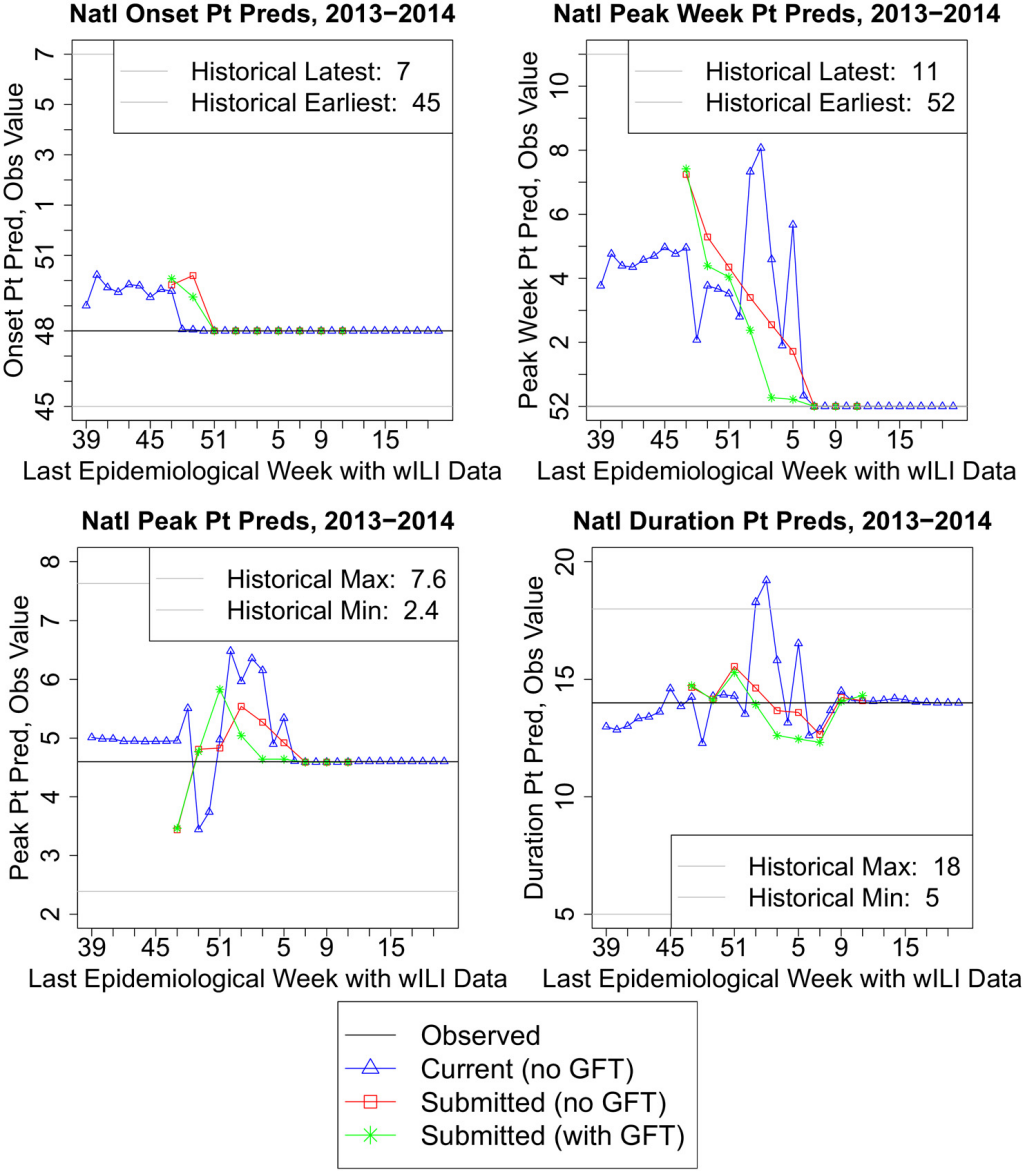
Point predictions and observed values of the forecasting targets for the 2013–2014 season. Black, observed target value; blue, our current framework’s predictions using revised ILINet wILI data; red, our submitted point predictions using ILINet data only; green, our submitted point predictions that used both ILINet and GFT data. Historical target value ranges exclude the 2009–2010 and 2013–2014 seasons.

Fig 3 shows (A) the observed national onset, peak week, peak height, and duration for the 2013–2014 season; (B) retrospective forecasts using revised wILI data only (no GFT); (C) real-time forecasts using wILI data only; and (D) real-time forecasts using both wILI and GFT. The real-time forecasts (submitted to the CDC as part of the prediction challenge) used older versions of the forecasting framework and wILI data. The small error in the onset before it occurred, as well as some of the error in peak week and height predictions, can be attributed to not factoring in holiday effects; at least some of these effects are smoothed out by the trend filtering process, or shifted to different times and heights by the peak week and height transformations. Later errors in the peak week, peak, and duration result from latching onto transformed versions of one or two past epidemic curves with two peaks.

### Estimated average error from cross-validation

A forecasting method’s performance can vary greatly between seasons, so a single season provides limited evaluation power. We use leave-one-out cross-validation on historical data to provide a more stable estimate of the average point prediction error from retrospective forecasts.

For each historical season *s*_cv_, we produced forecasts using the rest of the historical seasons to build the prior, and recorded the average error of our point predictions across these 15 seasons for each week in the flu season. One detail to note is that these error estimates were generated using the final revision of the wILI data, and do not include any effects from approximating the most recent wILI values from the tentative values available in real-time.

**Fig 4:**
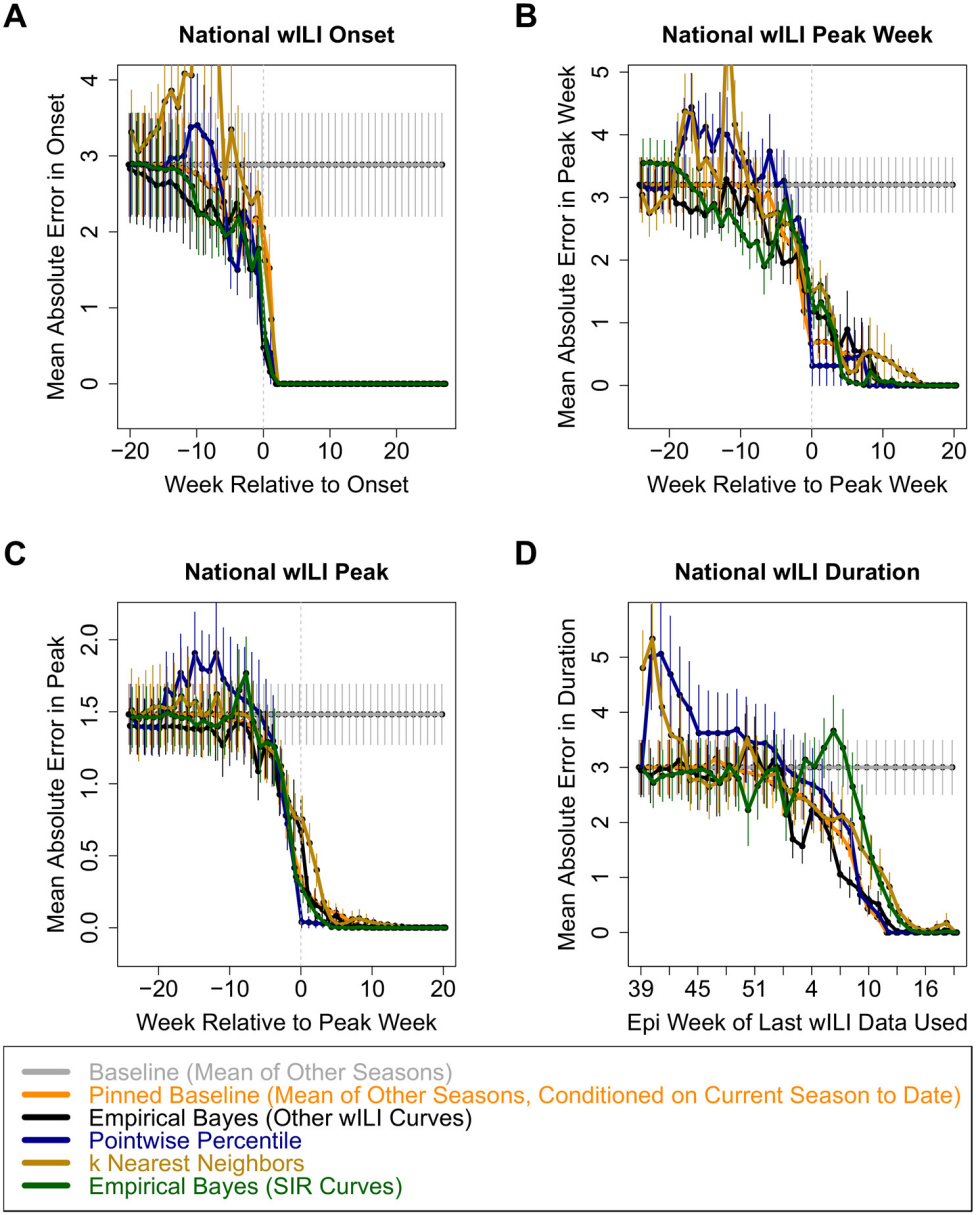
Cross-validated mean absolute error estimates and standard error bars for point predictions for (A) onset, (B) peak week, (C) peak height, and (D) duration. (The onset and duration were defined based on the 2% national threshold set by CDC for the 2013–2014 season.)

Fig 4 shows the cross-validated error for national point predictions of our current empirical Bayes framework, as well a few other approaches, for each for the four forecasting targets, aligned by the observed onset, peak week, or epidemiological week. S3 Fig shows these plots for both national and regional predictions, as well as the estimated accuracy and reliability of forecasts aligned by the point predictions for onset, peak week, and season end. S1 Table provides an alternative summary of the national cross-validation results, estimating the bias and variance of each forecasting approach, and aligning by epidemiological week. We use locally linear kernel regression [49] to estimate the mean absolute error when aligning by point predictions, GNU Parallel [50] to generate plots on multiple processors in parallel, and the xtable package [51] while typesetting the table.

We display predictions of ${\text{tar}}_{j}^{r}(y^{r,\;s_{\text{cv}}})$ for a few methods. **Baseline (Mean of Other Seasons)**: takes the average target value across the 14 other seasons, completely ignoring any data from the current season; provides an idea of whether other forecasters provide reasonable levels of error at the beginning of the season, and how much they benefit from incorporating data from the season they are forecasting. **Pinned Baseline (Mean of Other Seasons, Conditioned on Current Season to Date)**: constructs 14 possible wILI trajectories for the current season by using the available observations for previous weeks and other historical curves for future weeks; reports the mean target value across these 14 trajectories; this is another very generic baseline that allows us to see the effect of using more complex wILI models and forecasting methods. **Pointwise Percentile (P2014)** [44]: Constructs a single possible future wILI trajectory using the pointwise *q*th quantile from other seasons; estimates an appropriate value of *q* from the observed data so far, trying to match more recent observations more closely than less recent ones. *k* **Nearest Neighbors (knn)**: Uses a method similar to existing systems for shorter-term prediction [28] to identify *k* sections of other seasons’ data that best match recent observations, and uses them to construct and weight *k* possible future wILI trajectories. **Empirical Bayes (Transformed Versions of Other Seasons’ Curves)**: Our current framework, using transformed versions of other seasons’ curves to form the prior. **Empirical Bayes (SIR Curves)**: Our current framework, using scaled and shifted SIR curves rather than other seasons’ curves to form the prior; this is a somewhat similar approach to the SIRS-EAKF method used by the contest winner [20]. S5 Fig shows the fitted (not forecasted) SIR curves for national historical data, which were used to estimate a distribution over SIR, scale, and shift parameters, and S7 Fig shows two fits to regional data.

Fig 4 indicates that, for all forecasting targets and most weeks, the average point prediction error for the EB method is similar (overlapping error bars) or lower than the average error for the best predictor for that target and week. An important feature of this approach is that it provides a smooth distribution over possible curves and target values, rather than just a single point. From this distribution, we can calculate point predictions to minimize some expected type of error or loss, build credible intervals, and make probabilistic statements about future wILI and target values.

## Discussion

We developed an empirical Bayes approach to forecasting epidemic curves and targets, and applied it to wILI estimates to generate predictions for the 2013–2014 influenza season as part of a CDC challenge. Our method’s forecasts for the season were reasonable to the human eye, and cross-validated error estimates indicate that it competes with or improves upon results from various baseline predictors. This method generates a distribution over future wILI curves and forecasting targets, rather than just point predictions.

### Notes on methodology

The framework has a tendency to “latch” onto a particular shape in the mid to late season, forming predictions for the current season using transformed versions of a single past season. This phenomenon is undesirable in many cases, and was one motivation for using transformations of past curves, rather than just the curves themselves. S2 Fig illustrates how latching would be much more frequent and problematic if we did not use transformations. We find that latching occurs less frequently as more historical data becomes available, and S4 Fig shows that forecast error decreases as well.

S2 Text discusses current limitations of the framework and future work, such as ways to improve forecasts by incorporating additional types of surveillance data (e.g., Twitter activity, thermometer sales, lab tests, weather, and vaccination data), dependencies between geographical units, and more accurate models of reporting behavior (e.g., by modeling holiday effects and improving the noise model). It should also be possible to automatically select what transformations and data to use by minimizing cross-validated prediction error on historical data.

### Biology, epidemiology, and forecasting

The strength of our non-mechanistic forecasting technique is that it relies more on the raw data and less on models of how that data came about. Current biological and epidemiological models of influenza, while grounded in 100 years of significant theoretical and mathematical development, still neglect or grossly simplify much that is not well understood or not yet well estimated, including subtype cross-protection, spatial dynamics, and demographic, behavioral and climatic conditions. Furthermore, since true influenza incidence is not known even in hindsight, we fall back on forecasting wILI. But to do so mechanistically, a large number of other processes must be understood and estimated, including the contributions of and interactions with non-influenza respiratory illnesses, the non iid nature of ILINet, variability in medical care seeking among both ILI and non-ILI patients, and more. In our opinion, these complications make non-mechanistic methods an attractive starting point for developing forecasting technology. The flip side of this approach is that our methods provide only modest insight into the biological and epidemiological processes underlying influenza. To boot:

- The usefulness of time-shifting the wILI curves confirms our intuition that the same week-on-week dynamics of influenza can be at play at different times of the year in different seasons.
- The usefulness of the Empirical Bayes approach in general suggests that the universe of wILI curves may well differ substantially from the conventionally parameterized compartmental models, likely due to the complex interaction of subtypes, the presence on non-influenza ILI, and the spread dynamics over the large regions involved.
- Our analysis revealed the Holiday Effect (mentioned in the discussion of the distributional and point predictions for the 2013–2014 season, and visible in S5, S7 and S8 Figs) as a systematic and significant phenomenon in current wILI surveillance data. This effect consists of both a drop in the number of non-ILI office visits, as well as (in some seasons) a rise in the absolute number of ILI office visits, during the major holidays. While it is not unexpected that non-acute office visits are down during the holiday period, a deeper investigation of acute-care seeking behavior may be called for. Regardless, both these phenomena should be accounted for in any modeling or forecasting approach that uses this data.
- The fact that our method’s accuracy continues to improve with more historical seasons (see S4 Fig) suggests that the universe of wILI curves is not adequately sampled with 15 seasons, and that adding more seasons as they become available will likely further improve our method’s accuracy.

Since the presented framework models epidemic curves rather than the underlying epidemiological process, it can be more readily applied to similar settings than complex mechanistic models which require adjustment based on some of the factors listed above. We have already used it to predict dengue incidence in the 2014 World Cup game cities with little modification [44], and expect that application to additional diseases with semi-regular seasonal outbreaks would require little adjustment, and could be considered as a baseline for other, more specialized, predictors; it would not, however, apply to diseases with non-seasonal behavior, emerging diseases or invasion scenarios.

While we provide detailed analysis of our method’s accuracy in the supporting information, to be useful to decision makers, the accuracy and reliability of any method must be distilled down to a few numbers. How should accuracy results be aggregated across regions, seasons, and time-of-forecast? This is a non-trivial question for forecasting time varying events. Aggregating as function of time relative to the events occurrence (as in S3A Fig) is useful for analysis, but is not actionable because the time of the event is not known at forecast time. Aggregating as a function of absolute time (as in S1 Table), while actionable, is not very useful for events whose timing varies considerably from season to season and from region to region. Perhaps the most useful way to aggregate accuracy results is by predicted-time-to-event (as in S3B Fig). Whether these forecasts are already sufficient to influence action regarding influenza in the US or else must first be further improved is a question best left to public health officials, but it is our hope that by offering our methods and cross validated results we will both enrich the growing body of forecasting technologies and stimulate others to publish the results of their methods on these same test sets, targets, and metrics.

## Supporting Information

S1 Fig Weekly forecasts for the 2013–2014 season. Begins with week 41, using only preseason data (through 2013 week 39), and ends with week 22, when the entire season (through 2014 week 20) has been observed.
(PDF)

S2 Fig Week 49 predictions with and without using transformations in prior. The forecast without transformations is composed almost entirely of trajectories that are nearly identical to one of three past seasons. The forecast with transformations contains a much wider variety of curves.
(PDF)

S3 Fig Cross-validated error plots for national and regional point predictions. We used leave-one-out cross-validation to estimate the expected error of the point predictors discussed. This figure contains plots of (A) the average point prediction error against epidemic weeks aligned by the observed onset and peak week, (B) the point prediction error distribution and mean against predicted onset and peak week, and (C) the probability of point predictions being within certain thresholds of the observed values against predicted onset and peak week, for the four forecasting targets, both nationally and regionally, for EB and the other forecasters described in the cross-validation section.
Estimation of the mean in (B) and probabilities in (C) is performed using locally linear kernel regression [49]. This series of plots was generated with a script that used GNU Parallel to generate plots in parallel on multiple processors [50].
(PDF)

S4 Fig Cross-validation study of effects of using less seasons to form prior. We applied the EB framework using randomly selected subsets of the available seasons to see how many seasons are needed for passable forecast results, and how prediction accuracy improves with additional data. This figure contains plots of (A) the average point prediction error against epidemic weeks aligned by the observed onset and peak week, (B) the coverage of 90% credible intervals against epidemic weeks aligned by the observed onset and peak week, (C) the point prediction error distribution and mean against predicted onset and peak week, and (D) the probability of point predictions being within certain thresholds of the observed values against predicted onset and peak week, for the four forecasting targets, both nationally and regionally, for EB using 2, 4, 8, or 15 other wILI curves to form the prior.
Estimation of the mean in (C) and probabilities in (D) is performed using locally linear kernel regression [49]. This series of plots was generated with a script that used GNU Parallel to generate plots in parallel on multiple processors [50].
(PDF)

S5 Fig Curves fitted by trend filtering and SIR model. Contains the national wILI trajectory for each historical season, and the curves fit by (A) trend filtering, which are transformed to form the EB prior over curves, and (B) the single-strain fully-mixed affine-observation SIR model, which are used by EB-SIR to form a prior over SIR parameters.
(PDF)

S6 Fig Diagram of the generation process for ILINet and GFT data. We are interested in influenza and other ILI incidence, but cannot observe them directly. Instead, we rely on wILI as a measure of flu prevalence, and sometimes use GFT to approximate wILI. Shaded nodes, unobserved quantities; shaded dashed nodes, proprietary data; unshaded nodes, publicly available data; thin arrows, dependencies; thick arrows, deterministic dependencies.
(PDF)

S7 Fig Trend filtering, SIR, and smoothing spline fits for HHS region 3 for two seasons. The quadratic trend filtering fit was performed with the cv.trendfilter [41] method, which automatically selects a level of smoothness to use. The cubic natural smoothing spline fit was produced by smooth.spline [46], which also automatically selects a level of smoothness, but by different criteria. (A) 2008–2009 season: trend filtering and smoothing splines both smooth out the holiday effects. The smoothing spline appears to overfit to noise in the preseason and early flu season. (B) 2006–2007 season: in addition to holiday effects, there is a large jump in wILI at week 40, which coincides with the beginning of the influenza season and a large jump in the number of reporting providers (from about 30 to over 100). The trend filtering procedure has trouble matching the beginning-of-season and holiday effects, attributing most of these effects to noise and smoothing them out. The smooth.spline procedure selects a level of smoothness that essentially duplicates the observed wILI and would produce a noise estimate near 0, which does not seem appropriate. Alternative methods of selecting a level of smoothness may produce looser fits and avoid these near-0 noise estimates, though.
Beginning-of-season and holiday effects can be incorporated in both of the smoothing procedures, and would likely improve the resulting fits. Regional wILI dynamics are generally not tightly fit by the described SIR model.
(PDF)

S8 Fig National and regional residuals after trend-filtering fit. We took the difference between the observed wILI and trend-filtered fit for each historical season and region, and put them on the same scale by dividing by the standard deviation of residuals in the corresponding season and region (giving a *z*-score). (A) A histogram of the scaled residuals, together with a histogram for a standard normal random sample of the same size. (B) The empirical cumulative distribution function of the residuals, together with the cumulative distribution function of a standard normal random variable. (C,D,E) The (unscaled) residuals for the trends filtering fits to the national data for the (C) 2010–2011, (D) 2011–2012, and (E) 2012–2013 seasons. The trend filtering fits corresponding to (C), (D), and (E) are shown in S5 Fig. The winter holiday effect is unusually pronounced in (E), while (C) and (D) show more typical residual patterns. The trend-filtering residuals do not look exactly normally distributed; deviations from normality may be due to holiday effects, autocorrelation between residuals, and/or non-normality of wILI measurements.
(PDF)

S1 Table Leave-one-out cross-validated bias and variance estimates, aligned by epidemiological week. Most years are assigned 52 epidemiological weeks, but a few are assigned 53; we examine prediction error at weeks 39–52 and 1–20 when the first year of a season has 52 weeks, and weeks 39–53 and 1–19 when the first year has 53 weeks. Predictors are assigned the following abbreviations: “bl”, baseline (mean of other seasons); “splice”, pinned baseline (mean of other seasons, conditioned on current season to date); “eb”, empirical Bayes framework, using other seasons’ wili curves; “per”, pointwise percentile method; “knn”, *k*-nearest neighbors method; “ebsir”, empirical Bayes framework, modified to use SIR curves. Predictions were generated as if the final revision of the wILI data was available immediately. The week number in the leftmost column is the epidemiological week of the latest wILI measurement revealed to the predictors; EW39 corresponds to using no wILI values from the left-out season. All units of measurement are omitted. This table was generated using the xtable package [51].
(PDF)

S1 Text Importance sampling algorithm. Pseudocode for one algorithm used to build a collection of possible futures for the current season.
(PDF)

S2 Text Current limitations and future work. (PDF)
