# Supplementary material for: Flexible Modeling of Epidemics with an Empirical Bayes Framework
Source: PLoS Comput Biol. 2015 Aug 28;11(8):e1004382. doi: 10.1371/journal.pcbi.1004382 (PMC4552841; doi:10.1371/journal.pcbi.1004382)
Supplement: S1 Fig — Begins with week 41, using only preseason data (through 2013 week 39), and ends with week 22, when the entire season (through 2014 week 20) has been observed. (PDF) [file pcbi.1004382.s001.pdf]

Weekly forecasts for the 2013–2014 season

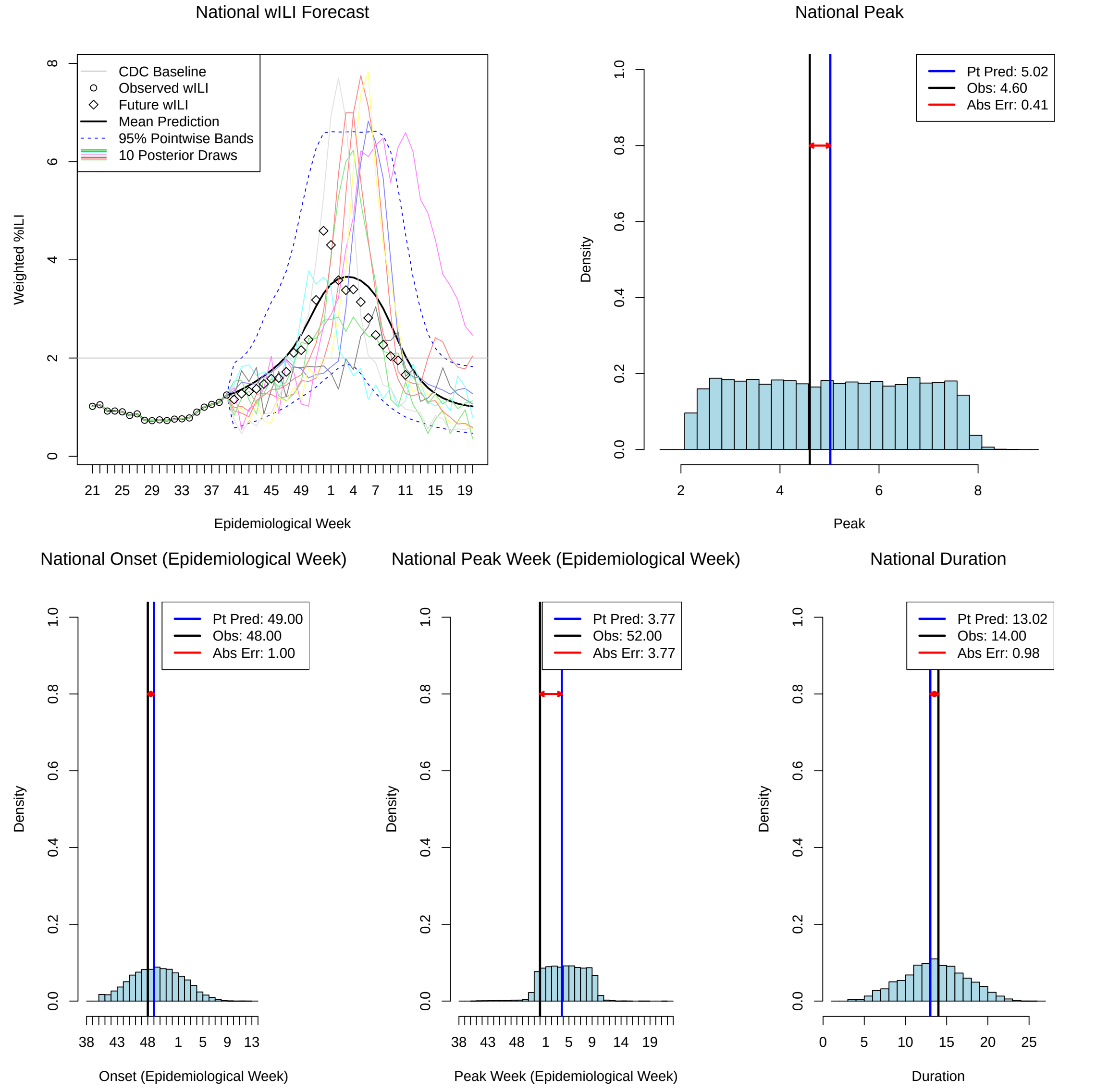

2013–2014 National Forecast, Week 41, using the current framework and the final wILI values. The wILI observations for the week of the forecast and preceding week, are not used in the forecast. “Pt Pred” (point prediction), the posterior mean target value; “Obs”, the observed value, “Abs Err”, the absolute error of the point prediction.

Weekly forecasts for the 2013–2014 season

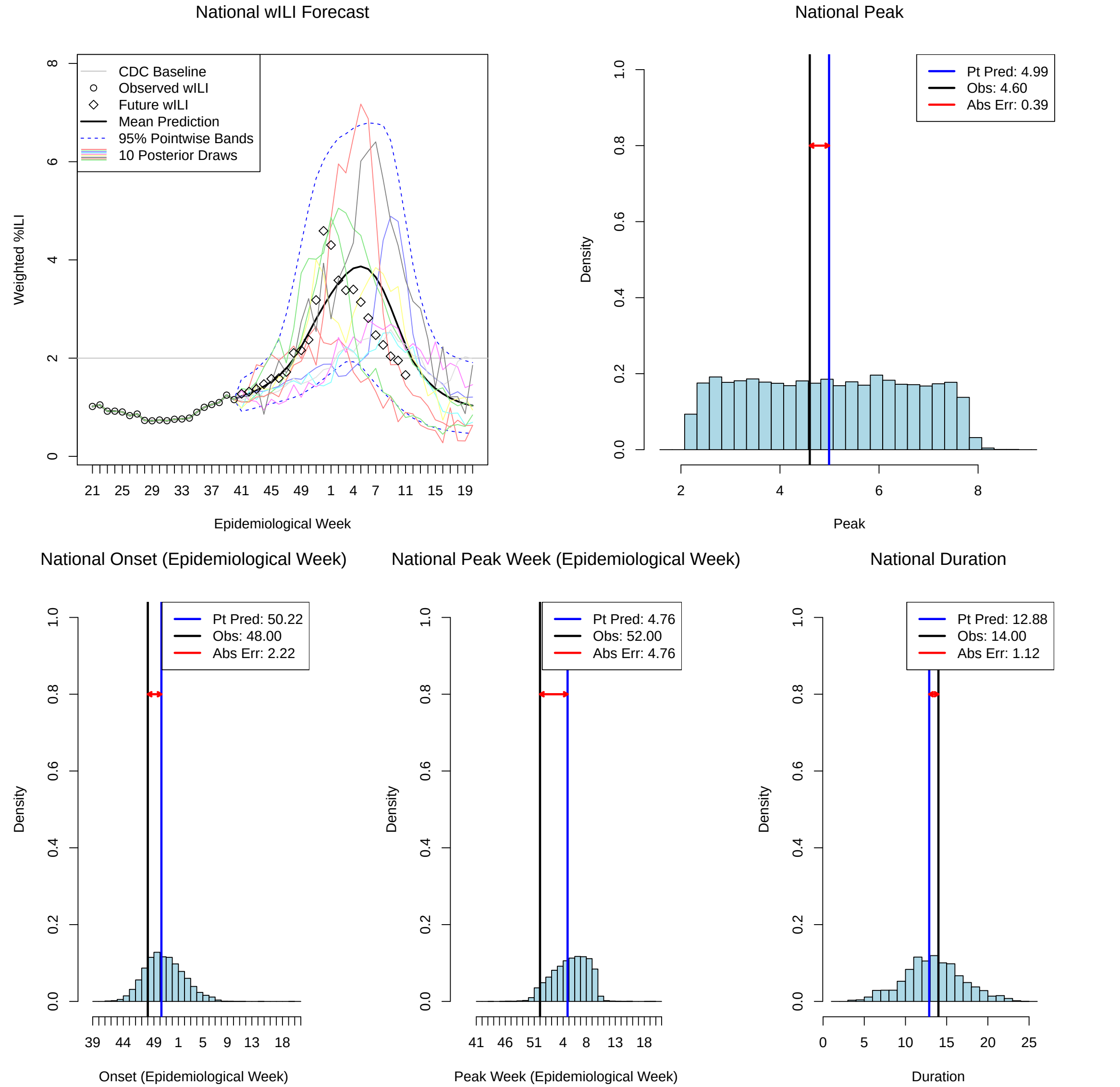

2013–2014 National Forecast, Week 42, using the current framework and the final wILI values. The wILI observations for the week of the forecast and preceding week, are not used in the forecast. "Pt Pred" (point prediction), the posterior mean target value; "Obs", the observed value, "Abs Err", the absolute error of the point prediction.

Weekly forecasts for the 2013–2014 season

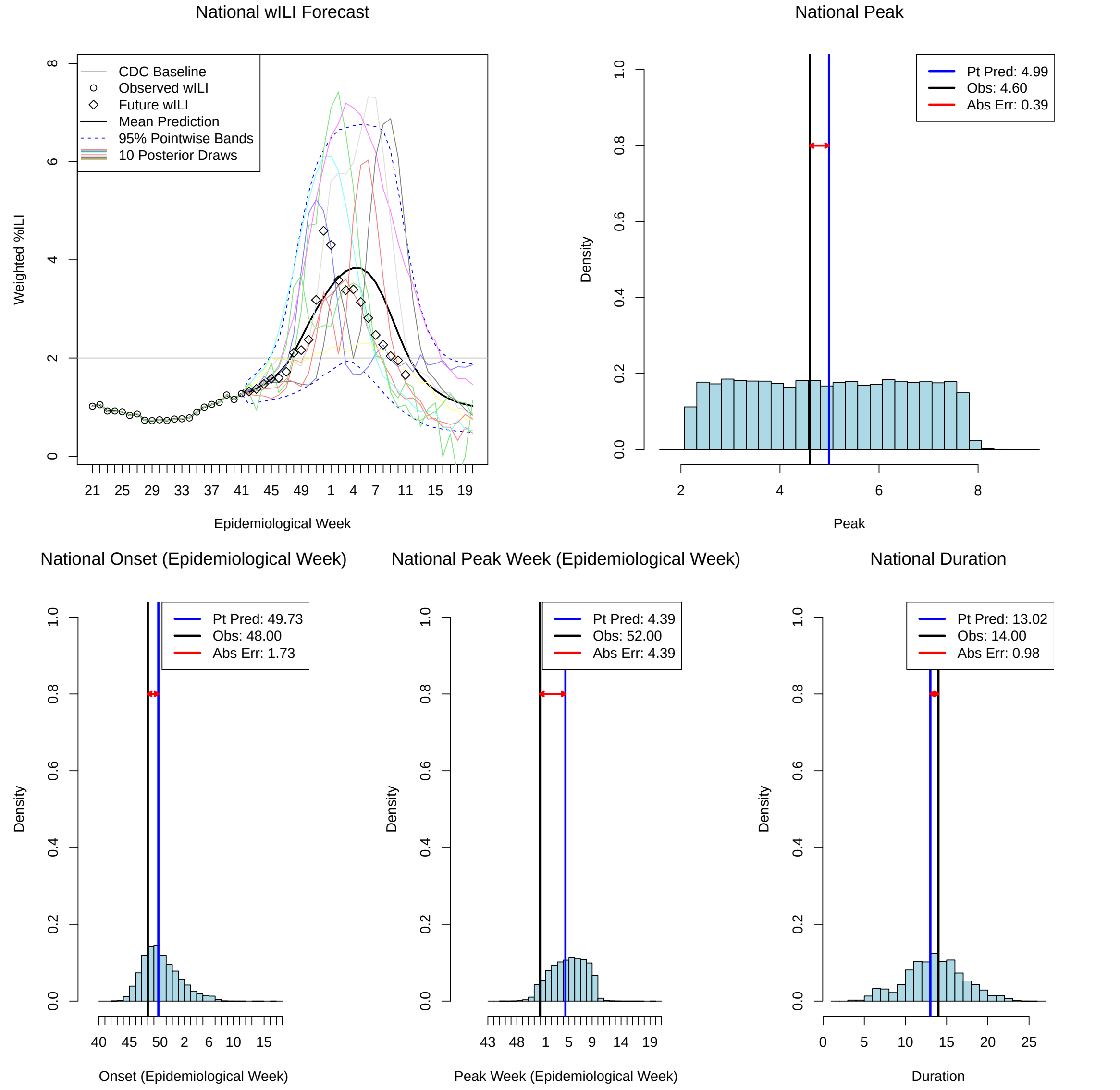

2013–2014 National Forecast, Week 43, using the current framework and the final wILI values. The wILI observations for the week of the forecast and preceding week, are not used in the forecast. "Pt Pred" (point prediction), the posterior mean target value; "Obs", the observed value, "Abs Err", the absolute error of the point prediction.

Weekly forecasts for the 2013–2014 season

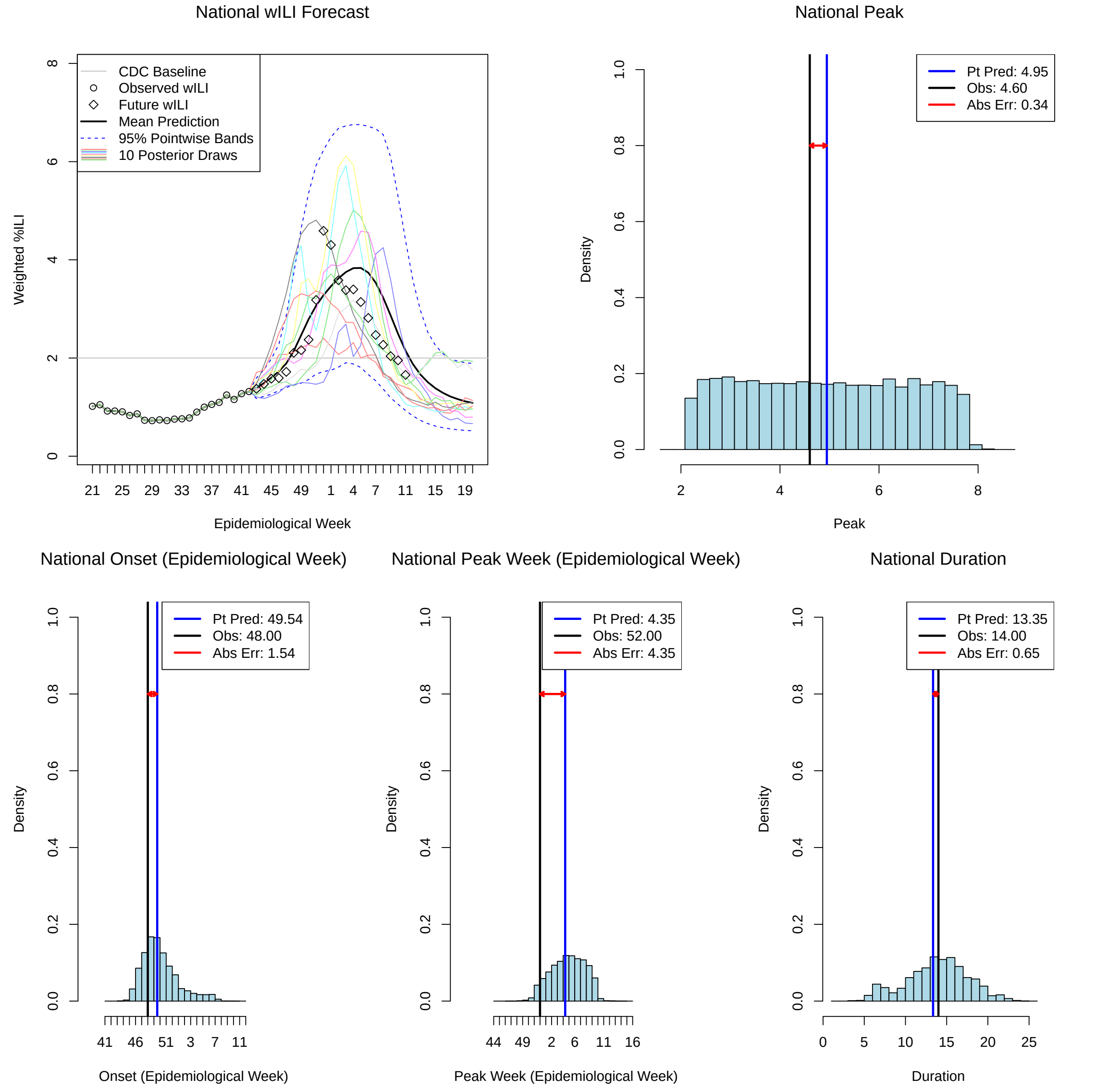

2013–2014 National Forecast, Week 44, using the current framework and the final wILI values. The wILI observations for the week of the forecast and preceding week, are not used in the forecast. "Pt Pred" (point prediction), the posterior mean target value; "Obs", the observed value, "Abs Err", the absolute error of the point prediction.

Weekly forecasts for the 2013–2014 season

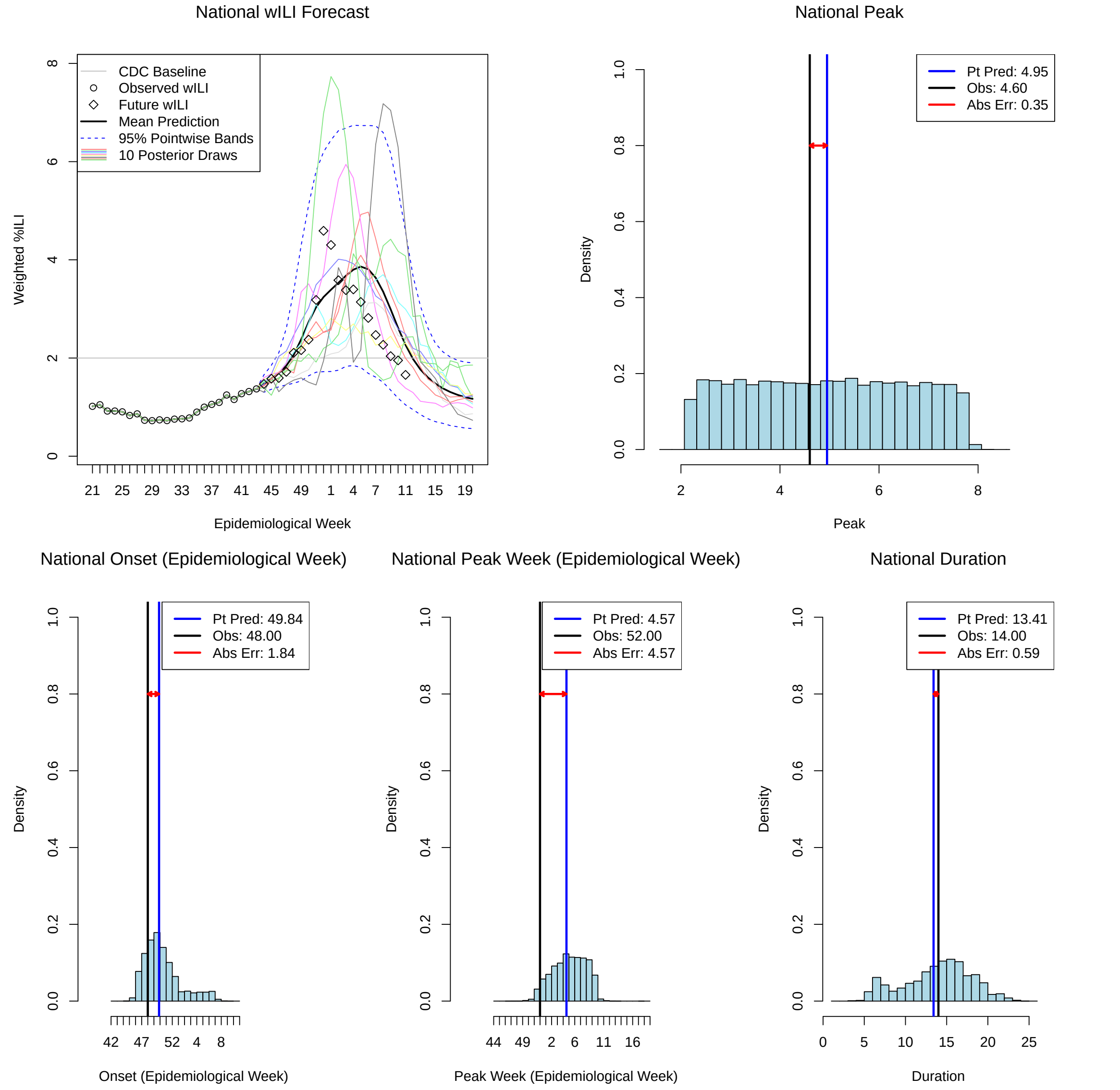

2013–2014 National Forecast, Week 45, using the current framework and the final wILI values. The wILI observations for the week of the forecast and preceding week, are not used in the forecast. "Pt Pred" (point prediction), the posterior mean target value; "Obs", the observed value, "Abs Err", the absolute error of the point prediction.

Weekly forecasts for the 2013–2014 season

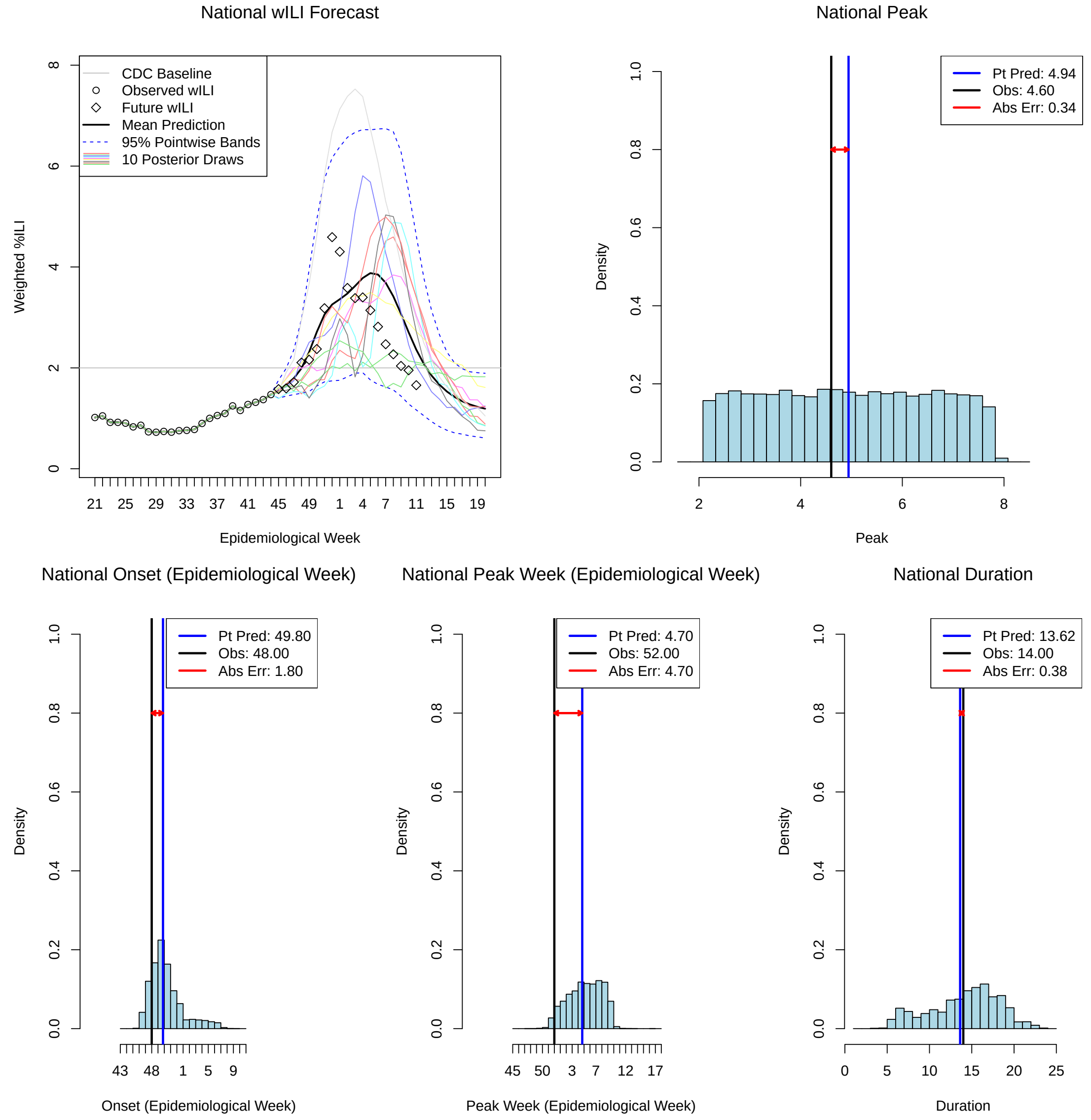

2013–2014 National Forecast, Week 46, using the current framework and the final wILI values. The wILI observations for the week of the forecast and preceding week, are not used in the forecast. "Pt Pred" (point prediction), the posterior mean target value; "Obs", the observed value, "Abs Err", the absolute error of the point prediction.

Weekly forecasts for the 2013–2014 season

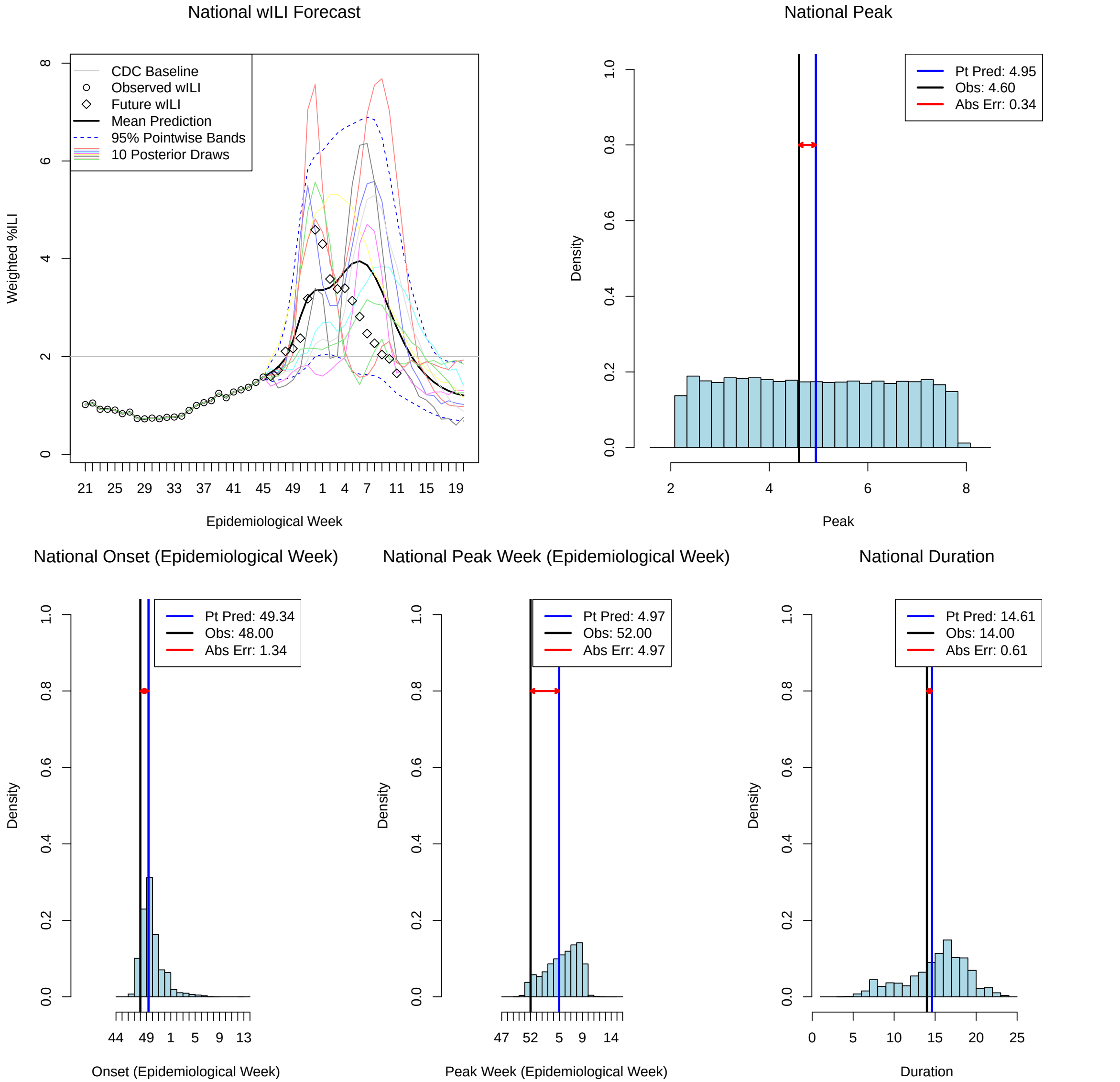

2013–2014 National Forecast, Week 47, using the current framework and the final wILI values. The wILI observations for the week of the forecast and preceding week, are not used in the forecast. "Pt Pred" (point prediction), the posterior mean target value; "Obs", the observed value, "Abs Err", the absolute error of the point prediction.

Weekly forecasts for the 2013–2014 season

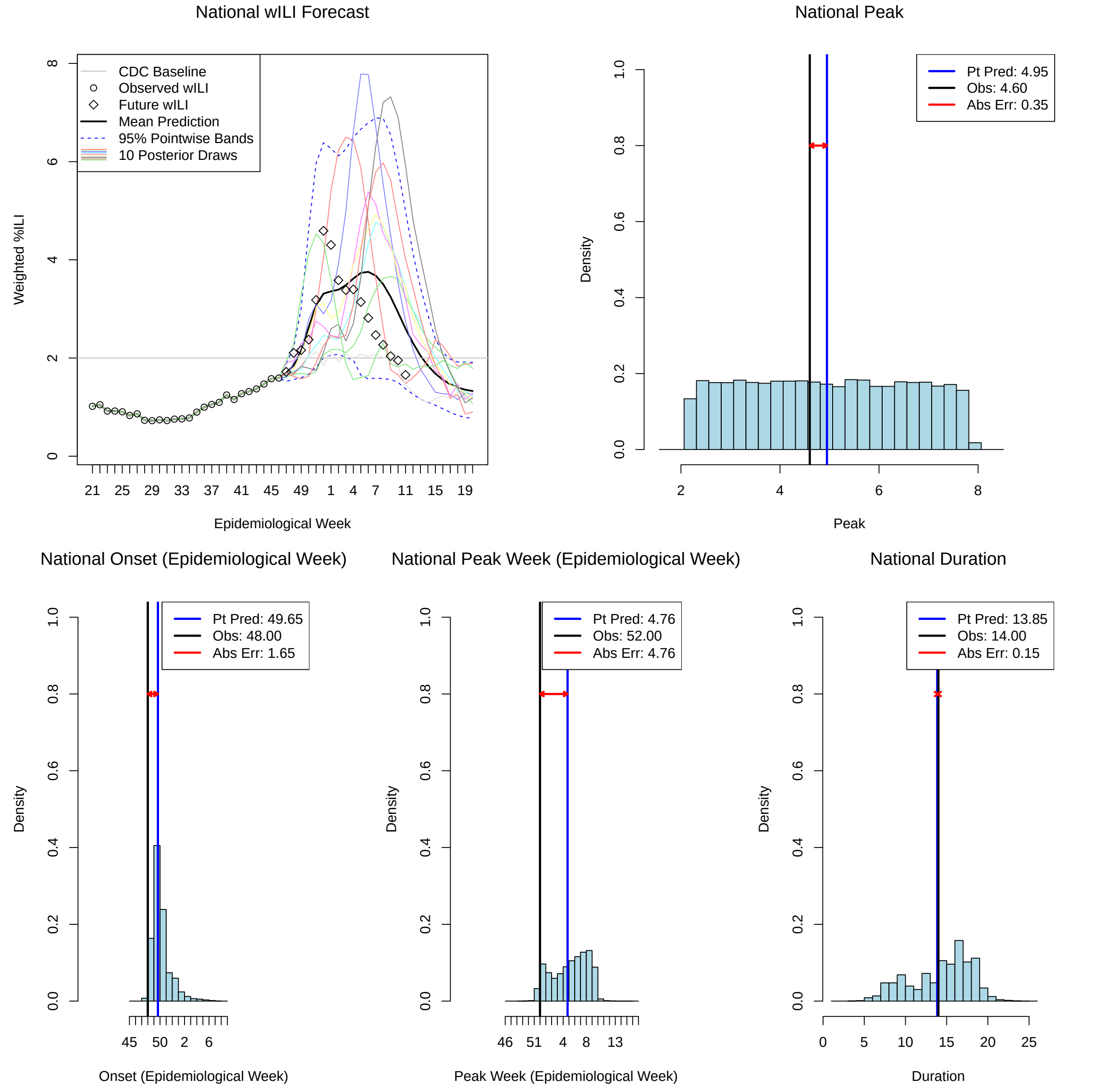

2013–2014 National Forecast, Week 48, using the current framework and the final wILI values. The wILI observations for the week of the forecast and preceding week, are not used in the forecast. "Pt Pred" (point prediction), the posterior mean target value; "Obs", the observed value, "Abs Err", the absolute error of the point prediction.

Weekly forecasts for the 2013–2014 season

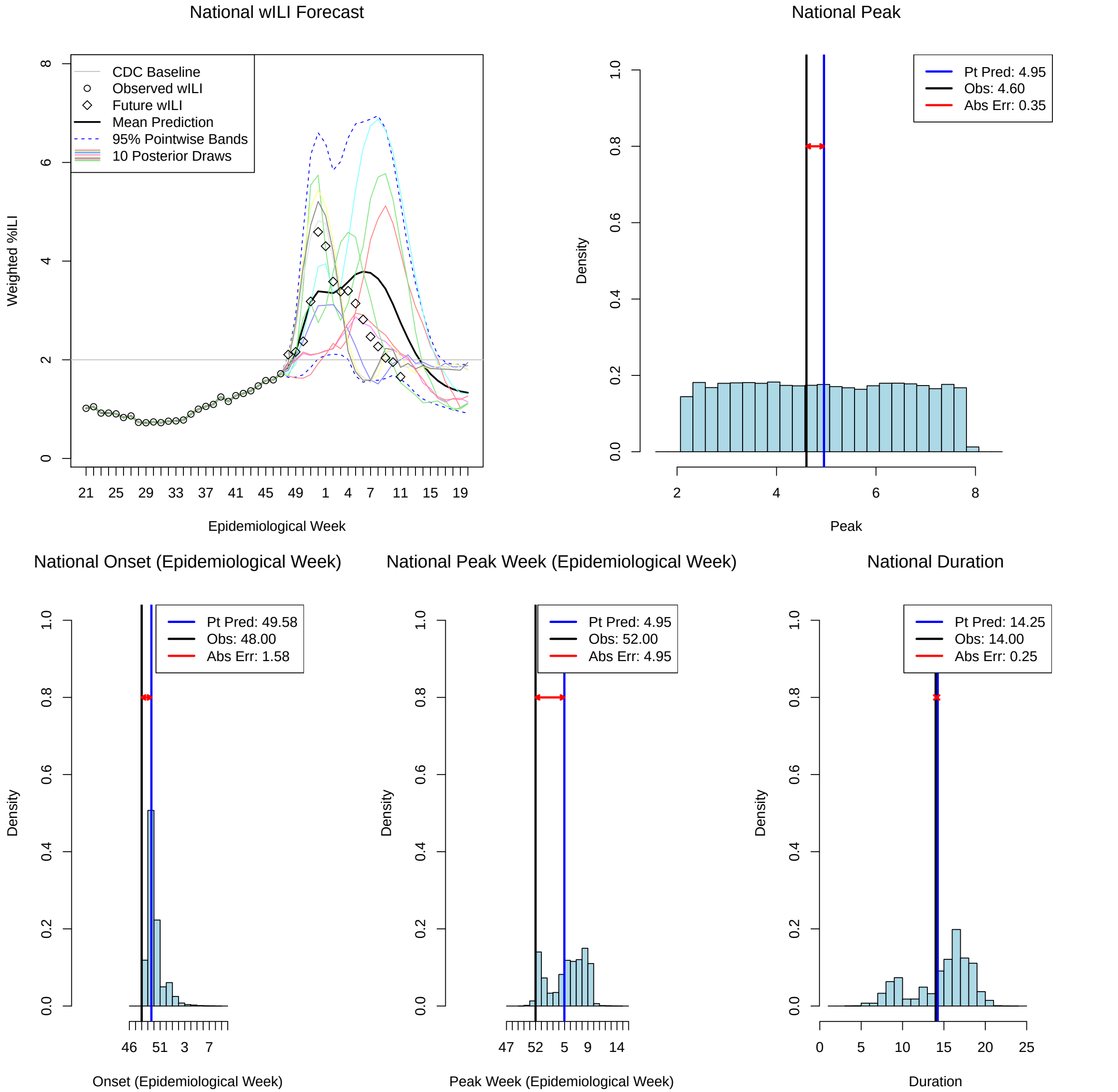

2013–2014 National Forecast, Week 49, using the current framework and the final wILI values. The wILI observations for the week of the forecast and preceding week, are not used in the forecast. "Pt Pred" (point prediction), the posterior mean target value; "Obs", the observed value, "Abs Err", the absolute error of the point prediction.

Weekly forecasts for the 2013–2014 season

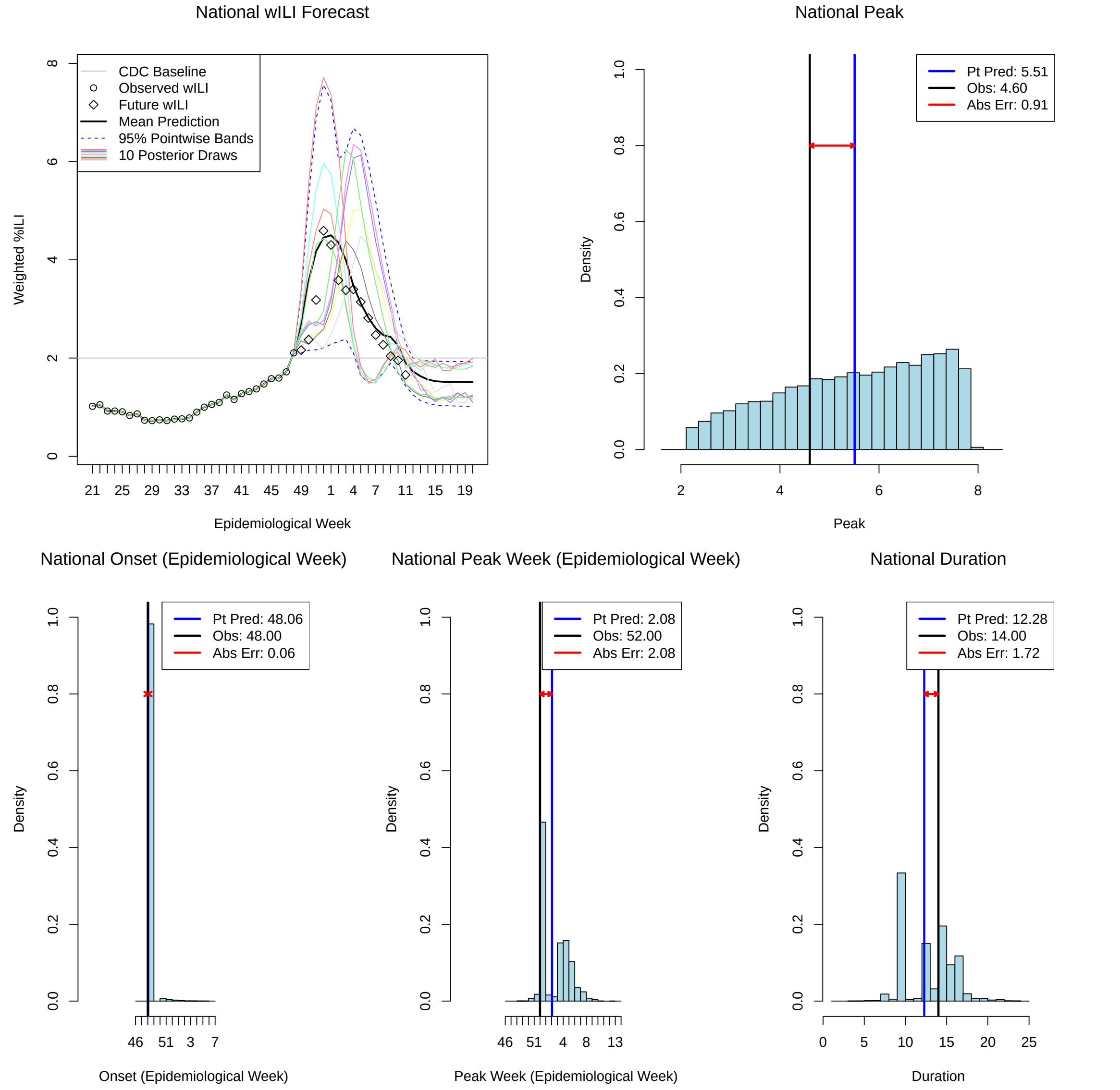

2013–2014 National Forecast, Week 50, using the current framework and the final wILI values. The wILI observations for the week of the forecast and preceding week, are not used in the forecast. “Pt Pred” (point prediction), the posterior mean target value; “Obs”, the observed value, “Abs Err”, the absolute error of the point prediction.

Weekly forecasts for the 2013–2014 season

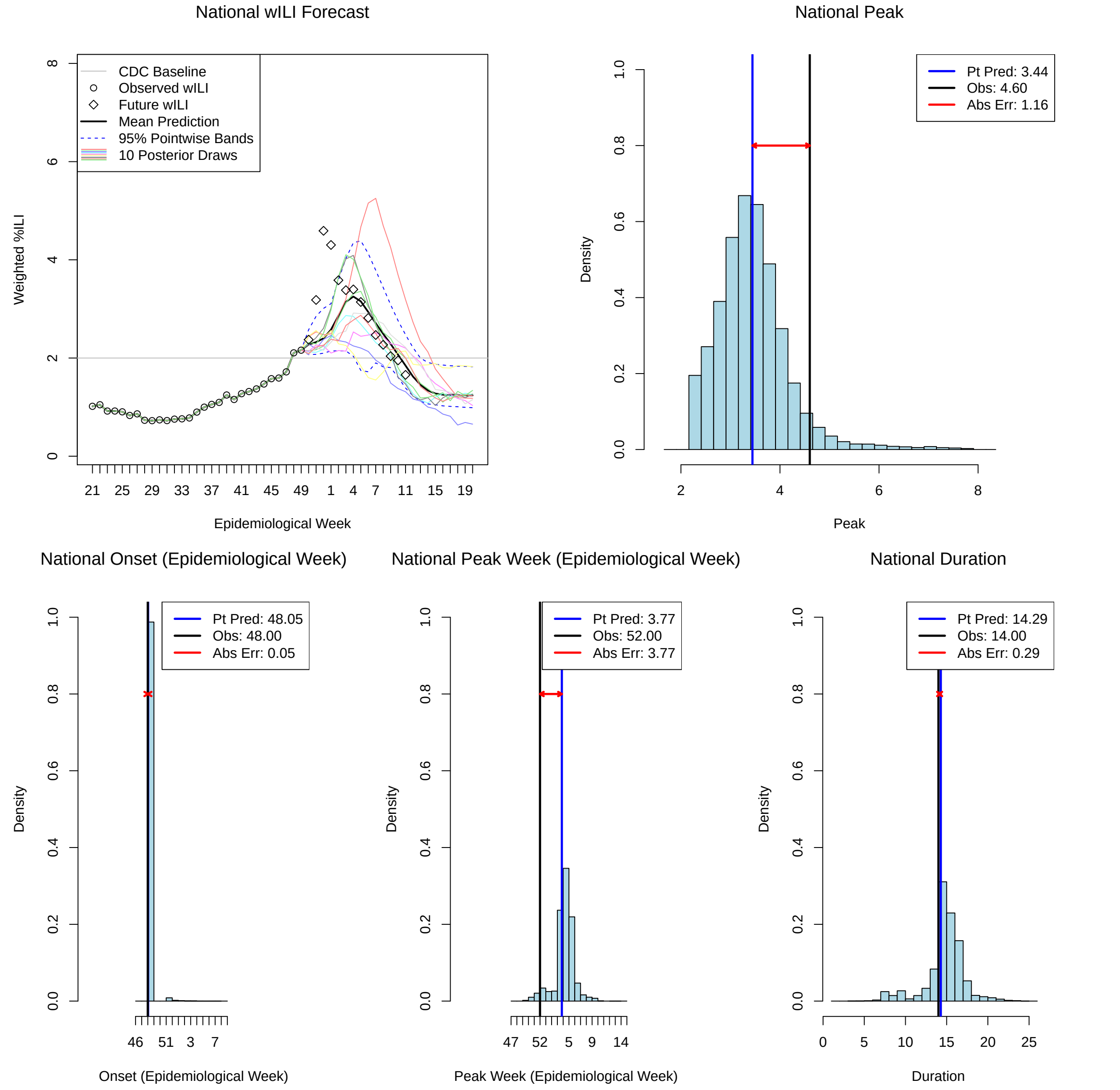

2013–2014 National Forecast, Week 51, using the current framework and the final wILI values. The wILI observations for the week of the forecast and preceding week, are not used in the forecast. "Pt Pred" (point prediction), the posterior mean target value; "Obs", the observed value, "Abs Err", the absolute error of the point prediction.

Weekly forecasts for the 2013–2014 season

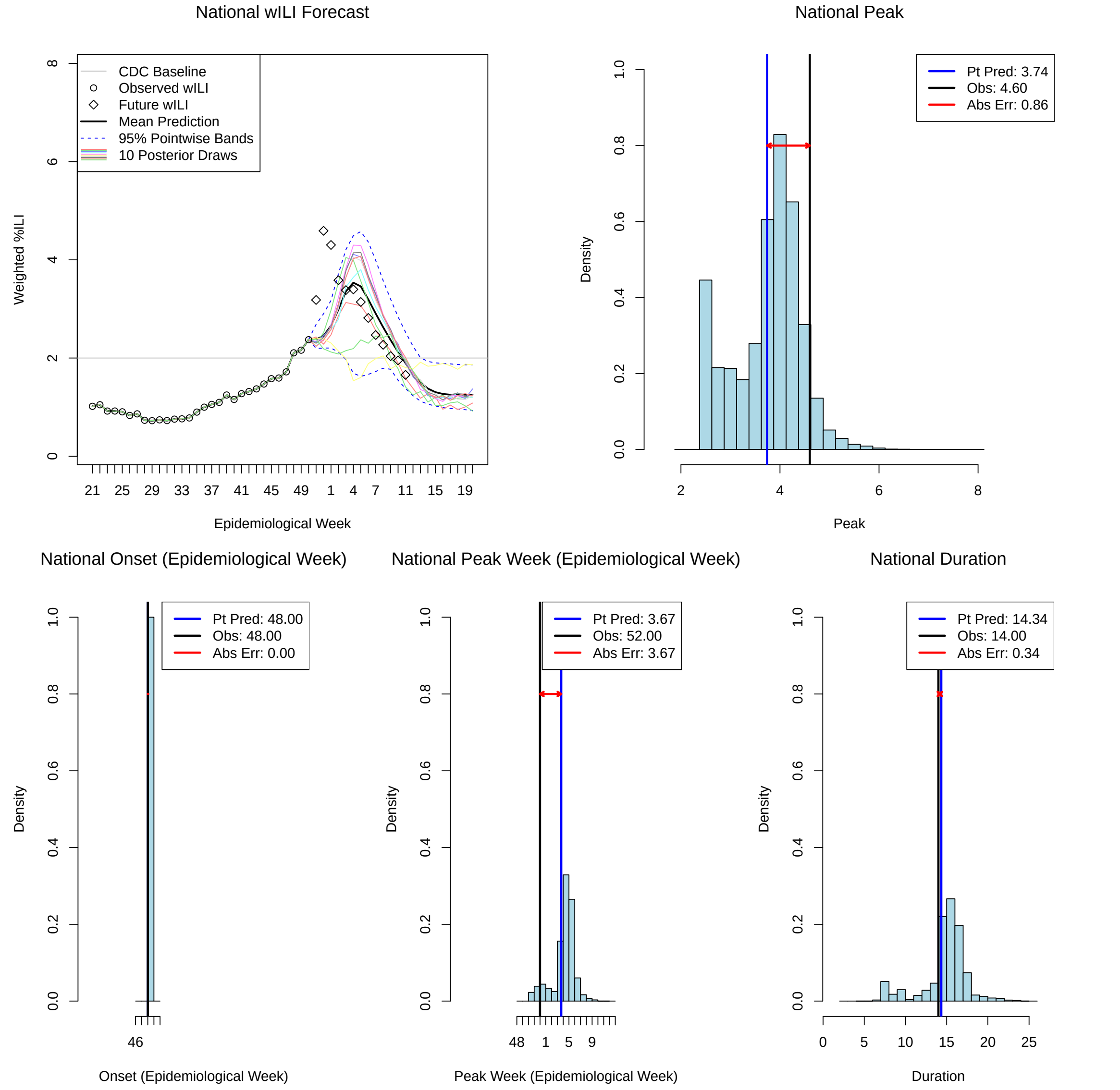

2013–2014 National Forecast, Week 52, using the current framework and the final wILI values. The wILI observations for the week of the forecast and preceding week, are not used in the forecast. "Pt Pred" (point prediction), the posterior mean target value; "Obs", the observed value, "Abs Err", the absolute error of the point prediction.

Weekly forecasts for the 2013–2014 season

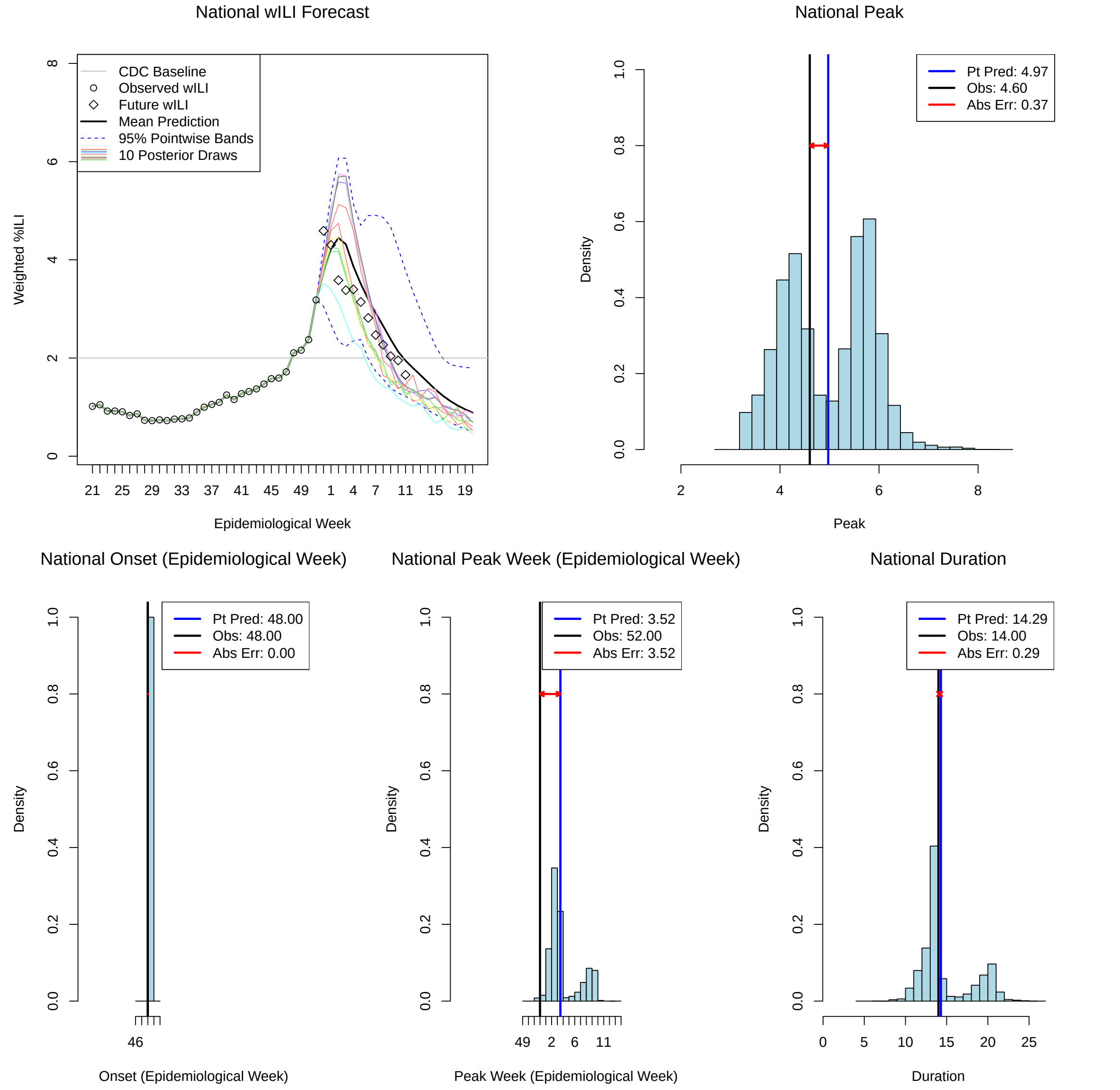

2013–2014 National Forecast, Week 1, using the current framework and the final wILI values. The wILI observations for the week of the forecast and preceding week, are not used in the forecast. "Pt Pred" (point prediction), the posterior mean target value; "Obs", the observed value, "Abs Err", the absolute error of the point prediction.

Weekly forecasts for the 2013–2014 season

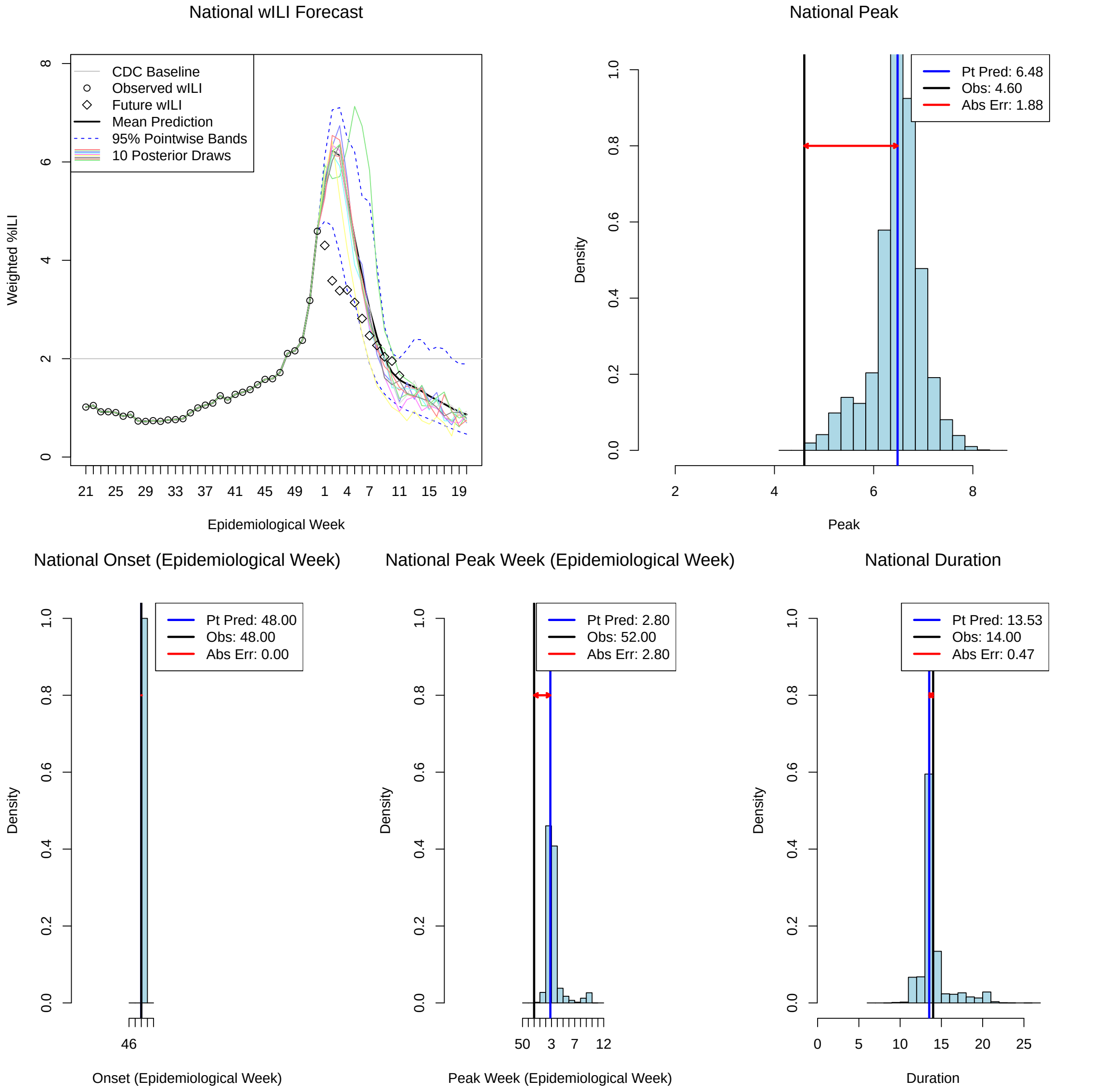

2013–2014 National Forecast, Week 2, using the current framework and the final wILI values. The wILI observations for the week of the forecast and preceding week, are not used in the forecast. "Pt Pred" (point prediction), the posterior mean target value; "Obs", the observed value, "Abs Err", the absolute error of the point prediction.

Weekly forecasts for the 2013–2014 season

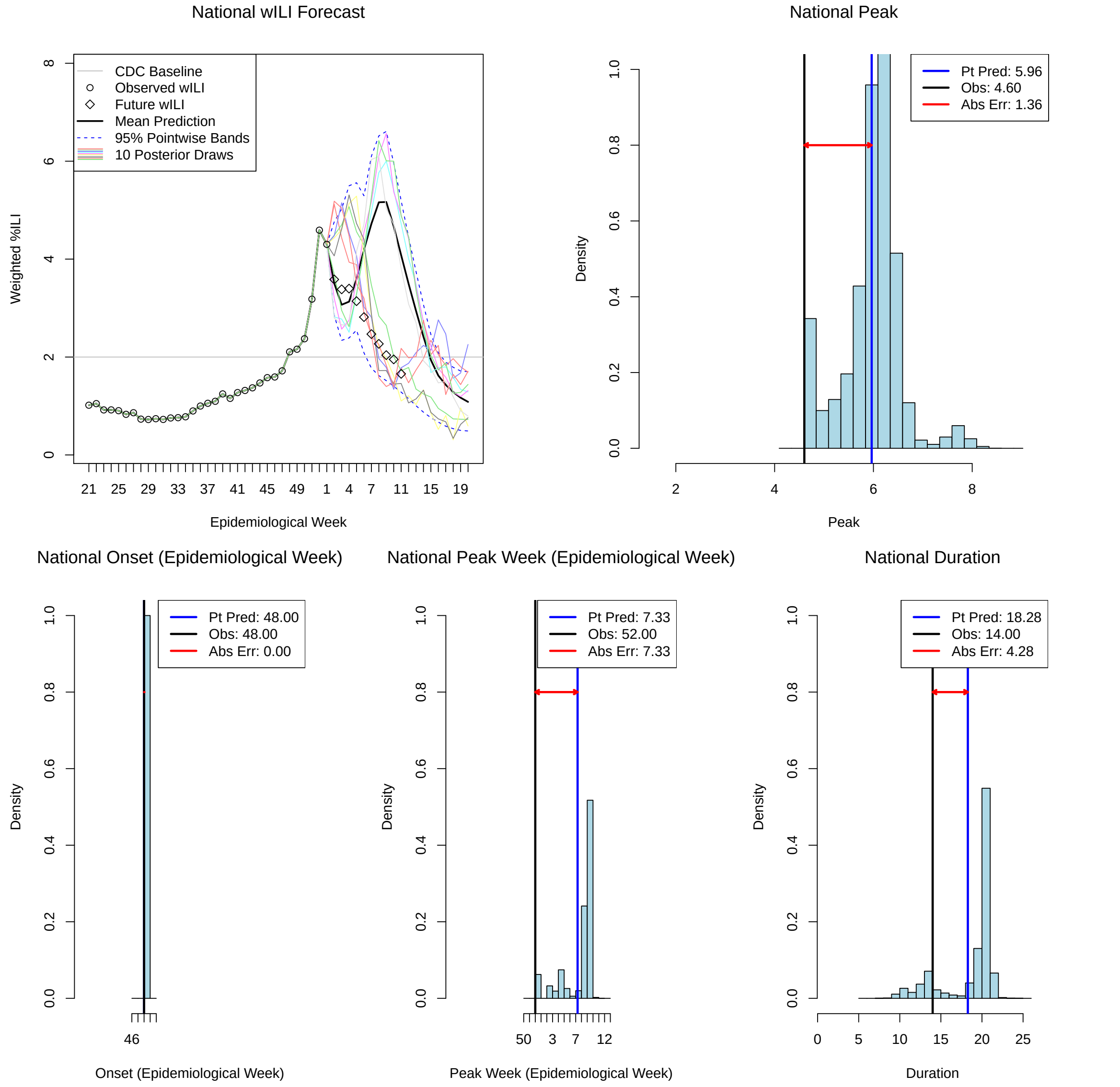

2013–2014 National Forecast, Week 3, using the current framework and the final wILI values. The wILI observations for the week of the forecast and preceding week, are not used in the forecast. "Pt Pred" (point prediction), the posterior mean target value; "Obs", the observed value, "Abs Err", the absolute error of the point prediction.

Weekly forecasts for the 2013–2014 season

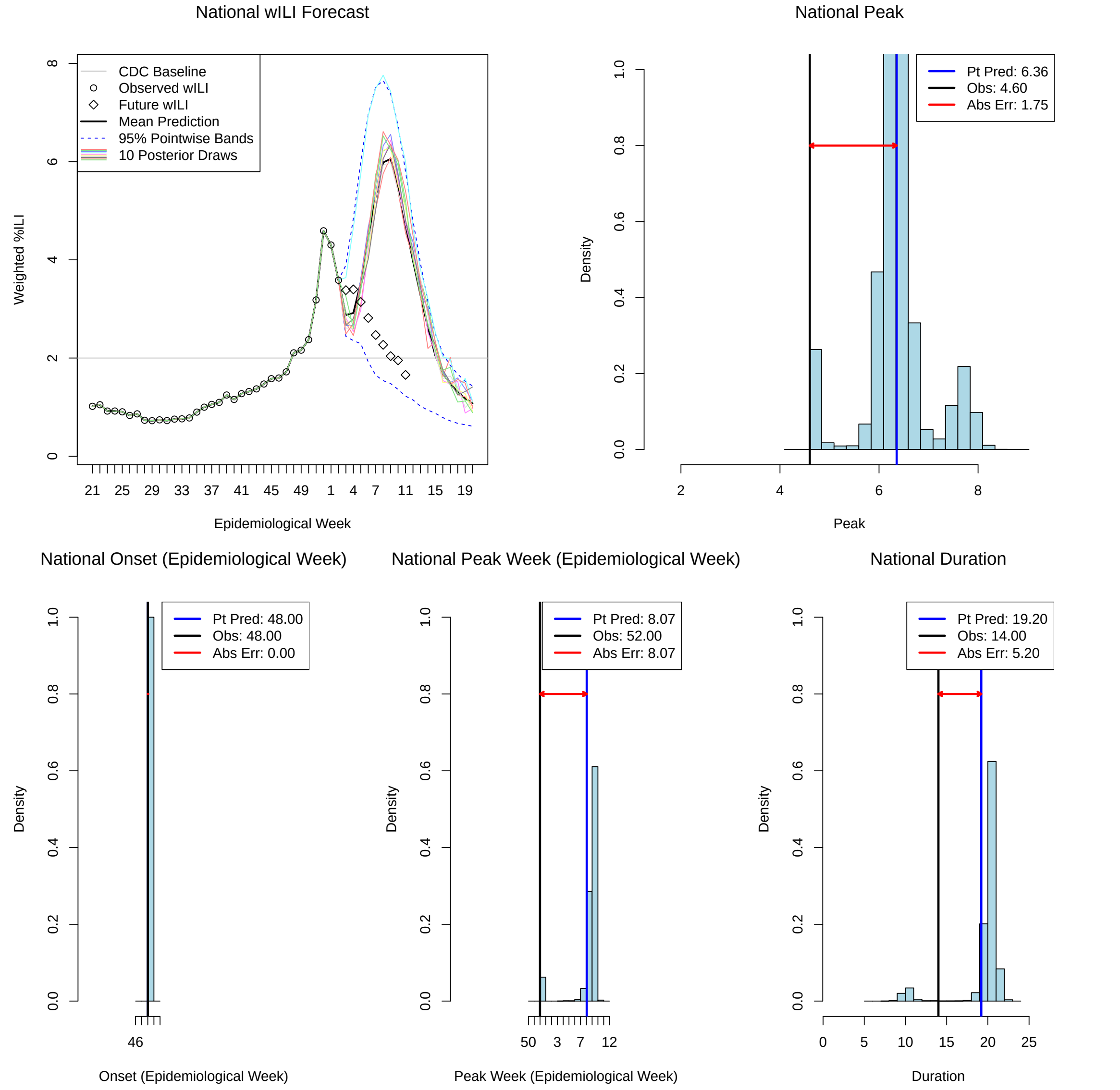

2013–2014 National Forecast, Week 4, using the current framework and the final wILI values. The wILI observations for the week of the forecast and preceding week, are not used in the forecast. "Pt Pred" (point prediction), the posterior mean target value; "Obs", the observed value, "Abs Err", the absolute error of the point prediction.

Weekly forecasts for the 2013–2014 season

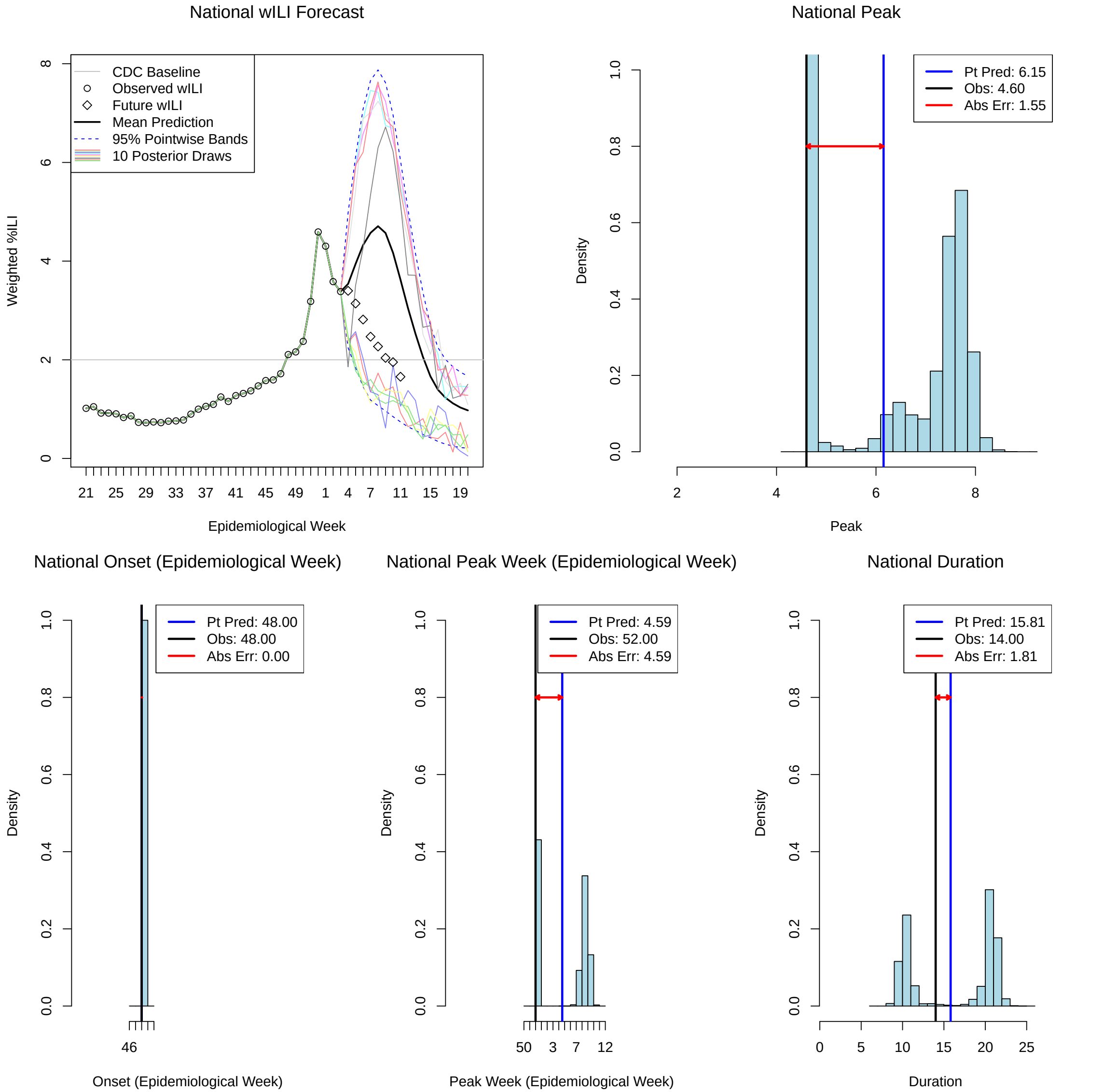

2013–2014 National Forecast, Week 5, using the current framework and the final wILI values. The wILI observations for the week of the forecast and preceding week, are not used in the forecast. "Pt Pred" (point prediction), the posterior mean target value; "Obs", the observed value, "Abs Err", the absolute error of the point prediction.

Weekly forecasts for the 2013–2014 season

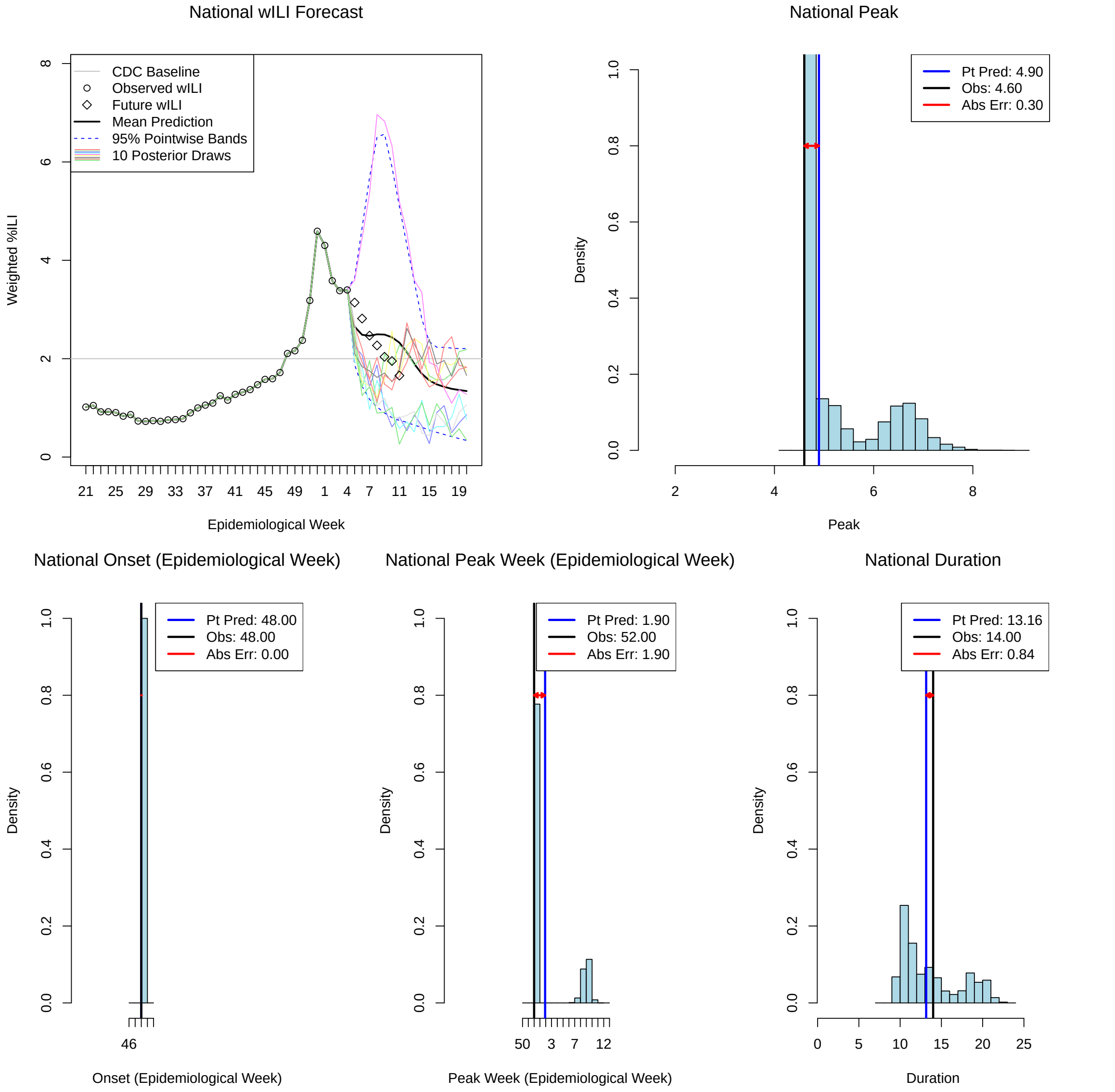

2013–2014 National Forecast, Week 6, using the current framework and the final wILI values. The wILI observations for the week of the forecast and preceding week, are not used in the forecast. "Pt Pred" (point prediction), the posterior mean target value; "Obs", the observed value, "Abs Err", the absolute error of the point prediction.

Weekly forecasts for the 2013–2014 season

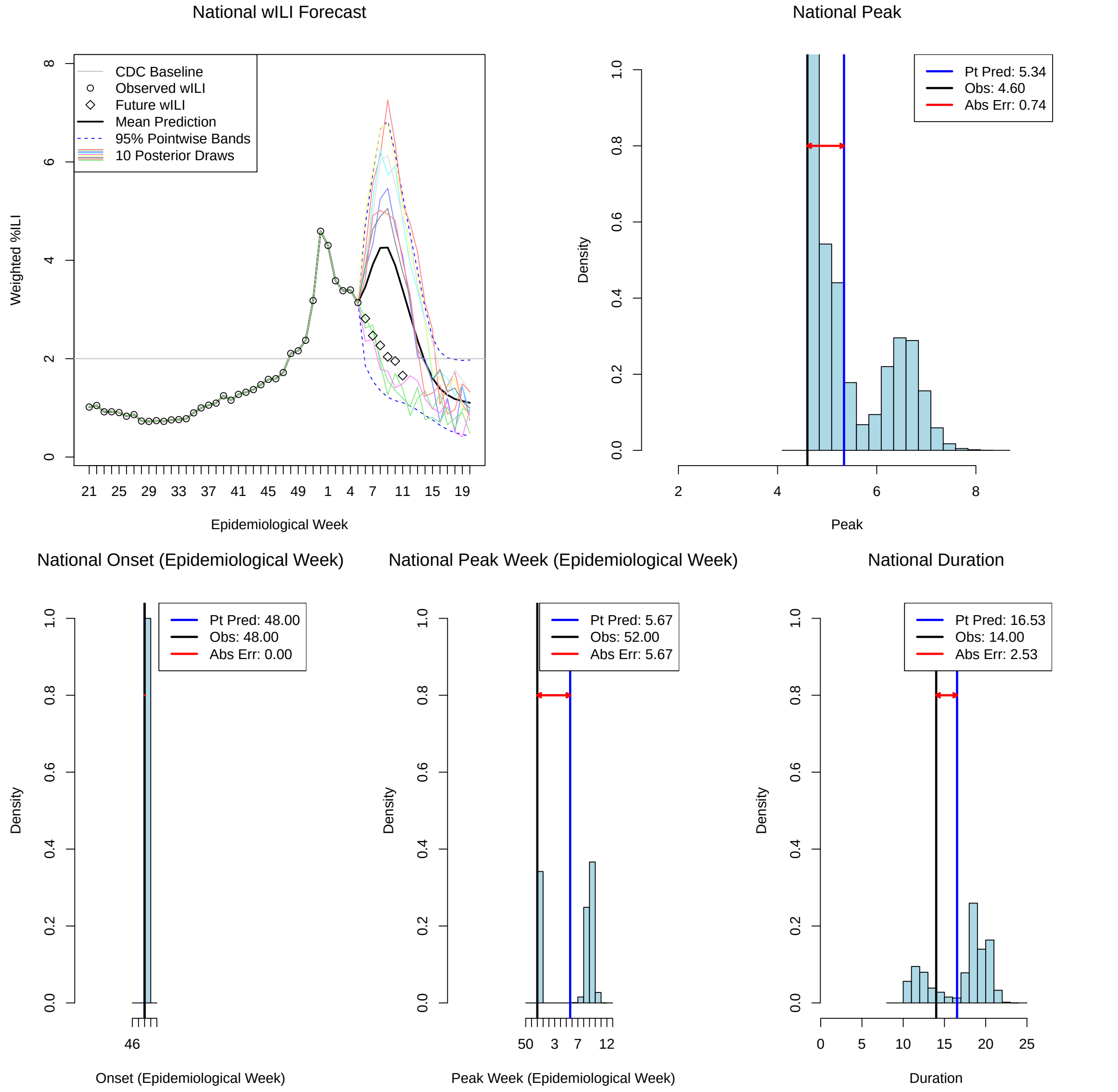

2013–2014 National Forecast, Week 7, using the current framework and the final wILI values. The wILI observations for the week of the forecast and preceding week, are not used in the forecast. "Pt Pred" (point prediction), the posterior mean target value; "Obs", the observed value, "Abs Err", the absolute error of the point prediction.

Weekly forecasts for the 2013–2014 season

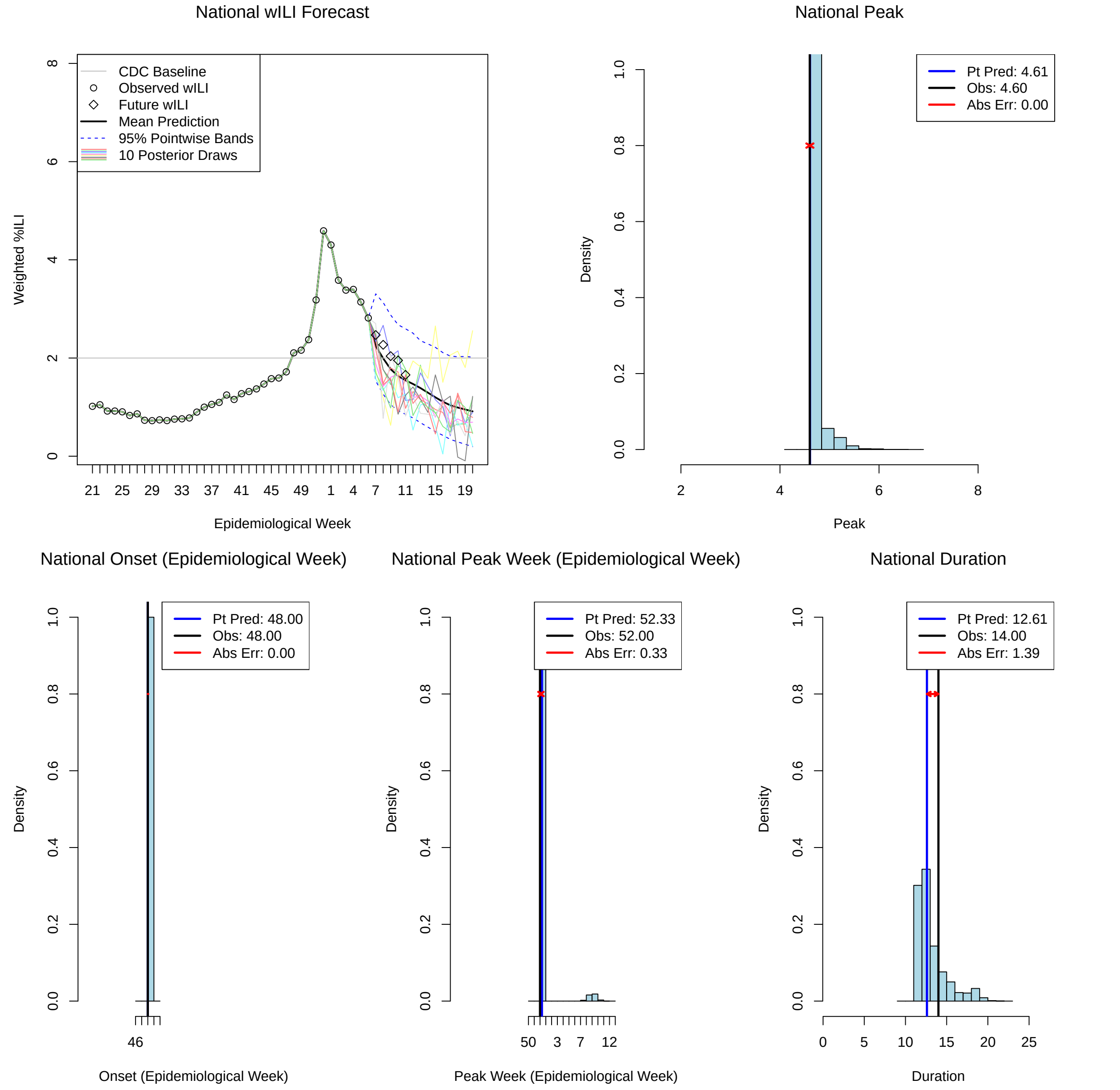

2013–2014 National Forecast, Week 8, using the current framework and the final wILI values. The wILI observations for the week of the forecast and preceding week, are not used in the forecast. “Pt Pred” (point prediction), the posterior mean target value; “Obs”, the observed value, “Abs Err”, the absolute error of the point prediction.

Weekly forecasts for the 2013–2014 season

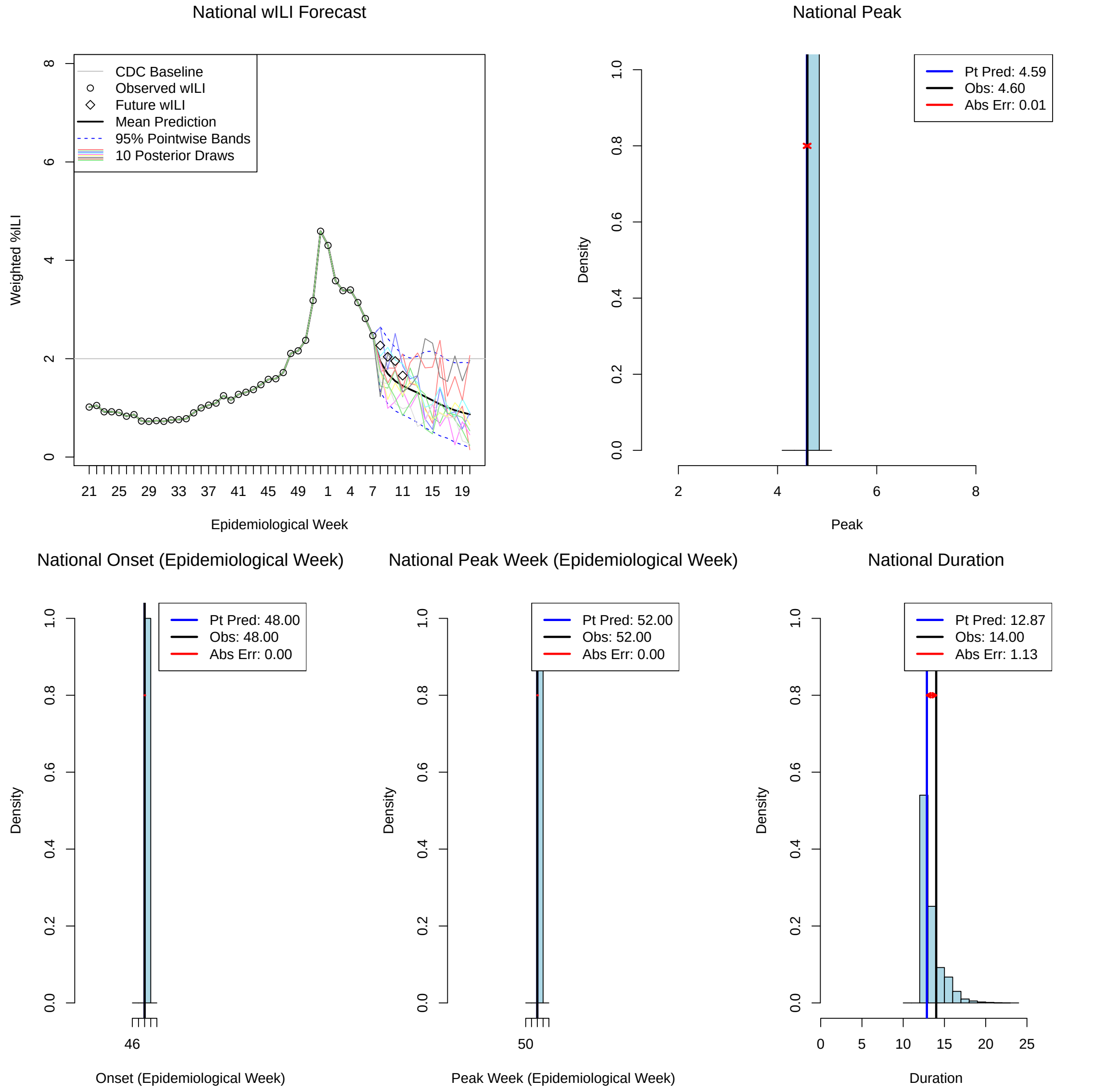

2013–2014 National Forecast, Week 9, using the current framework and the final wILI values. The wILI observations for the week of the forecast and preceding week, are not used in the forecast. "Pt Pred" (point prediction), the posterior mean target value; "Obs", the observed value, "Abs Err", the absolute error of the point prediction.

Weekly forecasts for the 2013–2014 season

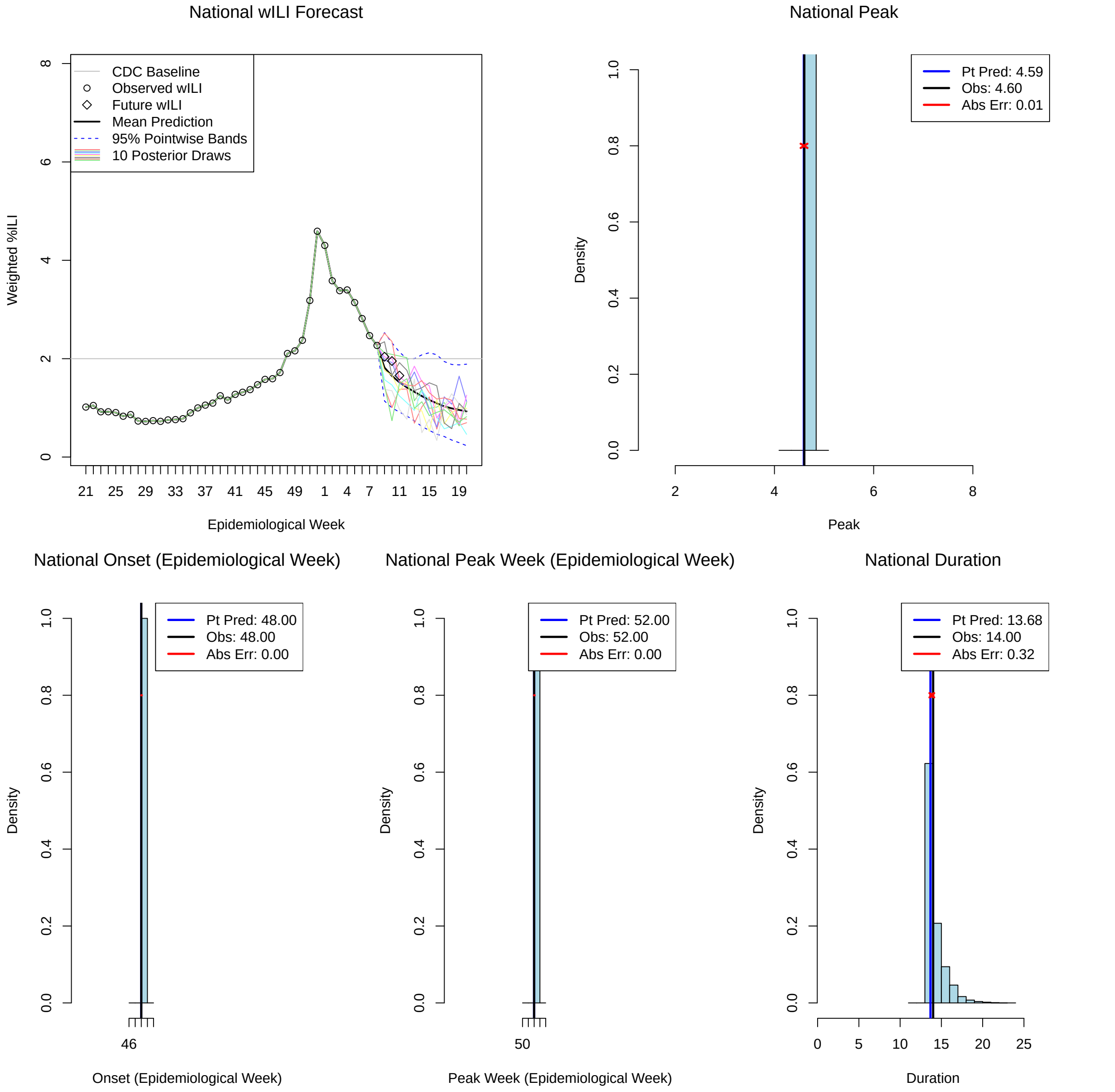

2013–2014 National Forecast, Week 10, using the current framework and the final wILI values. The wILI observations for the week of the forecast and preceding week, are not used in the forecast. “Pt Pred” (point prediction), the posterior mean target value; “Obs”, the observed value, “Abs Err”, the absolute error of the point prediction.

Weekly forecasts for the 2013–2014 season

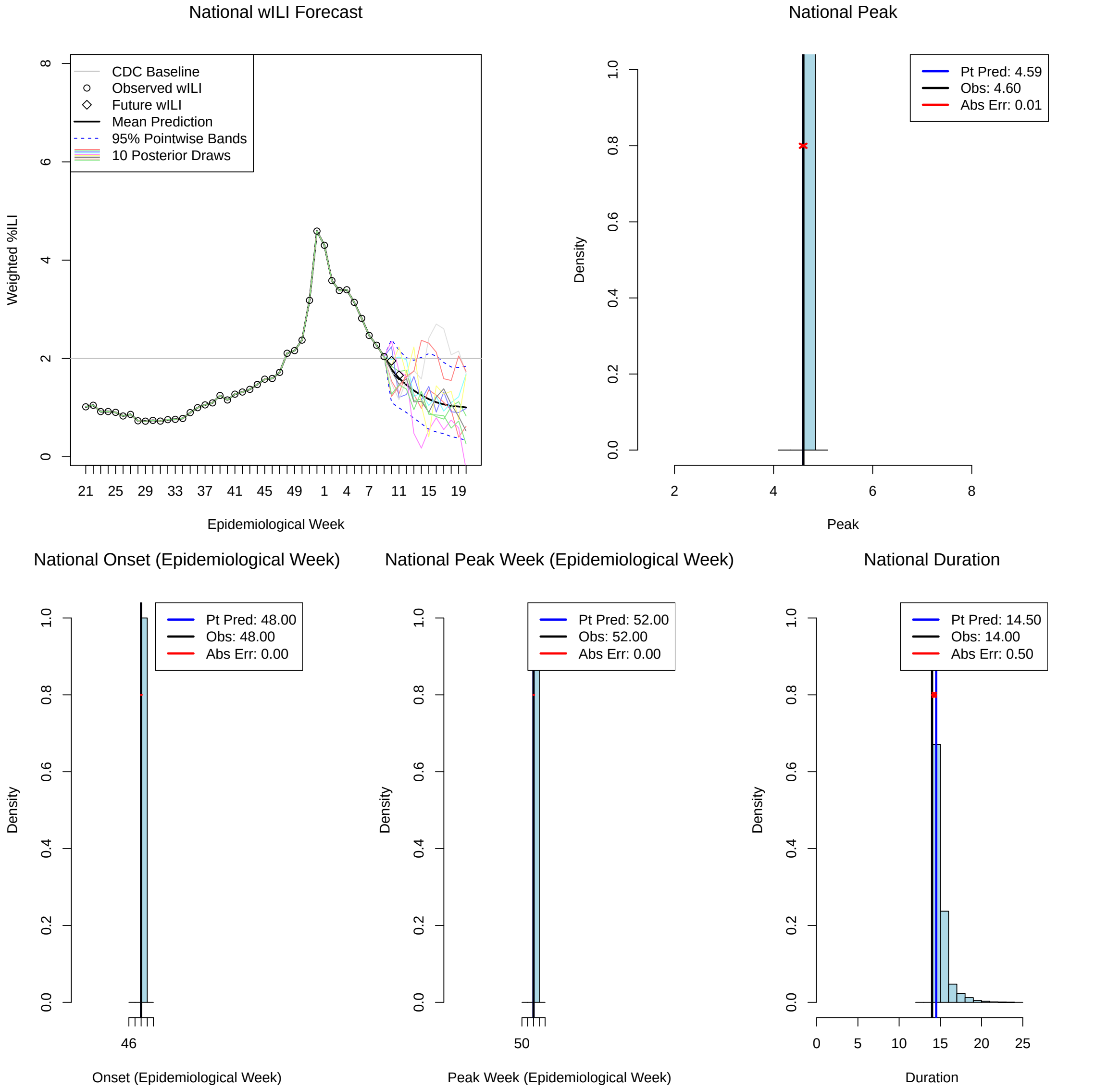

2013–2014 National Forecast, Week 11, using the current framework and the final wILI values. The wILI observations for the week of the forecast and preceding week, are not used in the forecast. "Pt Pred" (point prediction), the posterior mean target value; "Obs", the observed value, "Abs Err", the absolute error of the point prediction.

Weekly forecasts for the 2013–2014 season

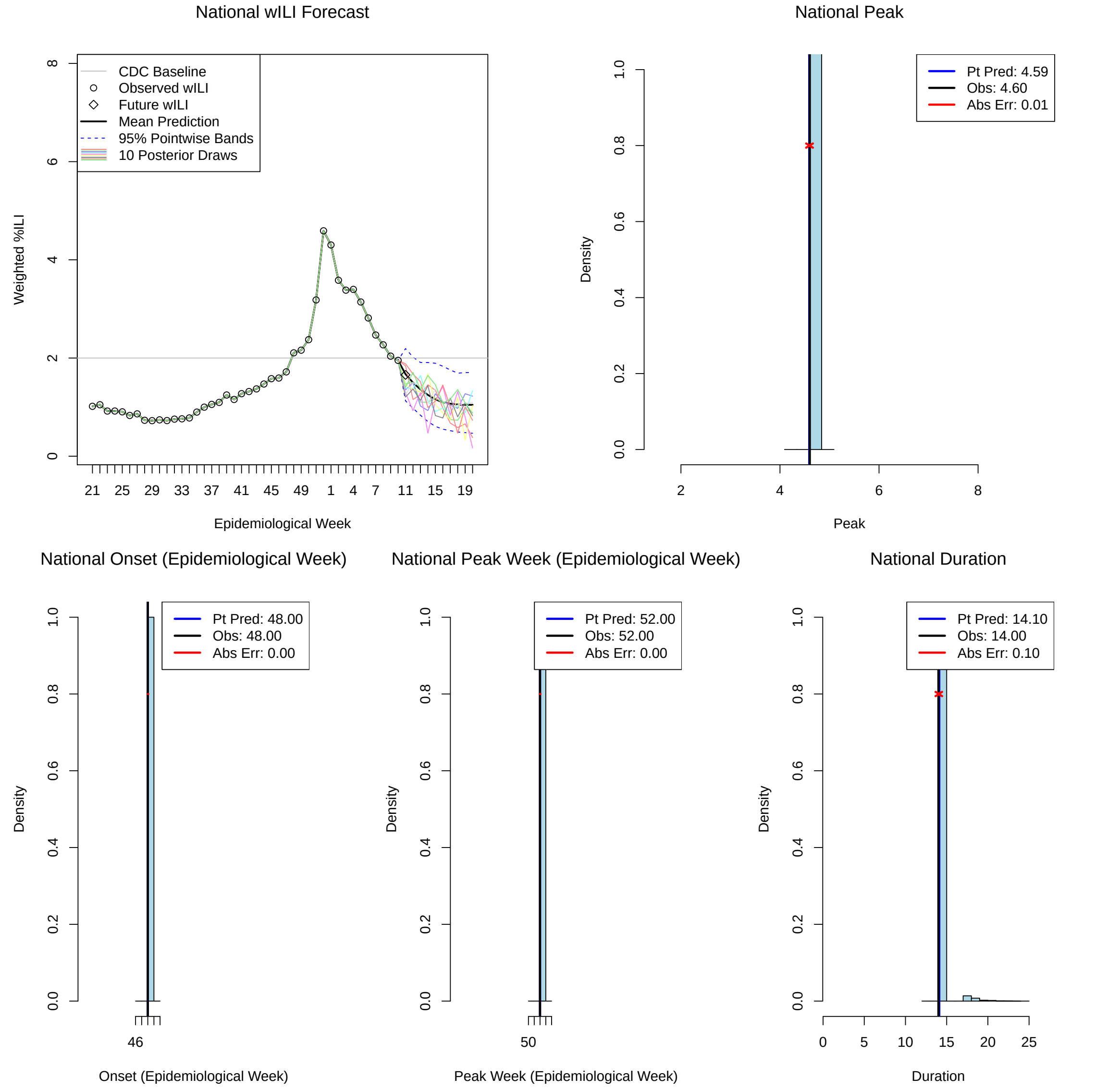

2013–2014 National Forecast, Week 12, using the current framework and the final wILI values. The wILI observations for the week of the forecast and preceding week, are not used in the forecast. “Pt Pred” (point prediction), the posterior mean target value; “Obs”, the observed value, “Abs Err”, the absolute error of the point prediction.

Weekly forecasts for the 2013–2014 season

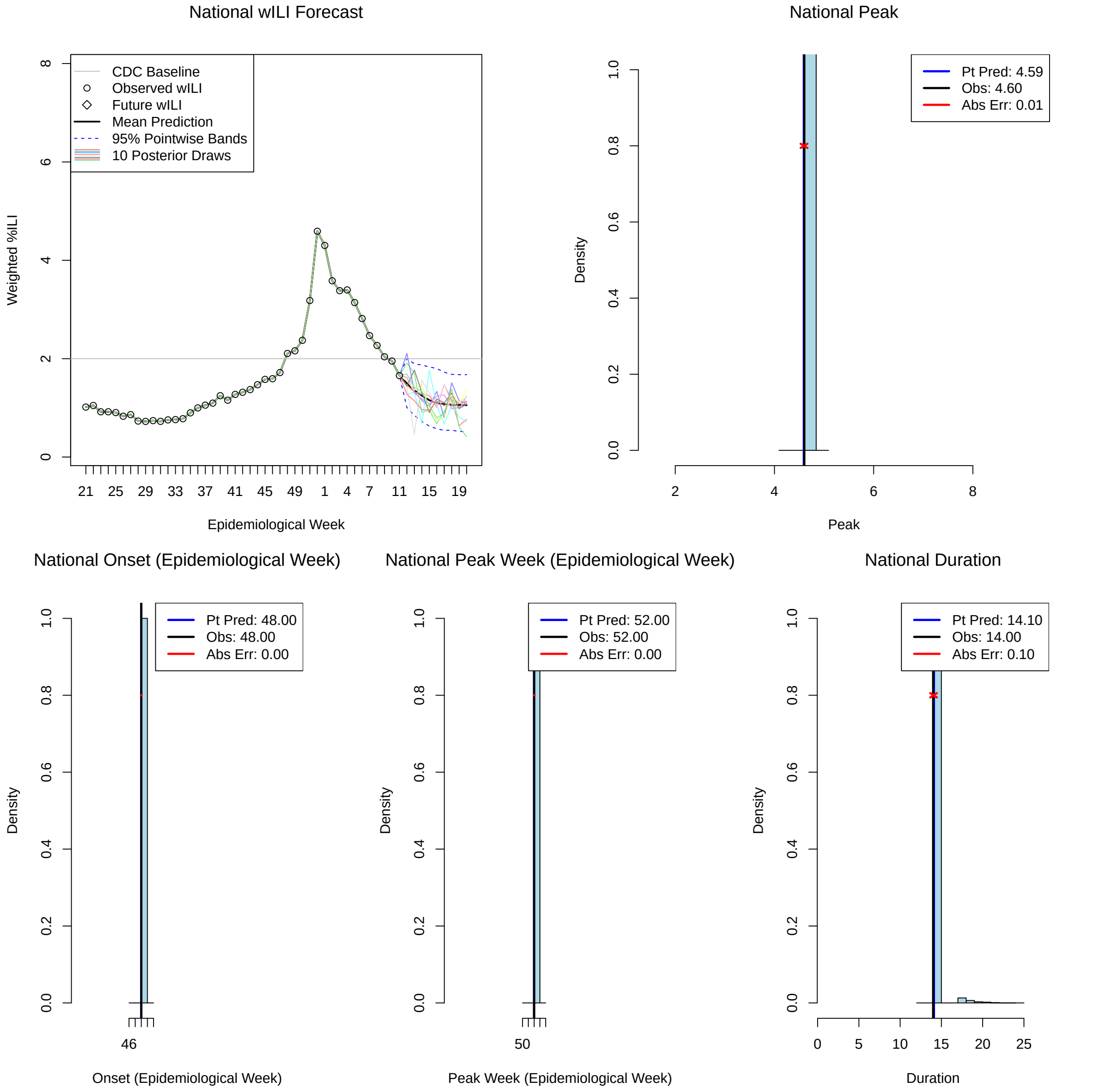

2013–2014 National Forecast, Week 13, using the current framework and the final wILI values. The wILI observations for the week of the forecast and preceding week, are not used in the forecast. "Pt Pred" (point prediction), the posterior mean target value; "Obs", the observed value, "Abs Err", the absolute error of the point prediction.

Weekly forecasts for the 2013–2014 season

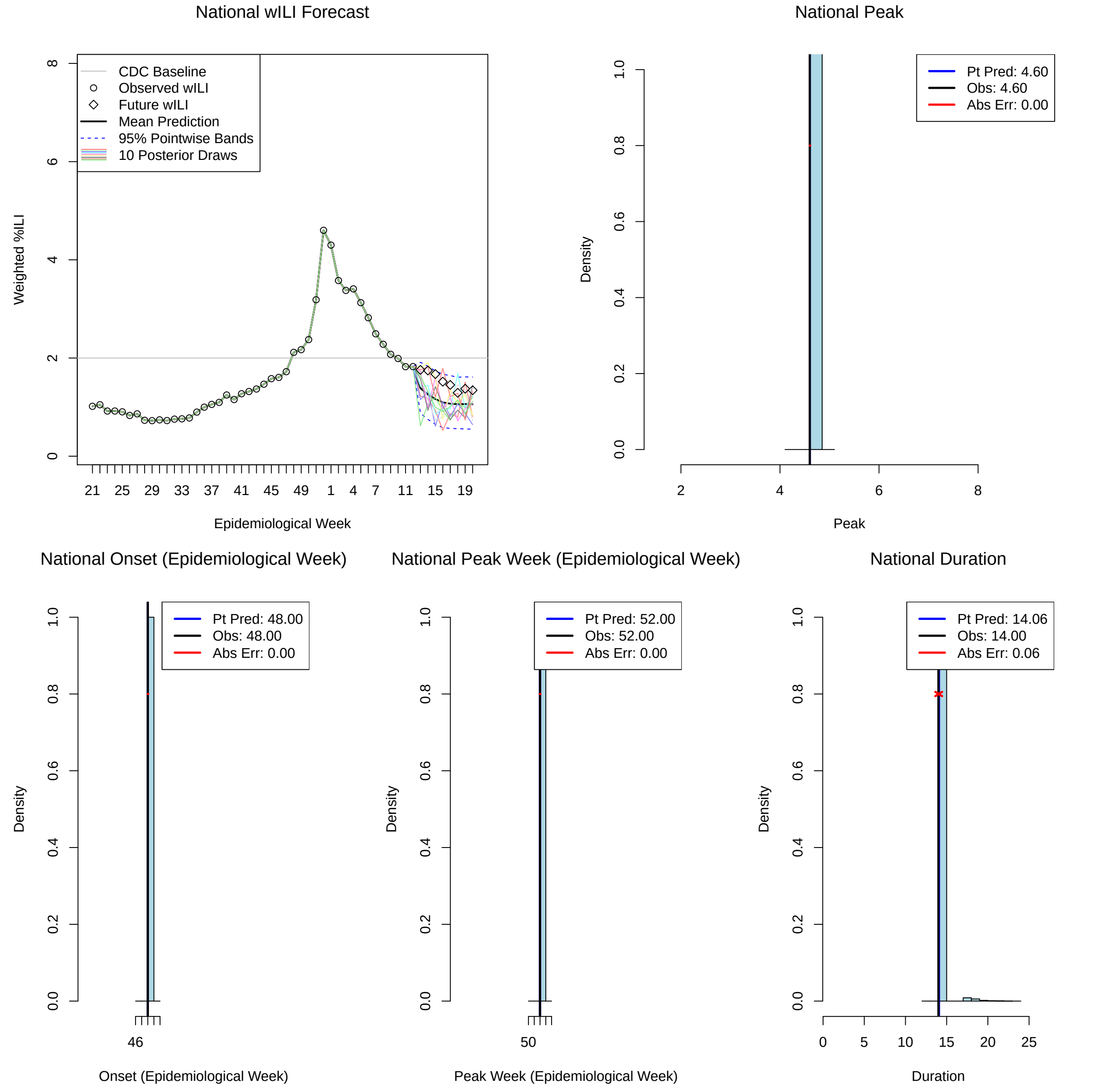

2013–2014 National Forecast, Week 14, using the current framework and the final wILI values. The wILI observations for the week of the forecast and preceding week, are not used in the forecast. “Pt Pred” (point prediction), the posterior mean target value; “Obs”, the observed value, “Abs Err”, the absolute error of the point prediction.

Weekly forecasts for the 2013–2014 season

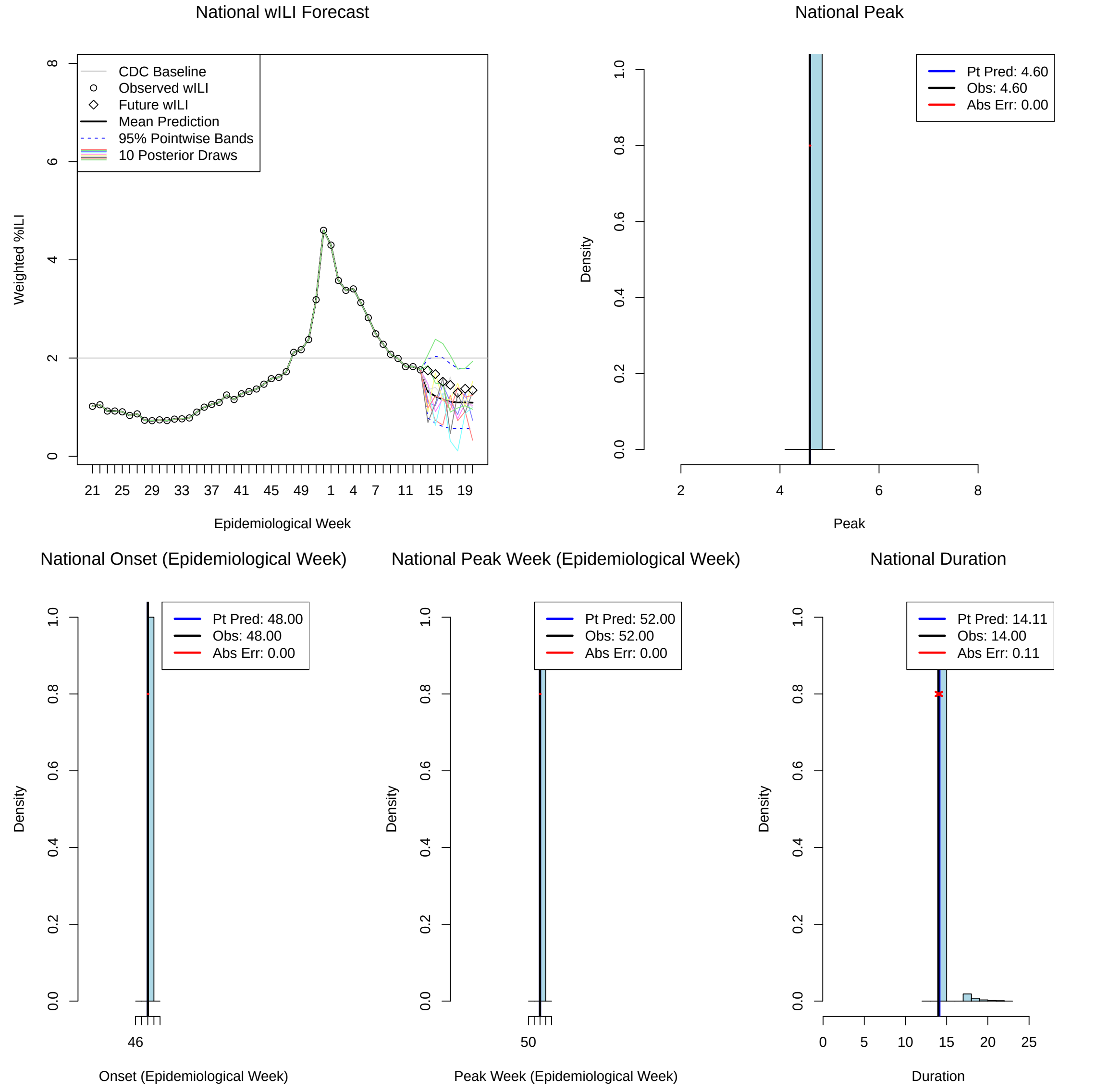

2013–2014 National Forecast, Week 15, using the current framework and the final wILI values. The wILI observations for the week of the forecast and preceding week, are not used in the forecast. "Pt Pred" (point prediction), the posterior mean target value; "Obs", the observed value, "Abs Err", the absolute error of the point prediction.

Weekly forecasts for the 2013–2014 season

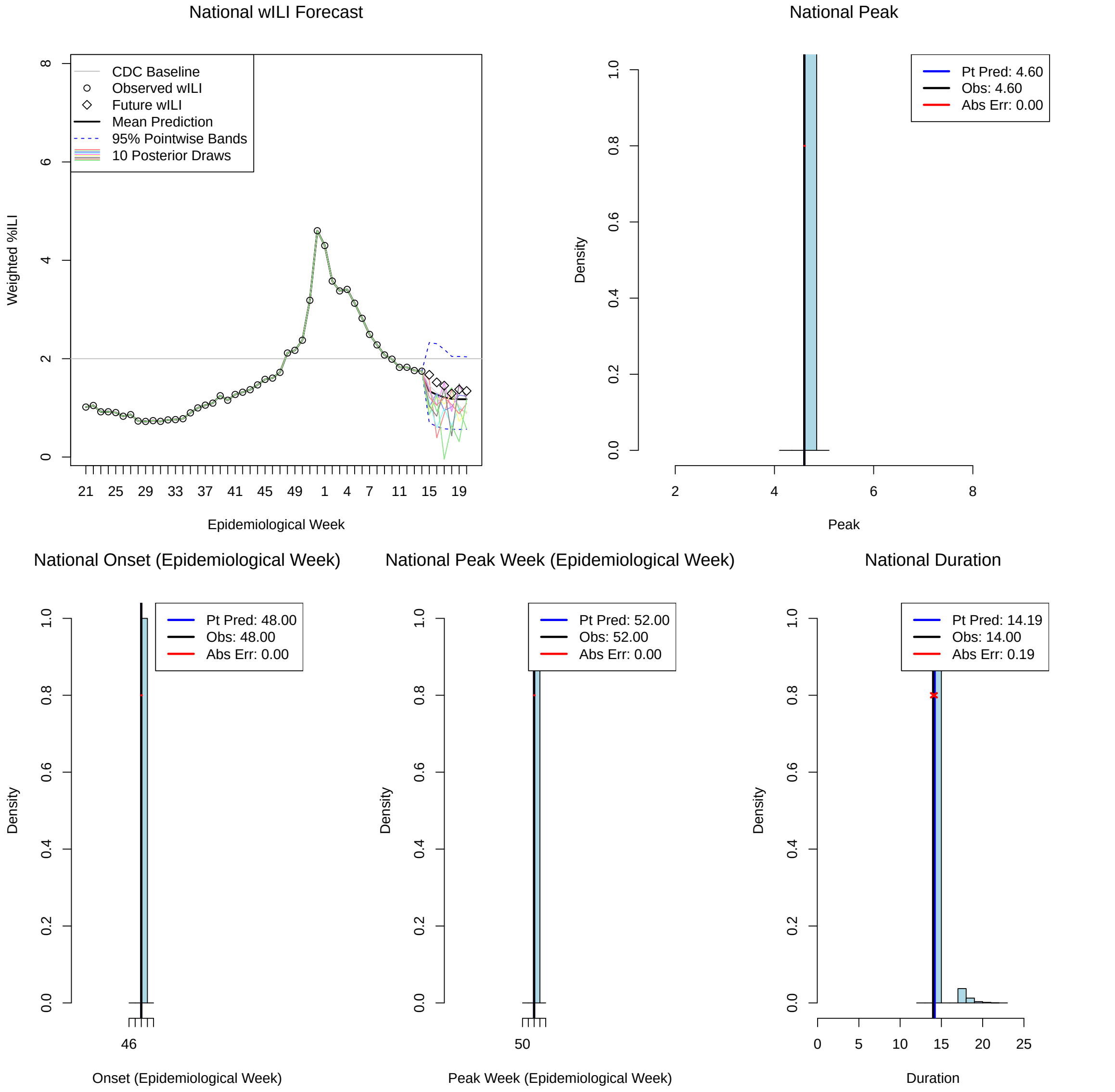

2013–2014 National Forecast, Week 16, using the current framework and the final wILI values. The wILI observations for the week of the forecast and preceding week, are not used in the forecast. "Pt Pred" (point prediction), the posterior mean target value; "Obs", the observed value, "Abs Err", the absolute error of the point prediction.

Weekly forecasts for the 2013–2014 season

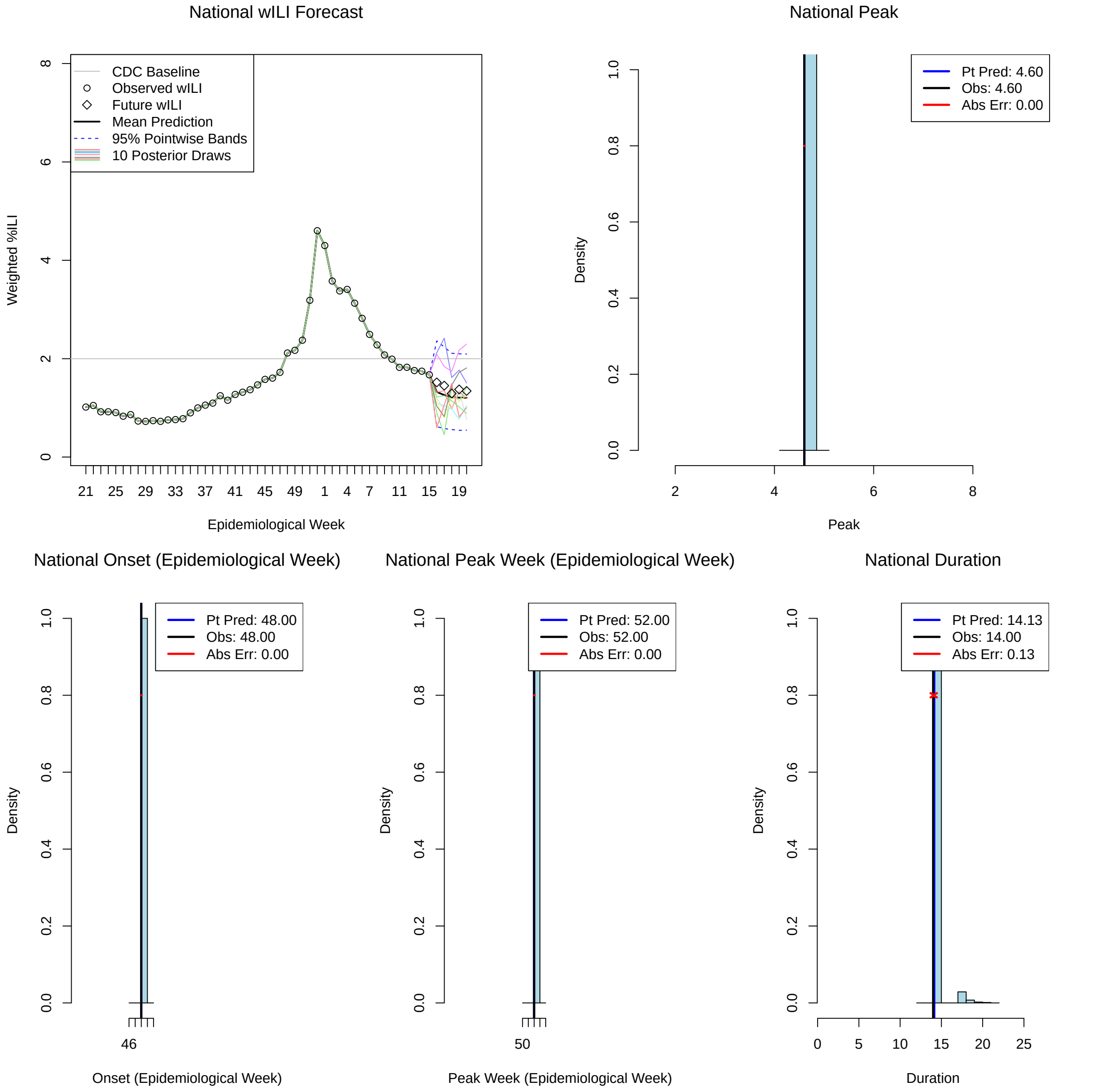

2013–2014 National Forecast, Week 17, using the current framework and the final wILI values. The wILI observations for the week of the forecast and preceding week, are not used in the forecast. "Pt Pred" (point prediction), the posterior mean target value; "Obs", the observed value, "Abs Err", the absolute error of the point prediction.

Weekly forecasts for the 2013–2014 season

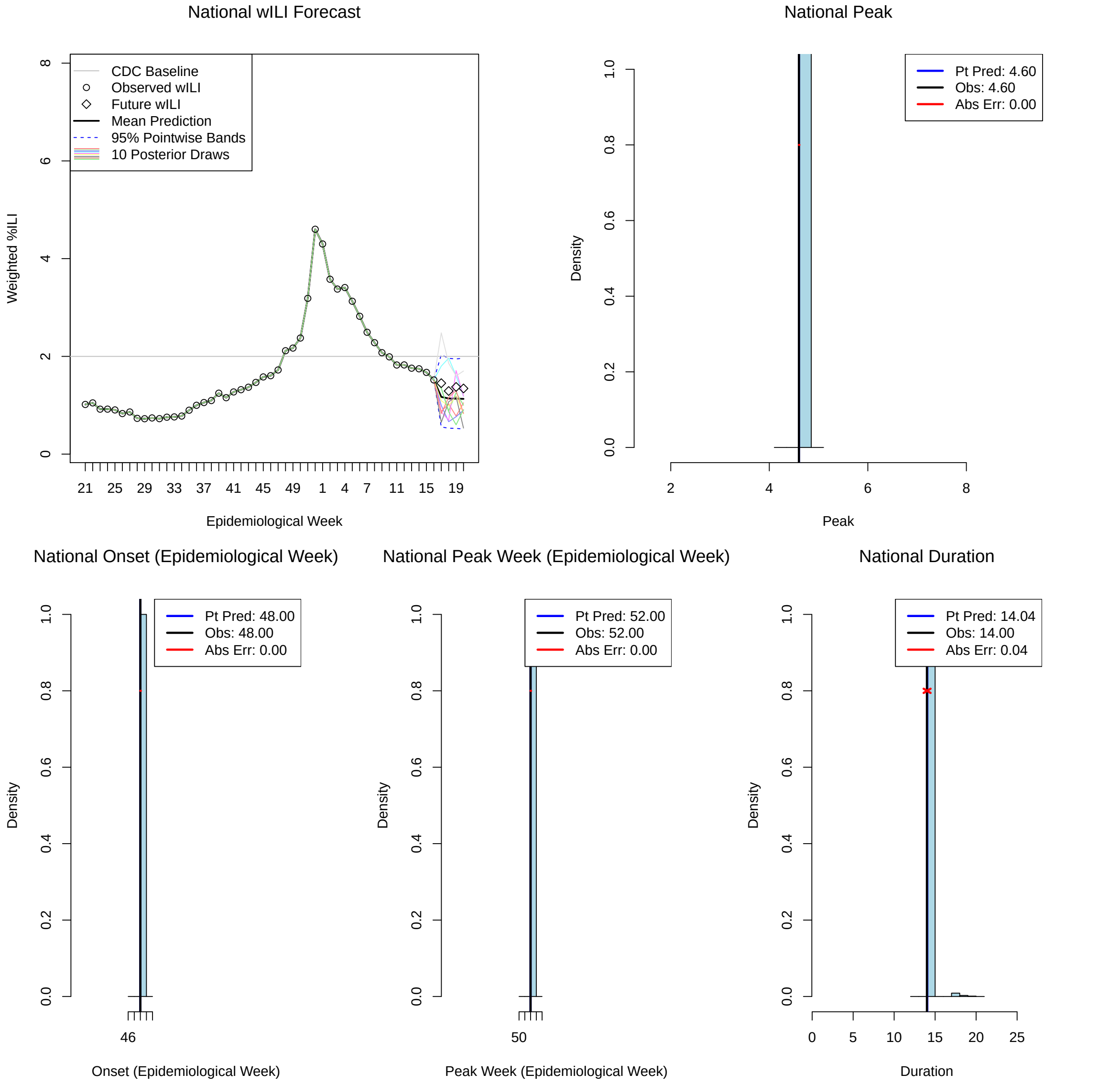

2013–2014 National Forecast, Week 18, using the current framework and the final wILI values. The wILI observations for the week of the forecast and preceding week, are not used in the forecast. "Pt Pred" (point prediction), the posterior mean target value; "Obs", the observed value, "Abs Err", the absolute error of the point prediction.

Weekly forecasts for the 2013–2014 season

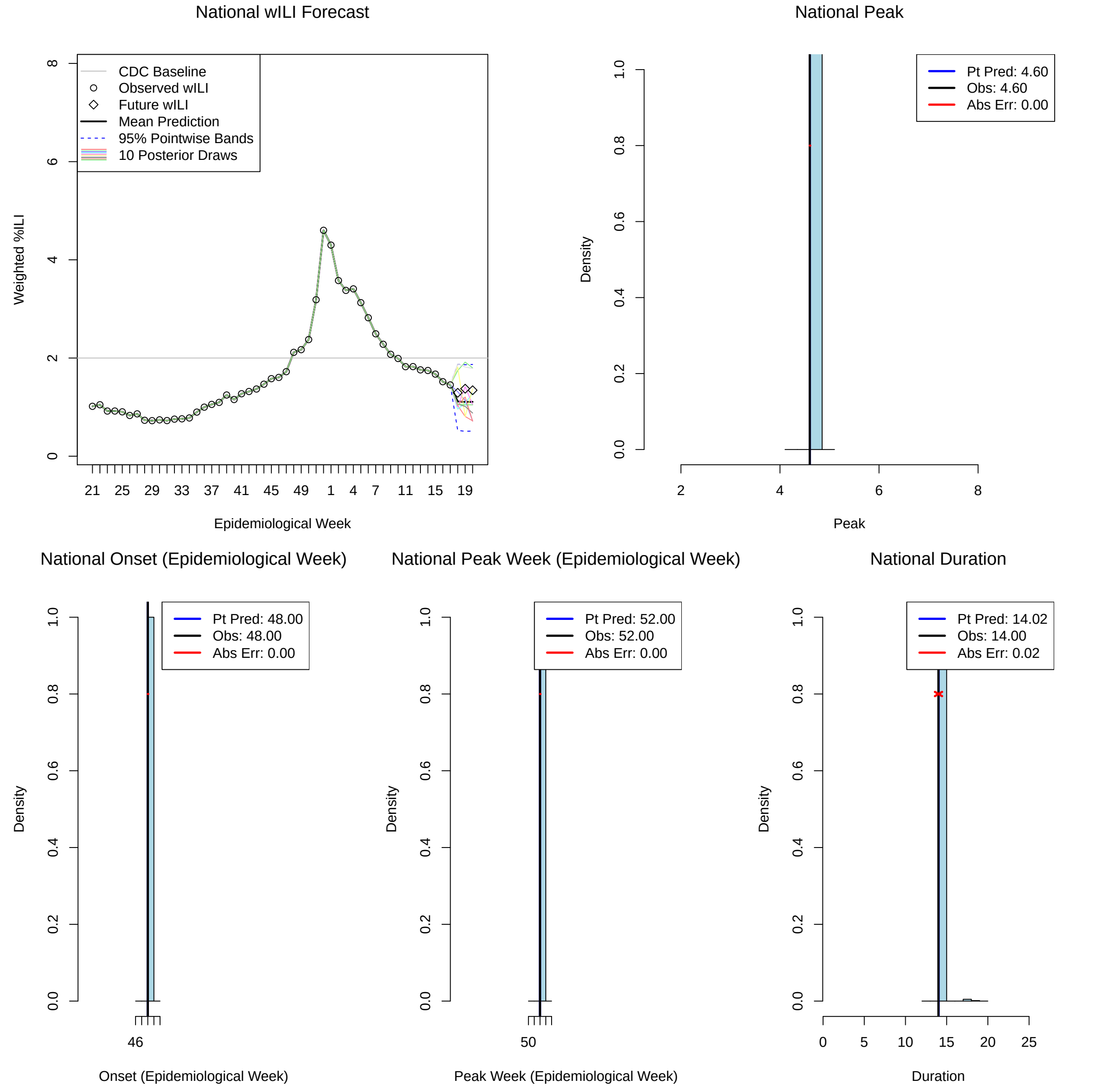

2013–2014 National Forecast, Week 19, using the current framework and the final wILI values. The wILI observations for the week of the forecast and preceding week, are not used in the forecast. "Pt Pred" (point prediction), the posterior mean target value; "Obs", the observed value, "Abs Err", the absolute error of the point prediction.

Weekly forecasts for the 2013–2014 season

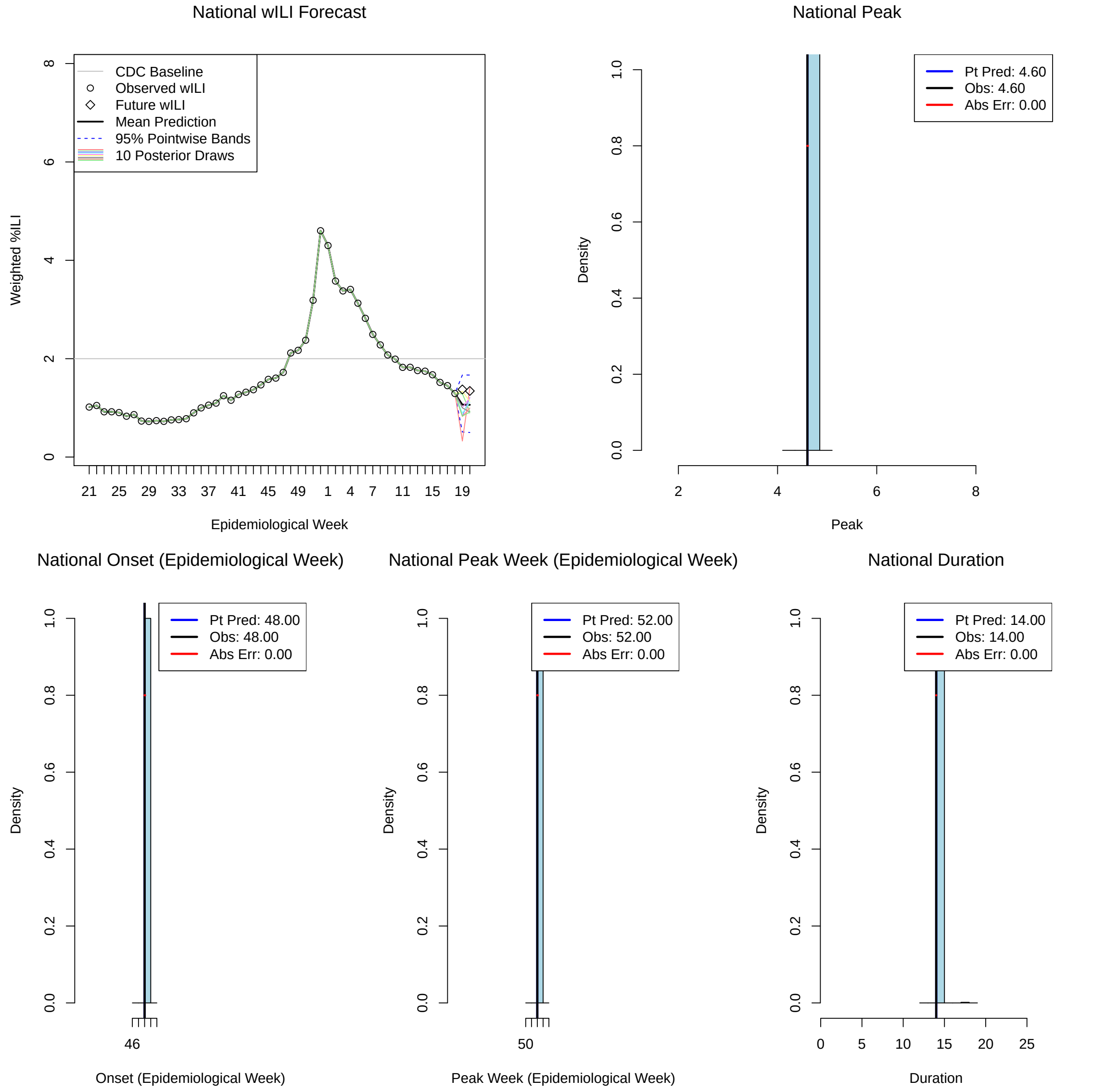

2013–2014 National Forecast, Week 20, using the current framework and the final wILI values. The wILI observations for the week of the forecast and preceding week, are not used in the forecast. "Pt Pred" (point prediction), the posterior mean target value; "Obs", the observed value, "Abs Err", the absolute error of the point prediction.

Weekly forecasts for the 2013–2014 season

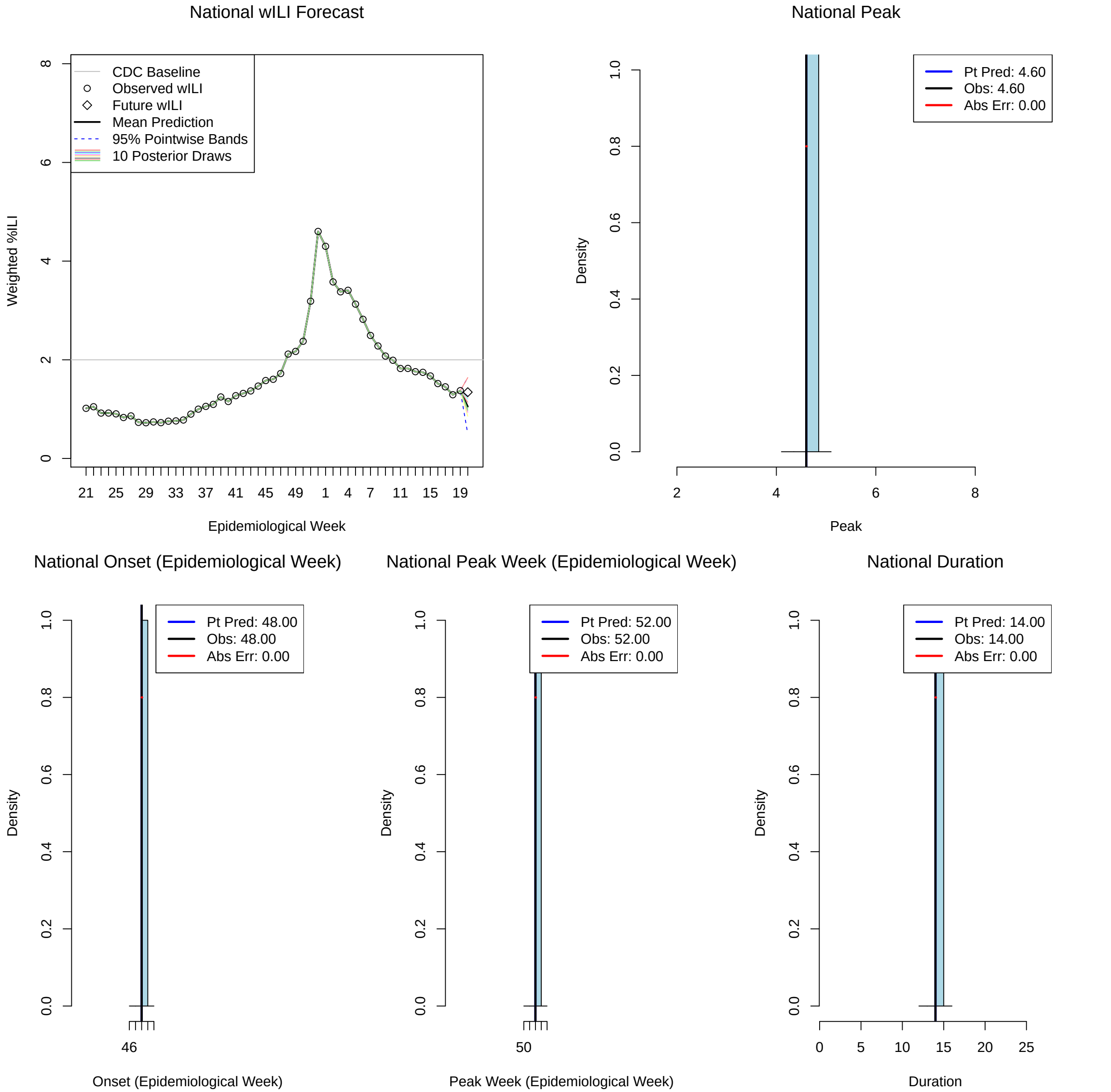

2013–2014 National Forecast, Week 21, using the current framework and the final wILI values. The wILI observations for the week of the forecast and preceding week, are not used in the forecast. "Pt Pred" (point prediction), the posterior mean target value; "Obs", the observed value, "Abs Err", the absolute error of the point prediction.

Weekly forecasts for the 2013–2014 season

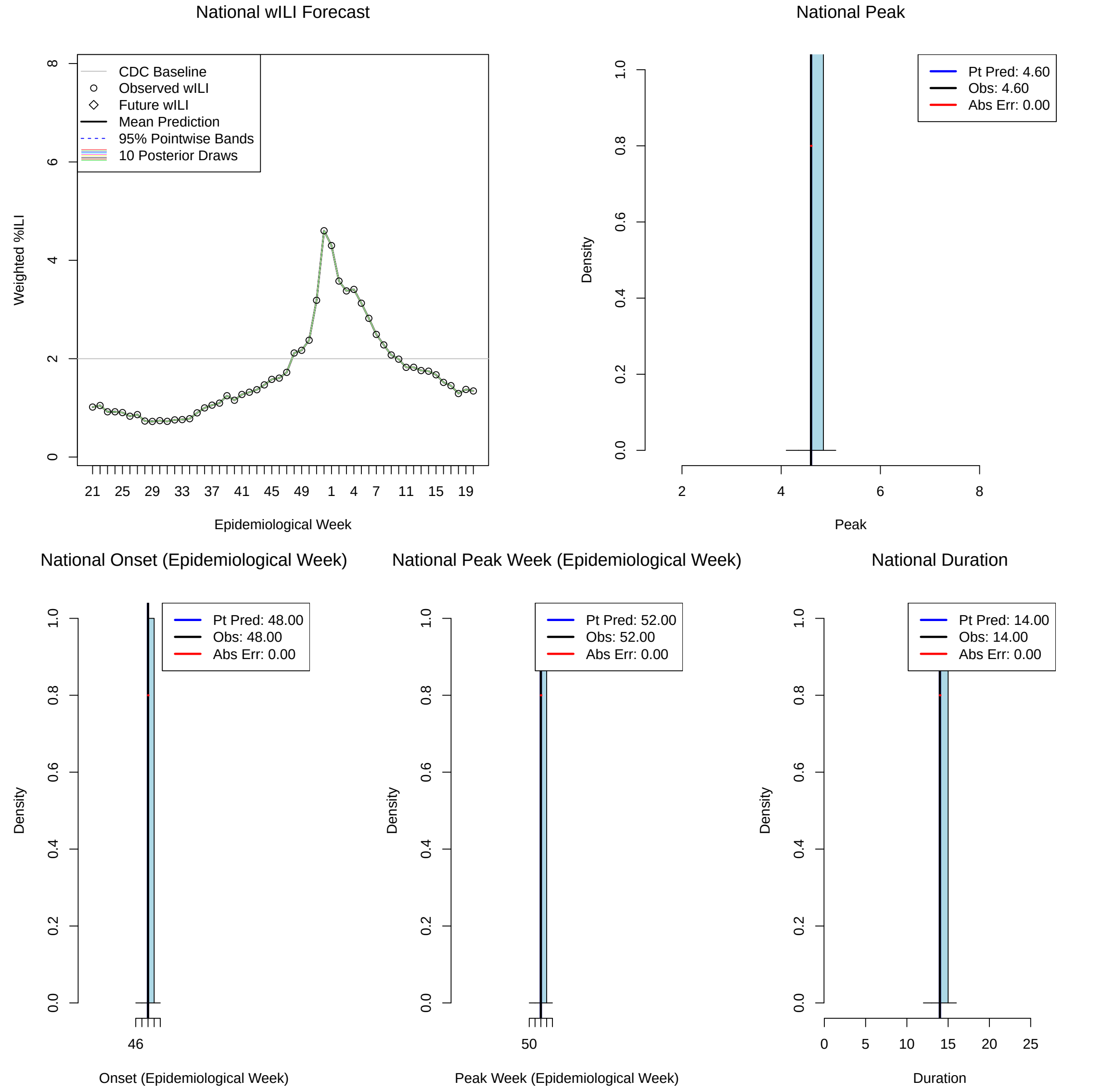

2013–2014 National Forecast, Week 22, using the current framework and the final wILI values. The wILI observations for the week of the forecast and preceding week, are not used in the forecast. "Pt Pred" (point prediction), the posterior mean target value; "Obs", the observed value, "Abs Err", the absolute error of the point prediction.
