# Supplementary material for: Flexible Modeling of Epidemics with an Empirical Bayes Framework
Source: PLoS Comput Biol. 2015 Aug 28;11(8):e1004382. doi: 10.1371/journal.pcbi.1004382 (PMC4552841; doi:10.1371/journal.pcbi.1004382)
Supplement: S2 Fig — The forecast without transformations is composed almost entirely of trajectories that are nearly identical to one of three past seasons. The forecast with transformations contains a much wider variety of curves. (PDF) [file pcbi.1004382.s002.pdf]

Week 49 predictions with and without using transformations in prior

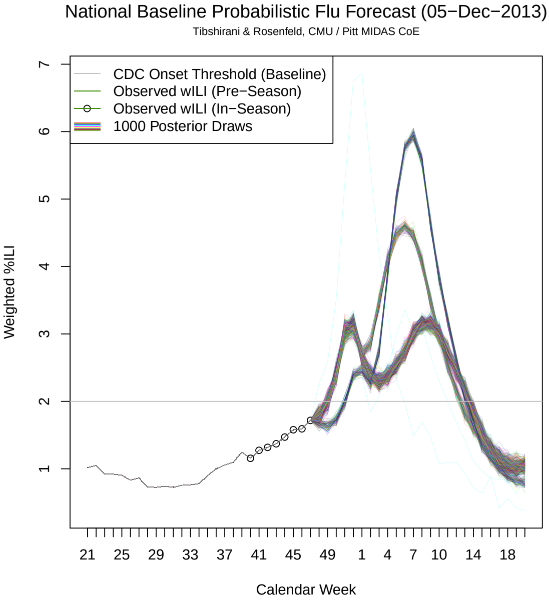

Prior without transformations

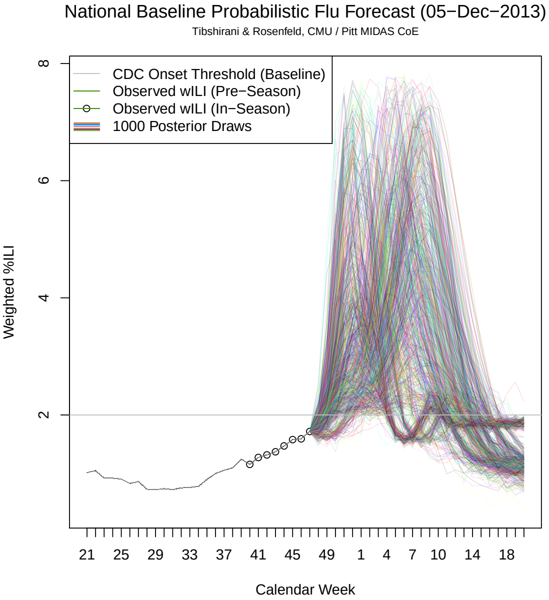

Prior with transformations
