# Supplementary material for: Flexible Modeling of Epidemics with an Empirical Bayes Framework
Source: PLoS Comput Biol. 2015 Aug 28;11(8):e1004382. doi: 10.1371/journal.pcbi.1004382 (PMC4552841; doi:10.1371/journal.pcbi.1004382)

Cross-validated error plots for national and regional point predictions

(A) The average point prediction error against epidemic weeks aligned by the observed onset and peak week.

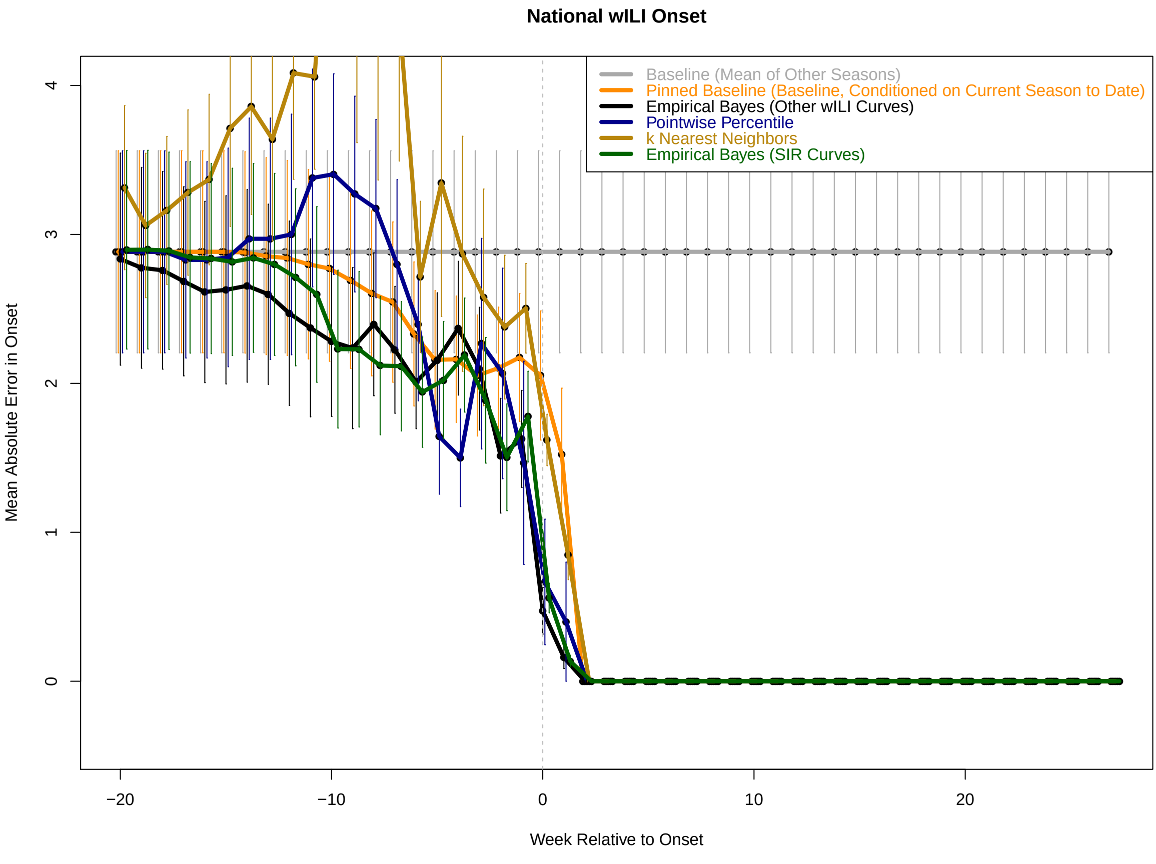

Cross-validated error plots for national and regional point predictions

(A) The average point prediction error against epidemic weeks aligned by the observed onset and peak week.

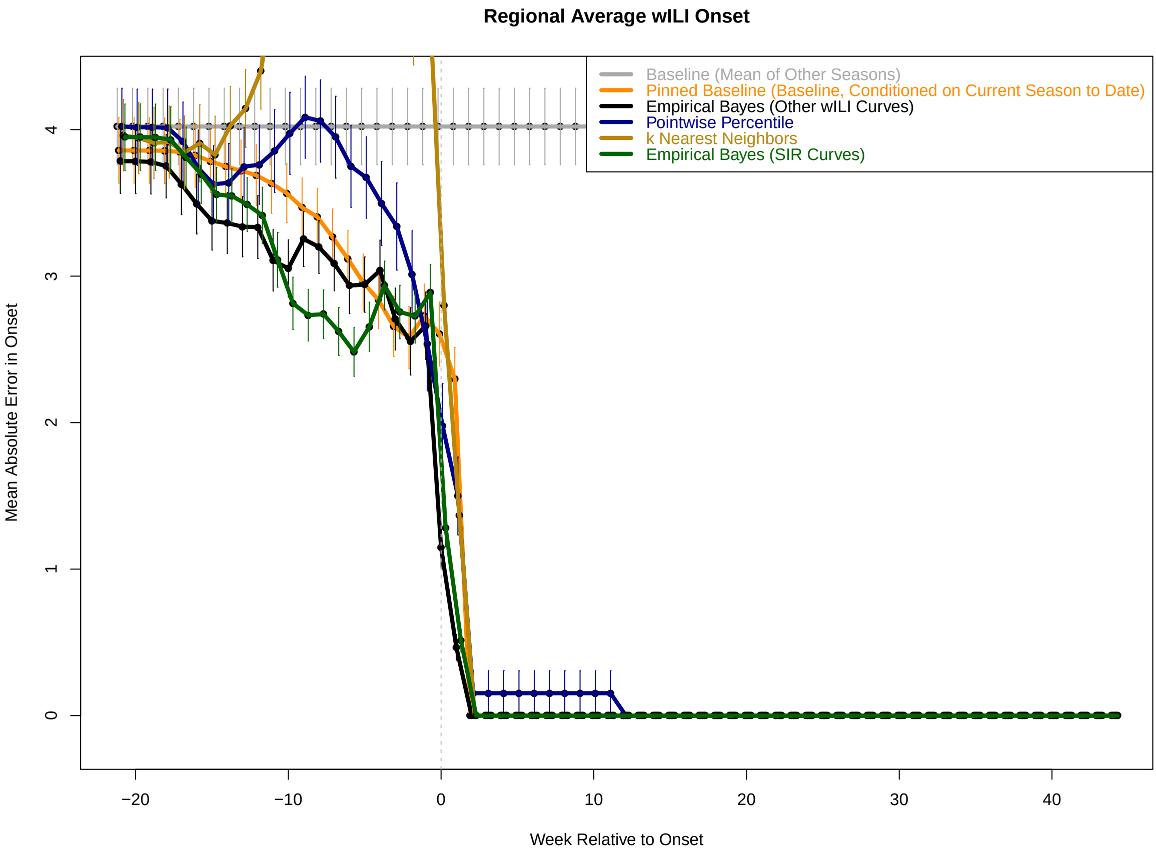

Cross-validated error plots for national and regional point predictions

(A) The average point prediction error against epidemic weeks aligned by the observed onset and peak week.

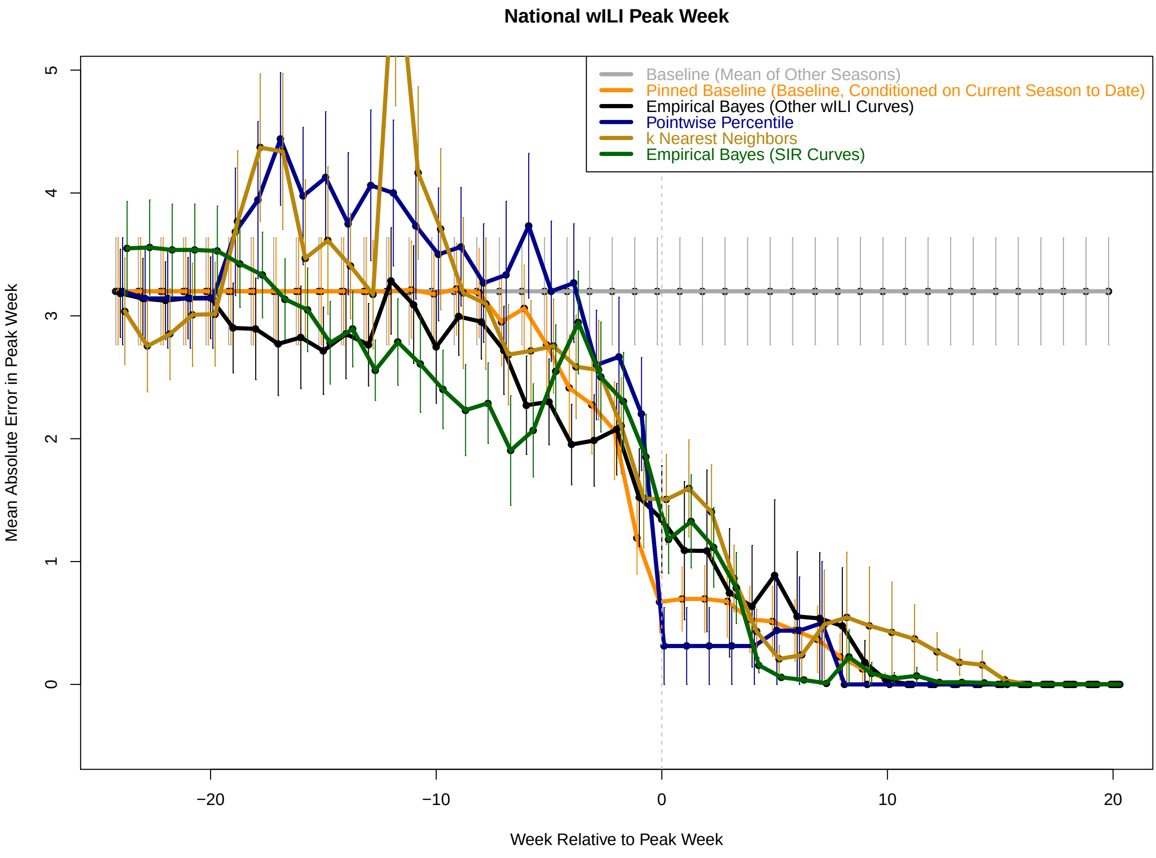

Cross-validated error plots for national and regional point predictions

(A) The average point prediction error against epidemic weeks aligned by the observed onset and peak week.

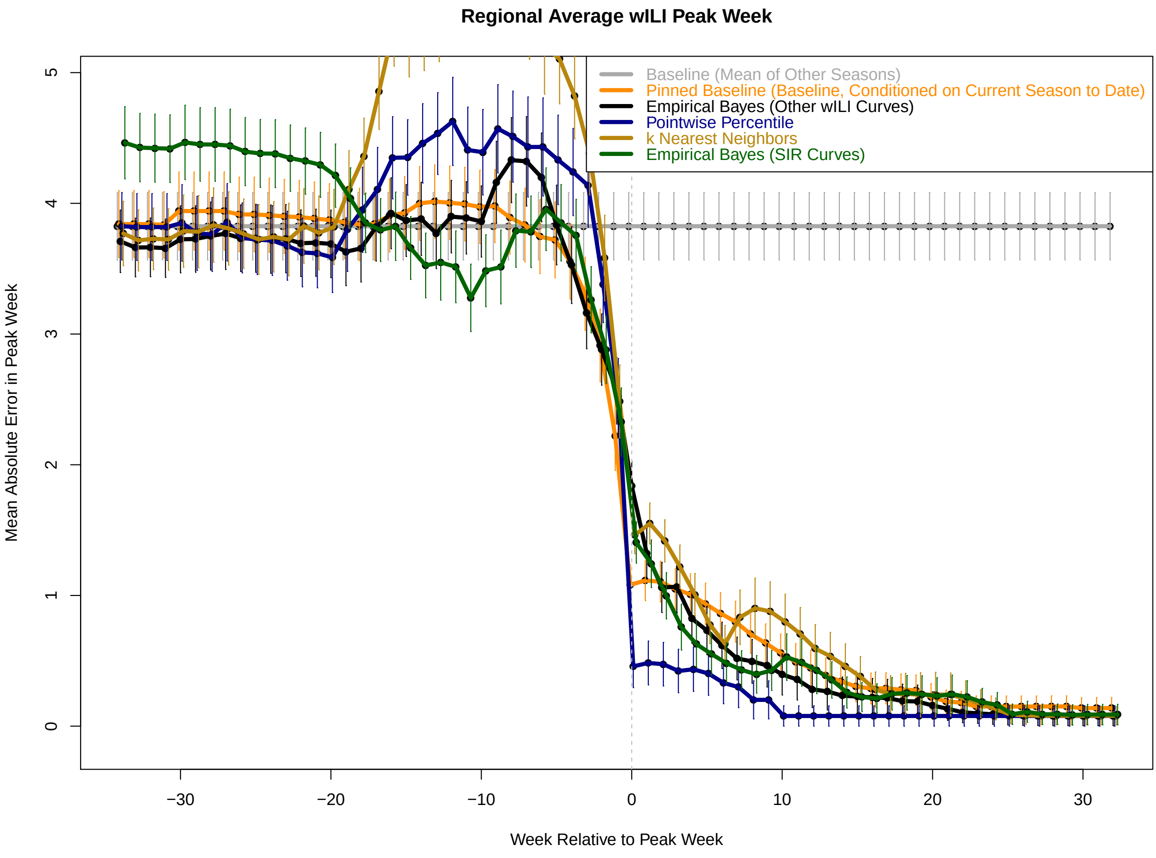

Cross-validated error plots for national and regional point predictions

(A) The average point prediction error against epidemic weeks aligned by the observed onset and peak week.

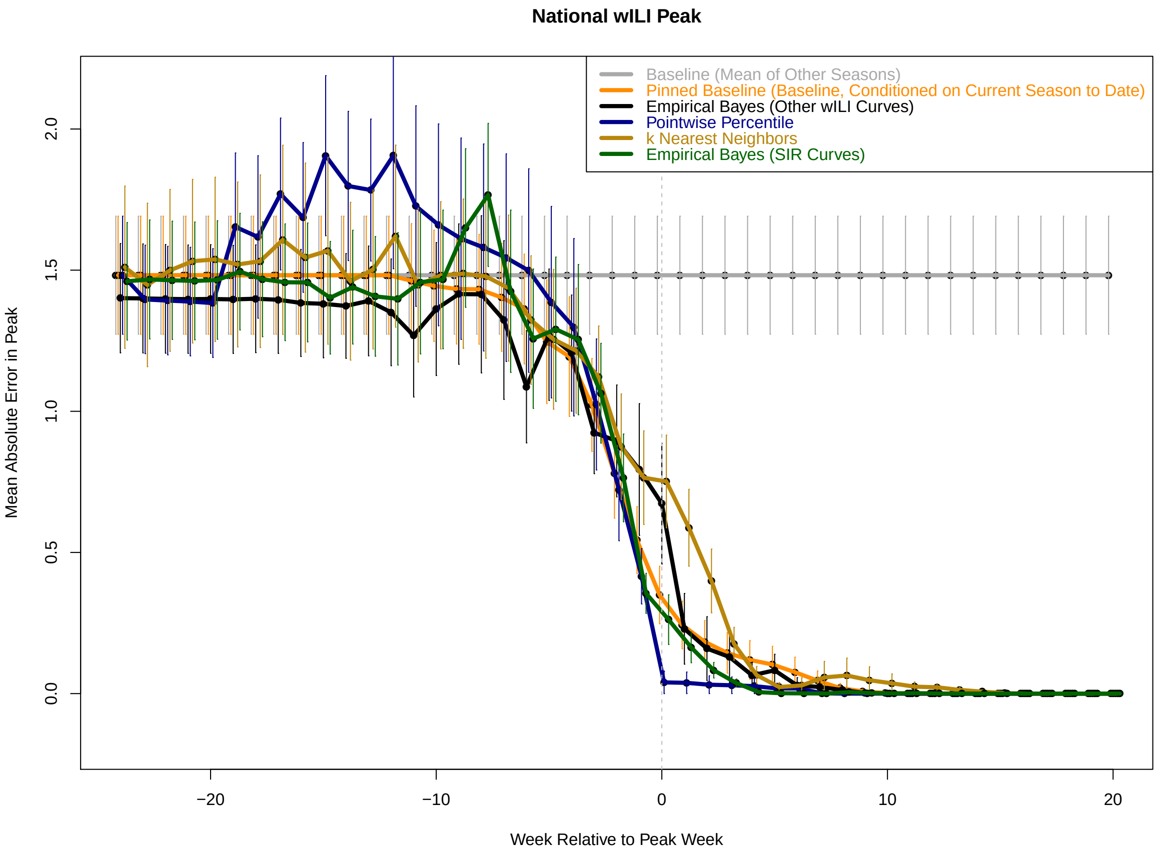

Cross-validated error plots for national and regional point predictions

(A) The average point prediction error against epidemic weeks aligned by the observed onset and peak week.

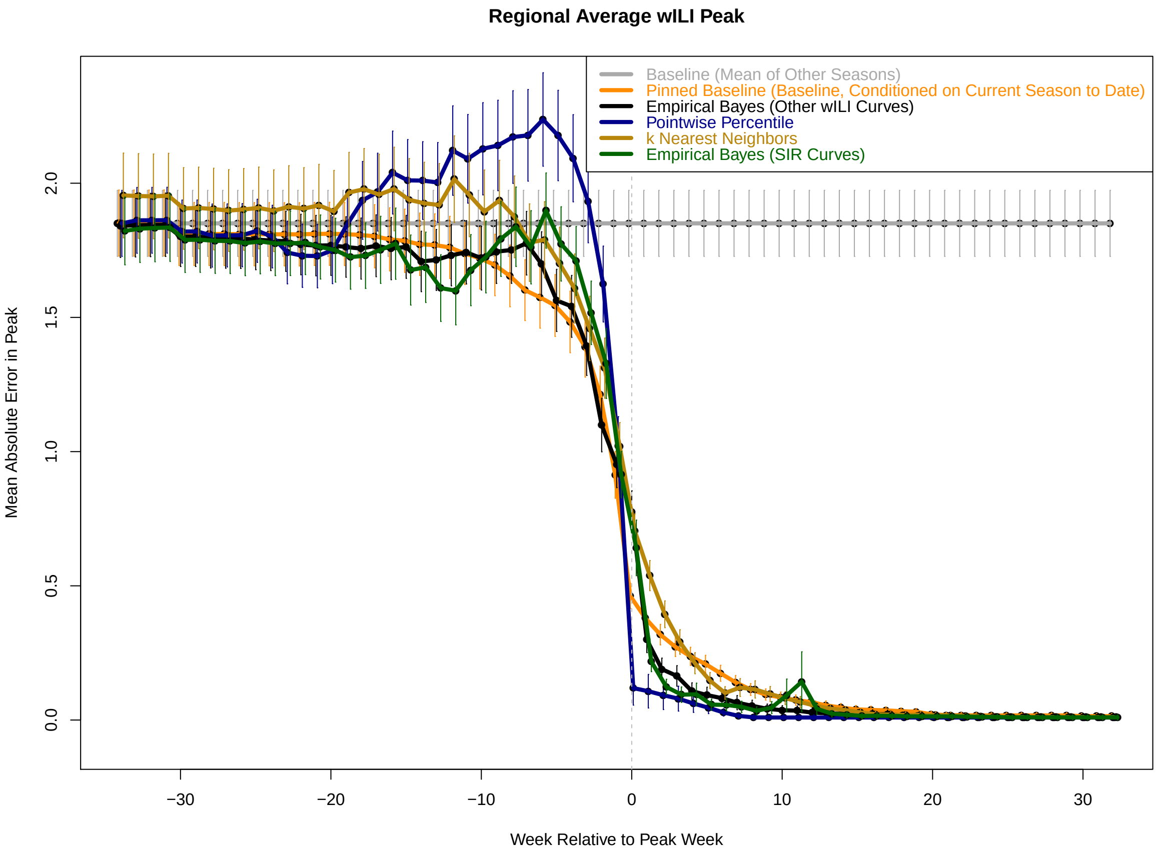

Cross-validated error plots for national and regional point predictions

(A) The average point prediction error against epidemic weeks aligned by the observed onset and peak week.

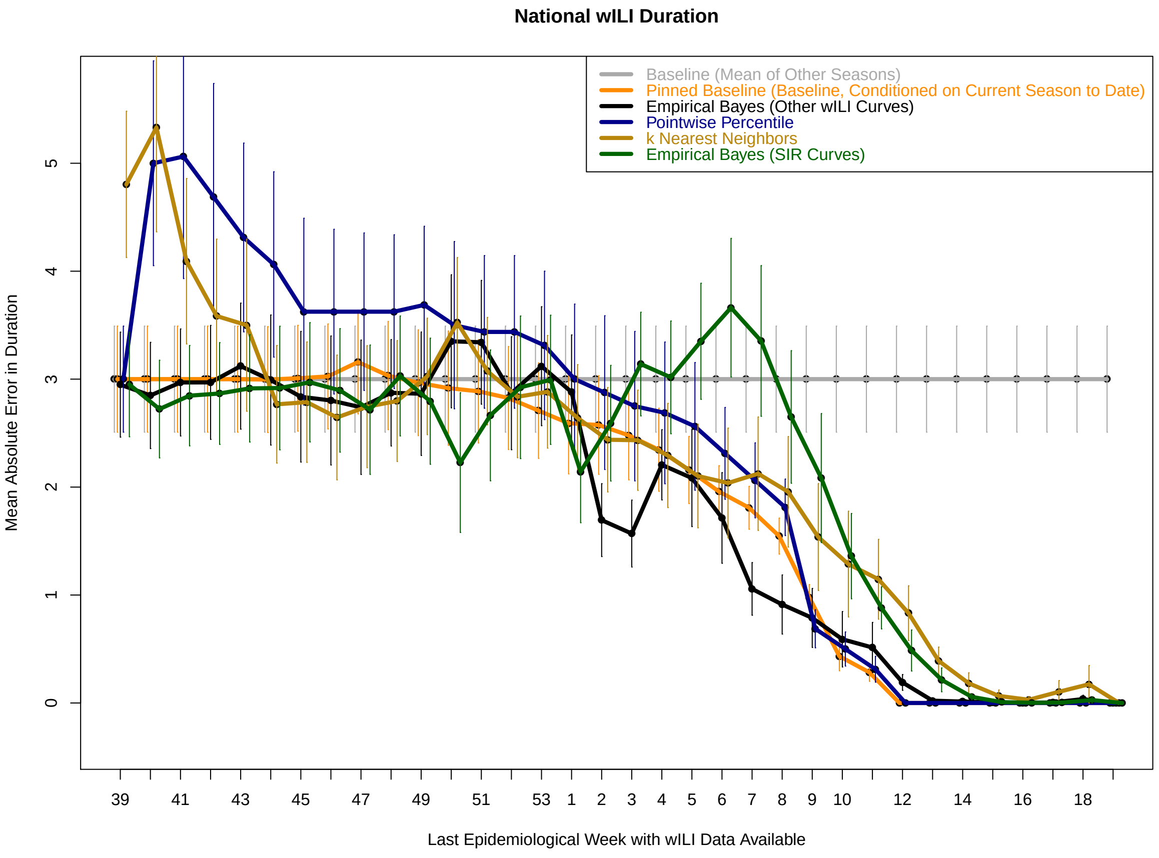

Cross-validated error plots for national and regional point predictions

(A) The average point prediction error against epidemic weeks aligned by the observed onset and peak week.

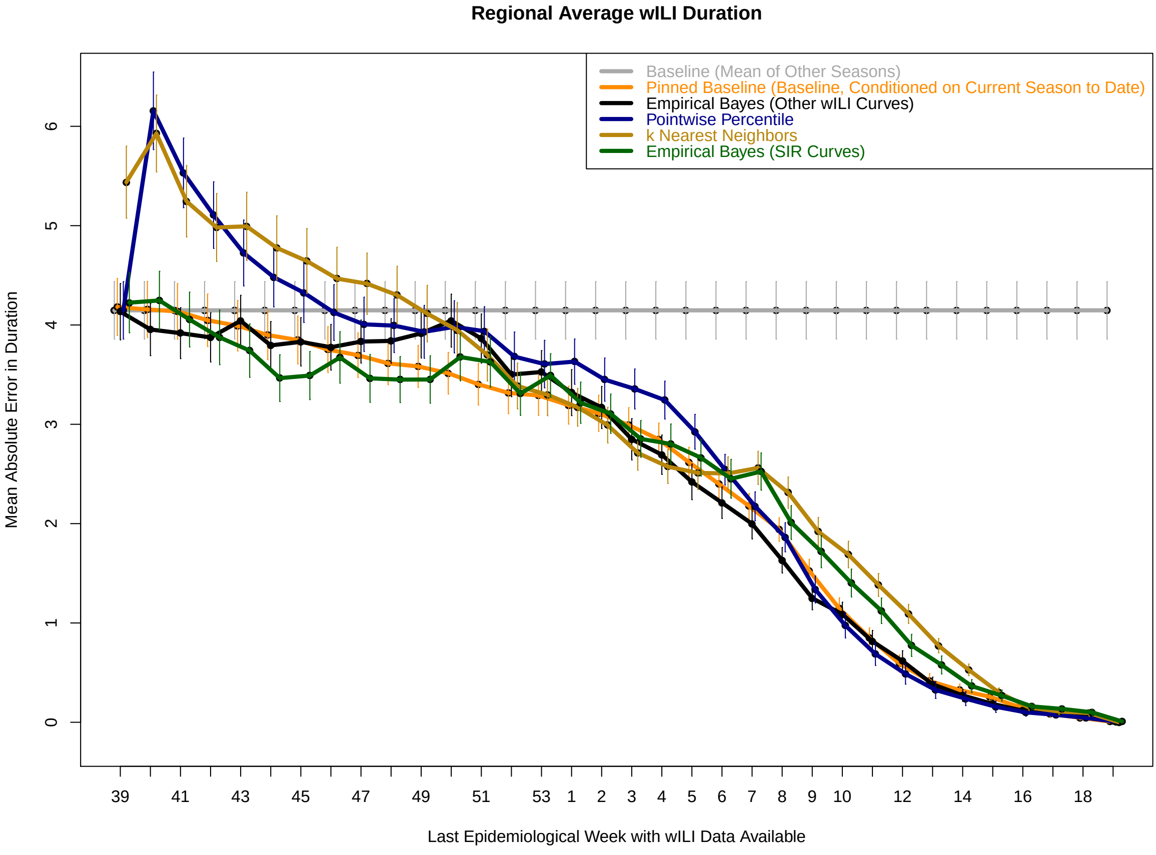

Cross-validated error plots for national and regional point predictions

(B) The point prediction error distribution and mean against predicted onset and peak week.

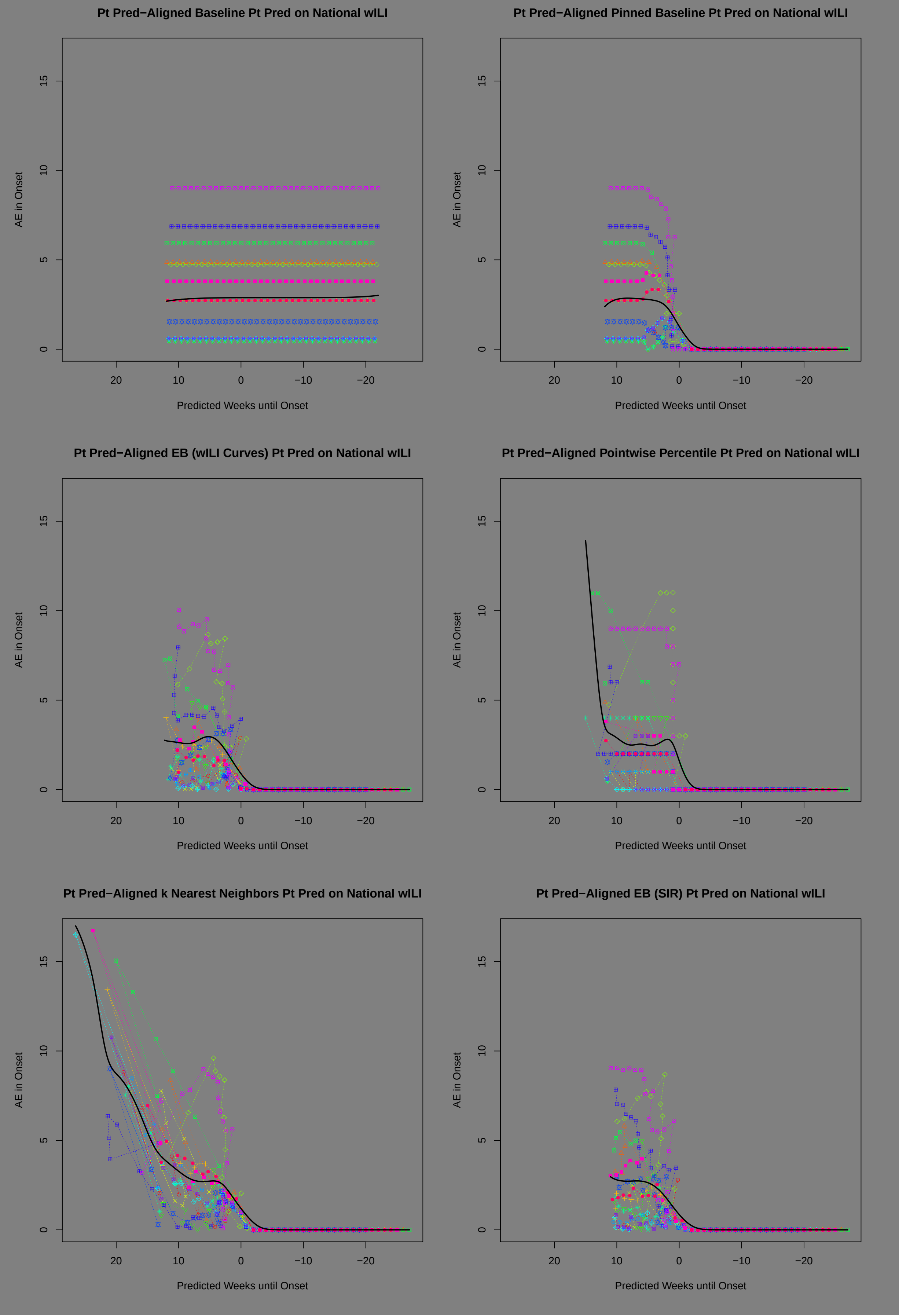

Cross-validated error plots for national and regional point predictions

(B) The point prediction error distribution and mean against predicted onset and peak week.

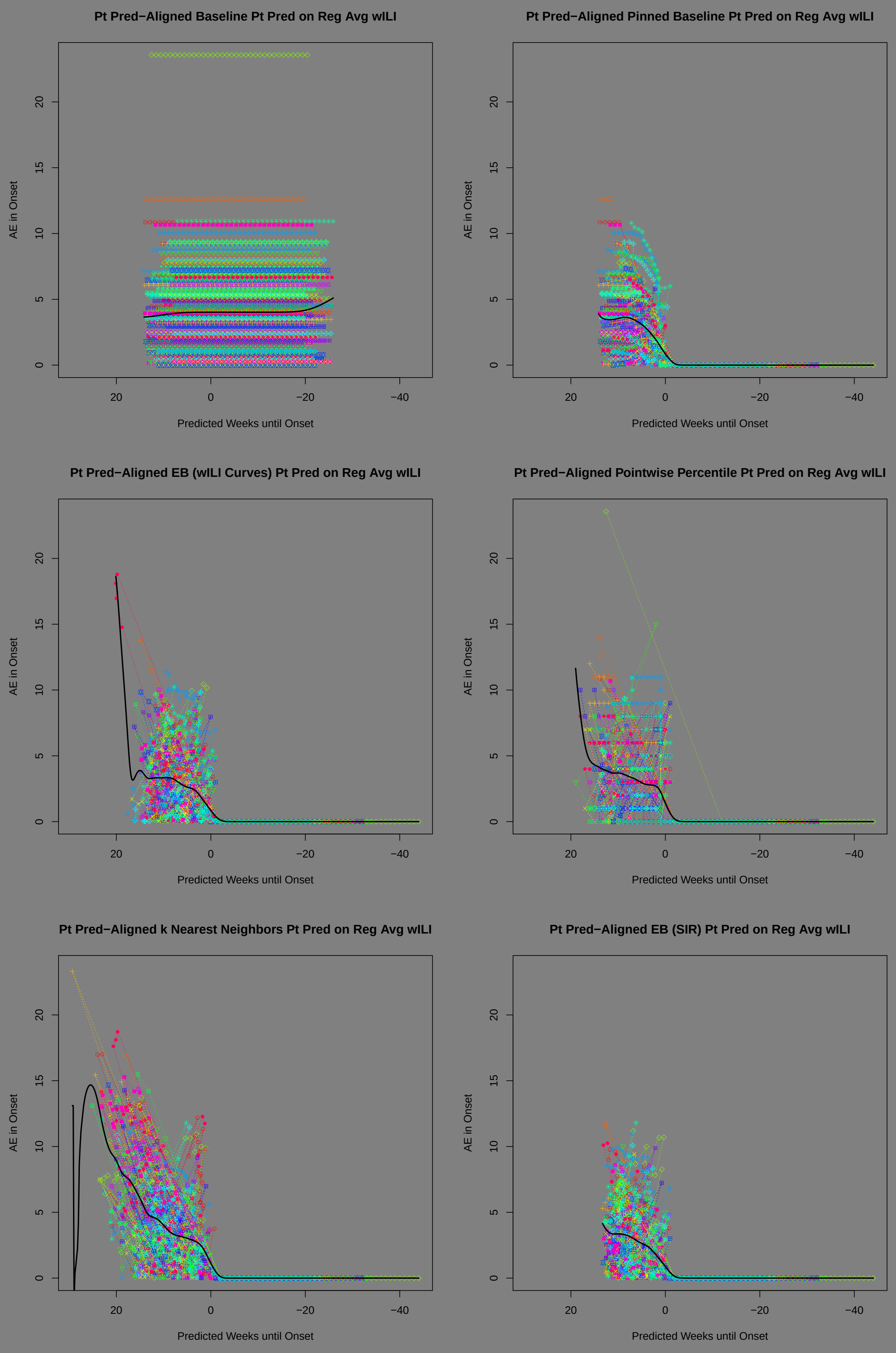

Cross-validated error plots for national and regional point predictions

(B) The point prediction error distribution and mean against predicted onset and peak week.

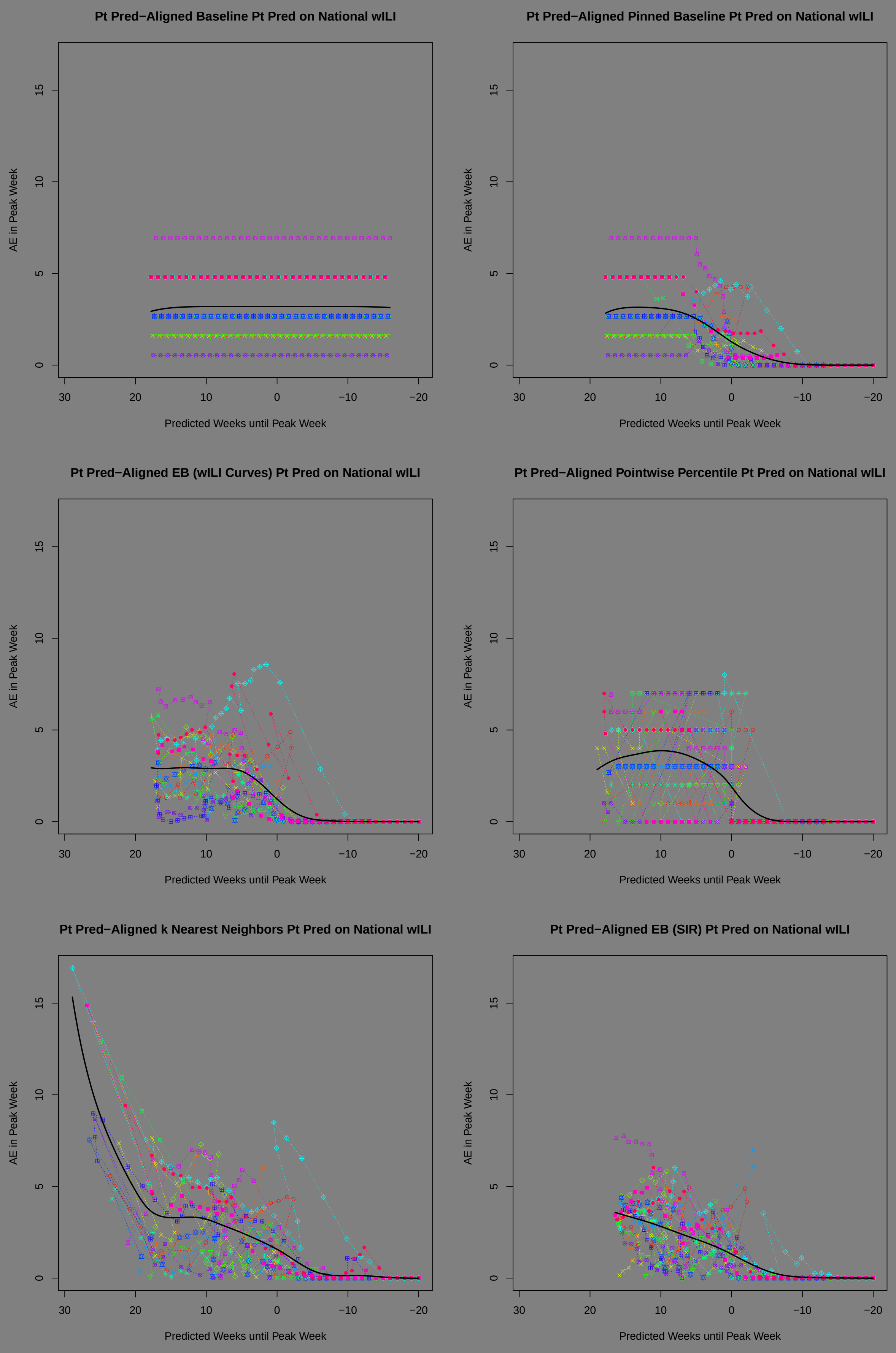

Cross-validated error plots for national and regional point predictions

(B) The point prediction error distribution and mean against predicted onset and peak week.

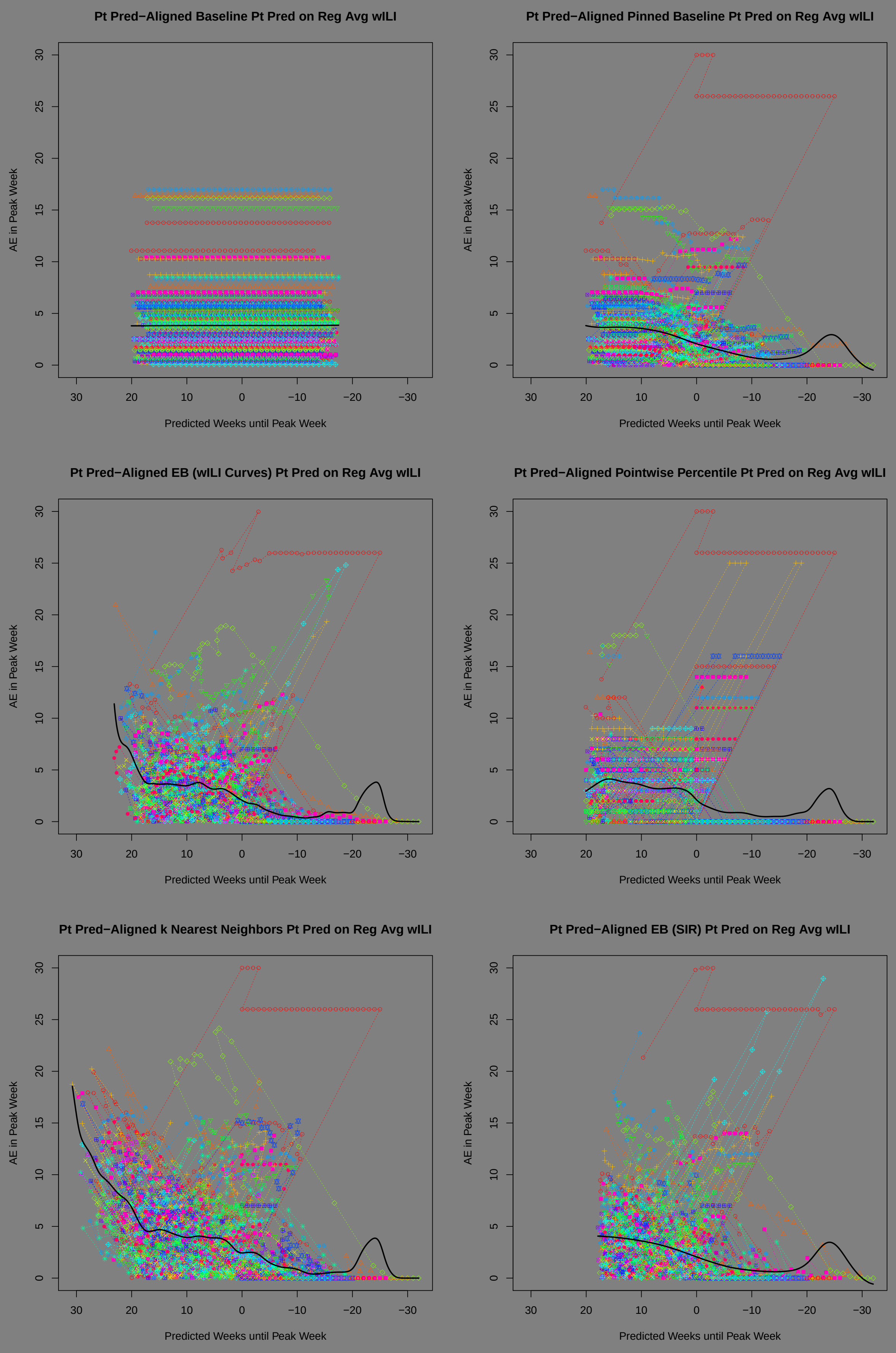

Cross-validated error plots for national and regional point predictions

(B) The point prediction error distribution and mean against predicted onset and peak week.

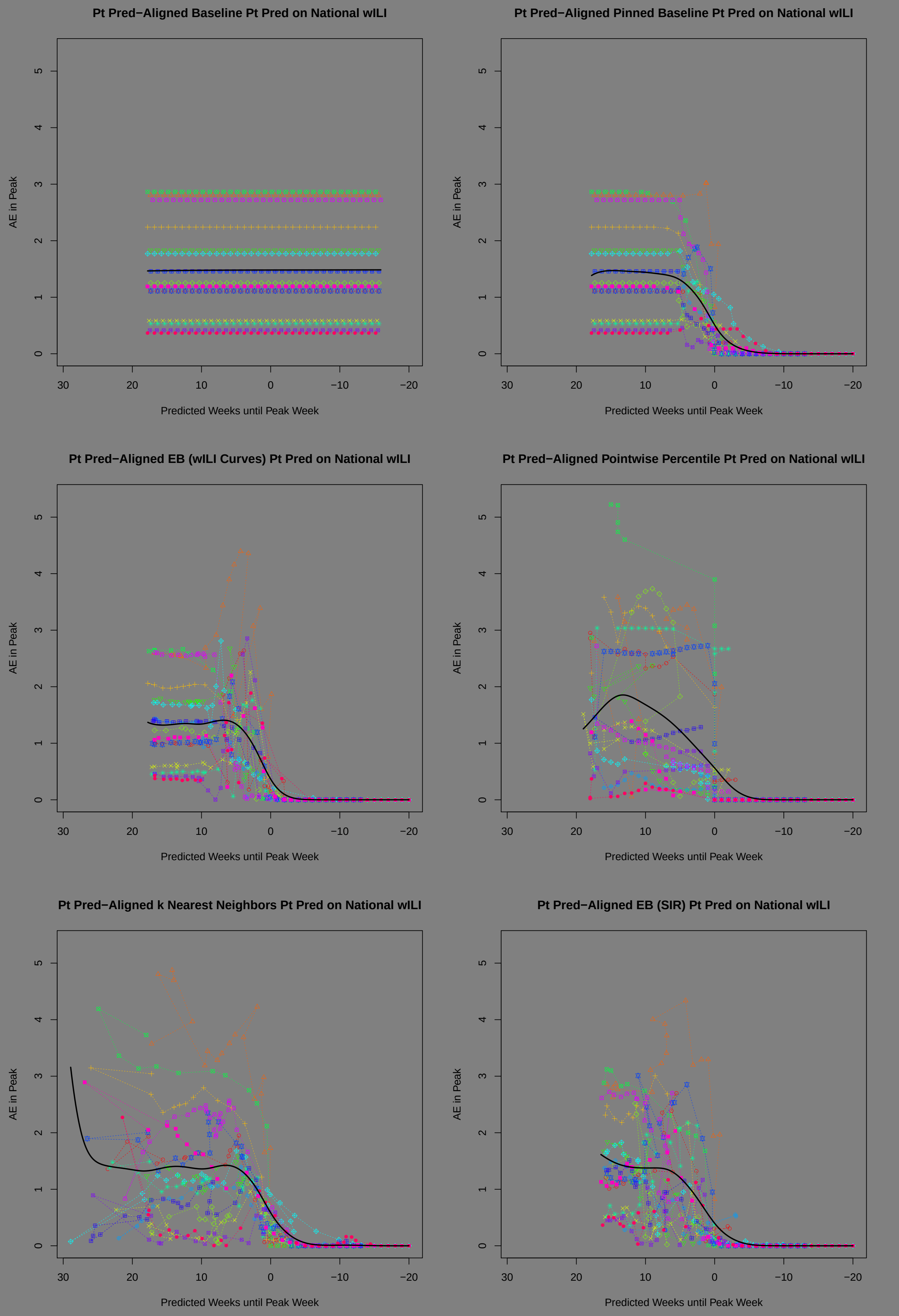

Cross-validated error plots for national and regional point predictions

(B) The point prediction error distribution and mean against predicted onset and peak week.

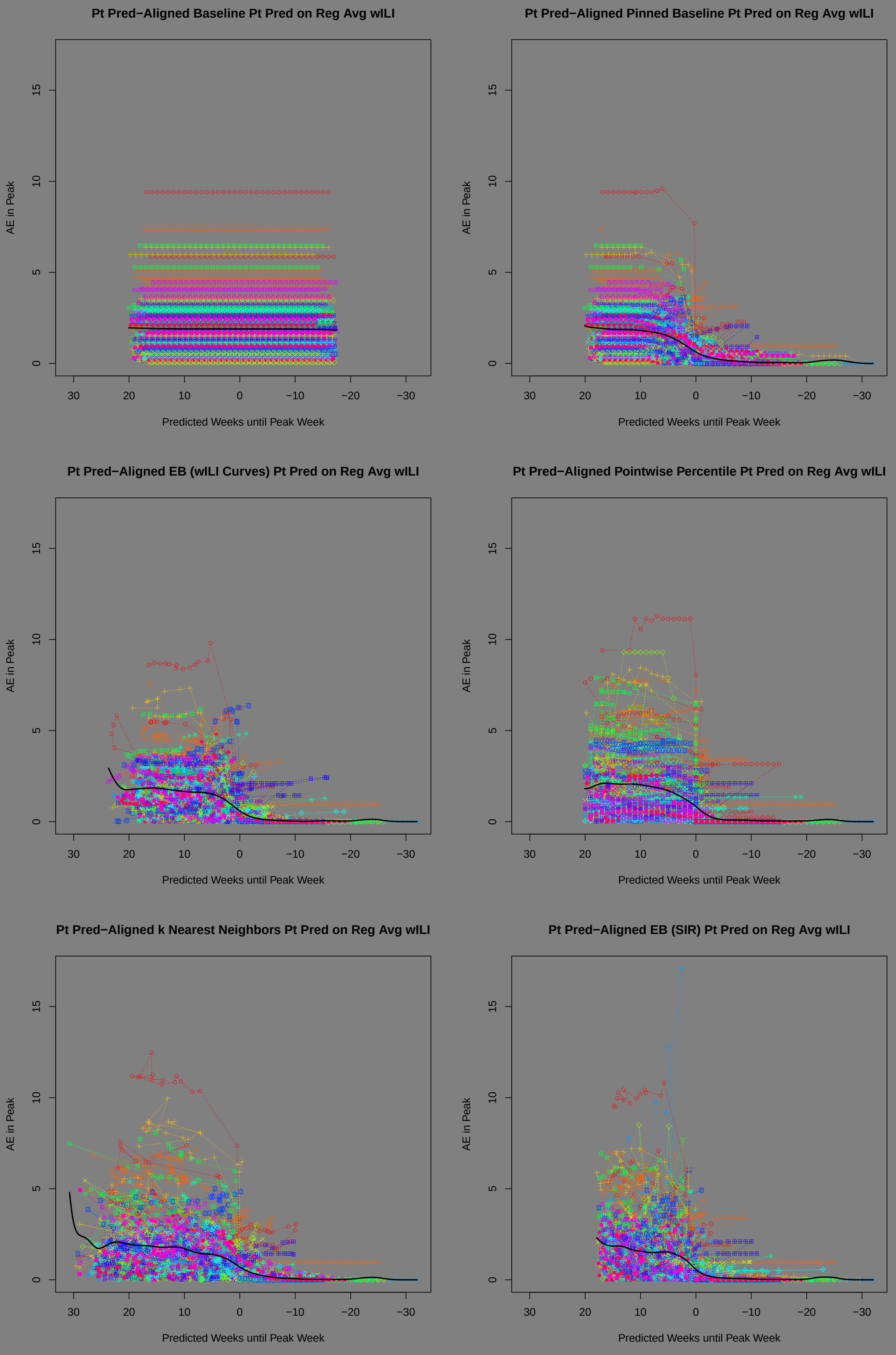

Cross-validated error plots for national and regional point predictions

(B) The point prediction error distribution and mean against predicted onset and peak week.

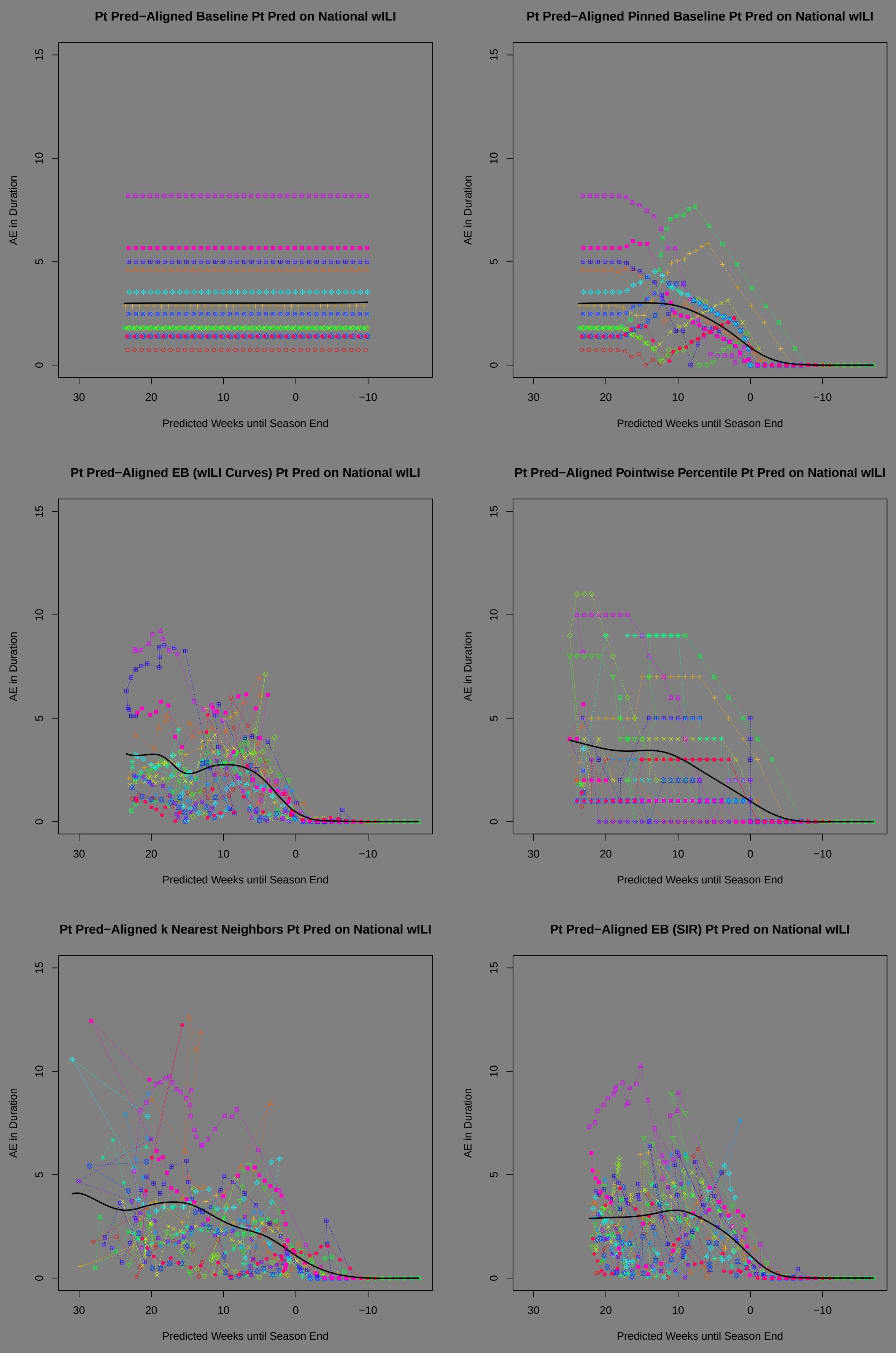

Cross-validated error plots for national and regional point predictions

(B) The point prediction error distribution and mean against predicted onset and peak week.

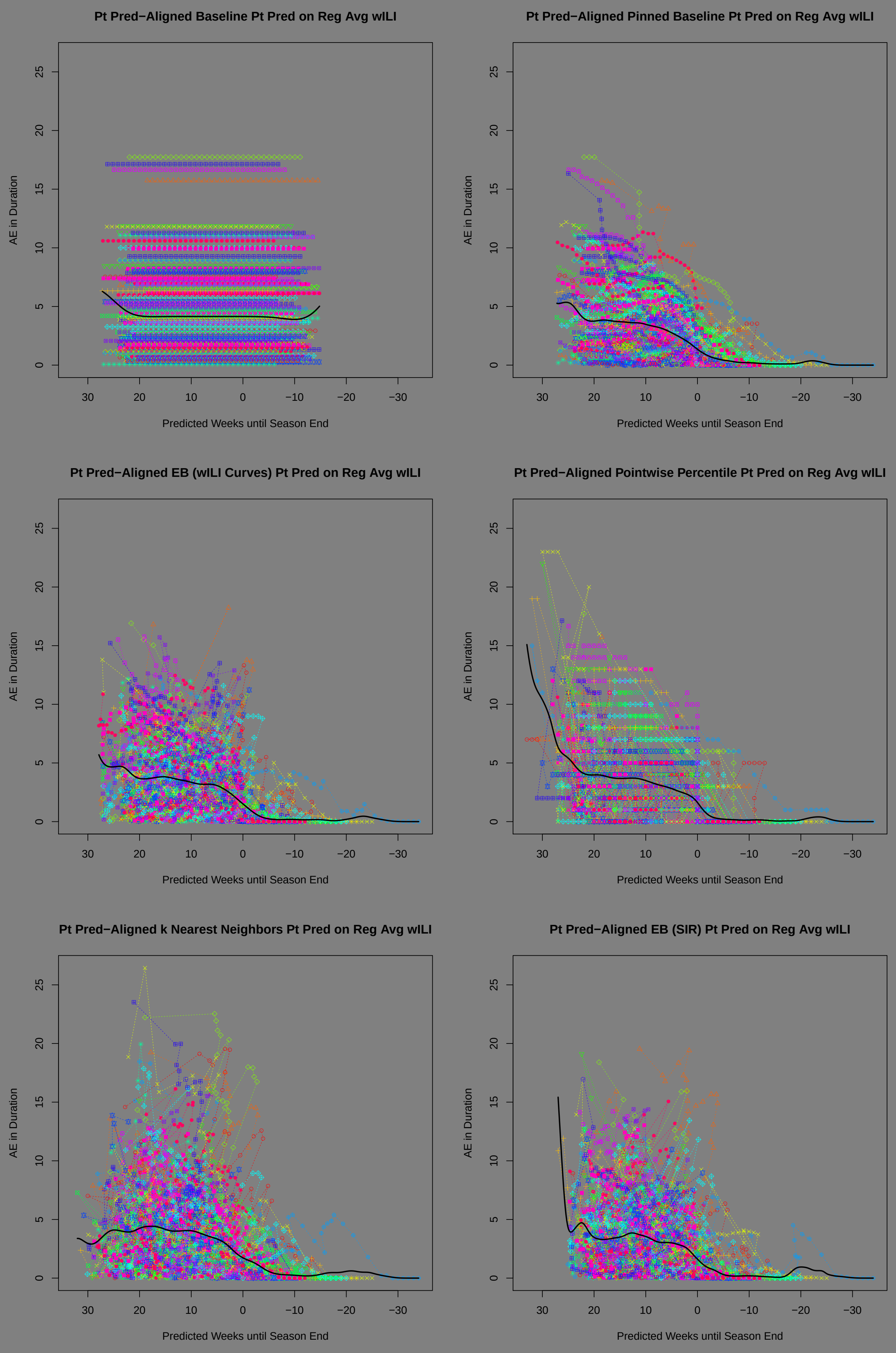

Cross-validated error plots for national and regional point predictions

(C) The estimated probability of point predictions being within certain thresholds of the observed values against predicted onset and peak week.

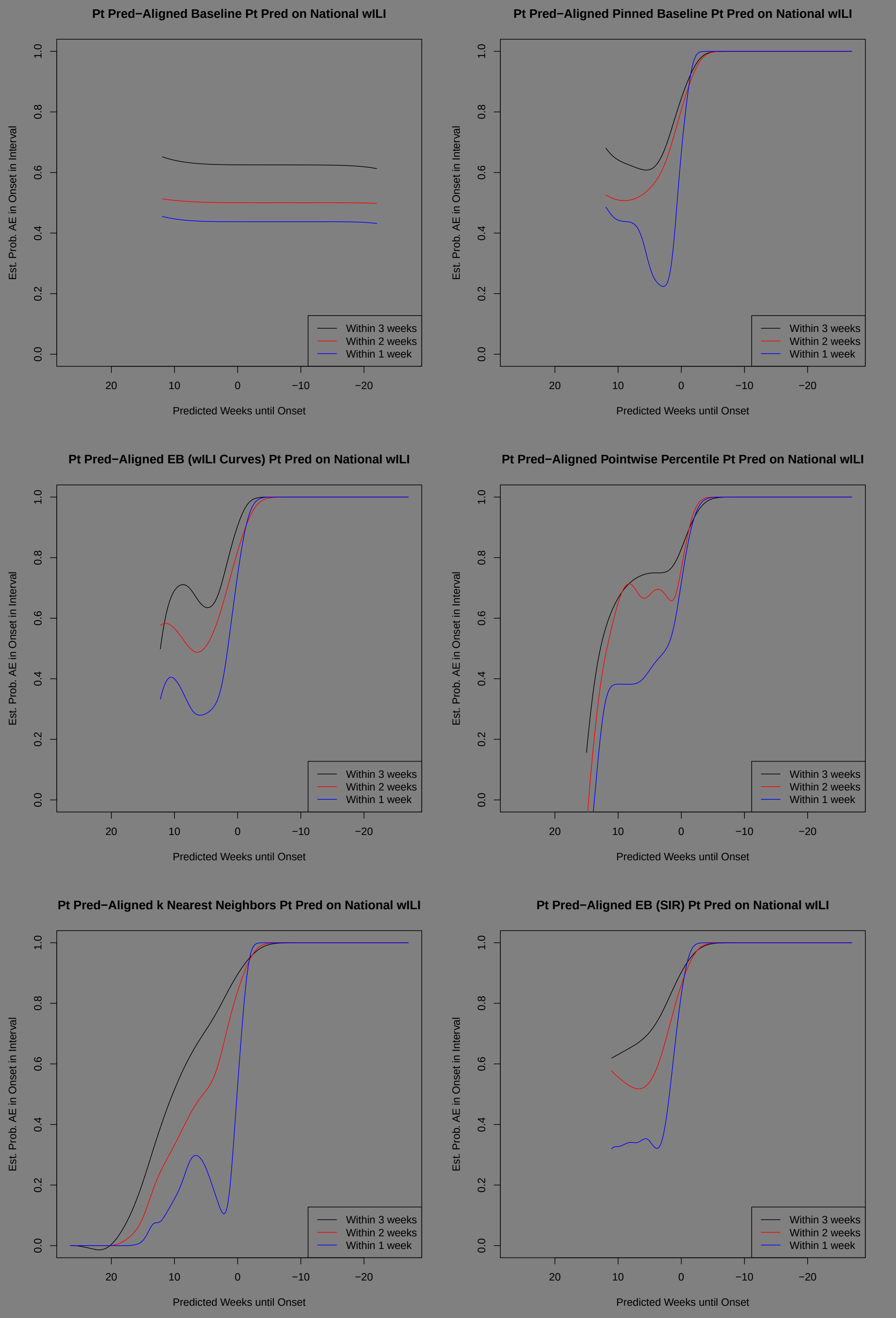

Cross-validated error plots for national and regional point predictions

(C) The estimated probability of point predictions being within certain thresholds of the observed values against predicted onset and peak week.

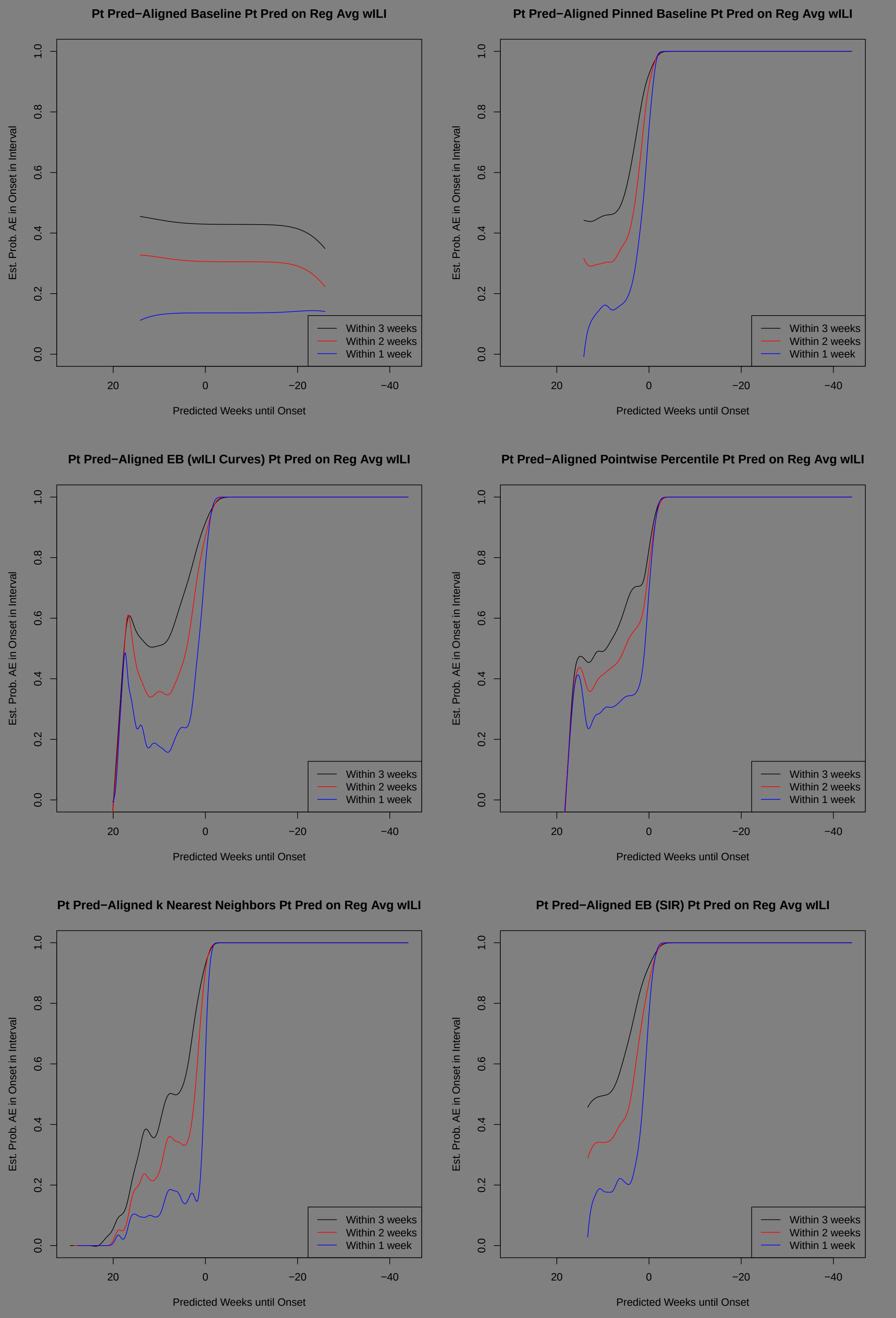

Cross-validated error plots for national and regional point predictions

(C) The estimated probability of point predictions being within certain thresholds of the observed values against predicted onset and peak week.

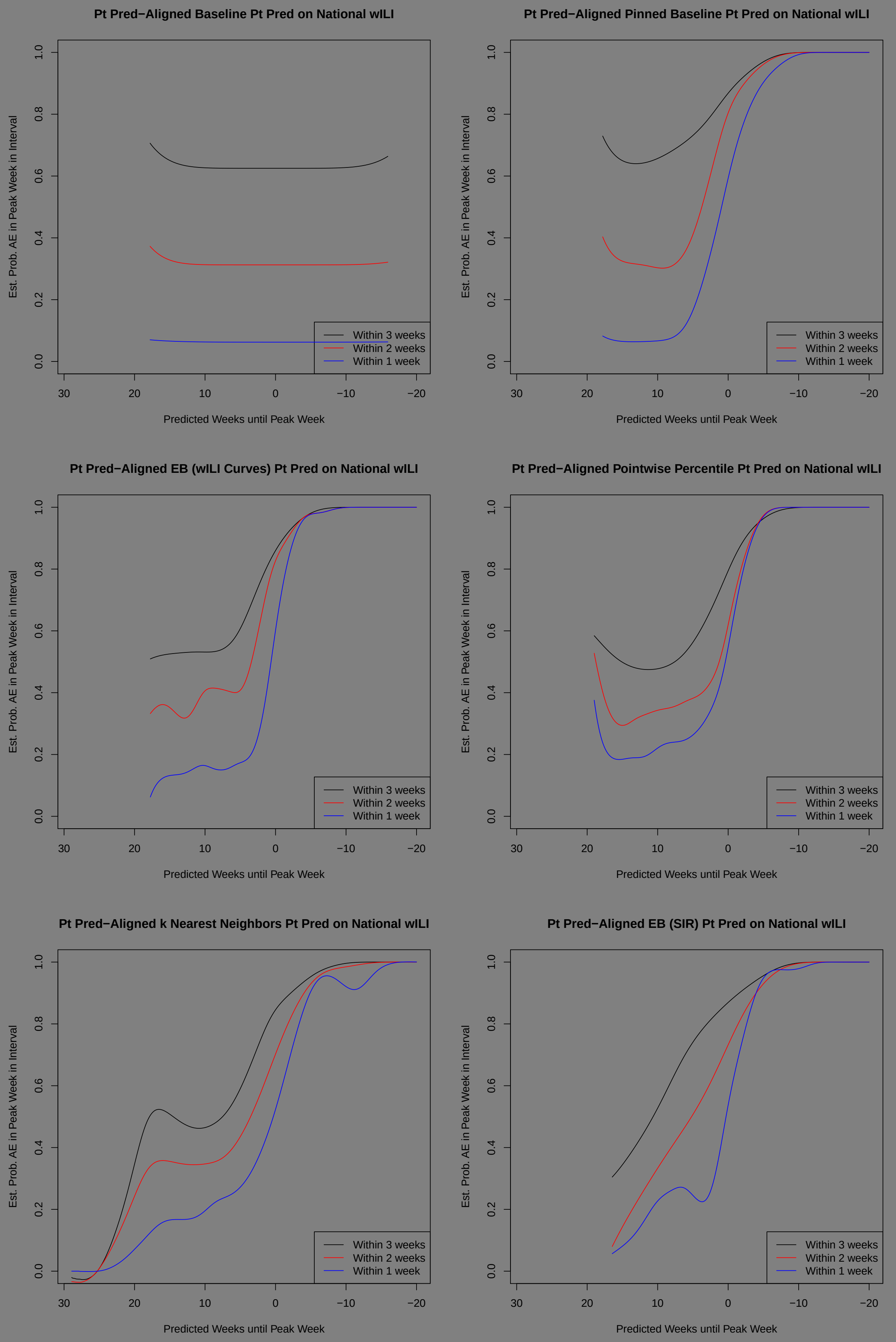

Cross-validated error plots for national and regional point predictions

(C) The estimated probability of point predictions being within certain thresholds of the observed values against predicted onset and peak week.

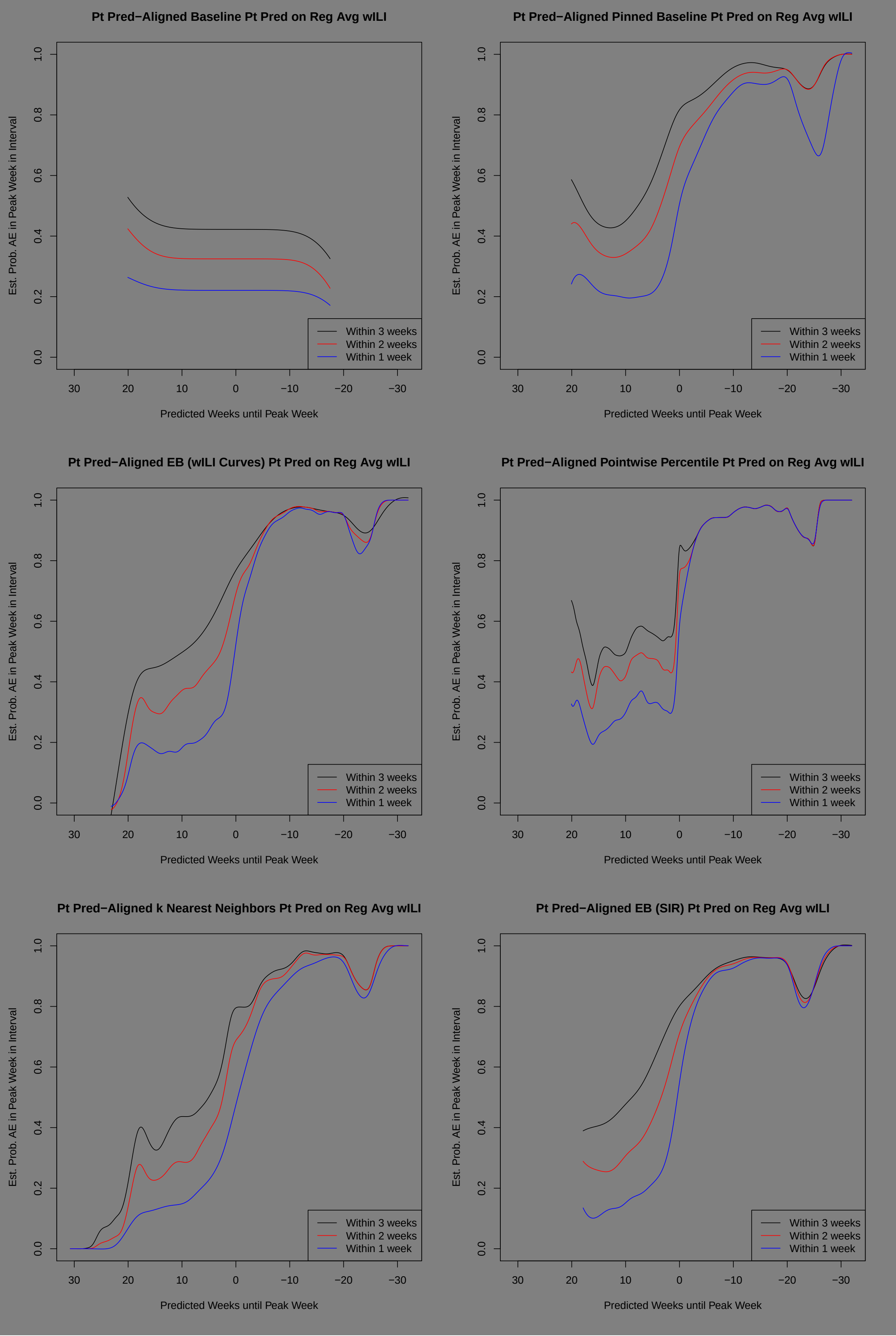

Cross-validated error plots for national and regional point predictions

(C) The estimated probability of point predictions being within certain thresholds of the observed values against predicted onset and peak week.

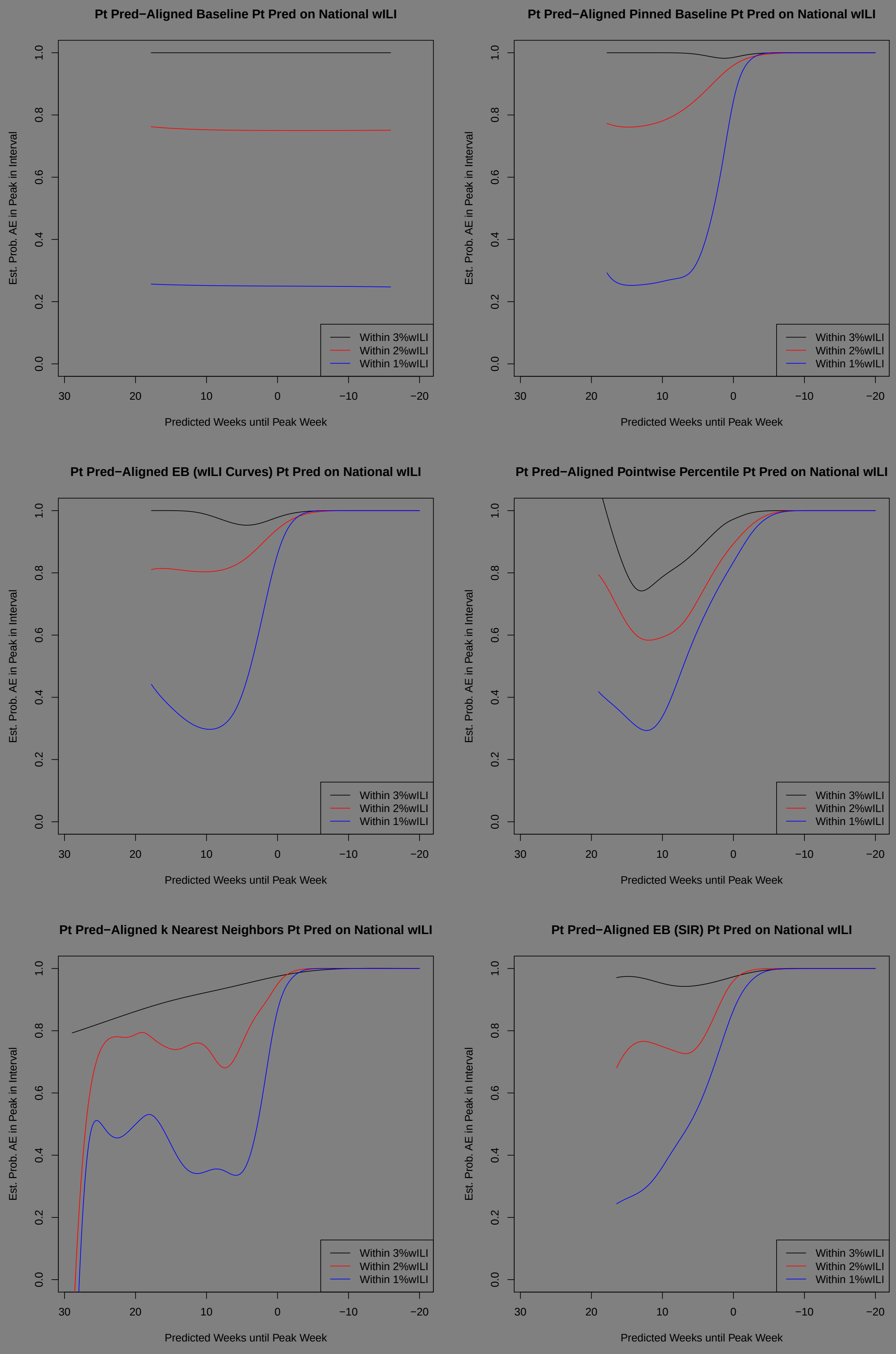

Cross-validated error plots for national and regional point predictions

(C) The estimated probability of point predictions being within certain thresholds of the observed values against predicted onset and peak week.

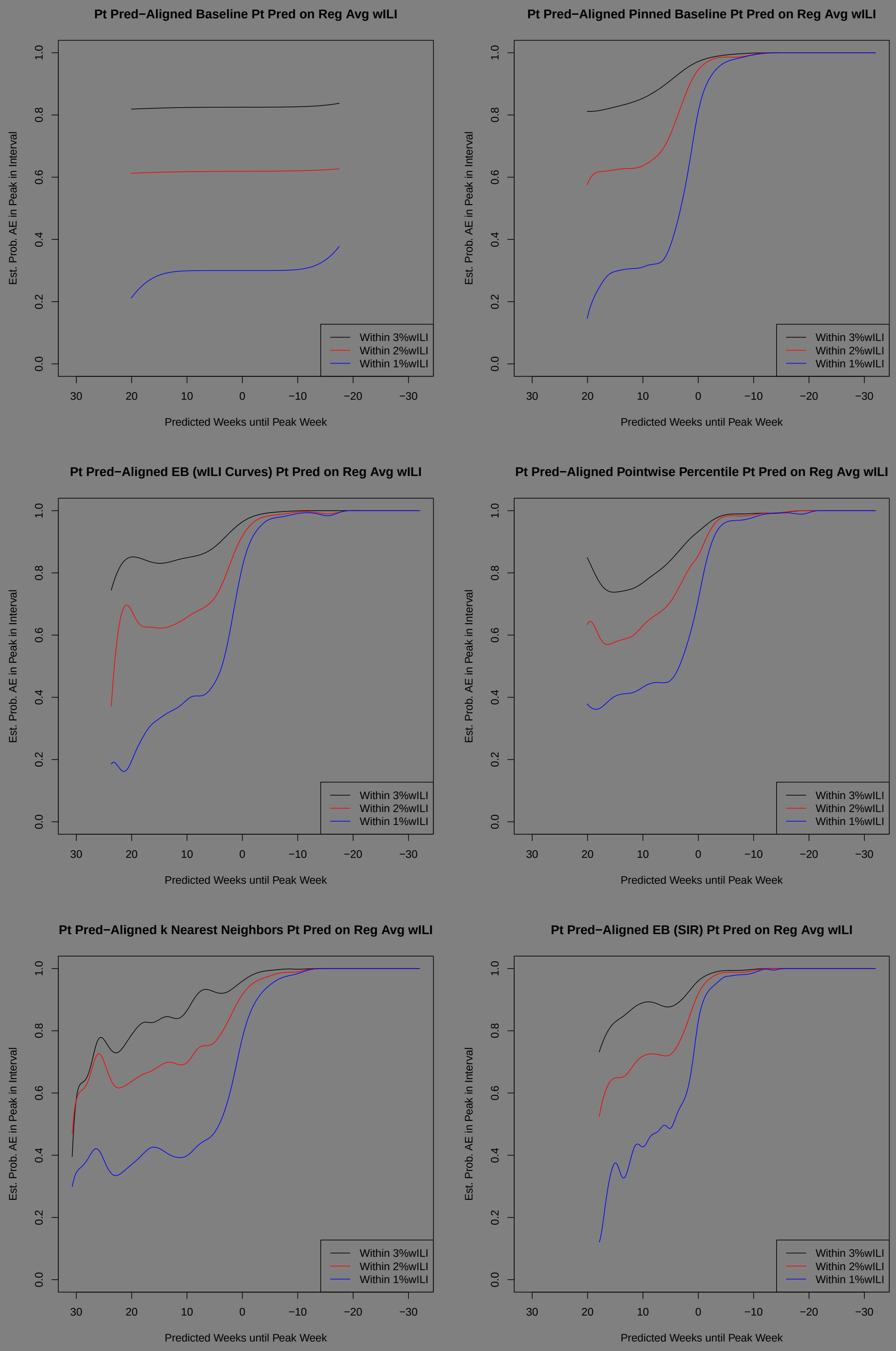

Cross-validated error plots for national and regional point predictions

(C) The estimated probability of point predictions being within certain thresholds of the observed values against predicted onset and peak week.

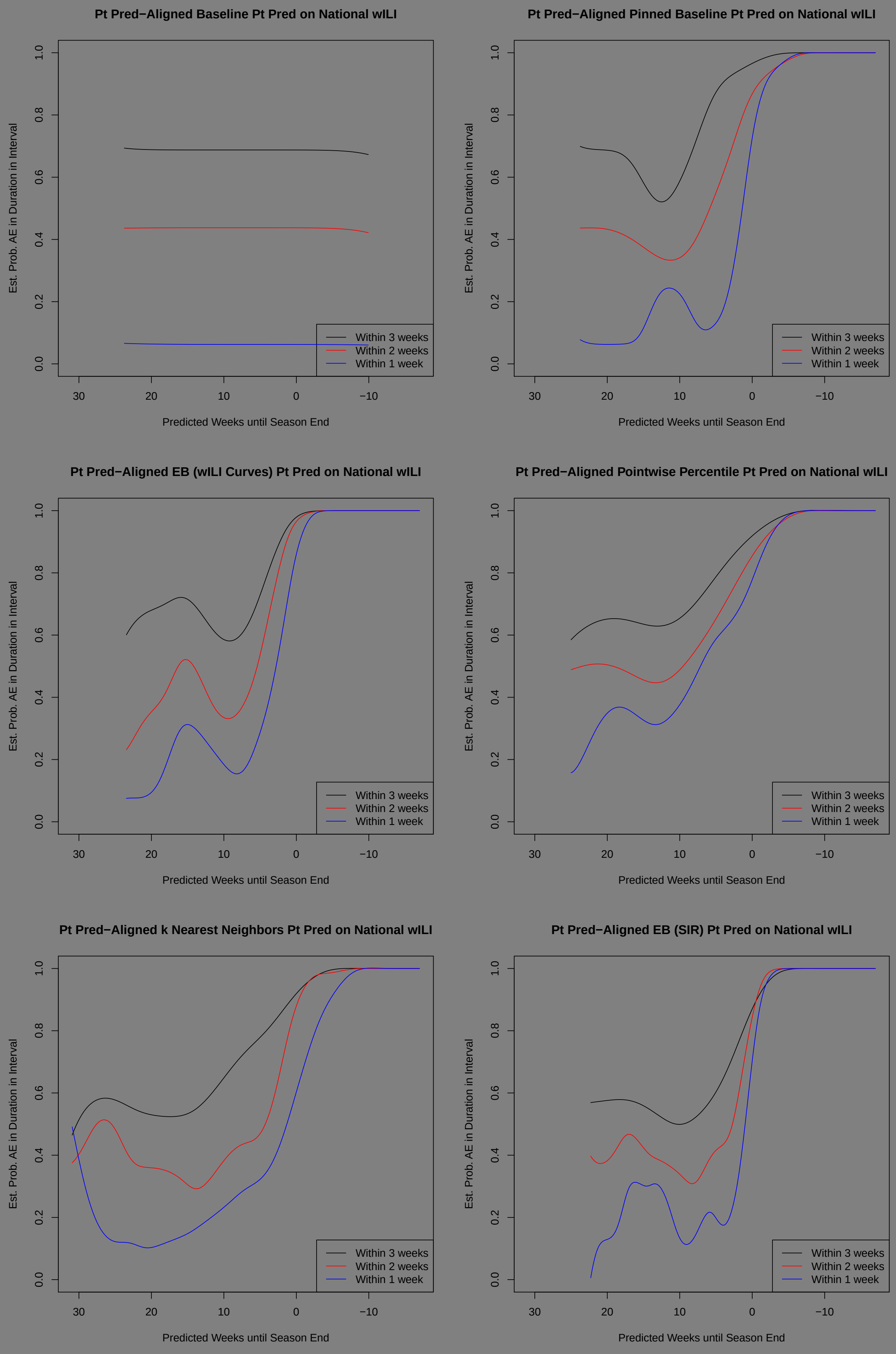

Cross-validated error plots for national and regional point predictions

(C) The estimated probability of point predictions being within certain thresholds of the observed values against predicted onset and peak week.

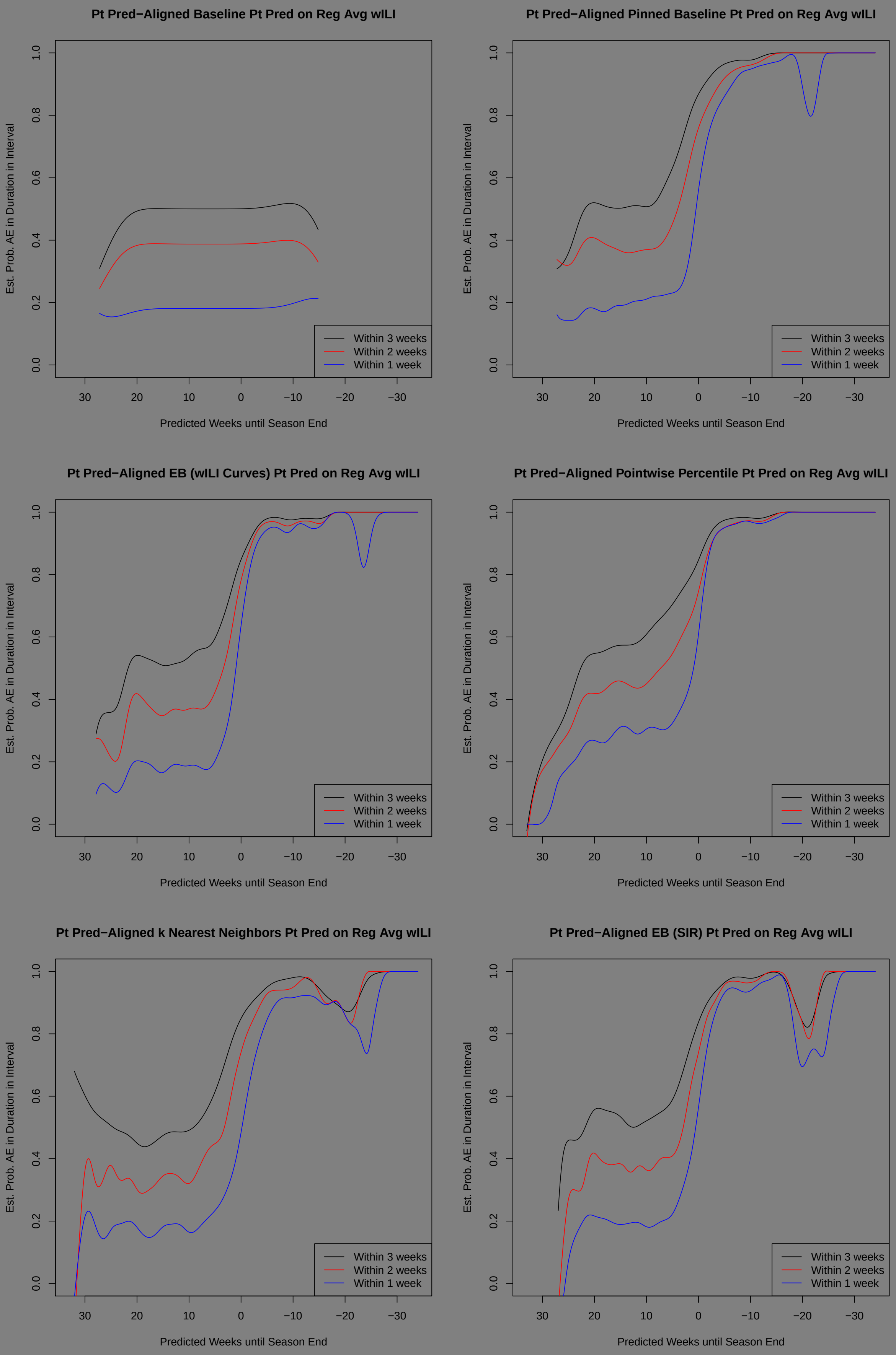

Supplement: S3 Fig — We used leave-one-out cross-validation to estimate the expected error of the point predictors discussed. This figure contains plots of (A) the average point prediction error against epidemic weeks aligned by the observed onset and peak week, (B) the point prediction error distribution and mean against predicted onset and peak week, and (C) the probability of point predictions being within certain thresholds of the observed values against predicted onset and peak week, for the four forecasting targets, both nationally and regionally, for EB and the other forecasters described in the cross-validation section. Estimation of the mean in (B) and probabilities in (C) is performed using locally linear kernel regression [49]. This series of plots was generated with a script that used GNU Parallel to generate plots in parallel on multiple processors [50]. (PDF) [file pcbi.1004382.s003.pdf]
