# Supplementary material for: Flexible Modeling of Epidemics with an Empirical Bayes Framework
Source: PLoS Comput Biol. 2015 Aug 28;11(8):e1004382. doi: 10.1371/journal.pcbi.1004382 (PMC4552841; doi:10.1371/journal.pcbi.1004382)

Cross-validation study of effects of using less seasons to form prior

(A) The average point prediction error against epidemic weeks aligned by the observed onset and peak week.

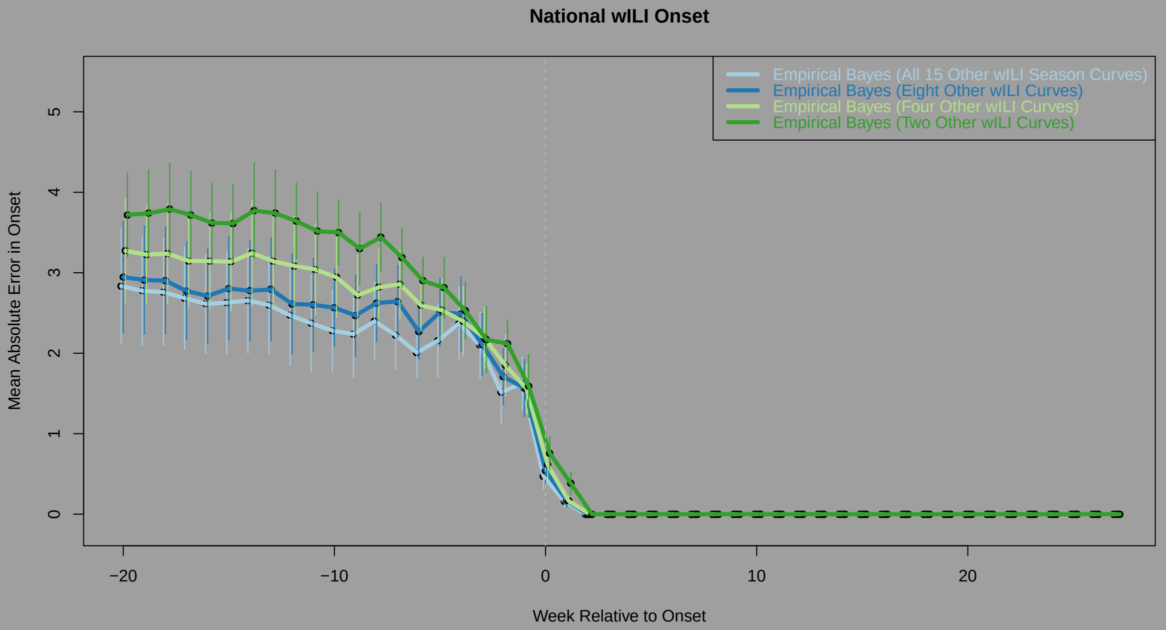

Cross-validation study of effects of using less seasons to form prior

(A) The average point prediction error against epidemic weeks aligned by the observed onset and peak week.

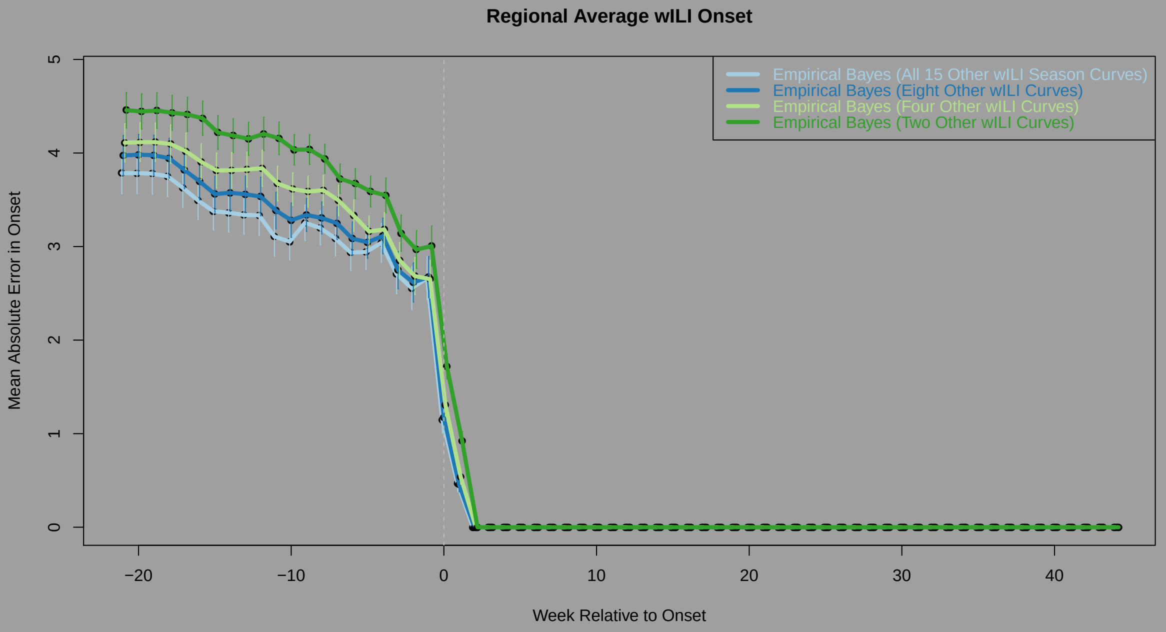

Cross-validation study of effects of using less seasons to form prior

(A) The average point prediction error against epidemic weeks aligned by the observed onset and peak week.

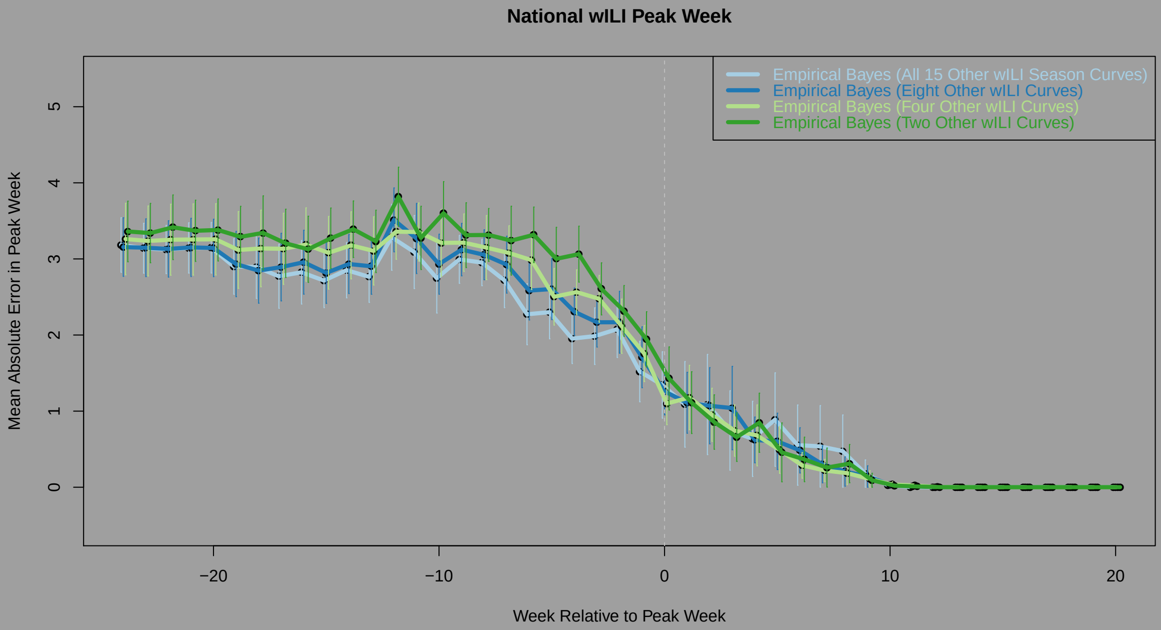

Cross-validation study of effects of using less seasons to form prior

(A) The average point prediction error against epidemic weeks aligned by the observed onset and peak week.

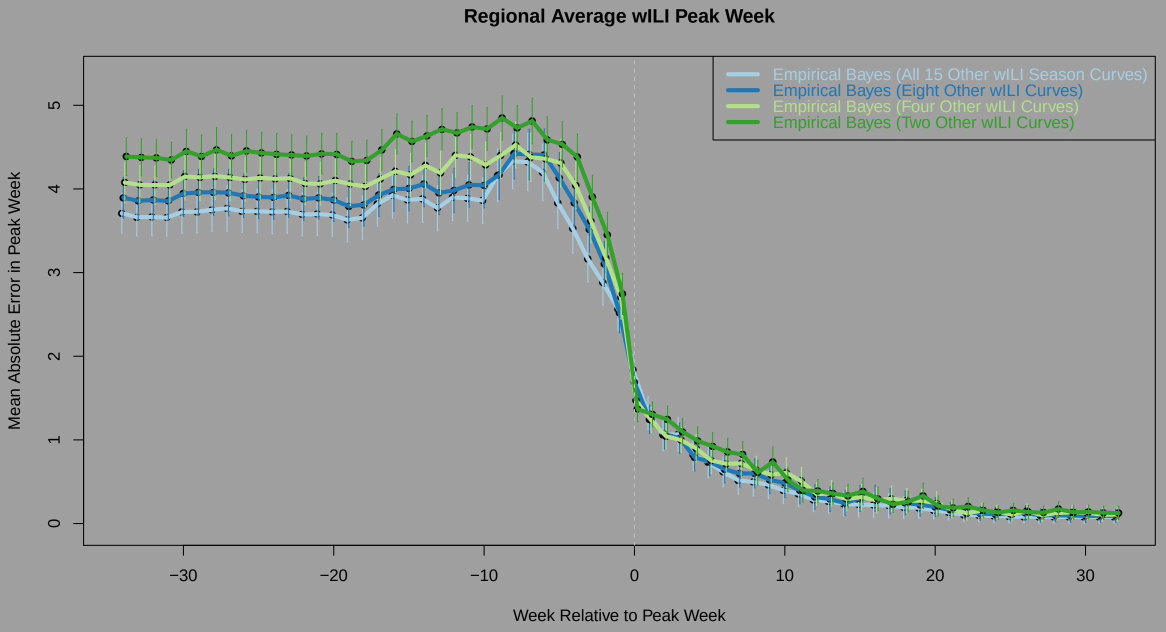

Cross-validation study of effects of using less seasons to form prior

(A) The average point prediction error against epidemic weeks aligned by the observed onset and peak week.

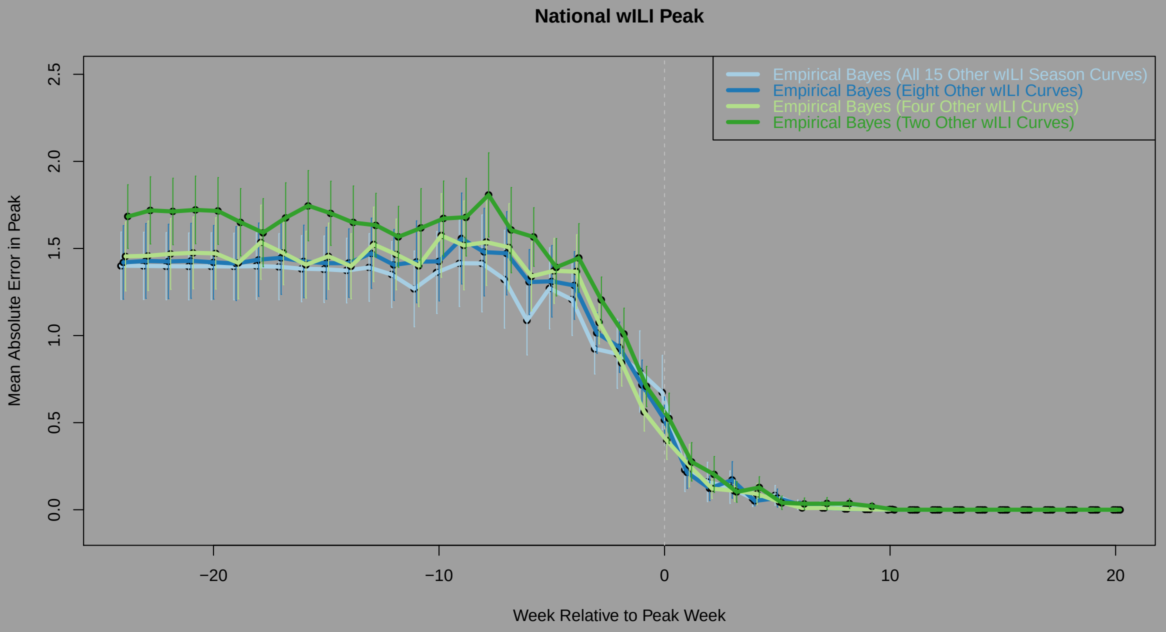

Cross-validation study of effects of using less seasons to form prior

(A) The average point prediction error against epidemic weeks aligned by the observed onset and peak week.

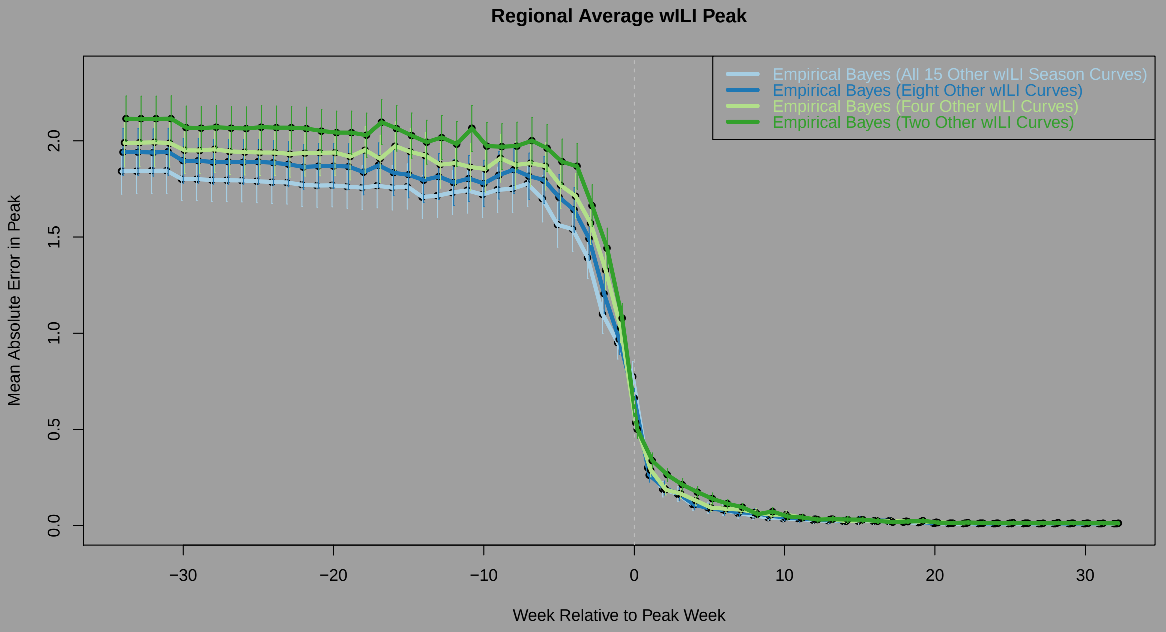

Cross-validation study of effects of using less seasons to form prior

(A) The average point prediction error against epidemic weeks aligned by the observed onset and peak week.

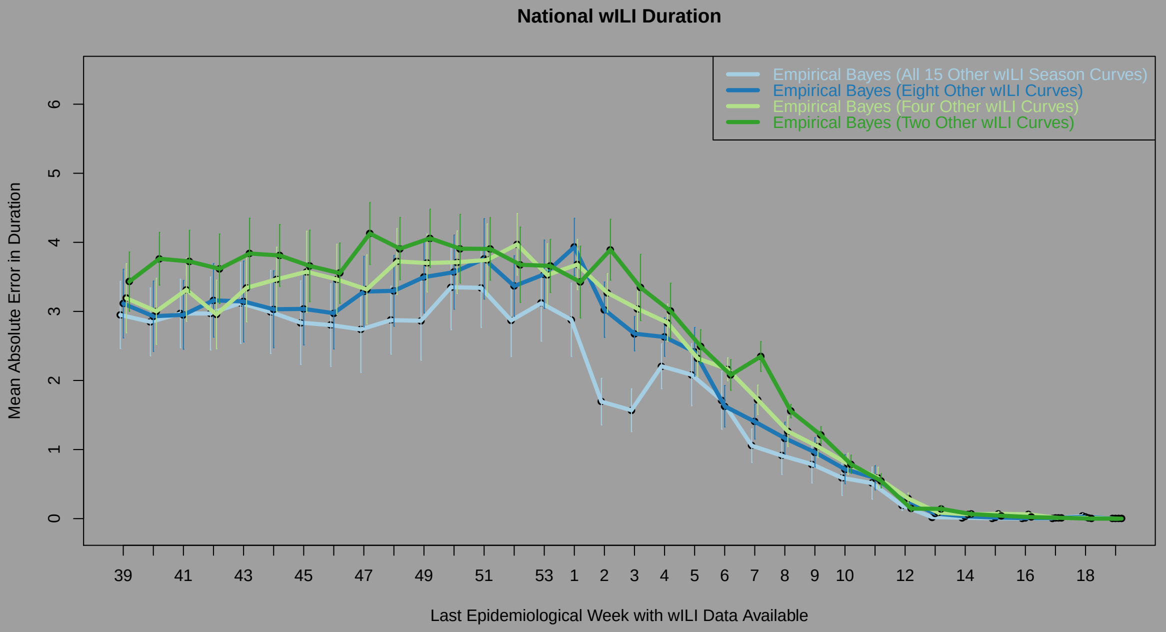

Cross-validation study of effects of using less seasons to form prior

(A) The average point prediction error against epidemic weeks aligned by the observed onset and peak week.

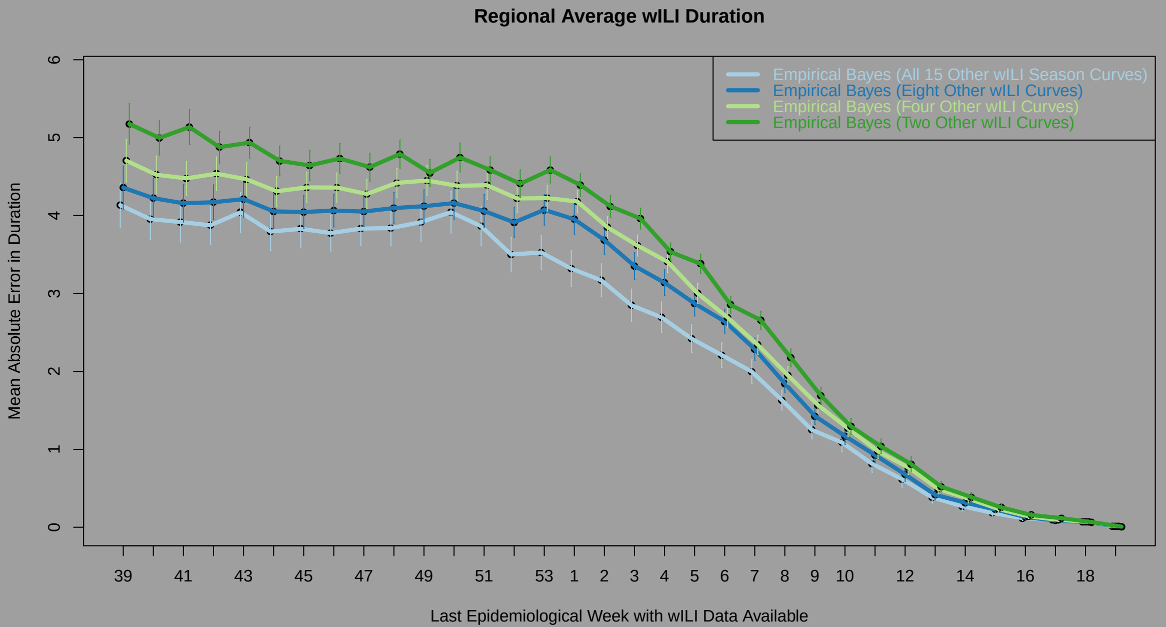

Cross-validation study of effects of using less seasons to form prior.

(B) The coverage of 90% credible intervals against epidemic weeks aligned by the observed onset and peak week.

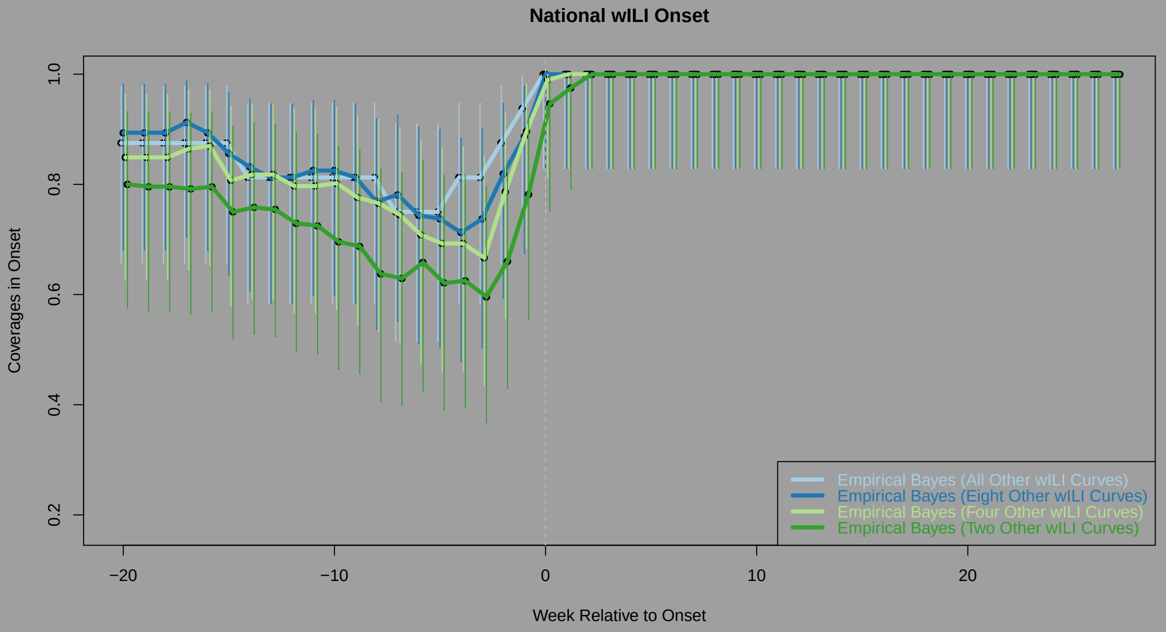

Cross-validation study of effects of using less seasons to form prior.

(B) The coverage of 90% credible intervals against epidemic weeks aligned by the observed onset and peak week.

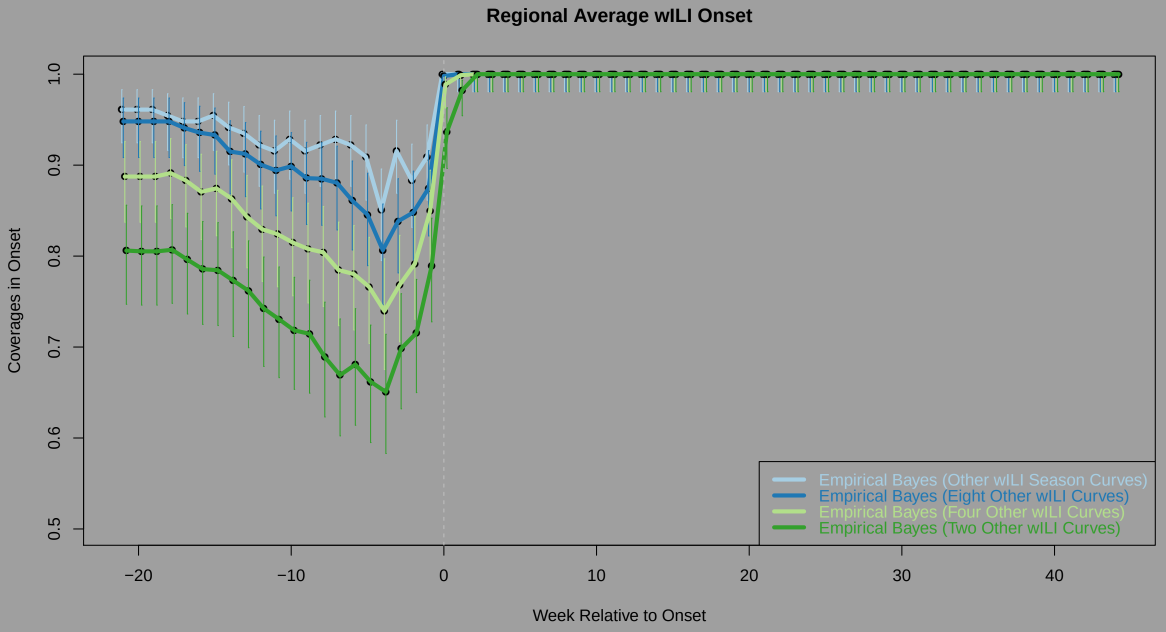

Cross-validation study of effects of using less seasons to form prior.

(B) The coverage of 90% credible intervals against epidemic weeks aligned by the observed onset and peak week.

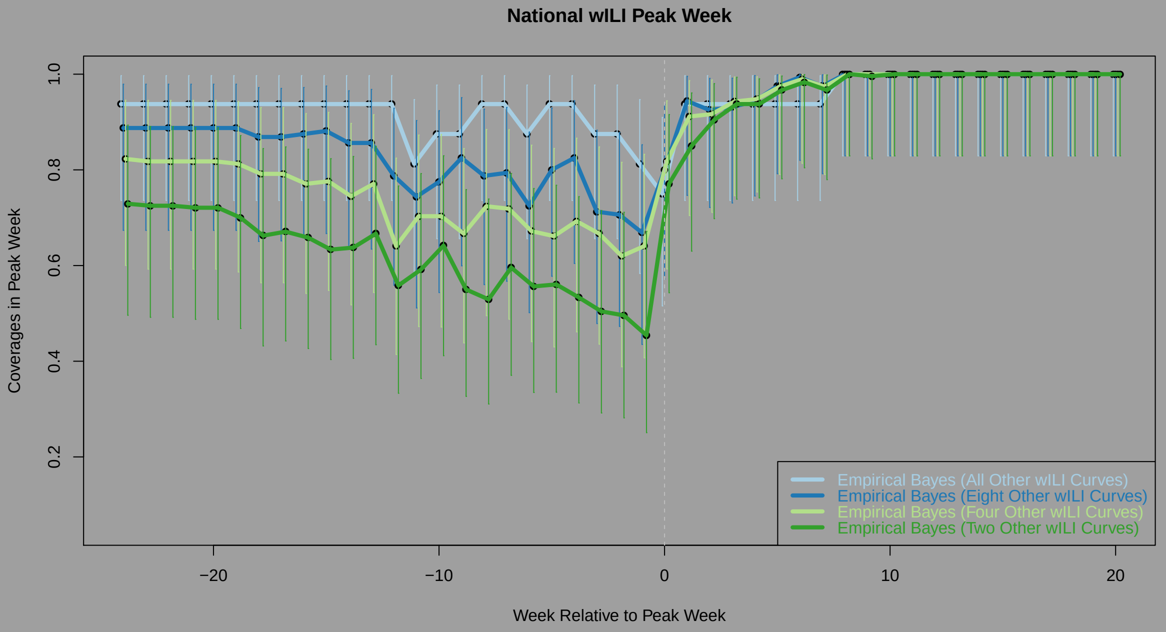

Cross-validation study of effects of using less seasons to form prior.

(B) The coverage of 90% credible intervals against epidemic weeks aligned by the observed onset and peak week.

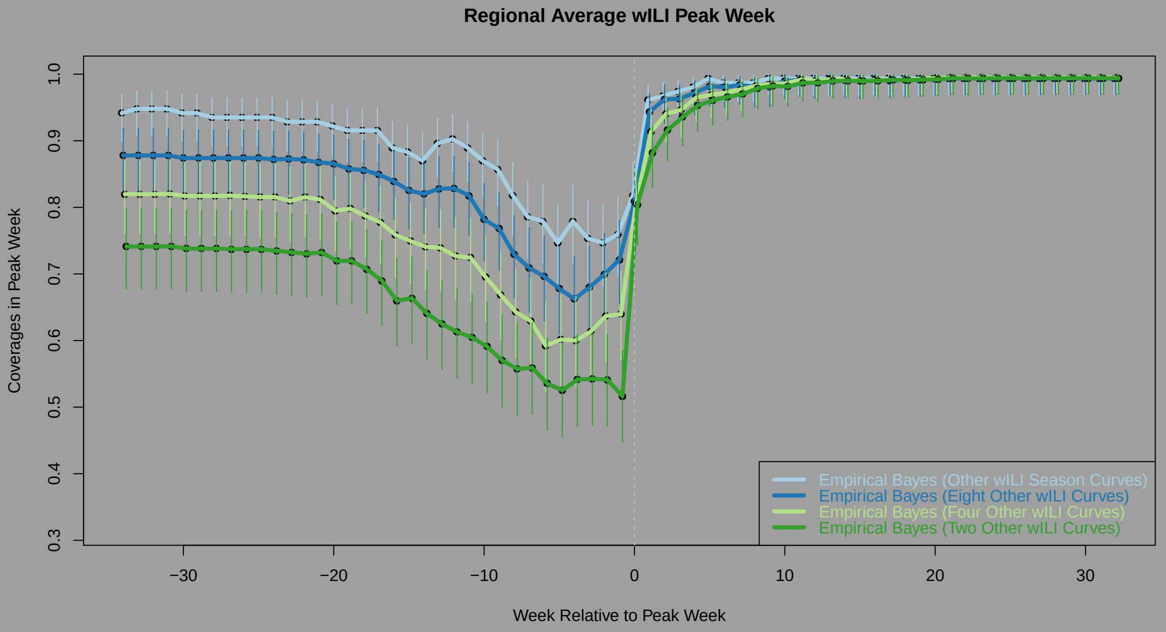

Cross-validation study of effects of using less seasons to form prior.

(B) The coverage of 90% credible intervals against epidemic weeks aligned by the observed onset and peak week.

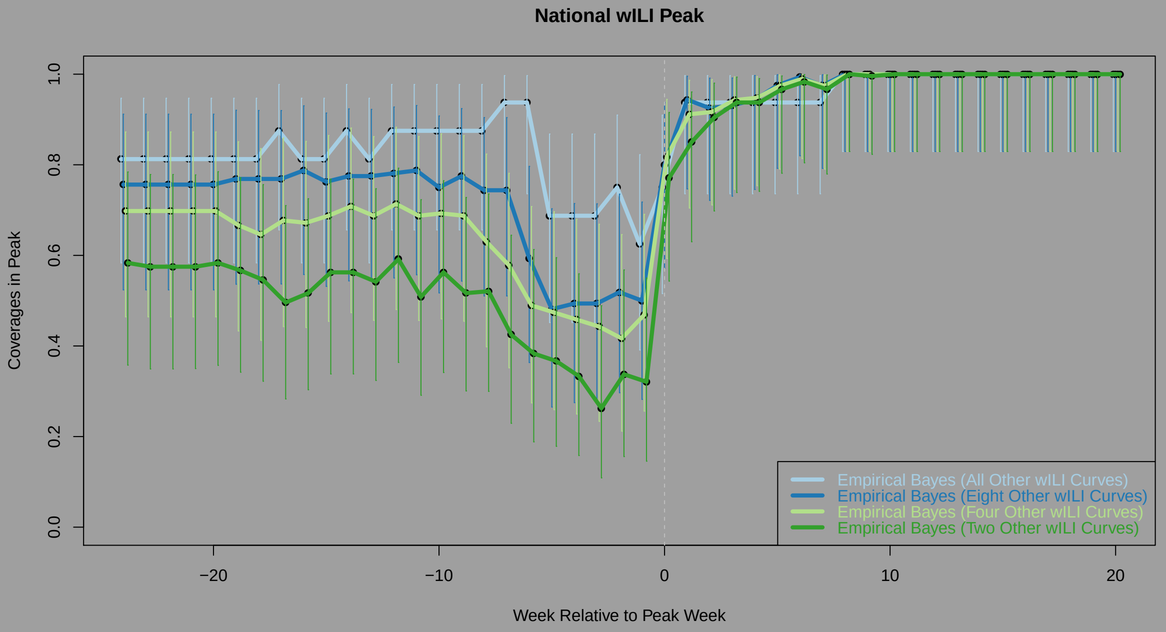

Cross-validation study of effects of using less seasons to form prior.

(B) The coverage of 90% credible intervals against epidemic weeks aligned by the observed onset and peak week.

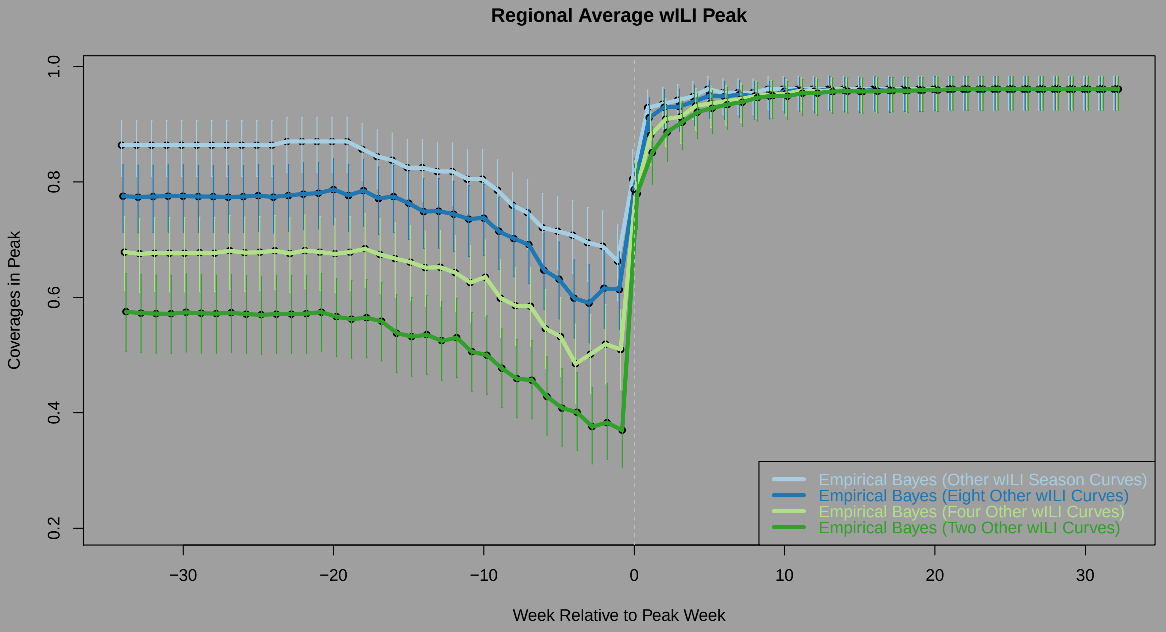

Cross-validation study of effects of using less seasons to form prior.

(B) The coverage of 90% credible intervals against epidemic weeks aligned by the observed onset and peak week.

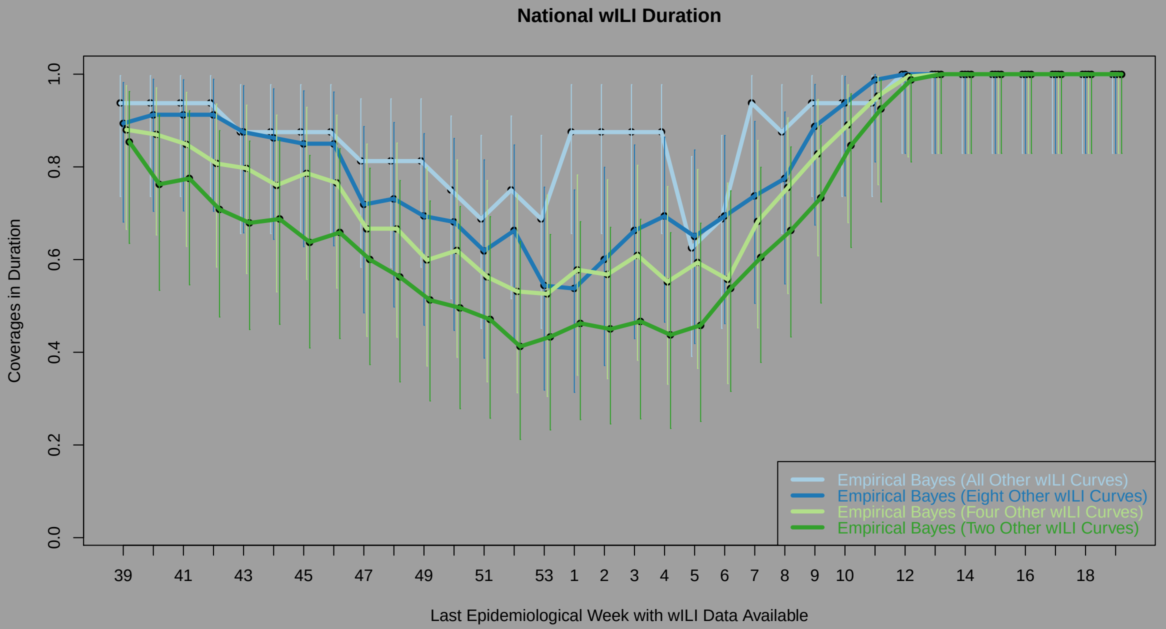

Cross-validation study of effects of using less seasons to form prior.

(B) The coverage of 90% credible intervals against epidemic weeks aligned by the observed onset and peak week.

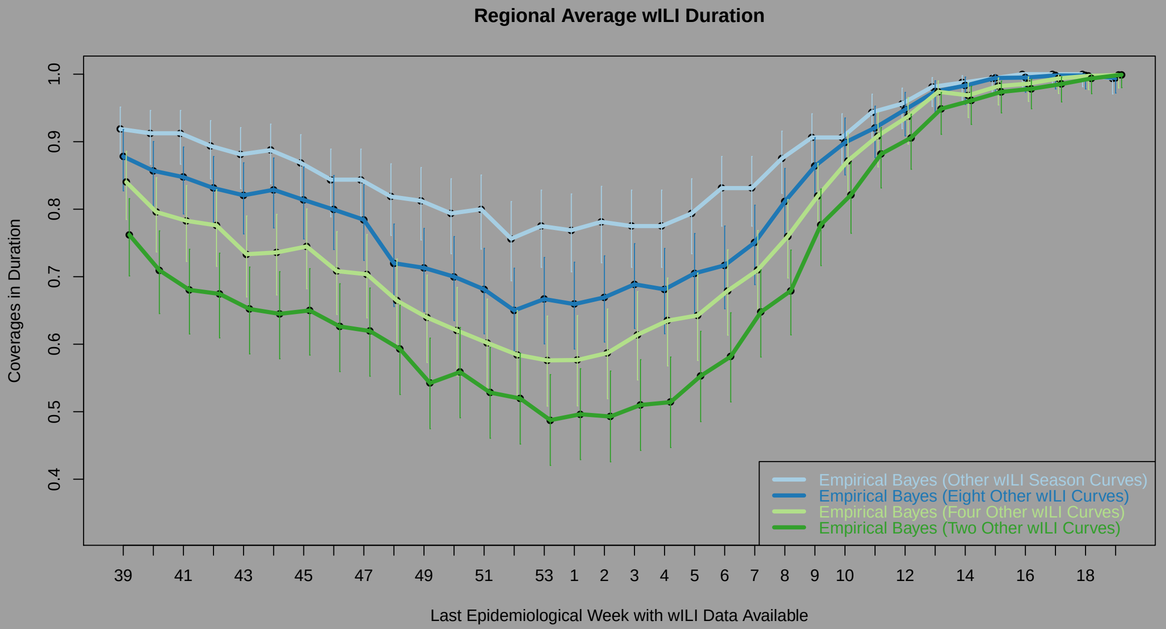

Cross-validation study of effects of using less seasons to form prior.

(C) The point prediction error distribution and mean against predicted onset and peak week.

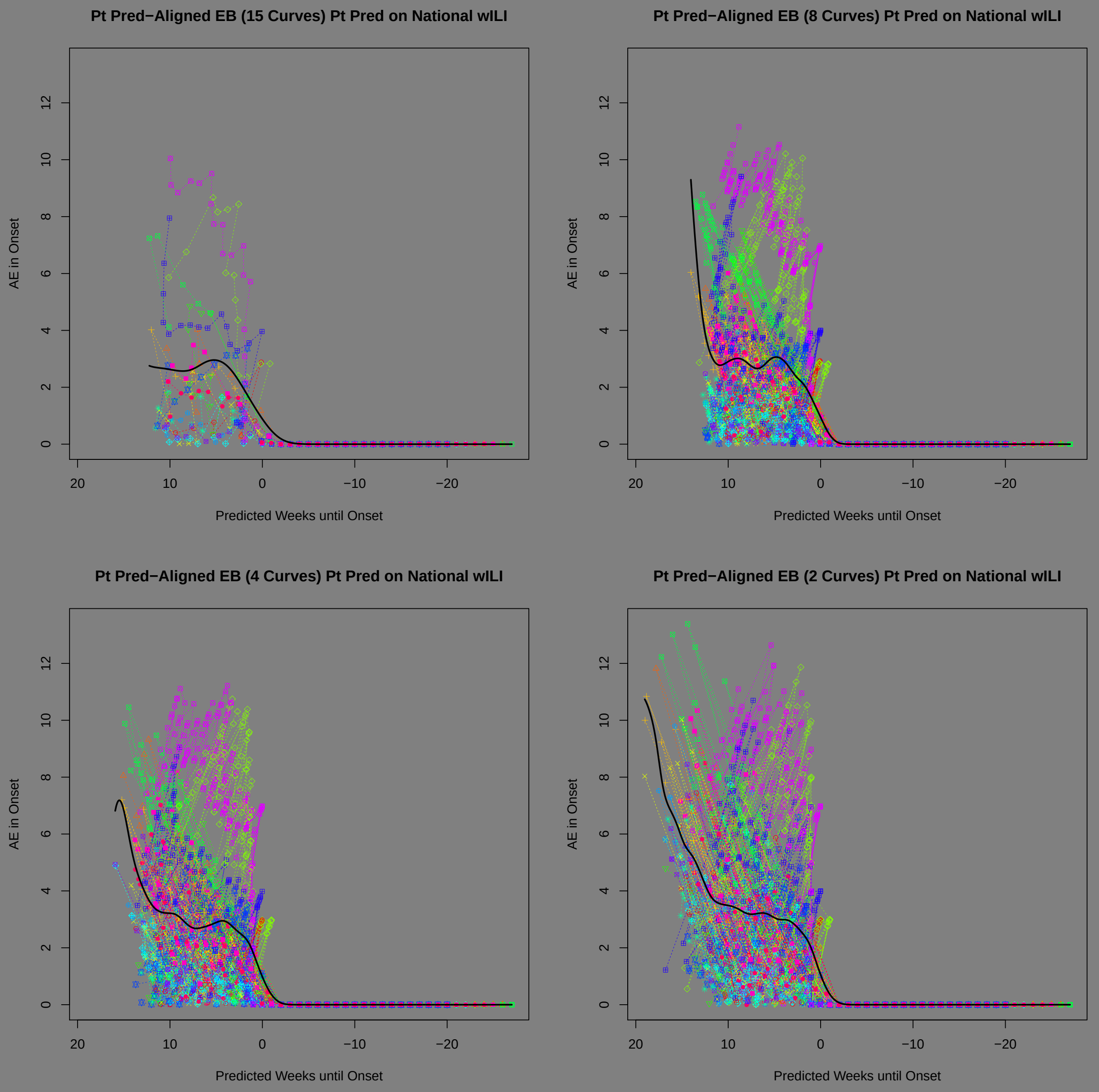

Cross-validation study of effects of using less seasons to form prior.

(C) The point prediction error distribution and mean against predicted onset and peak week.

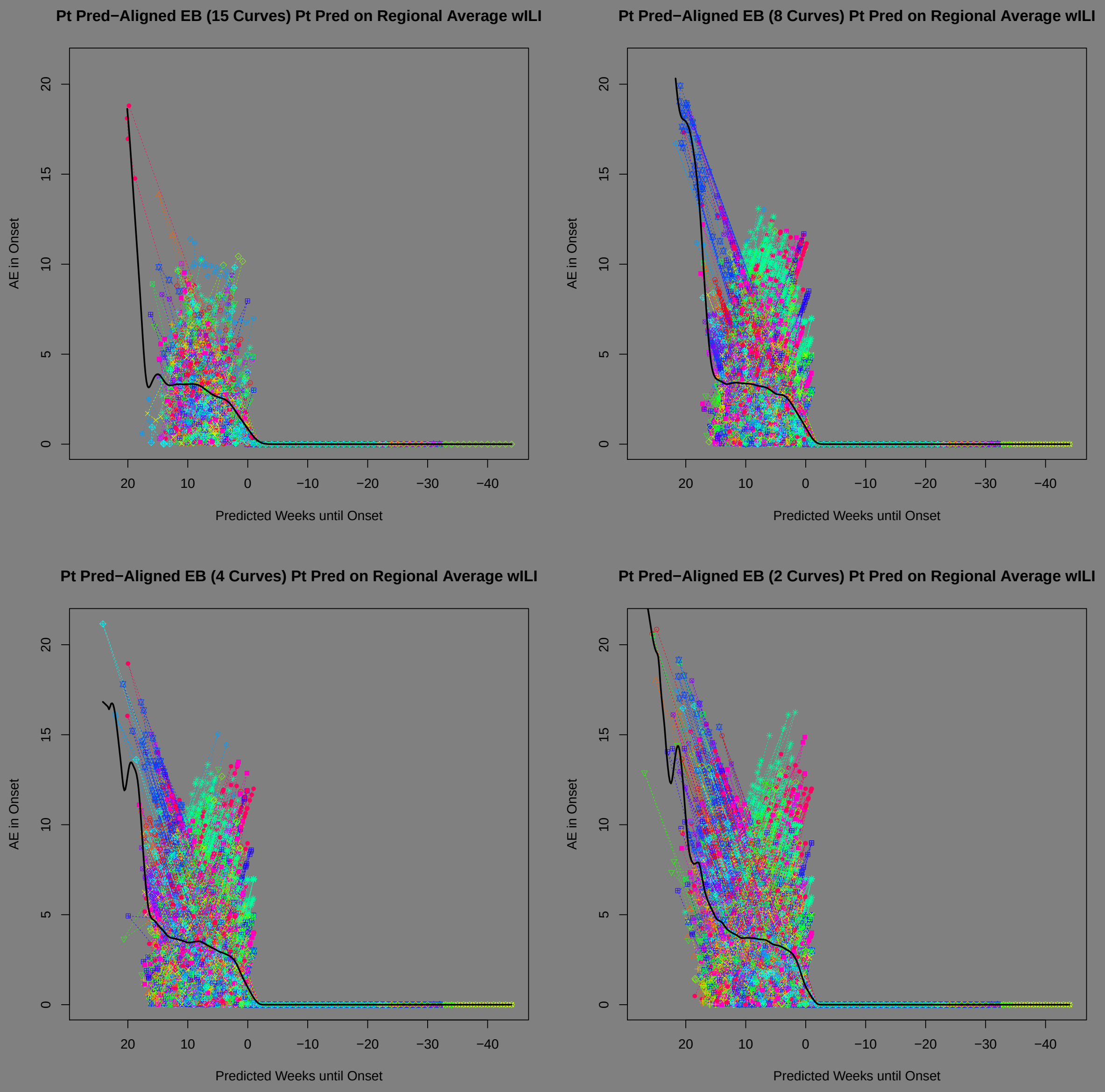

Cross-validation study of effects of using less seasons to form prior.

(C) The point prediction error distribution and mean against predicted onset and peak week.

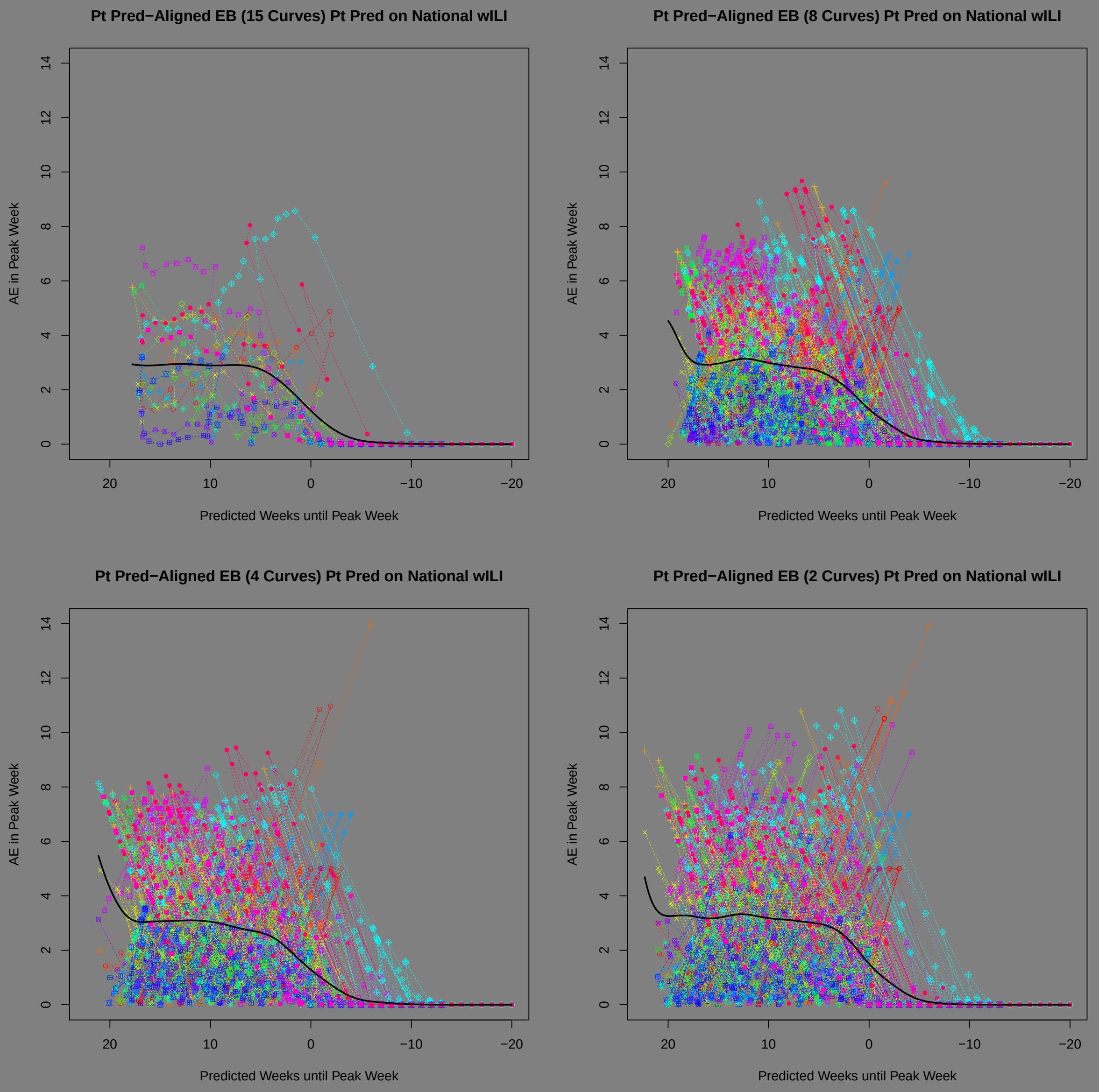

Cross-validation study of effects of using less seasons to form prior.

(C) The point prediction error distribution and mean against predicted onset and peak week.

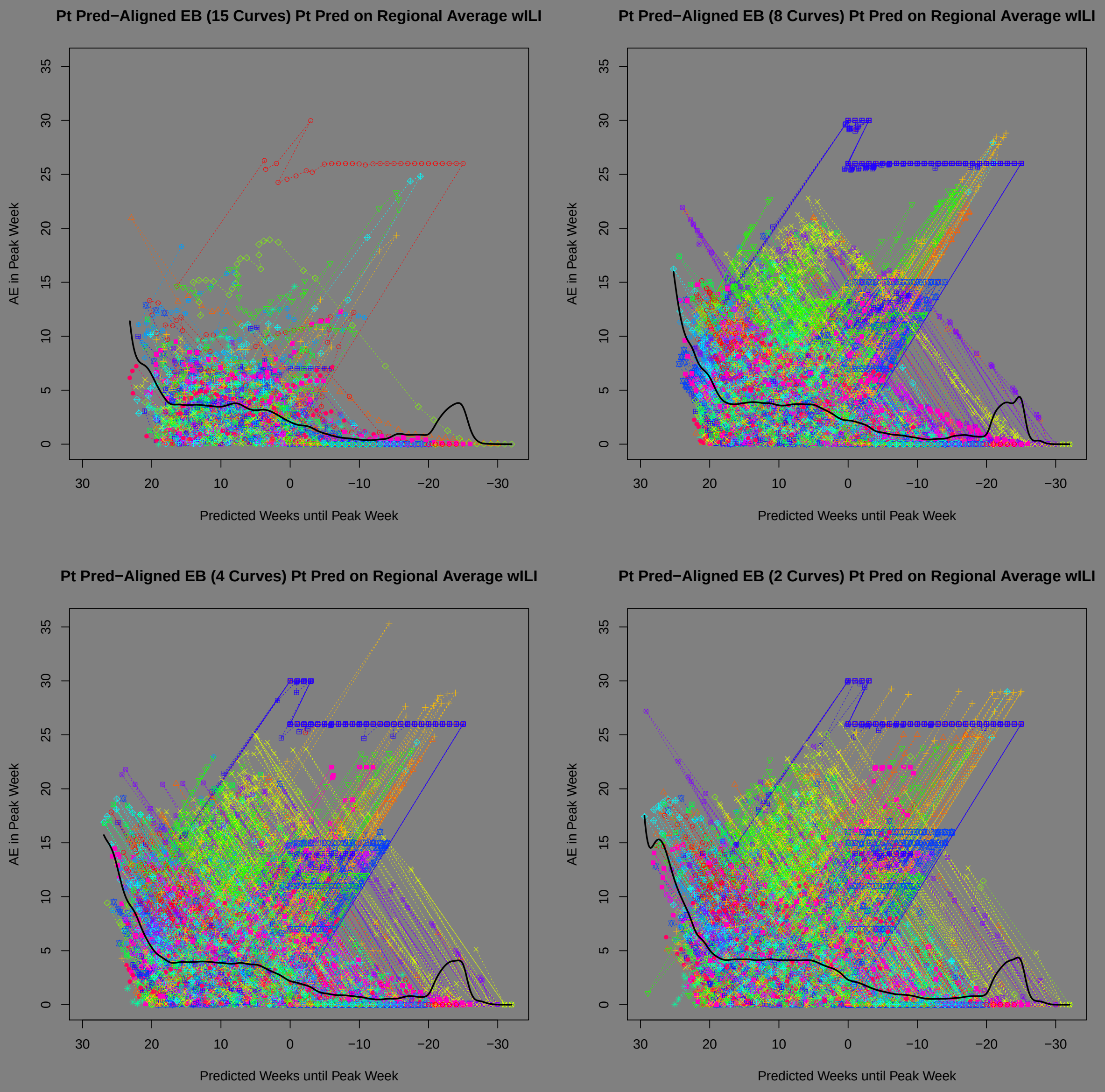

Cross-validation study of effects of using less seasons to form prior.

(C) The point prediction error distribution and mean against predicted onset and peak week.

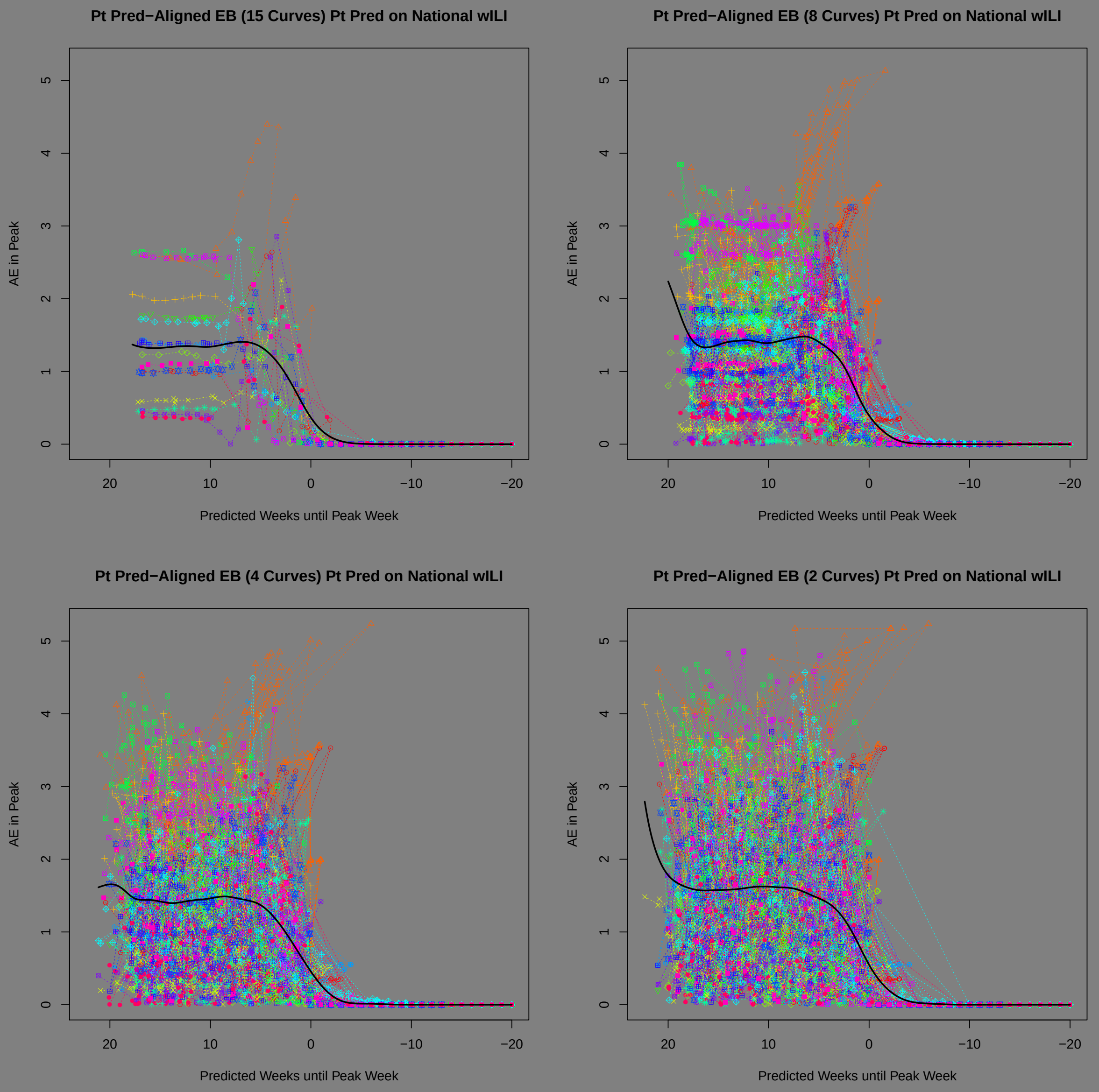

Cross-validation study of effects of using less seasons to form prior.

(C) The point prediction error distribution and mean against predicted onset and peak week.

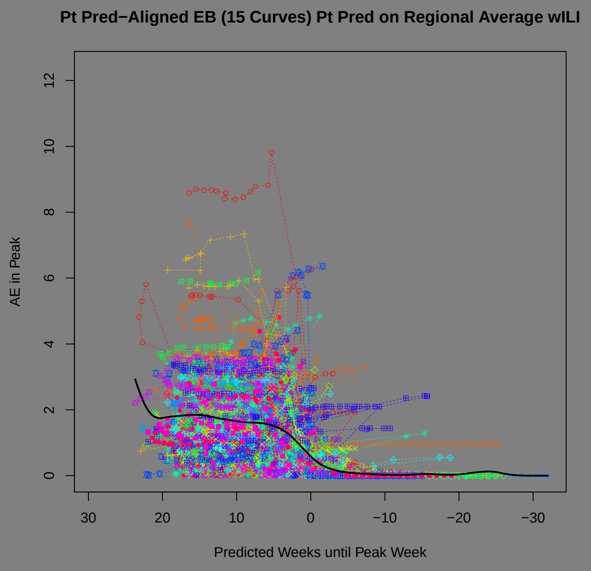

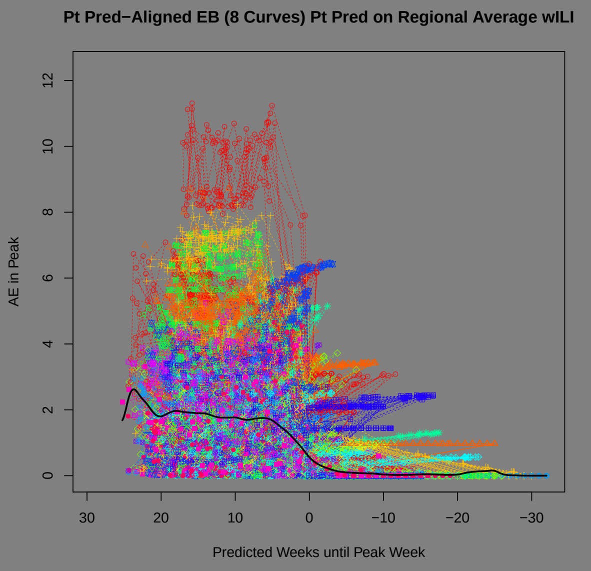

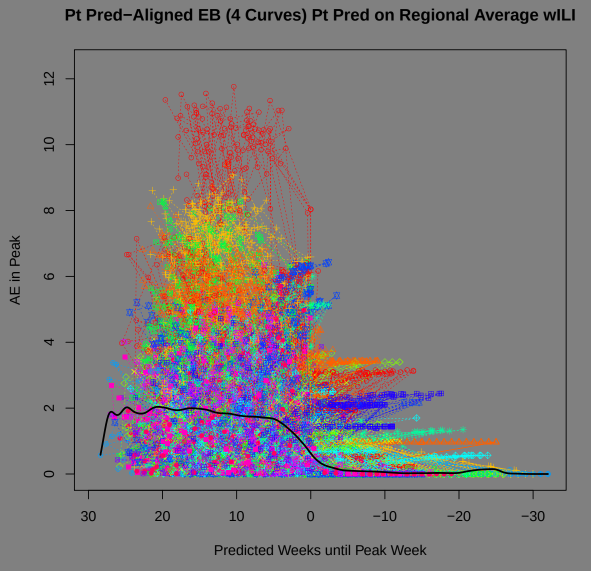

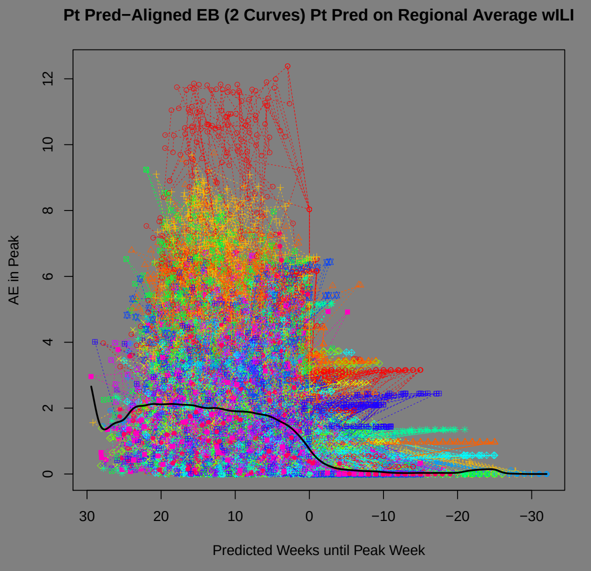

Cross-validation study of effects of using less seasons to form prior.

(C) The point prediction error distribution and mean against predicted onset and peak week.

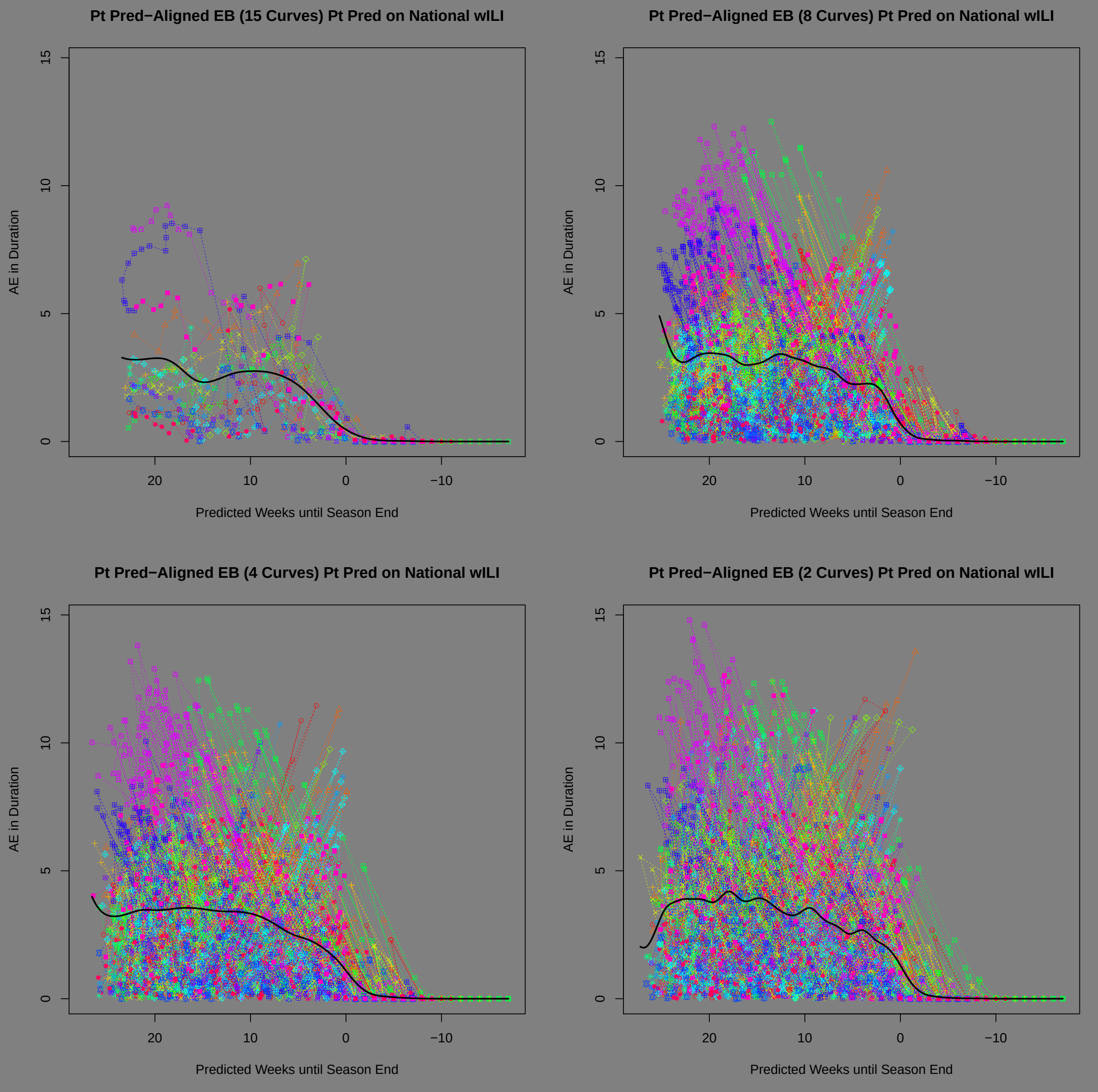

Cross-validation study of effects of using less seasons to form prior.

(C) The point prediction error distribution and mean against predicted onset and peak week.

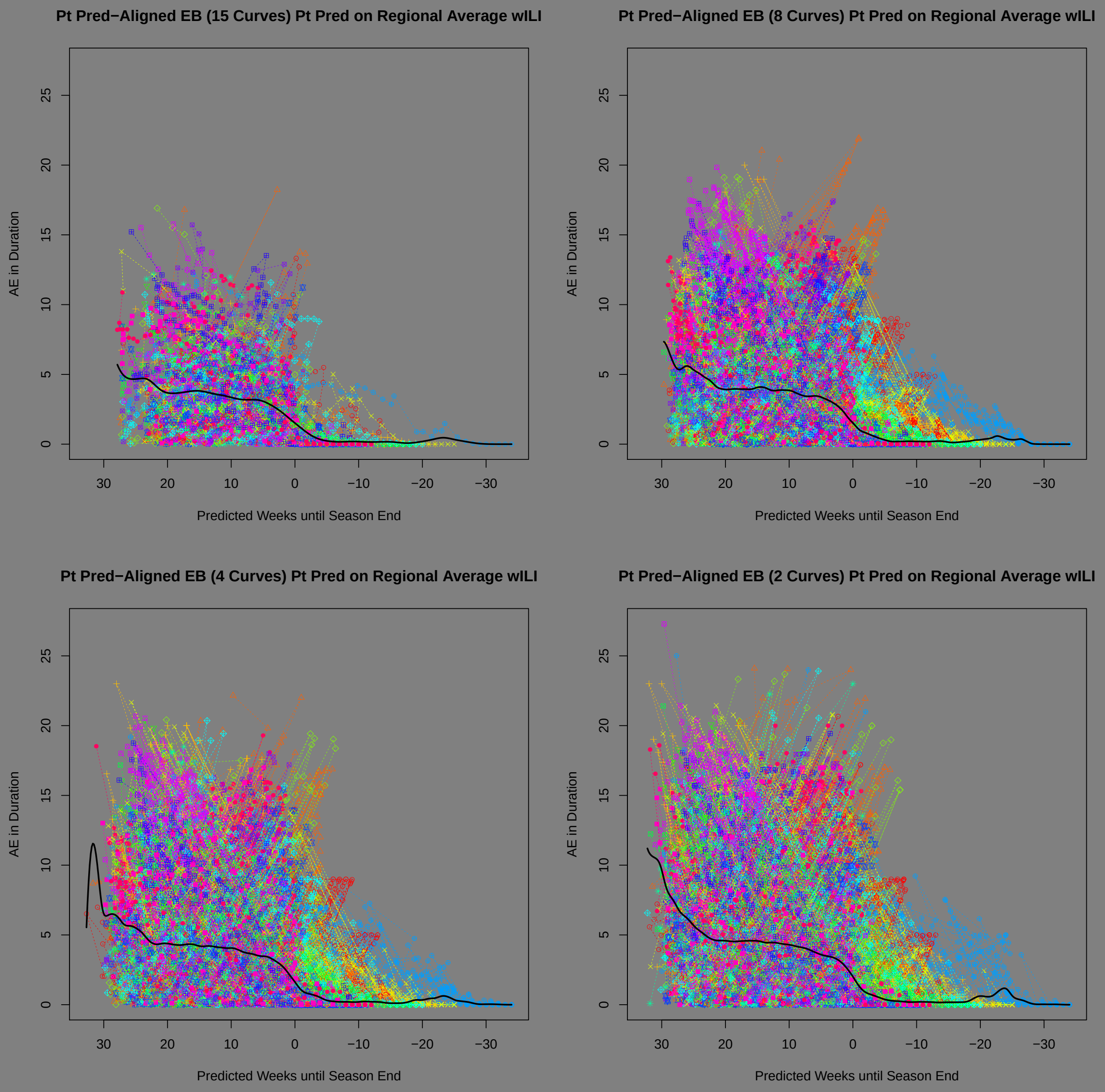

Cross-validation study of effects of using less seasons to form prior.

(D) The probability of point predictions being within certain thresholds of the observed values against predicted onset and peak week.

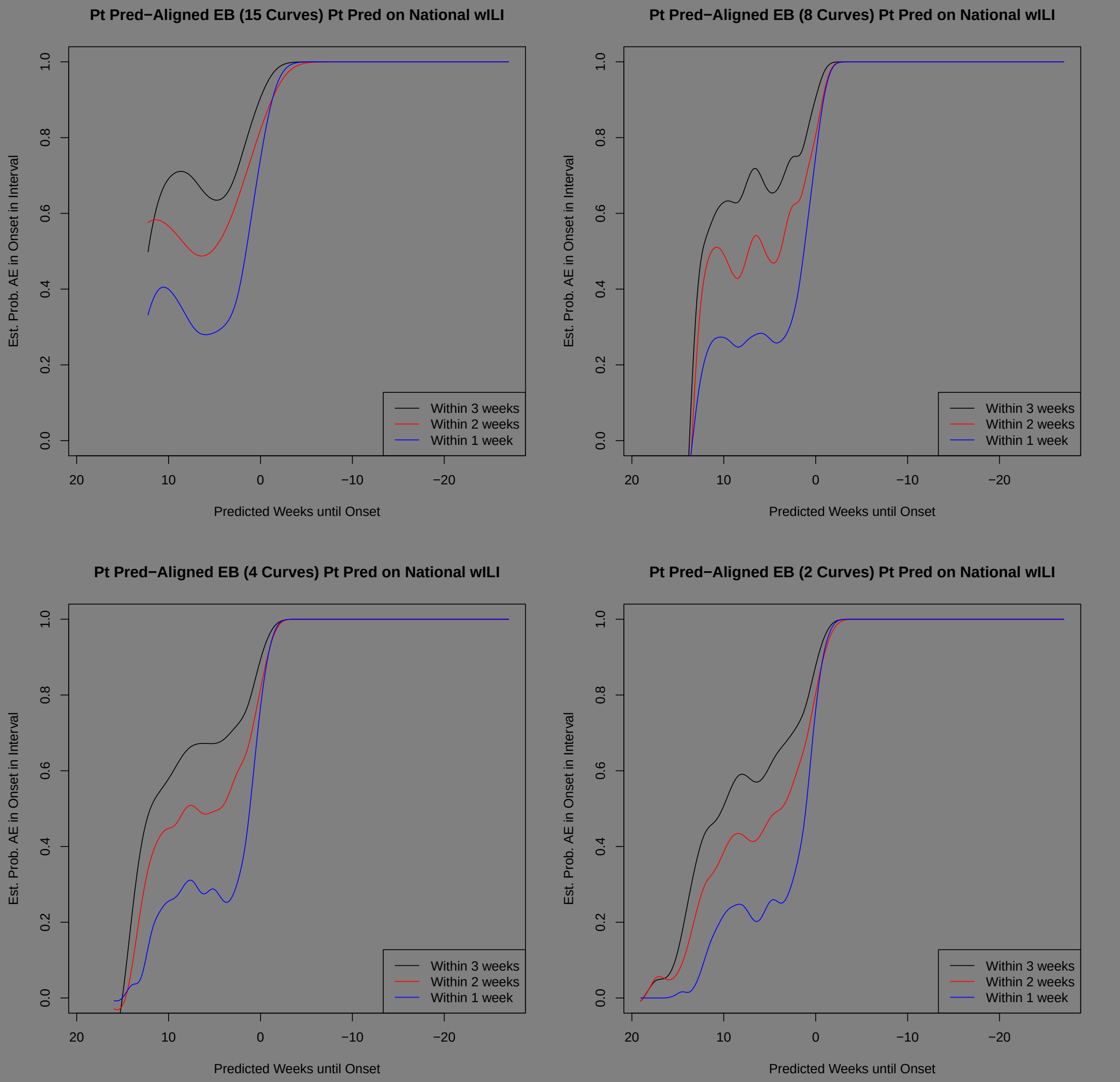

Cross-validation study of effects of using less seasons to form prior.

(D) The probability of point predictions being within certain thresholds of the observed values against predicted onset and peak week.

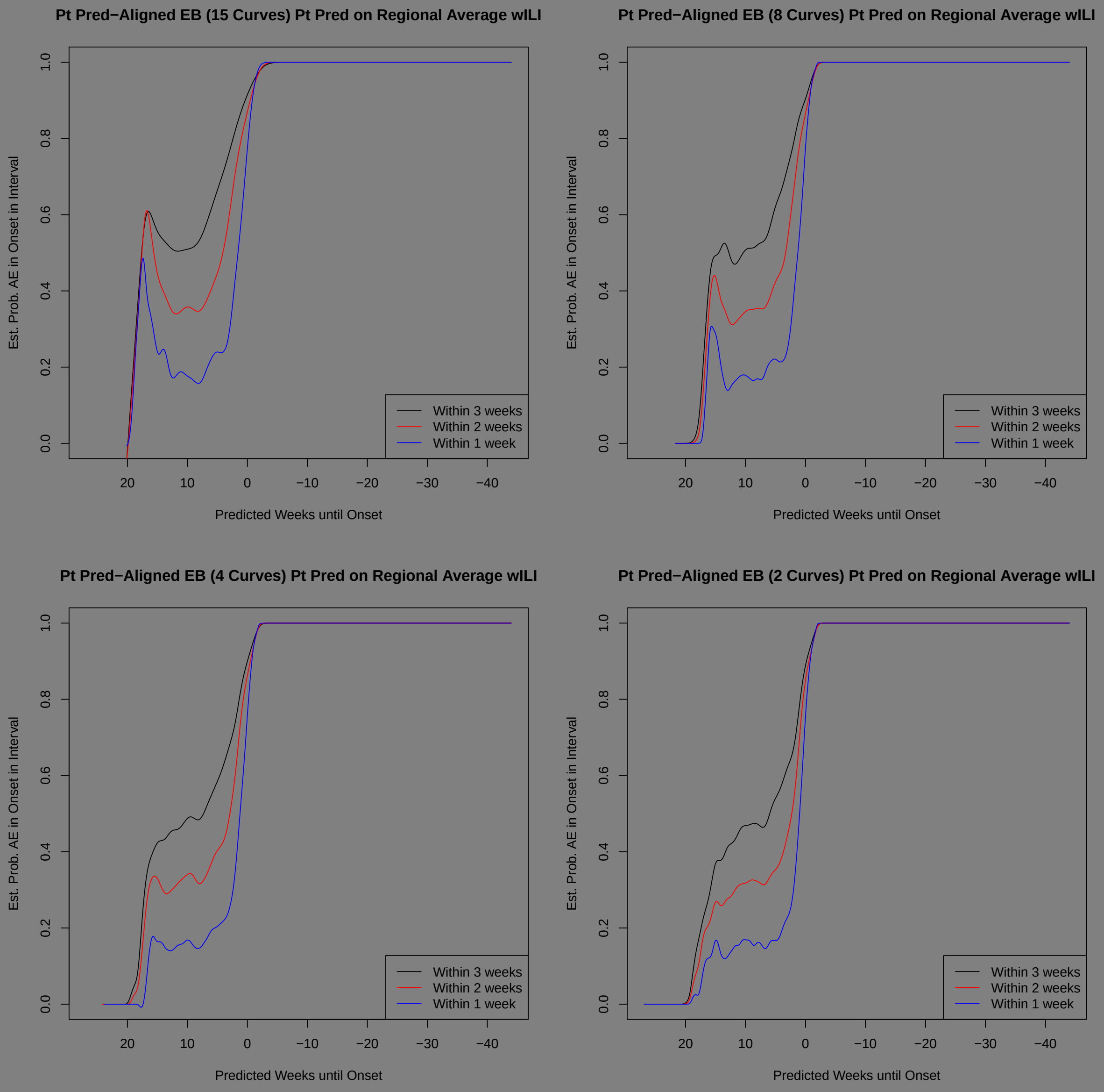

Cross-validation study of effects of using less seasons to form prior.

(D) The probability of point predictions being within certain thresholds of the observed values against predicted onset and peak week.

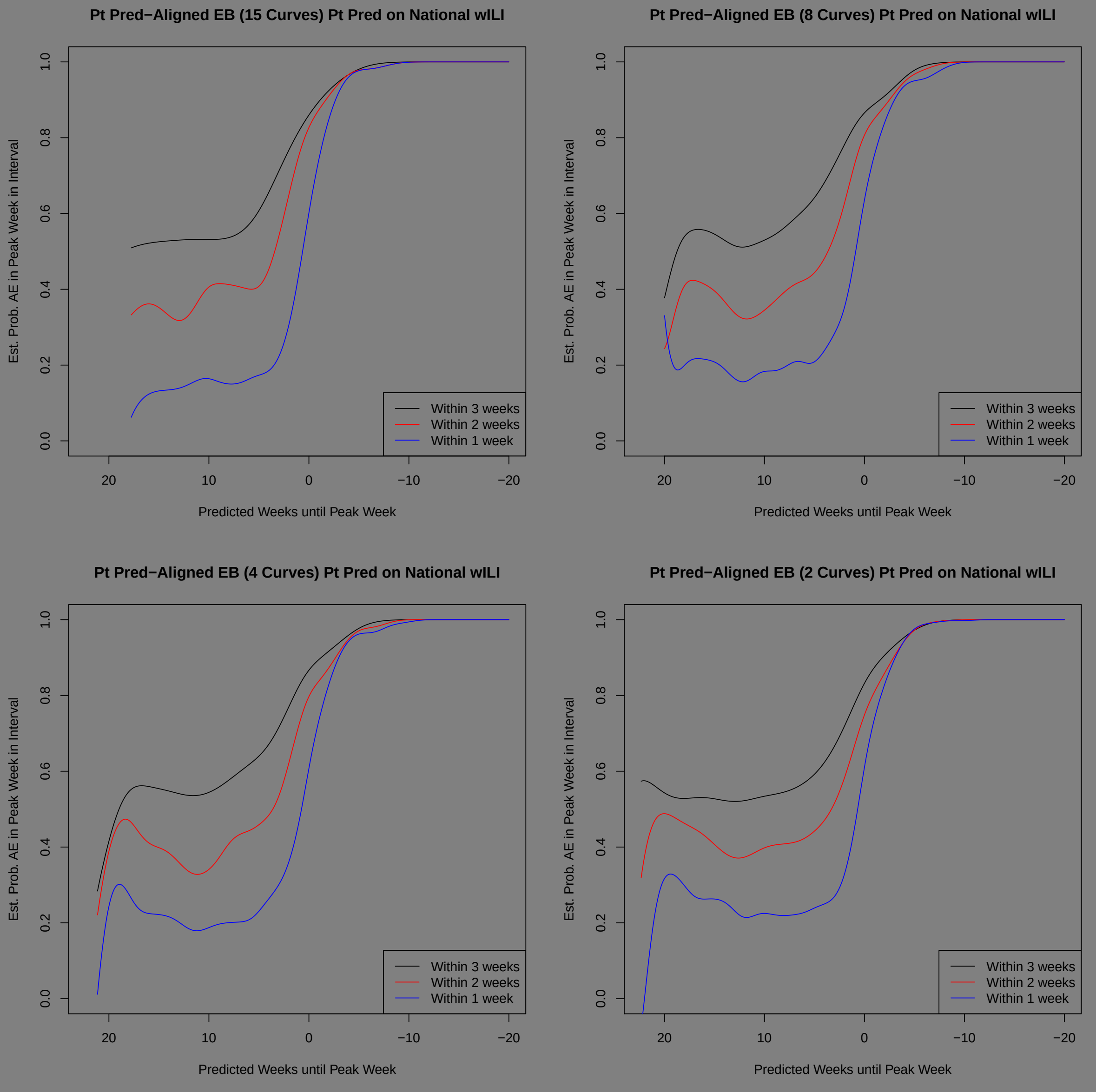

Cross-validation study of effects of using less seasons to form prior.

(D) The probability of point predictions being within certain thresholds of the observed values against predicted onset and peak week.

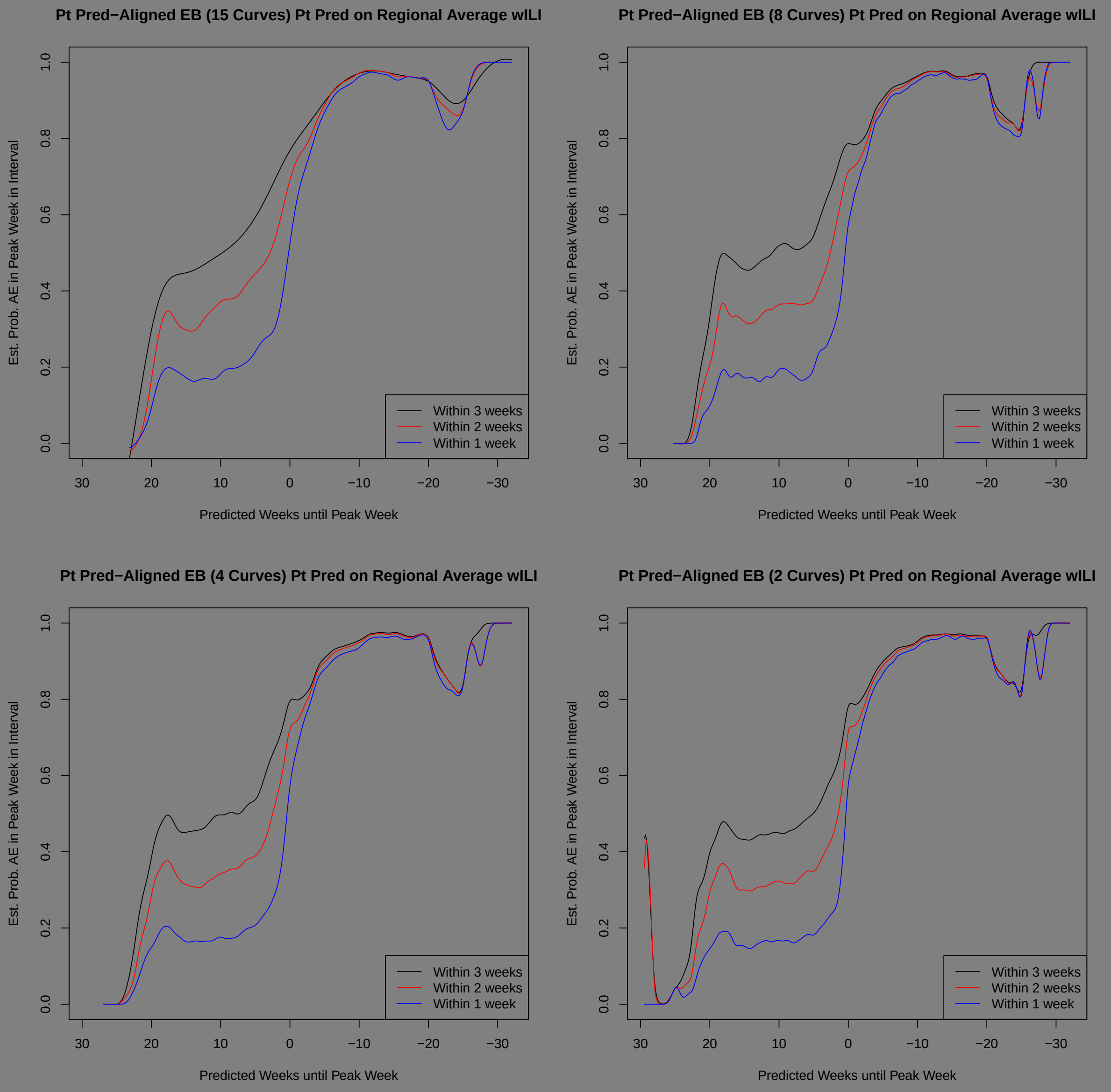

Cross-validation study of effects of using less seasons to form prior.

(D) The probability of point predictions being within certain thresholds of the observed values against predicted onset and peak week.

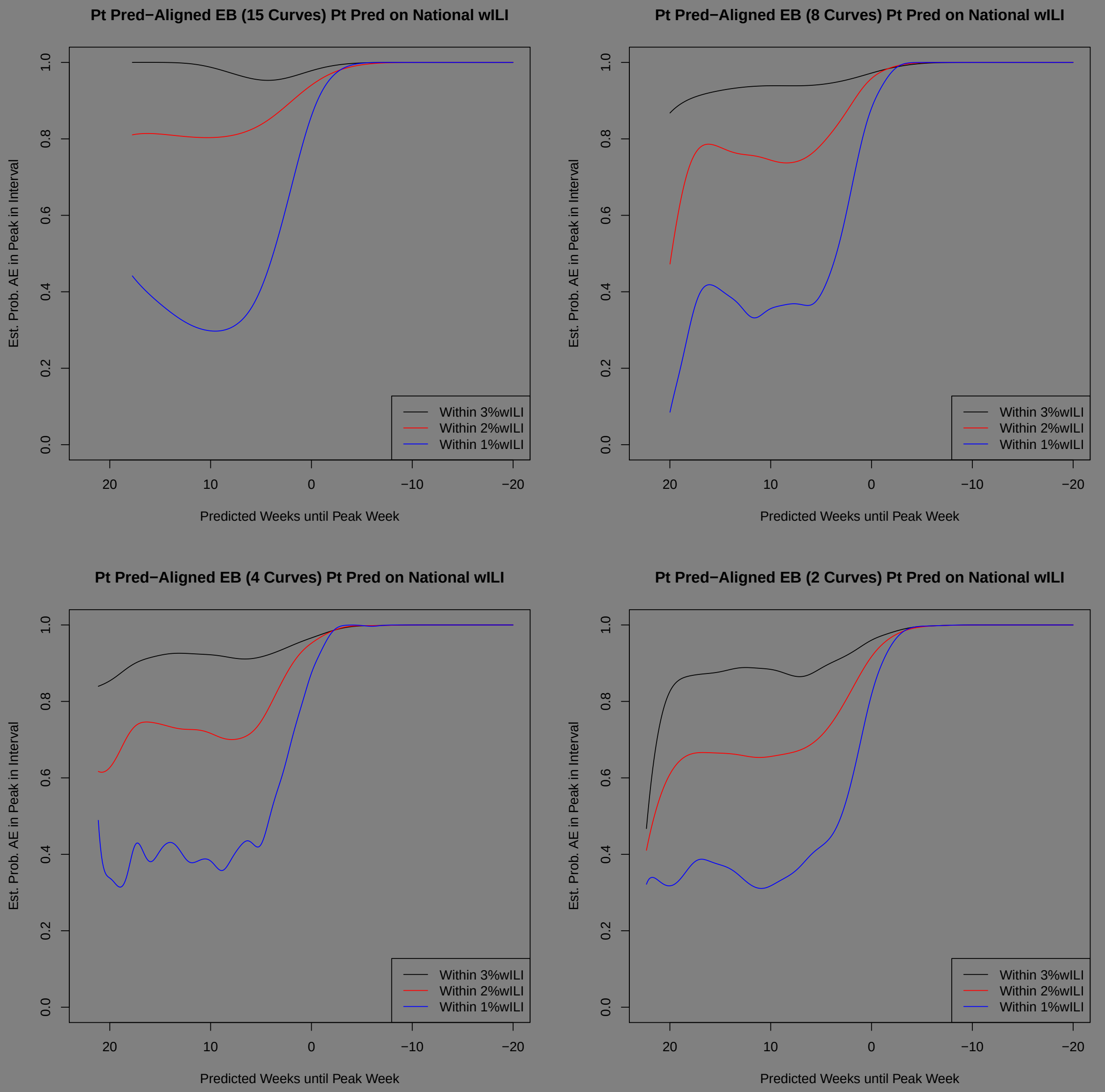

Cross-validation study of effects of using less seasons to form prior.

(D) The probability of point predictions being within certain thresholds of the observed values against predicted onset and peak week.

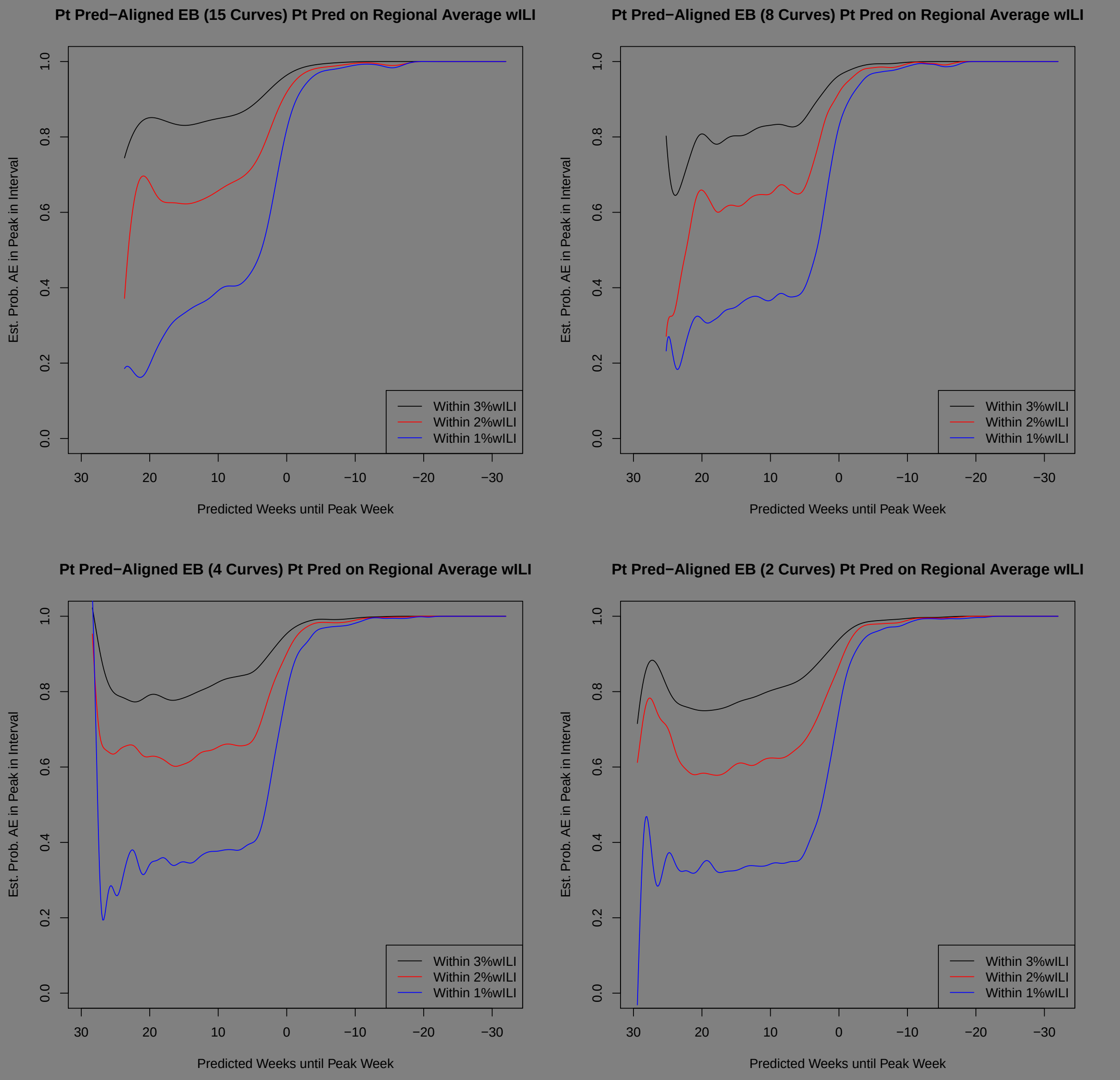

Cross-validation study of effects of using less seasons to form prior.

(D) The probability of point predictions being within certain thresholds of the observed values against predicted onset and peak week.

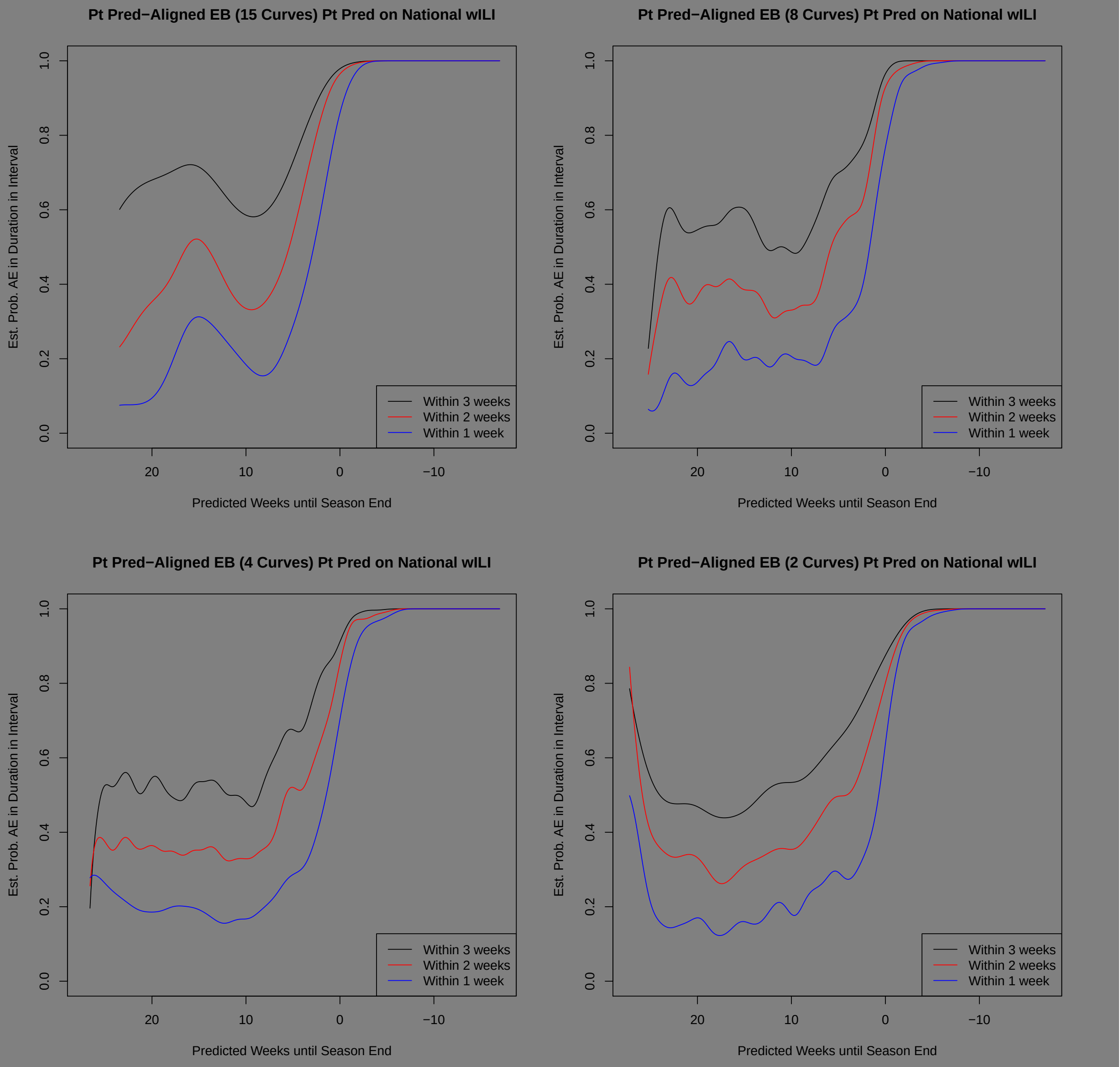

Cross-validation study of effects of using less seasons to form prior.

(D) The probability of point predictions being within certain thresholds of the observed values against predicted onset and peak week.

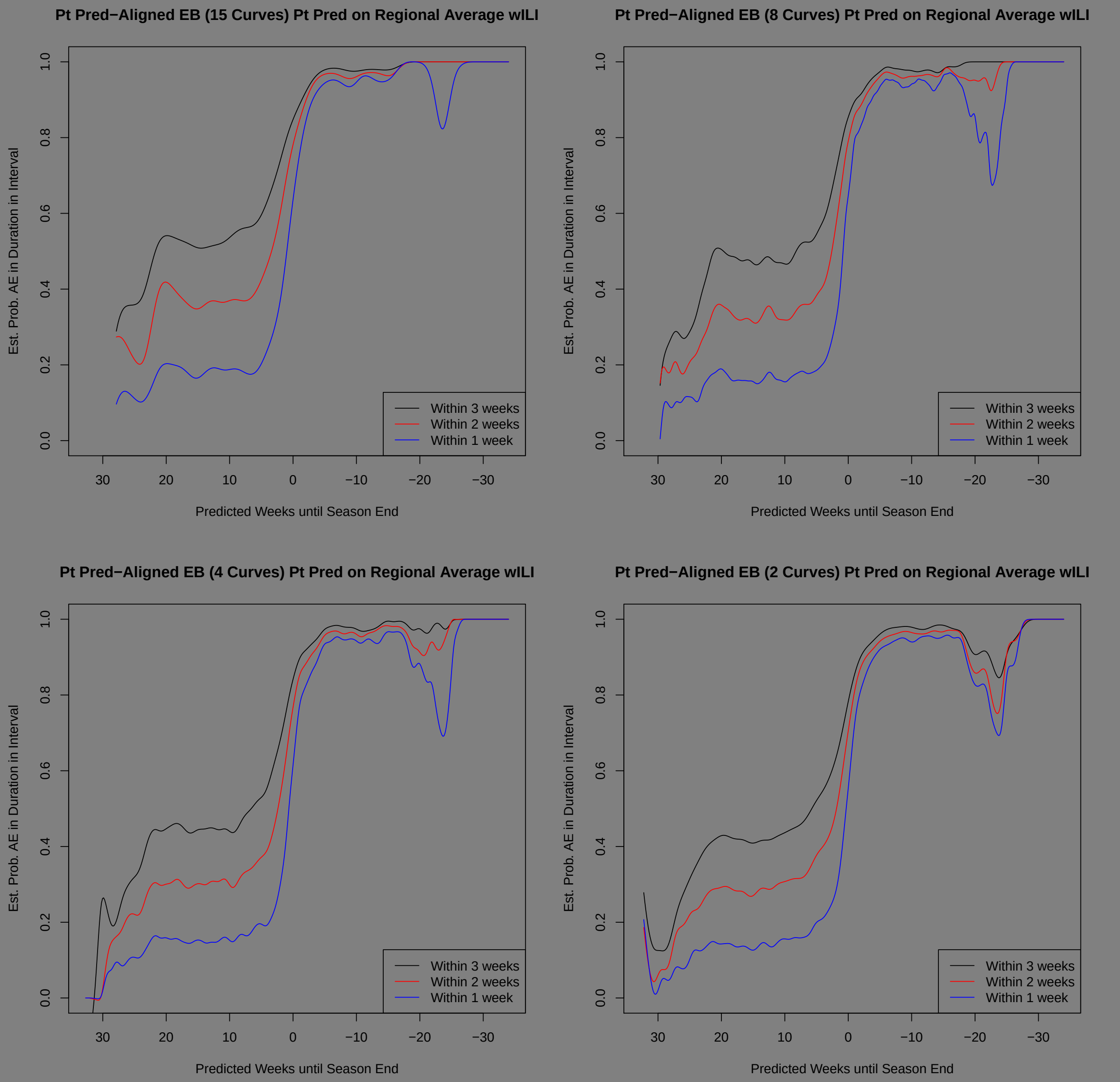

Supplement: S4 Fig — We applied the EB framework using randomly selected subsets of the available seasons to see how many seasons are needed for passable forecast results, and how prediction accuracy improves with additional data. This figure contains plots of (A) the average point prediction error against epidemic weeks aligned by the observed onset and peak week, (B) the coverage of 90% credible intervals against epidemic weeks aligned by the observed onset and peak week, (C) the point prediction error distribution and mean against predicted onset and peak week, and (D) the probability of point predictions being within certain thresholds of the observed values against predicted onset and peak week, for the four forecasting targets, both nationally and regionally, for EB using 2, 4, 8, or 15 other wILI curves to form the prior. Estimation of the mean in (C) and probabilities in (D) is performed using locally linear kernel regression [49]. This series of plots was generated with a script that used GNU Parallel to generate plots in parallel on multiple processors [50]. (PDF) [file pcbi.1004382.s004.pdf]
