# Supplementary material for: Flexible Modeling of Epidemics with an Empirical Bayes Framework
Source: PLoS Comput Biol. 2015 Aug 28;11(8):e1004382. doi: 10.1371/journal.pcbi.1004382 (PMC4552841; doi:10.1371/journal.pcbi.1004382)

Curves fitted by trend filtering and SIR model

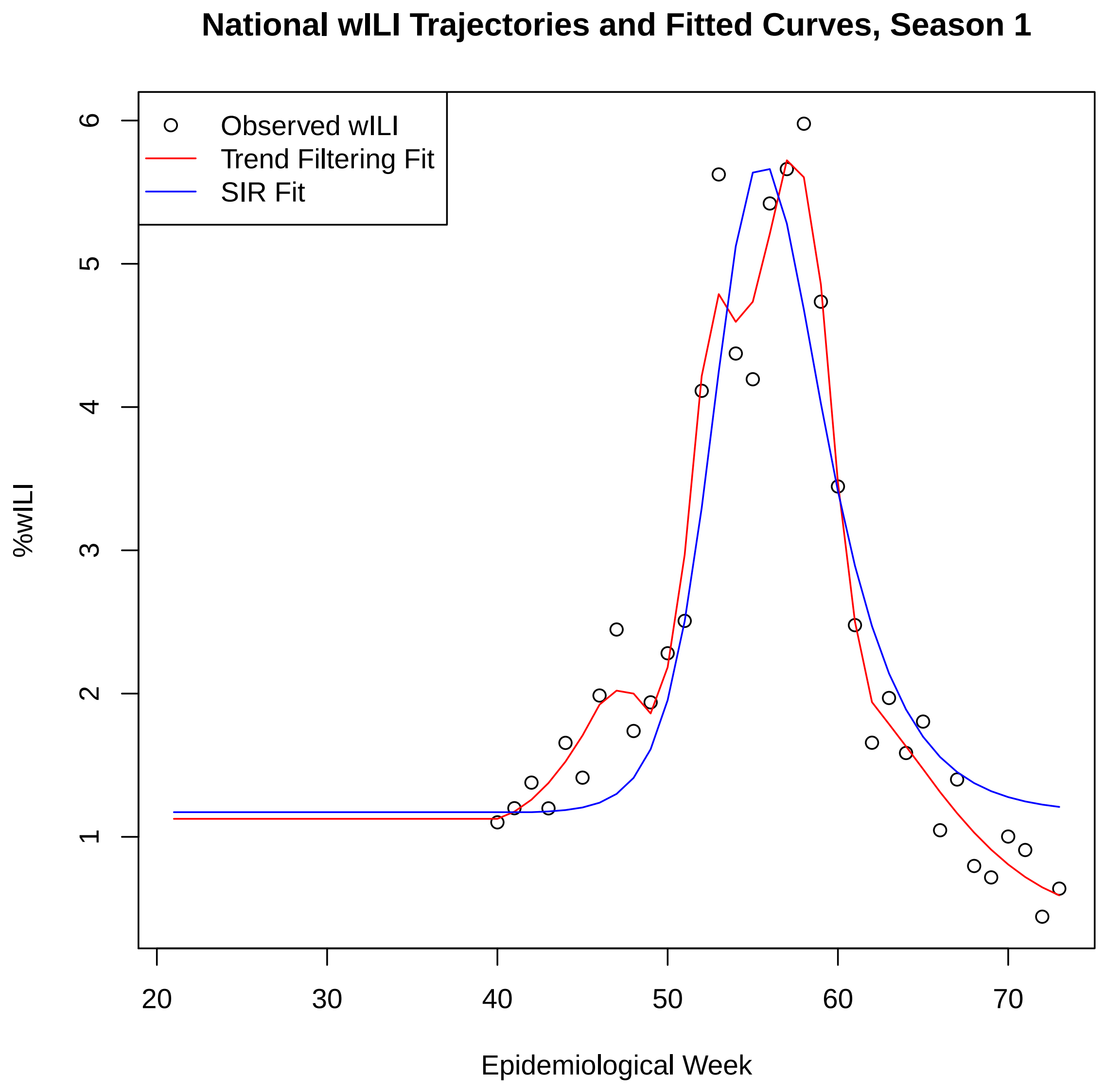

Curves fitted by trend filtering and SIR model

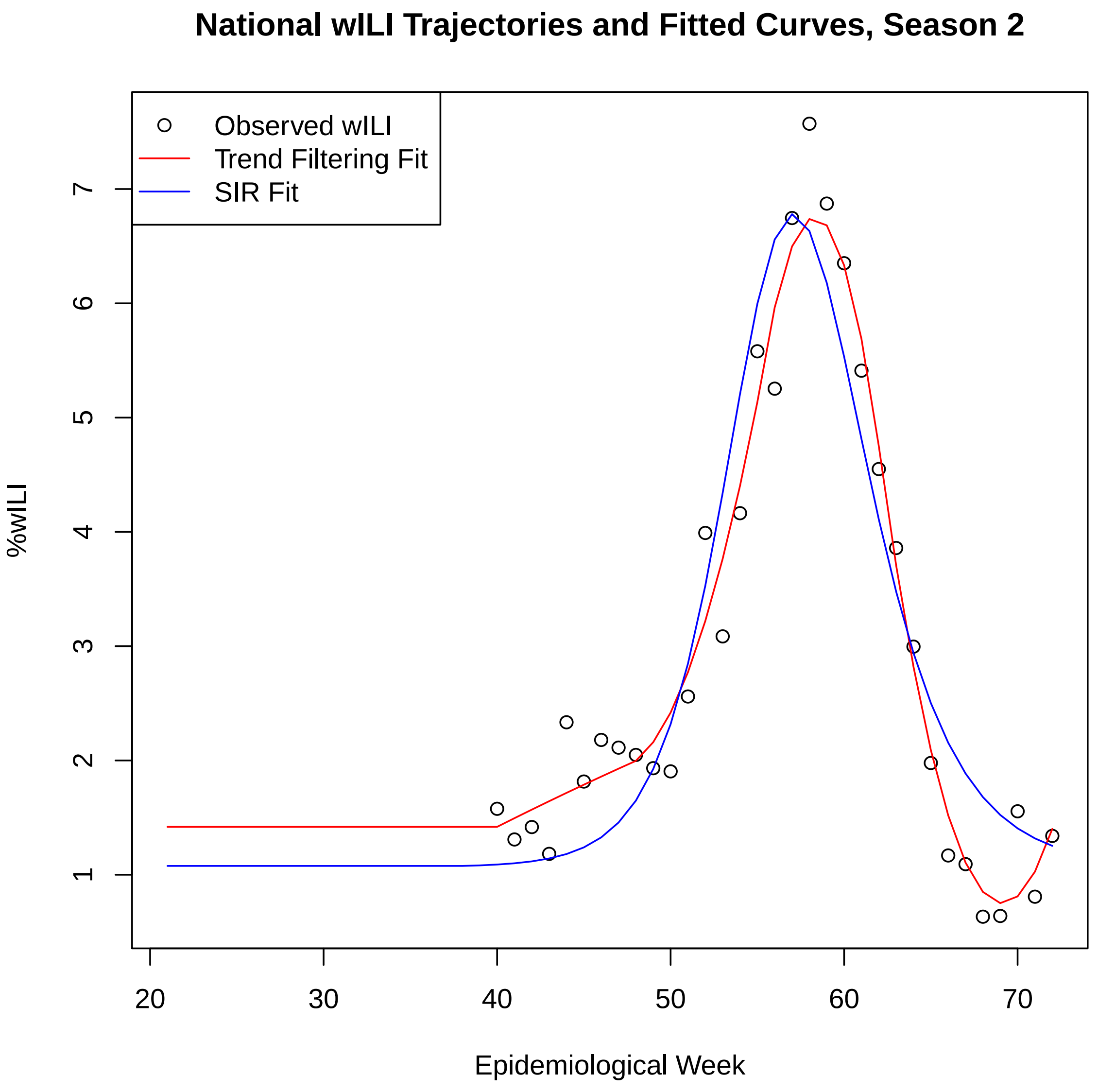

Curves fitted by trend filtering and SIR model

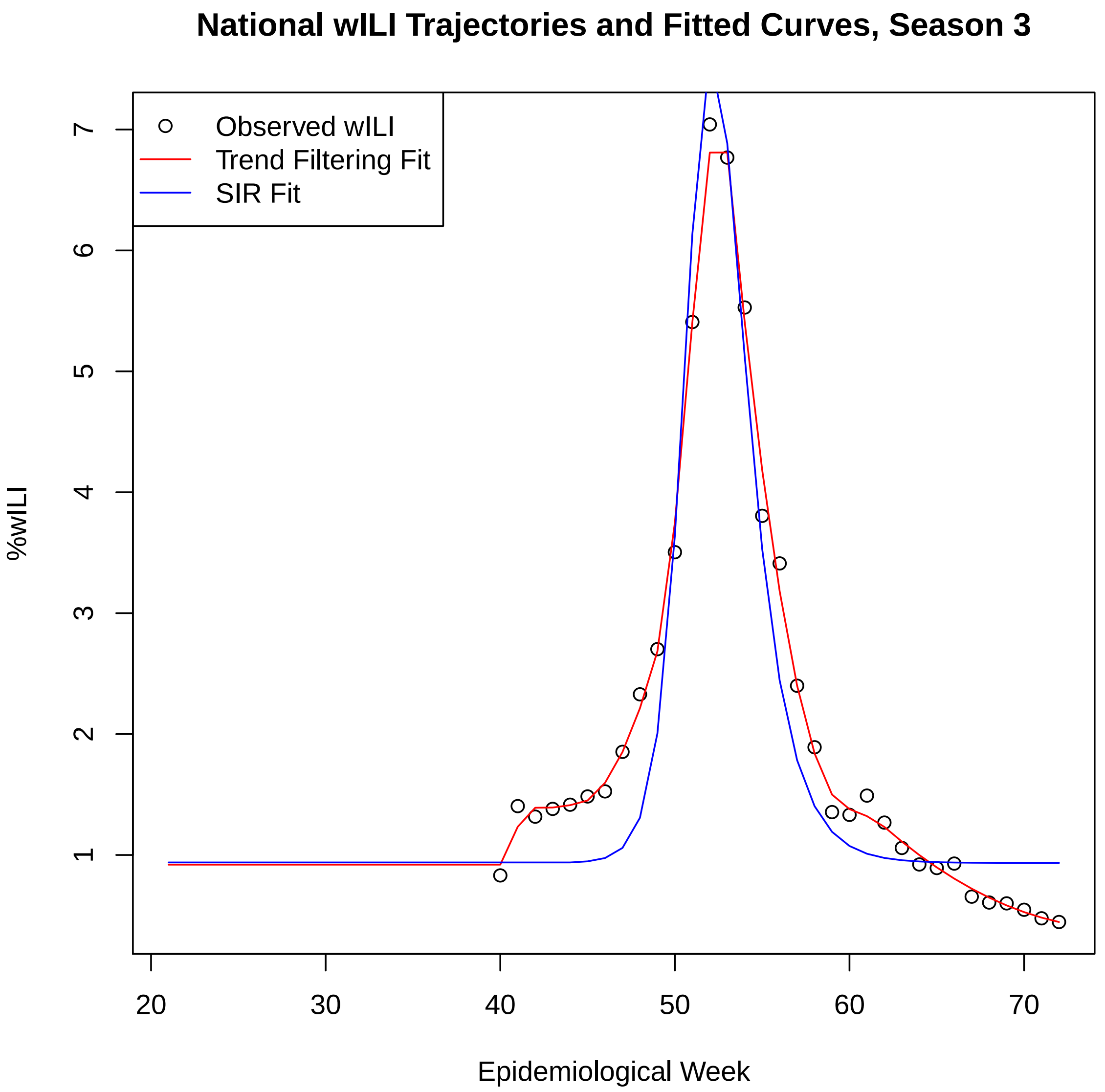

Curves fitted by trend filtering and SIR model

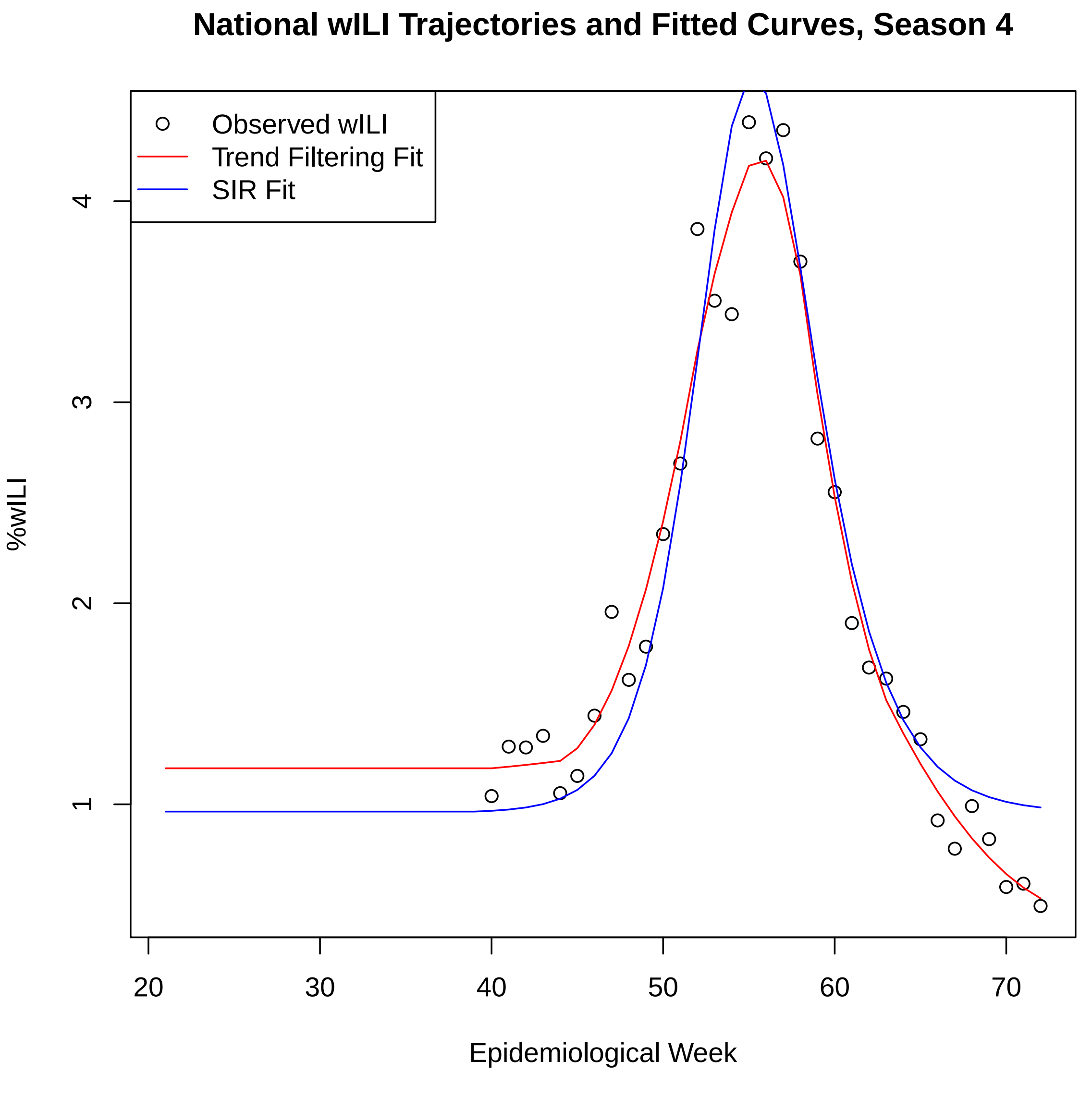

Curves fitted by trend filtering and SIR model

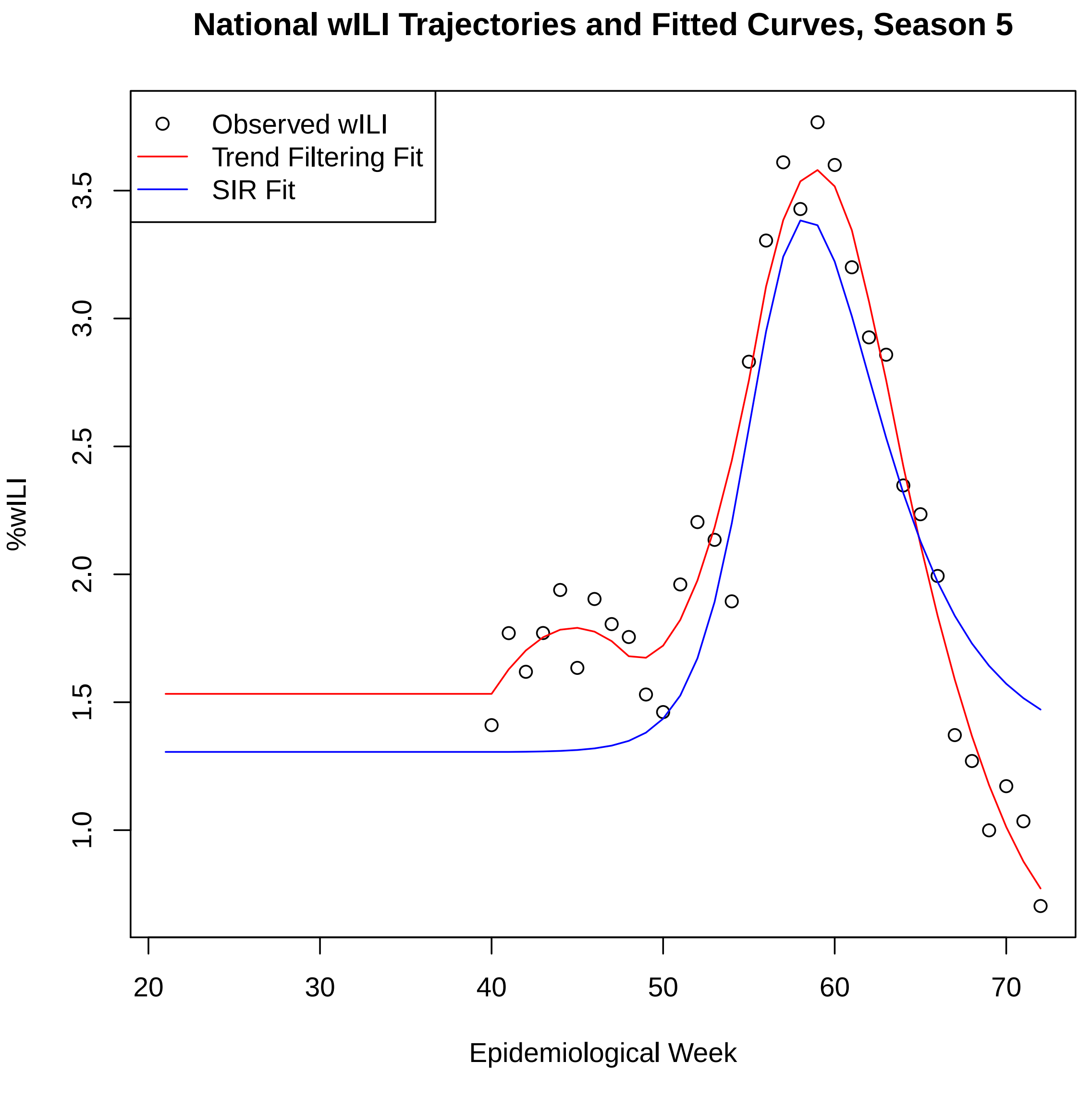

Curves fitted by trend filtering and SIR model

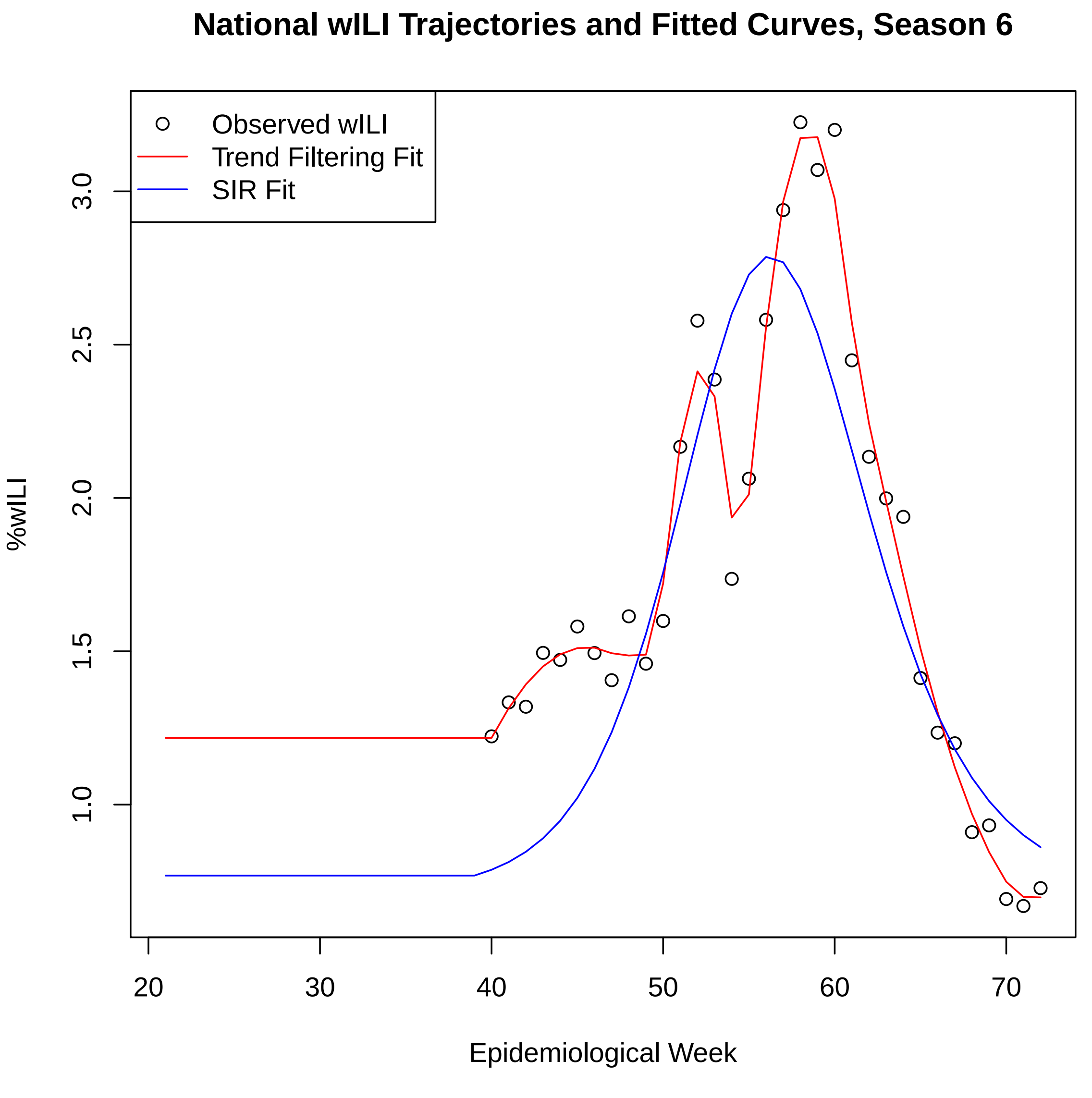

Curves fitted by trend filtering and SIR model

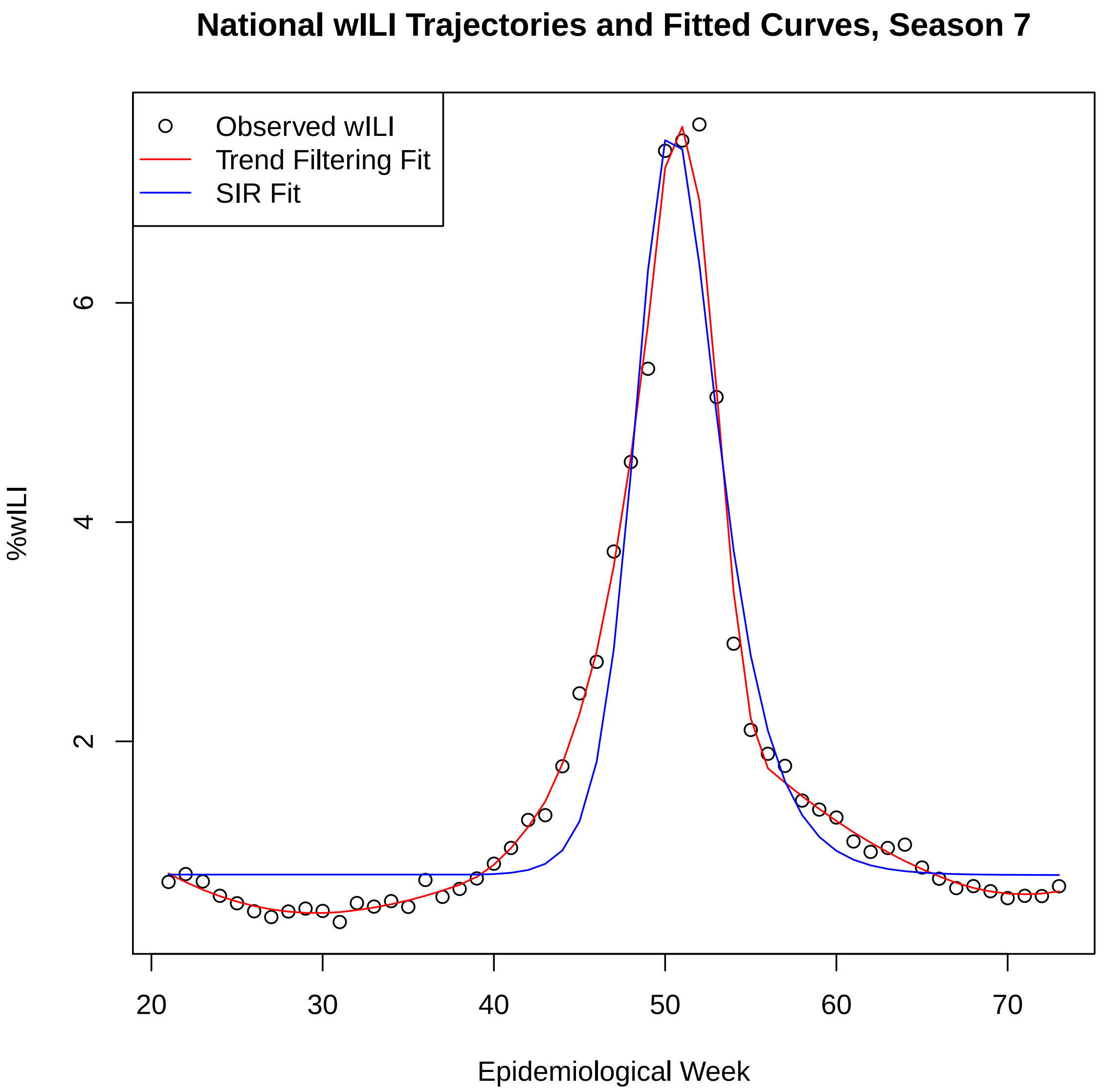

Curves fitted by trend filtering and SIR model

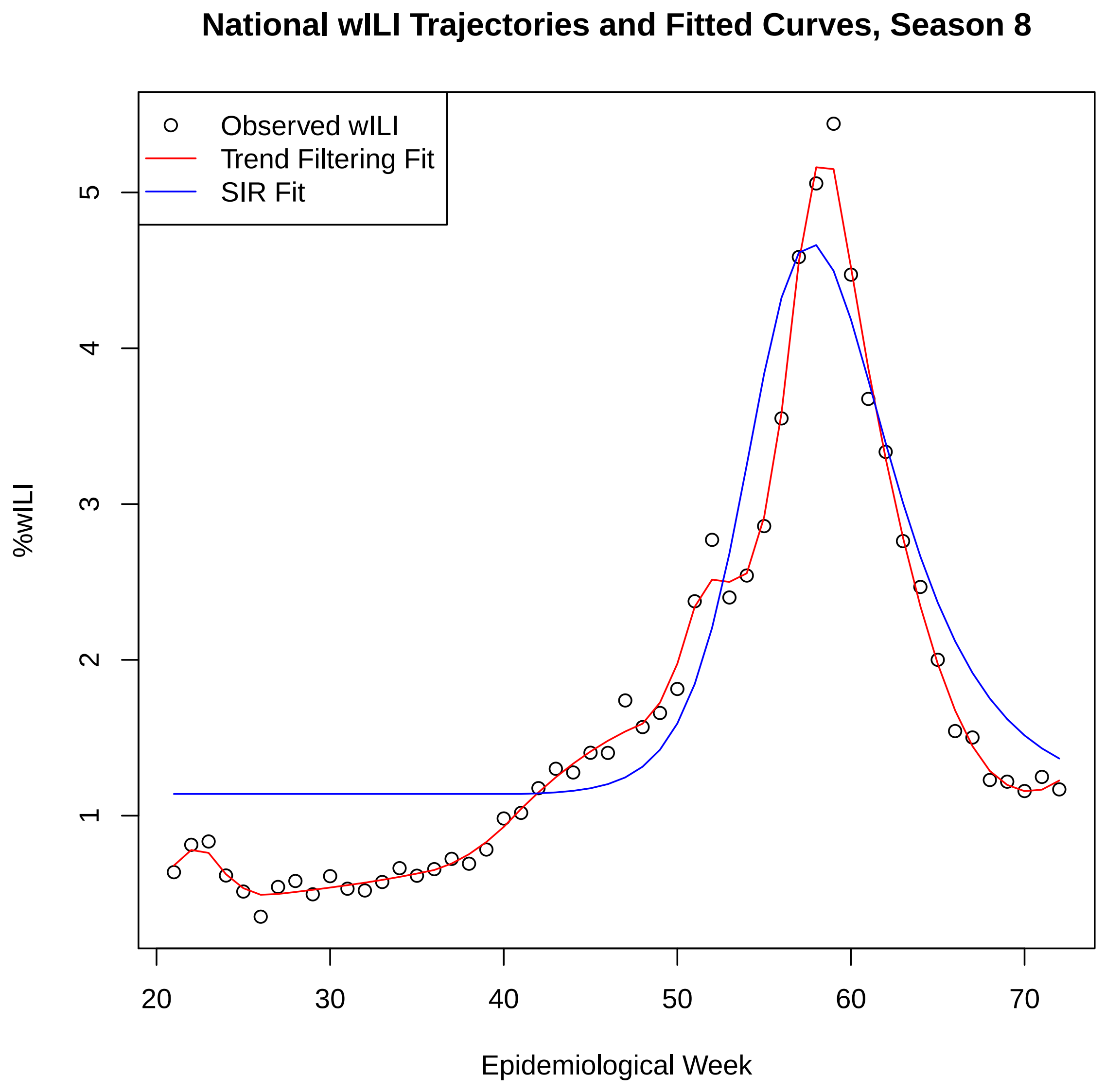

Curves fitted by trend filtering and SIR model

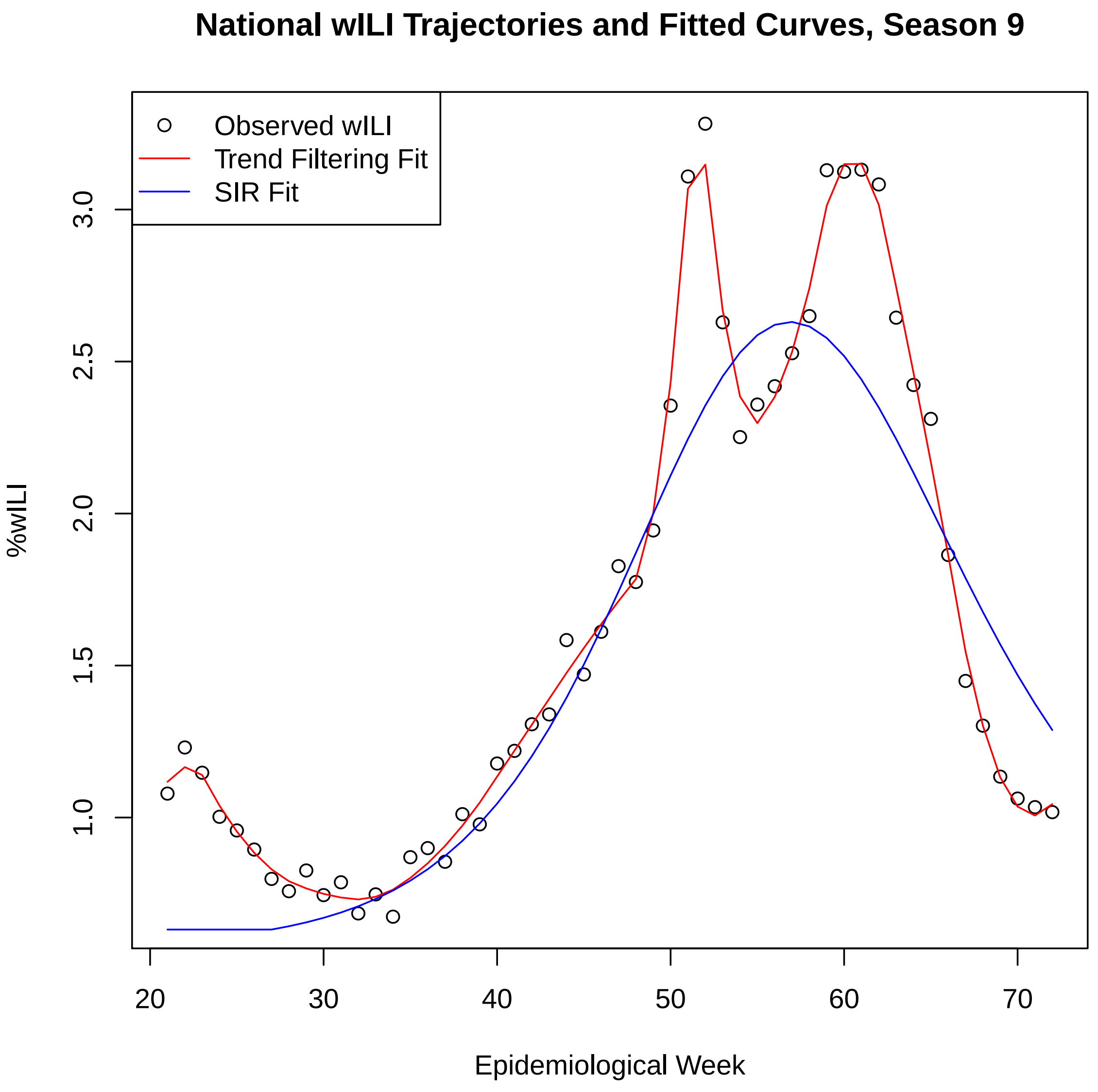

Curves fitted by trend filtering and SIR model

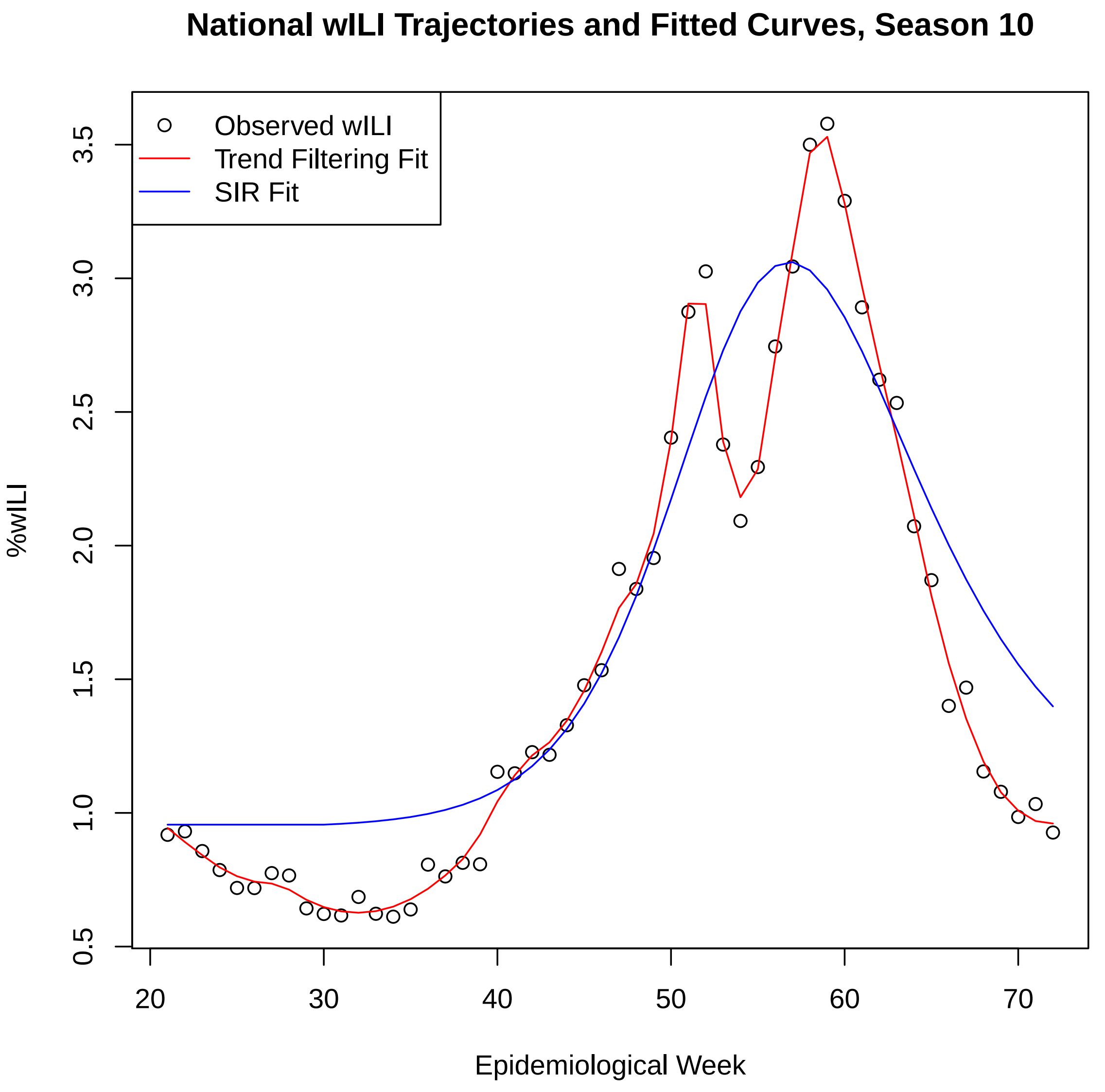

Curves fitted by trend filtering and SIR model

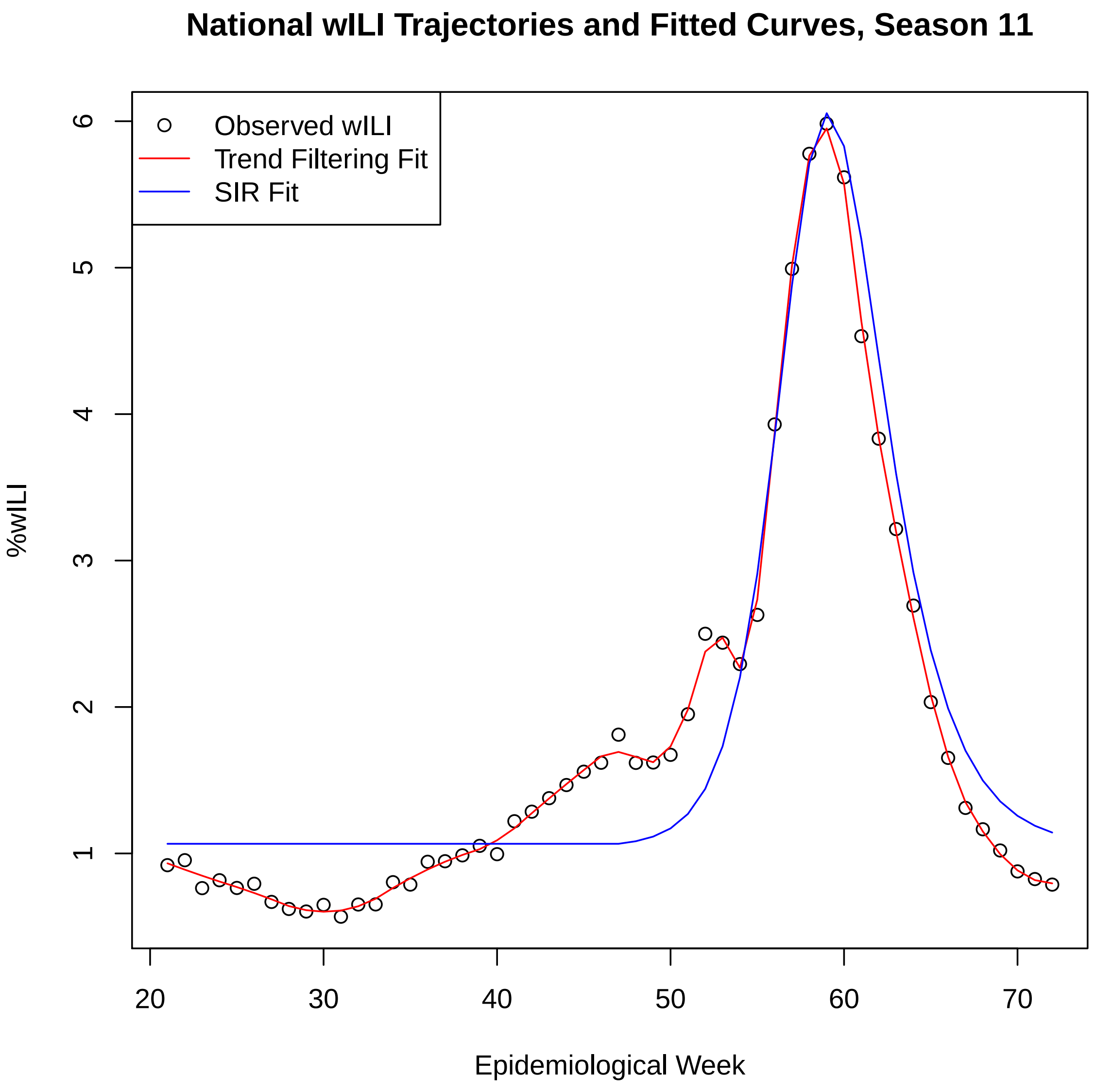

Curves fitted by trend filtering and SIR model

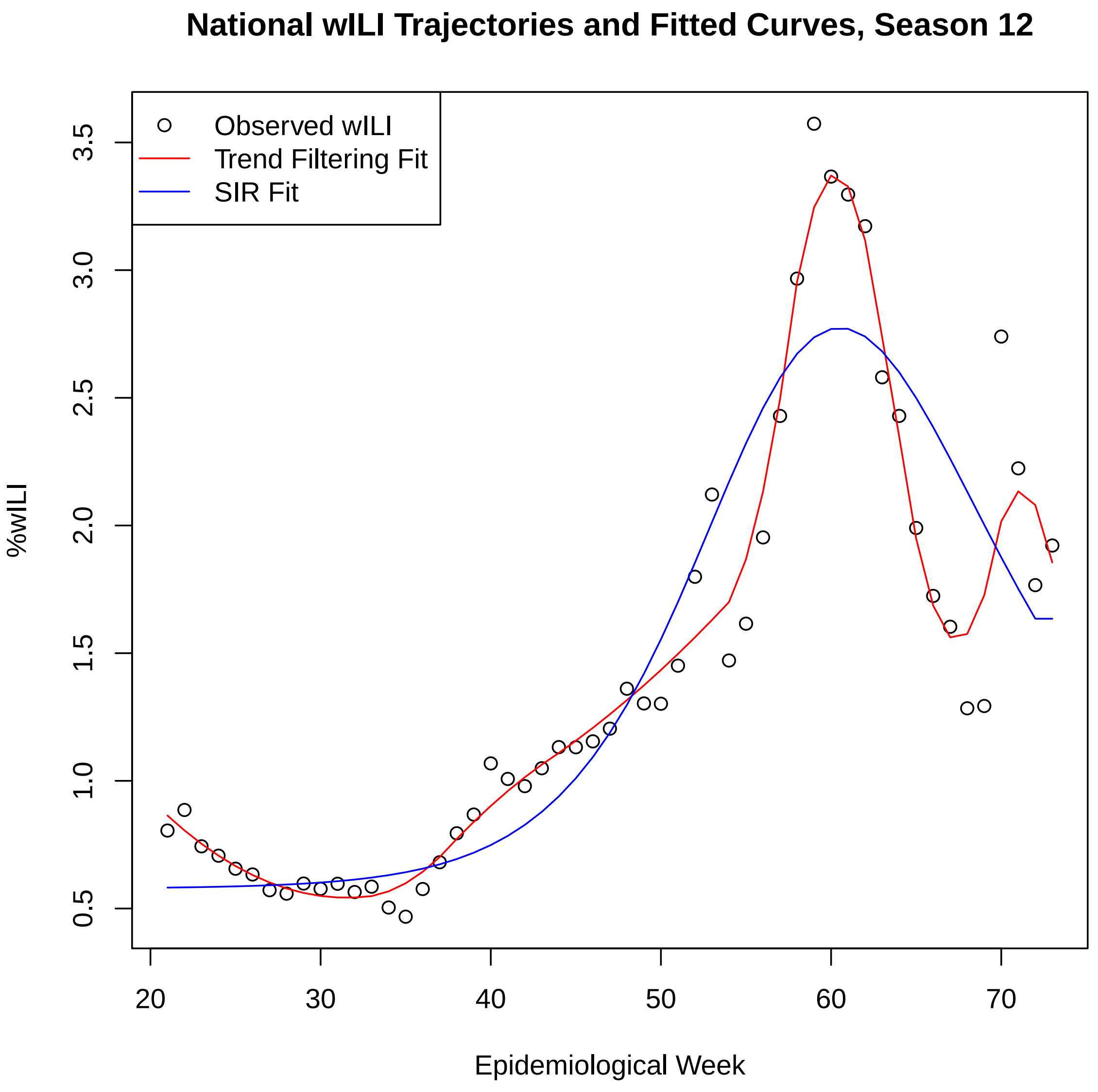

Curves fitted by trend filtering and SIR model

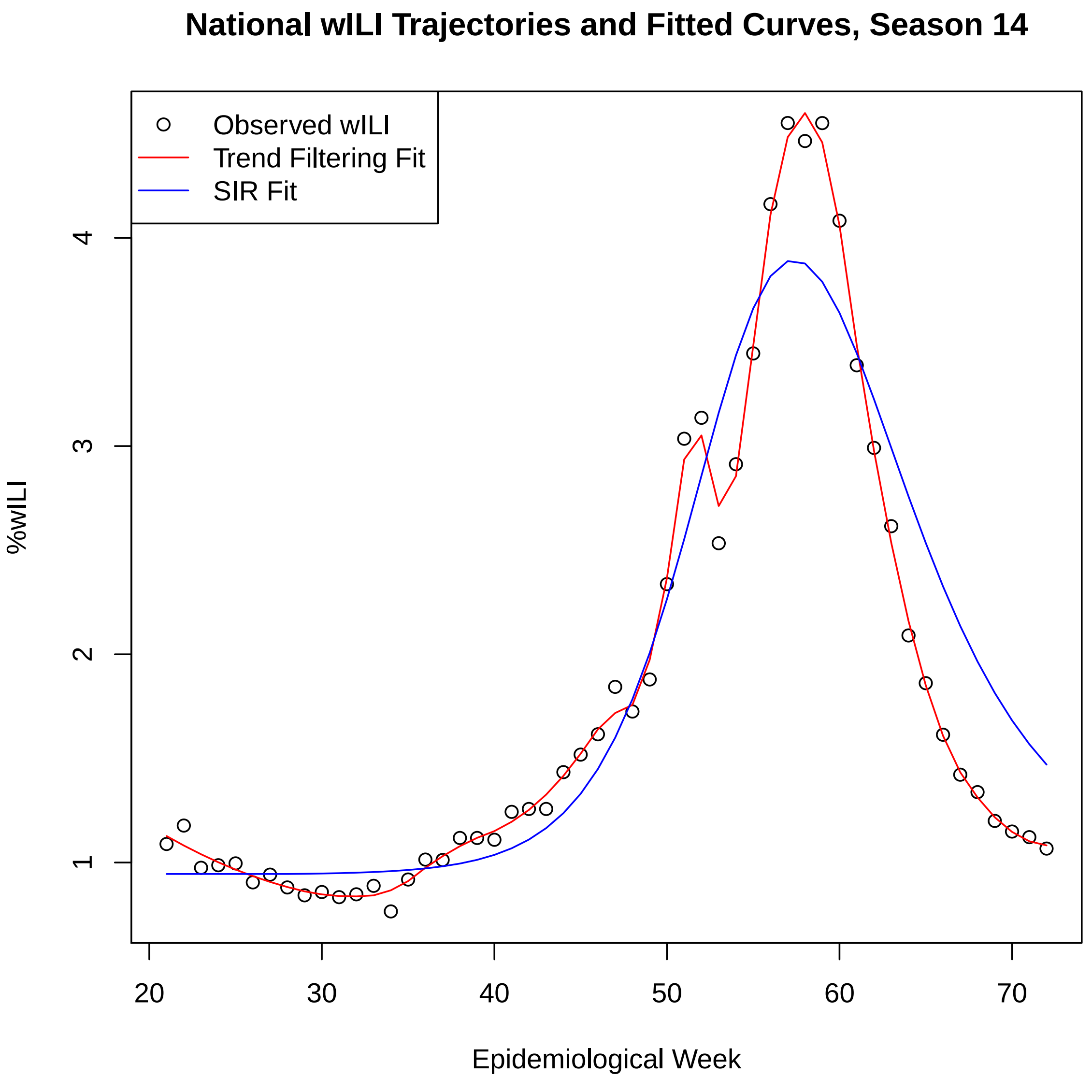

Curves fitted by trend filtering and SIR model

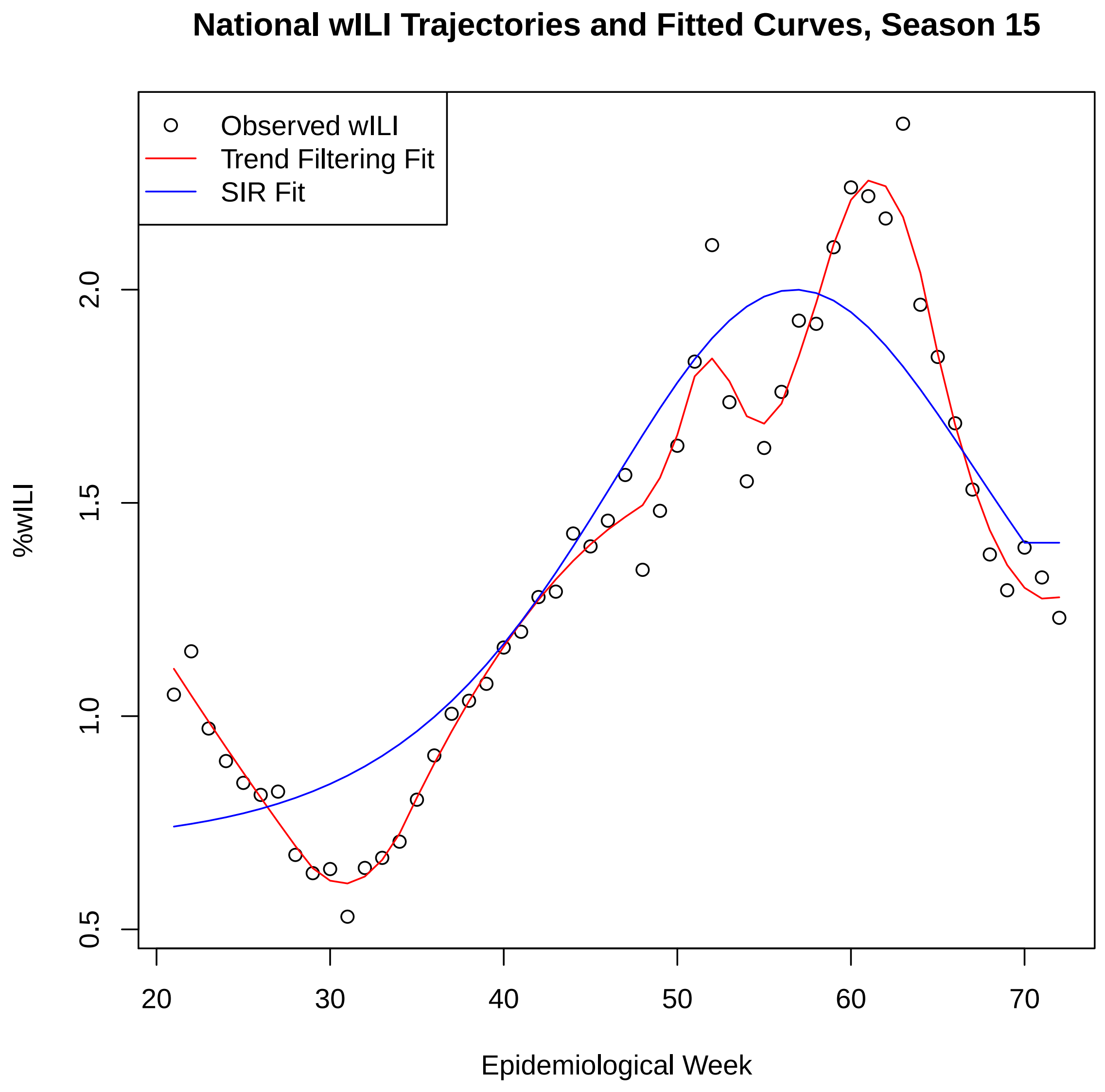

Curves fitted by trend filtering and SIR model

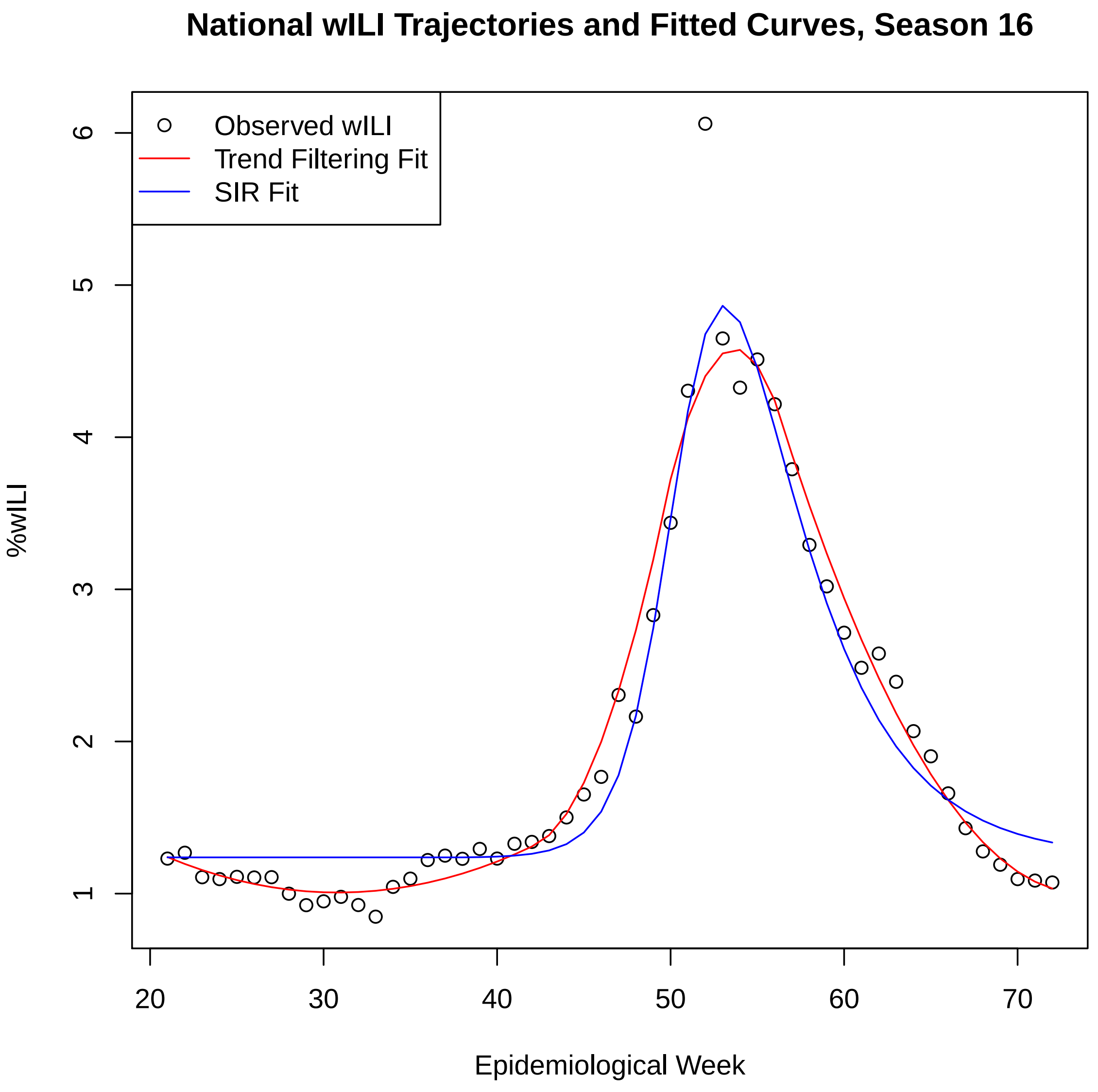

Supplement: S5 Fig — Contains the national wILI trajectory for each historical season, and the curves fit by (A) trend filtering, which are transformed to form the EB prior over curves, and (B) the single-strain fully-mixed affine-observation SIR model, which are used by EB-SIR to form a prior over SIR parameters. (PDF) [file pcbi.1004382.s005.pdf]
