# Supplementary figures and images for: Flexible Modeling of Epidemics with an Empirical Bayes Framework
Source: PLoS Comput Biol. 2015 Aug 28;11(8):e1004382. doi: 10.1371/journal.pcbi.1004382 (PMC4552841; doi:10.1371/journal.pcbi.1004382)

# Diagram of the generation process for ILINet and GFT data

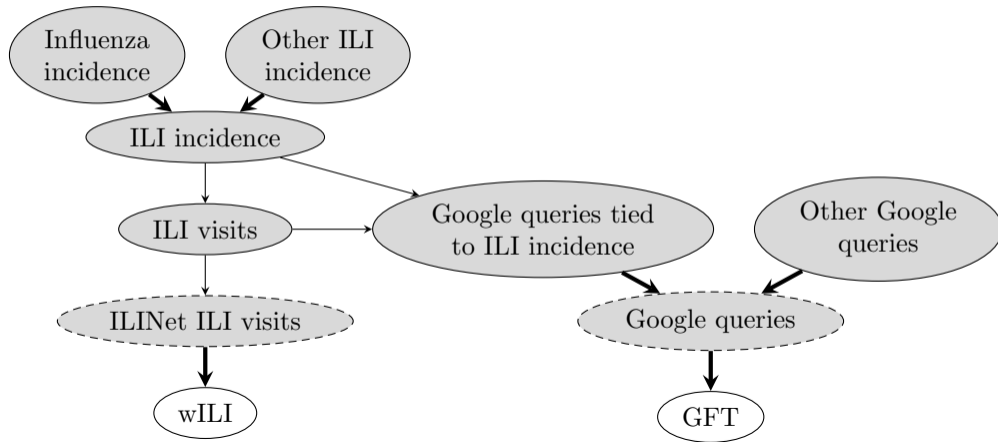

Supplement: S6 Fig — We are interested in influenza and other ILI incidence, but cannot observe them directly. Instead, we rely on wILI as a measure of flu prevalence, and sometimes use GFT to approximate wILI. Shaded nodes, unobserved quantities; shaded dashed nodes, proprietary data; unshaded nodes, publicly available data; thin arrows, dependencies; thick arrows, deterministic dependencies. (PDF) [file pcbi.1004382.s006.pdf]
