# Supplementary material for: Flexible Modeling of Epidemics with an Empirical Bayes Framework
Source: PLoS Comput Biol. 2015 Aug 28;11(8):e1004382. doi: 10.1371/journal.pcbi.1004382 (PMC4552841; doi:10.1371/journal.pcbi.1004382)

Trend filtering, SIR, and smoothing spline fits for HHS region 3 for two seasons

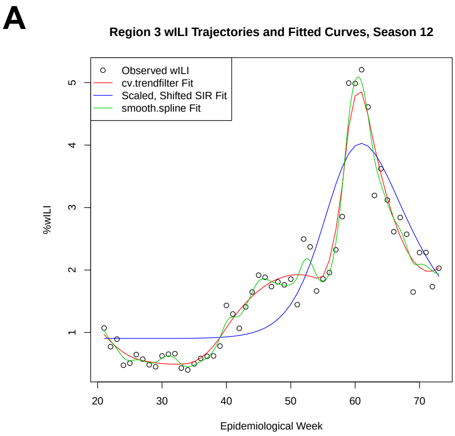

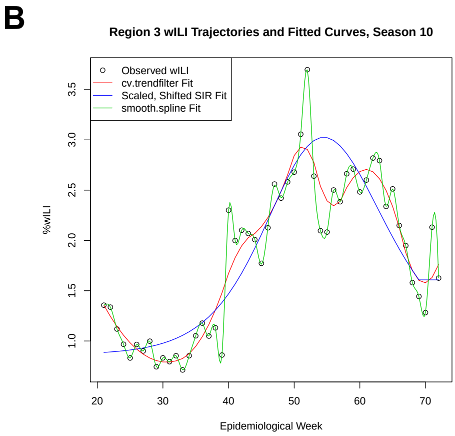

Supplement: S7 Fig — The quadratic trend filtering fit was performed with the cv.trendfilter [41] method, which automatically selects a level of smoothness to use. The cubic natural smoothing spline fit was produced by smooth.spline [46], which also automatically selects a level of smoothness, but by different criteria. (A) 2008–2009 season: trend filtering and smoothing splines both smooth out the holiday effects. The smoothing spline appears to overfit to noise in the preseason and early flu season. (B) 2006–2007 season: in addition to holiday effects, there is a large jump in wILI at week 40, which coincides with the beginning of the influenza season and a large jump in the number of reporting providers (from about 30 to over 100). The trend filtering procedure has trouble matching the beginning-of-season and holiday effects, attributing most of these effects to noise and smoothing them out. The smooth.spline procedure selects a level of smoothness that essentially duplicates the observed wILI and would produce a noise estimate near 0, which does not seem appropriate. Alternative methods of selecting a level of smoothness may produce looser fits and avoid these near-0 noise estimates, though. Beginning-of-season and holiday effects can be incorporated in both of the smoothing procedures, and would likely improve the resulting fits. Regional wILI dynamics are generally not tightly fit by the described SIR model. (PDF) [file pcbi.1004382.s007.pdf]
