# Supplementary material for: Flexible Modeling of Epidemics with an Empirical Bayes Framework
Source: PLoS Comput Biol. 2015 Aug 28;11(8):e1004382. doi: 10.1371/journal.pcbi.1004382 (PMC4552841; doi:10.1371/journal.pcbi.1004382)

National and regional residuals after trend-filtering fit

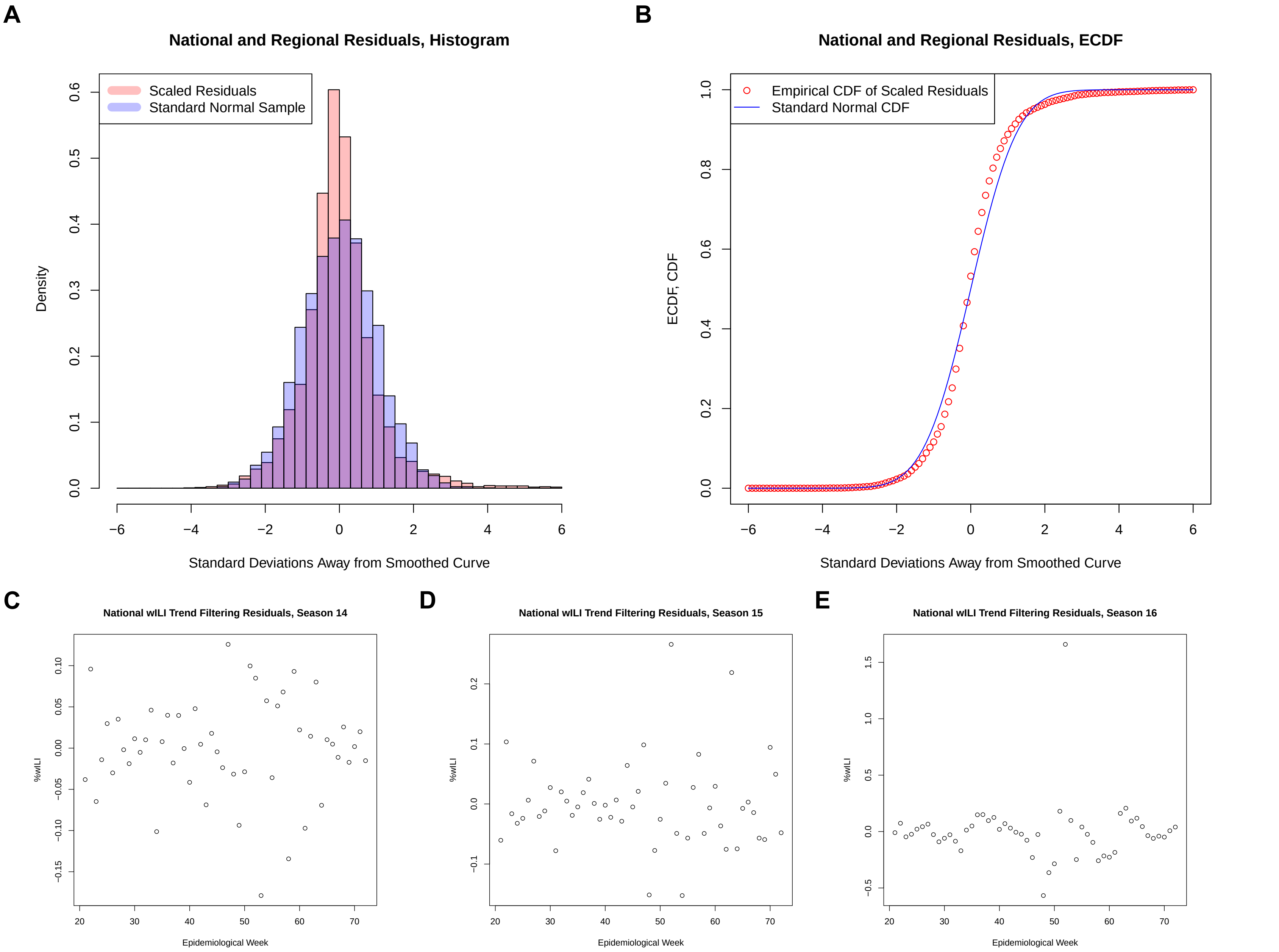

Supplement: S8 Fig — We took the difference between the observed wILI and trend-filtered fit for each historical season and region, and put them on the same scale by dividing by the standard deviation of residuals in the corresponding season and region (giving a z-score). (A) A histogram of the scaled residuals, together with a histogram for a standard normal random sample of the same size. (B) The empirical cumulative distribution function of the residuals, together with the cumulative distribution function of a standard normal random variable. (C,D,E) The (unscaled) residuals for the trends filtering fits to the national data for the (C) 2010–2011, (D) 2011–2012, and (E) 2012–2013 seasons. The trend filtering fits corresponding to (C), (D), and (E) are shown in S5 Fig. The winter holiday effect is unusually pronounced in (E), while (C) and (D) show more typical residual patterns. The trend-filtering residuals do not look exactly normally distributed; deviations from normality may be due to holiday effects, autocorrelation between residuals, and/or non-normality of wILI measurements. (PDF) [file pcbi.1004382.s008.pdf]
