# Supplementary material for: Flexible Modeling of Epidemics with an Empirical Bayes Framework
Source: PLoS Comput Biol. 2015 Aug 28;11(8):e1004382. doi: 10.1371/journal.pcbi.1004382 (PMC4552841; doi:10.1371/journal.pcbi.1004382)
Supplement: S1 Table — Most years are assigned 52 epidemiological weeks, but a few are assigned 53; we examine prediction error at weeks 39–52 and 1–20 when the first year of a season has 52 weeks, and weeks 39–53 and 1–19 when the first year has 53 weeks. Predictors are assigned the following abbreviations: “bl”, baseline (mean of other seasons); “splice”, pinned baseline (mean of other seasons, conditioned on current season to date); “eb”, empirical Bayes framework, using other seasons’ wili curves; “per”, pointwise percentile method; “knn”, k-nearest neighbors method; “ebsir”, empirical Bayes framework, modified to use SIR curves. Predictions were generated as if the final revision of the wILI data was available immediately. The week number in the leftmost column is the epidemiological week of the latest wILI measurement revealed to the predictors; EW39 corresponds to using no wILI values from the left-out season. All units of measurement are omitted. This table was generated using the xtable package [51]. (PDF) [file pcbi.1004382.s009.pdf]

Leave-one-out cross-validated bias and variance estimates, aligned by epidemiological week

	Onset		Peak Week		Peak Height		Duration	
Week, Predictor	Bias	Variance	Bias	Variance	Bias	Variance	Bias	Variance
All weeks, bl	0.0000	16.2276	0.0000	13.9567	0.0000	3.0419	0.0000	13.4590
EW39, splice	0.0000	16.2276	0.0000	13.9567	0.0000	3.0419	0.0000	13.4590
EW40, splice	0.0000	16.2276	0.0000	13.9567	0.0000	3.0419	0.0000	13.4590
EW41, splice	0.0000	16.2276	0.0000	13.9567	0.0000	3.0419	0.0000	13.4590
EW42, splice	0.0000	16.2276	0.0000	13.9567	0.0000	3.0419	0.0000	13.4590
EW43, splice	0.0000	16.2276	0.0000	13.9567	0.0000	3.0419	0.0000	13.4590
EW44, splice	-0.0042	16.1846	0.0000	13.9567	0.0000	3.0419	0.0042	13.4184
EW45, splice	0.0583	16.1232	-0.0750	13.2787	0.0002	3.0407	-0.0583	13.4921
EW46, splice	0.3750	15.2059	-0.0708	13.3117	0.0014	3.0334	-0.2667	13.4690
EW47, splice	0.1042	12.9831	-0.3000	12.7040	0.0111	2.9768	-0.1833	14.2889
EW48, splice	0.0167	11.1950	-0.3542	12.5930	0.0321	2.8631	-0.2000	13.7908
EW49, splice	-0.1417	9.1792	-0.4875	11.3924	0.0669	2.7065	-0.1167	13.2145
EW50, splice	-0.3125	6.7732	-0.7042	11.0477	0.1778	2.4180	-0.0833	13.5535
EW51, splice	-0.4375	4.1043	-1.0958	8.1914	0.2727	2.1471	0.0375	12.5635
EW52, splice	-0.5833	3.3964	-1.3375	5.8000	0.2563	1.7092	0.1417	12.1315
EW53/EW01, splice	-0.5485	2.2196	-1.1854	5.5341	0.1162	1.5391	0.1083	10.9410
EW01/EW02, splice	-0.3214	1.0898	-1.0137	5.1310	0.0216	1.4677	-0.0167	10.6889
EW02/EW03, splice	-0.2040	0.5769	-0.6836	5.1085	0.0925	1.0961	-0.0417	10.4035
EW03/EW04, splice	-0.0975	0.2635	-0.7700	4.9601	0.1232	0.9194	-0.0458	9.2704
EW04/EW05, splice	-0.0529	0.0653	-0.2064	3.5087	0.2847	0.4214	-0.0125	8.2372
EW05/EW06, splice	0.0000	0.0000	0.0740	2.5109	0.3343	0.1870	-0.0417	6.5167
EW06/EW07, splice	0.0000	0.0000	0.4177	1.8649	0.2576	0.0897	-0.0583	5.0044
EW07/EW08, splice	0.0000	0.0000	0.2958	0.9375	0.1218	0.0318	-0.0583	4.1161
EW08/EW09, splice	0.0000	0.0000	0.1667	0.5422	0.0586	0.0130	-0.0375	2.9920
EW09/EW10, splice	0.0000	0.0000	0.0708	0.0880	0.0212	0.0029	-0.2292	1.1893
EW10/EW11, splice	0.0000	0.0000	0.0458	0.0336	0.0057	0.0005	-0.3250	0.3786
EW11/EW12, splice	0.0000	0.0000	0.0292	0.0136	0.0015	0.0000	-0.1167	0.1799
EW12/EW13, splice	0.0000	0.0000	0.0292	0.0136	0.0015	0.0000	0.0000	0.0000
EW13/EW14, splice	0.0000	0.0000	0.0292	0.0136	0.0015	0.0000	0.0000	0.0000
EW14/EW15, splice	0.0000	0.0000	0.0292	0.0136	0.0015	0.0000	0.0000	0.0000
EW15/EW16, splice	0.0000	0.0000	0.0292	0.0136	0.0015	0.0000	0.0000	0.0000
EW16/EW17, splice	0.0000	0.0000	0.0292	0.0136	0.0015	0.0000	0.0000	0.0000
EW17/EW18, splice	0.0000	0.0000	0.0000	0.0000	0.0000	0.0000	0.0000	0.0000
EW18/EW19, splice	0.0000	0.0000	0.0000	0.0000	0.0000	0.0000	0.0000	0.0000
EW19/EW20, splice	0.0000	0.0000	0.0000	0.0000	0.0000	0.0000	0.0000	0.0000
EW39, eb	-1.3683	14.6834	-0.7048	12.3395	0.0581	2.6886	0.3120	12.9559
EW40, eb	-0.2359	17.0516	0.0637	14.8989	0.0478	2.6831	0.0681	12.5243
EW41, eb	-0.4482	17.4568	-0.0523	13.8363	0.0453	2.6277	0.2372	13.2797
EW42, eb	-0.5753	15.1804	-0.1636	12.7525	0.0386	2.6204	0.3461	13.7412
EW43, eb	-0.4303	15.9185	-0.0642	12.2951	0.0306	2.6084	0.4400	15.6307
EW44, eb	-0.8199	15.0793	-0.2945	13.8429	0.0438	2.5040	0.6382	14.9225
EW45, eb	-0.6538	10.5027	-0.2341	12.9821	0.0384	2.5454	0.5527	14.1195
EW46, eb	-0.6646	9.3439	-0.3130	12.2822	0.0479	2.5291	0.6044	13.7023
EW47, eb	-1.0978	8.7012	-0.6728	11.9278	0.1777	2.4366	0.5568	13.8698
EW48, eb	-0.5716	7.3771	-0.1990	8.3687	-0.0667	3.1534	0.0094	12.6947
EW49, eb	-0.2231	6.7093	0.0997	8.1231	-0.2105	3.0043	-0.0905	13.9767
EW50, eb	-0.3324	5.8932	-0.1981	9.1617	-0.1204	3.4127	-0.0765	18.0436
EW51, eb	-0.5213	3.4601	-0.4280	8.7569	0.0205	3.2622	0.1592	17.1434

	Onset		Peak Week		Peak Height		Duration	
Week, Predictor	Bias	Variance	Bias	Variance	Bias	Variance	Bias	Variance
EW52, eb	-0.7488	2.9597	-0.2013	8.0907	0.5062	1.2562	1.0584	11.9685
EW53/EW01, eb	-0.6605	2.2068	-0.0668	11.3573	0.2562	2.2659	0.8288	14.5039
EW01/EW02, eb	-0.2155	0.6258	0.2150	12.1756	-0.0904	1.8285	-0.1356	13.3206
EW02/EW03, eb	-0.0854	0.3488	0.2218	9.4779	0.2062	1.6053	-0.0067	4.8841
EW03/EW04, eb	-0.0125	0.1358	0.1467	7.5909	0.0646	1.1971	-0.1118	4.1492
EW04/EW05, eb	-0.0022	0.0023	0.8216	7.5349	0.1764	0.4593	0.7434	6.2939
EW05/EW06, eb	0.0537	0.0461	0.4864	5.3437	0.1103	0.0768	0.5264	7.5699
EW06/EW07, eb	0.0394	0.0248	0.6616	4.7960	0.0870	0.0485	0.5145	5.6693
EW07/EW08, eb	0.0186	0.0055	0.4665	3.7520	0.0144	0.0029	0.3912	1.9780
EW08/EW09, eb	0.0000	0.0000	0.1027	0.6475	-0.0030	0.0006	0.4322	1.8886
EW09/EW10, eb	0.0000	0.0000	-0.0541	0.1199	-0.0063	0.0007	0.3807	1.7018
EW10/EW11, eb	0.0000	0.0000	0.0232	0.0074	0.0008	0.0000	0.4339	1.2235
EW11/EW12, eb	0.0000	0.0000	0.0164	0.0043	0.0004	0.0000	0.4265	0.9528
EW12/EW13, eb	0.0000	0.0000	0.0093	0.0014	0.0002	0.0000	0.1904	0.0852
EW13/EW14, eb	0.0000	0.0000	0.0055	0.0005	0.0001	0.0000	0.0212	0.0024
EW14/EW15, eb	0.0000	0.0000	0.0045	0.0003	0.0000	0.0000	0.0132	0.0012
EW15/EW16, eb	0.0000	0.0000	0.0034	0.0002	0.0000	0.0000	0.0054	0.0002
EW16/EW17, eb	0.0000	0.0000	0.0010	0.0000	0.0000	0.0000	0.0021	0.0000
EW17/EW18, eb	0.0000	0.0000	0.0011	0.0000	0.0000	0.0000	0.0050	0.0003
EW18/EW19, eb	0.0000	0.0000	0.0006	0.0000	0.0000	0.0000	0.0358	0.0205
EW19/EW20, eb	0.0000	0.0000	0.0000	0.0000	0.0000	0.0000	0.0000	0.0000
EW39, per	0.0000	16.2276	0.0000	13.9567	0.0000	3.0419	0.0000	13.4590
EW40, per	-2.0000	15.6923	-1.2857	18.9890	-0.7537	6.3261	0.8750	40.2500
EW41, per	-2.2143	16.7967	-1.0714	24.9945	-0.7164	5.6286	1.4375	45.5958
EW42, per	-0.9333	25.3524	-0.8667	20.1238	-0.7362	5.1276	1.4375	38.9292
EW43, per	-0.2500	25.9333	-0.8125	20.1625	-0.8528	5.5846	1.1875	30.5625
EW44, per	-0.3125	22.6292	-0.3125	19.6958	-0.9532	4.9736	1.1875	27.8958
EW45, per	-0.5625	16.5292	-1.2500	20.8667	-0.9848	4.6183	1.3750	23.9833
EW46, per	-0.3750	14.2500	-1.6250	23.1833	-1.0356	4.1186	1.3750	21.3167
EW47, per	-0.6875	10.4958	-2.0000	17.8667	-1.0913	2.9846	1.5000	20.1333
EW48, per	-0.6250	9.7167	-1.1875	19.2292	-1.0935	2.5043	1.5000	19.7333
EW49, per	-0.7500	7.1333	-1.3750	15.0500	-1.0778	2.1149	1.5625	20.3958
EW50, per	-0.6875	6.2292	-0.8750	17.1833	-0.9751	1.9669	1.5000	20.2667
EW51, per	-0.6667	3.6667	-0.3333	13.8095	-0.9442	1.8090	1.3125	18.7625
EW52, per	-0.6667	3.6667	-1.8000	8.7429	-0.7581	1.6013	1.3125	18.7625
EW53/EW01, per	-0.5333	2.1238	-1.7333	7.9238	-0.6948	1.5197	1.1875	17.7625
EW01/EW02, per	-0.2667	1.0667	-1.7333	7.9238	-0.7035	1.4696	0.8750	16.5167
EW02/EW03, per	-0.2000	0.6000	-1.4667	6.4095	-0.5849	1.0879	0.7500	16.3333
EW03/EW04, per	-0.1333	0.2667	-1.2667	5.7810	-0.5144	0.7225	0.6250	15.3167
EW04/EW05, per	-0.0667	0.0667	-0.3333	5.6667	-0.2567	0.2249	0.5625	14.2625
EW05/EW06, per	0.0000	0.0000	-0.1333	5.1238	-0.0390	0.0609	0.4375	12.3958
EW06/EW07, per	0.0000	0.0000	0.3125	4.7625	0.0145	0.0030	0.5625	8.2625
EW07/EW08, per	0.0000	0.0000	-0.1875	0.5625	-0.0093	0.0014	0.5625	6.1292
EW08/EW09, per	0.0000	0.0000	-0.1875	0.5625	-0.0093	0.0014	0.4375	4.3958
EW09/EW10, per	0.0000	0.0000	-0.1875	0.5625	-0.0093	0.0014	-0.4375	0.7958
EW10/EW11, per	0.0000	0.0000	0.0000	0.0000	0.0000	0.0000	-0.3750	0.5167
EW11/EW12, per	0.0000	0.0000	0.0000	0.0000	0.0000	0.0000	-0.1875	0.2958
EW12/EW13, per	0.0000	0.0000	0.0000	0.0000	0.0000	0.0000	0.0000	0.0000
EW13/EW14, per	0.0000	0.0000	0.0000	0.0000	0.0000	0.0000	0.0000	0.0000
EW14/EW15, per	0.0000	0.0000	0.0000	0.0000	0.0000	0.0000	0.0000	0.0000

	Onset		Peak Week		Peak Height		Duration	
Week, Predictor	Bias	Variance	Bias	Variance	Bias	Variance	Bias	Variance
EW15/EW16, per	0.0000	0.0000	0.0000	0.0000	0.0000	0.0000	0.0000	0.0000
EW16/EW17, per	0.0000	0.0000	0.0000	0.0000	0.0000	0.0000	0.0000	0.0000
EW17/EW18, per	0.0000	0.0000	0.0000	0.0000	0.0000	0.0000	0.0000	0.0000
EW18/EW19, per	0.0000	0.0000	0.0000	0.0000	0.0000	0.0000	0.0000	0.0000
EW19/EW20, per	0.0000	0.0000	0.0000	0.0000	0.0000	0.0000	0.0000	0.0000
EW39, knn	-8.7120	13.8254	-10.9061	11.7477	-0.9117	2.8697	-4.1993	13.1550
EW40, knn	-10.4419	340.7679	-13.0872	352.4695	-1.2717	3.0460	-5.2232	20.7891
EW41, knn	-5.9967	118.3835	-7.2394	160.5568	-0.8421	4.0008	-2.4171	31.3419
EW42, knn	-3.3464	76.4803	-4.3017	111.8005	-0.6601	3.4380	-0.7567	29.3526
EW43, knn	-4.0971	145.0482	-5.1159	209.1143	-0.7264	3.5555	-0.4598	37.4966
EW44, knn	-0.3767	21.9166	-0.6958	21.9734	-0.6100	2.5889	0.4250	13.2386
EW45, knn	-0.7228	24.4833	-1.2457	26.6108	-0.5867	2.8380	0.3250	14.7753
EW46, knn	0.0228	14.1718	-0.4382	16.4066	-0.5359	2.8201	0.5946	16.1227
EW47, knn	-0.5367	9.2108	-0.9316	16.4781	-0.4399	3.1306	1.4297	14.5795
EW48, knn	-0.4158	6.6991	-0.6438	11.3774	-0.5132	3.3047	1.2676	16.8824
EW49, knn	-0.1716	6.1779	-0.4374	11.3338	-0.5436	3.2523	0.8126	20.5279
EW50, knn	-0.1185	5.0167	-0.6101	11.1436	-0.4573	3.6018	0.7415	23.9303
EW51, knn	-0.4462	5.6742	-0.8098	12.4346	-0.2724	2.4047	0.4500	17.9509
EW52, knn	-0.4984	3.5604	-1.0436	7.2930	0.1844	1.8262	1.0139	14.7664
EW53/EW01, knn	-0.5372	1.8718	-0.5462	6.6876	0.2313	2.0593	1.2877	14.4750
EW01/EW02, knn	-1.2646	19.4290	-1.6671	28.6025	-0.0855	1.5659	-0.0092	15.1151
EW02/EW03, knn	-0.8065	8.8553	-1.1175	18.5054	-0.0595	1.0623	-0.1121	11.4868
EW03/EW04, knn	-0.0063	0.0381	0.1679	4.5836	0.2228	0.9637	0.6256	7.8051
EW04/EW05, knn	0.0470	0.0171	0.5739	2.3217	0.2542	0.4810	0.6314	9.4912
EW05/EW06, knn	0.0921	0.1358	0.9400	1.8391	0.3936	0.3304	0.8940	10.3003
EW06/EW07, knn	0.0875	0.1226	0.9911	1.3874	0.4205	0.3237	0.9215	8.6460
EW07/EW08, knn	0.0404	0.0261	0.8891	1.5903	0.2952	0.2536	1.0343	6.8323
EW08/EW09, knn	-0.0000	0.0000	0.5718	1.3998	0.1799	0.2542	0.8621	6.7936
EW09/EW10, knn	0.0000	0.0000	0.4486	1.2254	0.1655	0.3569	0.8039	6.2430
EW10/EW11, knn	0.0000	0.0000	0.2817	1.2694	0.1754	0.4921	0.8469	4.9164
EW11/EW12, knn	0.0000	0.0000	0.3736	2.2338	0.1775	0.5039	0.9994	3.8841
EW12/EW13, knn	-0.0000	0.0000	0.4450	3.1680	0.1300	0.2703	0.5614	2.3919
EW13/EW14, knn	0.0000	0.0000	0.5086	4.1396	0.1072	0.1840	0.3264	1.2971
EW14/EW15, knn	0.0000	0.0000	0.4404	3.1034	0.0778	0.0969	0.1615	0.3353
EW15/EW16, knn	-0.0000	0.0000	0.1853	0.5493	0.0170	0.0046	0.0182	0.0053
EW16/EW17, knn	-0.0000	0.0000	-0.0000	0.0000	-0.0000	0.0000	-0.0000	0.0000
EW17/EW18, knn	-0.0000	0.0000	-0.0000	0.0000	0.0000	0.0000	-0.0000	0.0000
EW18/EW19, knn	0.0000	0.0000	0.0000	0.0000	0.0000	0.0000	0.0000	0.0000
EW19/EW20, knn	0.0000	0.0000	0.0000	0.0000	-0.0000	0.0000	-0.0000	0.0000
EW39, ebsir	-1.1954	14.5300	-1.6592	12.8341	-0.0627	2.9642	-0.5128	12.7544
EW40, ebsir	-0.7879	14.8024	-1.2641	13.7480	-0.0136	3.1515	-0.2290	11.1240
EW41, ebsir	-0.7453	16.6270	-1.1423	13.9564	0.0105	3.1475	0.2646	12.0290
EW42, ebsir	-0.6646	16.6953	-0.9911	13.7084	-0.0165	2.9851	0.6496	11.8704
EW43, ebsir	-0.3889	17.8120	-0.7142	14.3086	-0.0141	2.9573	0.7551	12.4100
EW44, ebsir	-0.8981	14.8169	-0.9798	14.2250	-0.0868	2.8470	1.4706	12.0126
EW45, ebsir	-0.5976	12.5010	-0.6744	13.1333	-0.1762	2.7527	1.4364	12.0954
EW46, ebsir	-0.7782	9.2146	-0.5740	11.2982	-0.3448	2.6660	2.1586	9.2101
EW47, ebsir	-1.1744	7.4824	-0.9366	11.9738	-0.4561	2.7483	2.2627	8.1528
EW48, ebsir	-0.6911	4.7284	-0.1093	8.3044	-0.7590	2.6897	2.6478	7.2520
EW49, ebsir	-0.0864	3.7140	0.7469	6.7075	-0.6428	3.1156	2.5966	6.5997

	Onset		Peak Week		Peak Height		Duration	
Week, Predictor	Bias	Variance	Bias	Variance	Bias	Variance	Bias	Variance
EW50, ebsir	0.3163	5.2890	0.9557	6.9338	-0.6230	3.3632	1.9161	8.0719
EW51, ebsir	-0.2200	3.6021	-0.0408	5.7004	-0.2286	3.0851	1.9464	9.4071
EW52, ebsir	-0.7124	3.0373	-0.4725	4.5852	-0.0652	1.8135	2.6739	8.4739
EW53/EW01, ebsir	-0.5237	1.8475	-0.2601	4.4875	-0.3084	1.7382	2.1696	10.2676
EW01/EW02, ebsir	0.0759	0.8087	0.1234	5.1736	-0.6142	1.2898	1.1904	6.9612
EW02/EW03, ebsir	-0.0246	0.4197	-0.4973	7.4017	-0.4369	1.5237	0.1789	11.7250
EW03/EW04, ebsir	0.0342	0.1005	-0.4478	4.8013	-0.3379	0.8846	0.7796	13.5534
EW04/EW05, ebsir	0.0970	0.1024	0.2626	1.4561	-0.1285	0.3395	1.4625	11.7869
EW05/EW06, ebsir	0.0689	0.0645	0.6088	1.1083	0.0682	0.0728	2.4003	10.4557
EW06/EW07, ebsir	0.0568	0.0516	0.8223	1.5552	0.1440	0.0563	2.4456	14.5140
EW07/EW08, ebsir	0.0148	0.0035	1.1156	2.3491	0.1313	0.0553	2.8339	11.2242
EW08/EW09, ebsir	0.0000	0.0000	0.8199	1.3911	0.0640	0.0161	2.2715	8.0125
EW09/EW10, ebsir	0.0000	0.0000	0.5975	2.1884	0.0420	0.0150	1.8930	6.5177
EW10/EW11, ebsir	0.0000	0.0000	0.2578	0.2479	0.0118	0.0009	1.2423	2.8229
EW11/EW12, ebsir	0.0000	0.0000	0.1878	0.3904	0.0089	0.0011	0.8815	0.6077
EW12/EW13, ebsir	0.0000	0.0000	0.1129	0.1425	0.0046	0.0003	0.4862	0.5783
EW13/EW14, ebsir	0.0000	0.0000	0.2448	0.8573	0.0107	0.0018	0.2145	0.1951
EW14/EW15, ebsir	0.0000	0.0000	0.0291	0.0122	0.0007	0.0000	0.0568	0.0104
EW15/EW16, ebsir	0.0000	0.0000	0.0154	0.0037	0.0003	0.0000	0.0077	0.0004
EW16/EW17, ebsir	0.0000	0.0000	0.0022	0.0001	0.0000	0.0000	0.0023	0.0000
EW17/EW18, ebsir	0.0000	0.0000	0.0028	0.0001	0.0000	0.0000	0.0038	0.0001
EW18/EW19, ebsir	0.0000	0.0000	0.0007	0.0000	0.0000	0.0000	0.0269	0.0116
EW19/EW20, ebsir	0.0000	0.0000	0.0003	0.0000	0.0000	0.0000	0.0000	0.0000
