# Supplementary material for: Flexible Modeling of Epidemics with an Empirical Bayes Framework
Source: PLoS Comput Biol. 2015 Aug 28;11(8):e1004382. doi: 10.1371/journal.pcbi.1004382 (PMC4552841; doi:10.1371/journal.pcbi.1004382)
Supplement: S1 Text — Pseudocode for one algorithm used to build a collection of possible futures for the current season. (PDF) [file pcbi.1004382.s010.pdf]

Importance sampling algorithm

Data: $y^{r,\, s_{\text{curr}}}$, the wILI observations so far; $z^{r, s_{\text{curr}}}$, a version of $y^{r,\, s_{\text{curr}}}$ with two extra points estimated from GFT; prior distributions of wILI curves, noise levels, and transformations

Result: weighted collection of curves

Let $\phi(x; \mu, \sigma)$ be the normal pdf;

for *a large number of times* **do**

  Randomly draw f^r, σ, ν, θ, and μ from the corresponding priors;

  Let $f^{r,\, s_{\text{curr}}}(i) = f_4^r(i) = b^r + \frac{\theta^r - b^r}{\max_j f^r(j) - b^r}\left[f^r\left(\frac{i-\mu^r}{\nu^r} + \arg\max_j f^r(j)\right) - b^r\right]$;

  Calculate weight $w = \prod_{i=1}^{\text{length}(z^{r, s_{\text{curr}}})} \phi(z; f^{r,\, s_{\text{curr}}}(i), \sigma)$;

  Let v be a 53-length vector, a possible curve for this season;

  for i *in* 1..length($y^{r,\, s_{\text{curr}}}$) **do**

    $v_i := y_i^{r,\, s_{\text{curr}}}$;

  end

  for i *in* (length($y^{r,\, s_{\text{curr}}}$) + 1)..53 **do**

    $v_i := f^{r,\, s_{\text{curr}}}(i)$;

  end

  Add curve v with weight w to the collection of possibilities for this season (the posterior estimate)

end

Algorithm 1: Importance sampling procedure

Note that, since the GFT estimates are not exact, we weight $f^{r,\, s_{\text{curr}}}$ based on $z^{r, s_{\text{curr}}}$, using both ILINet wILI observations and GFT data, but we construct each possible curve v using $f^{r,\, s_{\text{curr}}}$ and $y^{r,\, s_{\text{curr}}}$, using no GFT. However, since ILINet data can undergo revisions, we have also considered versions that construct each v ignoring some of the more recent values in $y^{r,\, s_{\text{curr}}}$.

To improve computational efficiency, we also use a modified version of the code above that first divides up the possible values of f^r, σ, ν, θ, and μ into bins and estimates the average weight of $f^{r,\, s_{\text{curr}}}$'s in each bin. By sampling values of f^r, σ, ν, θ, and μ more frequently from the higher-weighted bins (and correcting for this decision in the weight calculation), we are able to construct a collection of curves with a high total weight more quickly than the version above.
