# Supplementary material for: Flexible Modeling of Epidemics with an Empirical Bayes Framework
Source: PLoS Comput Biol. 2015 Aug 28;11(8):e1004382. doi: 10.1371/journal.pcbi.1004382 (PMC4552841; doi:10.1371/journal.pcbi.1004382)
Supplement: S2 Text — (PDF) [file pcbi.1004382.s011.pdf]

Current limitations and future work

Our framework bases forecasts for a geographical unit based on the ILINet wILI for that area, sometimes with adjustments to recent wILI based on GFT data for that area. We are investigating ways to improve forecasts by incorporating additional sources of data, dependencies between geographical units, and more accurate models of reporting behavior.

In addition to GFT, other proxy data sources, such as Twitter activity and thermometer sales, can be used to estimate some underlying flu signal; wILI and proxy data can be modeled as noisy measurements of this signal. This measurement model can be incorporated into the weighting step of the framework, and used to refine the results of the smoothing procedure when forming the prior.

The framework can also be adjusted to incorporate lab testing data and spatial similarities and interaction, although the process is less straightforward. Lab test samples in the U.S. are not uniform random samples of ILI doctors' visits; they are collected from hospital visits, which are biased towards certain strains and age groups, and sent in from doctors on a non-random basis, with a goal of detecting novel strains. The mixture of test types and collection policies have also changed over time, and subtyping is not always performed, introducing additional complications. One basic approach to incorporating this data is to use wILI and lab data together to produce a signal for each subtype and for non-flu ILI, forecast each signal separately, then combine these forecasts to produce wILI predictions. Incorporating weather, vaccination coverage, and wILI data from other regions, is more involved. A simplistic approach is to make forecasts of weather, vaccination, and/or wILI in other regions alongside the wILI prediction for the target region, extending the transformations to shift the additional data alongside the corresponding wILI curve, and then to adjust the weighting step to incorporate the additional data, adding a score or log-likelihood term that favors transformed histories that more closely resemble the current season in terms of these additional data sources. However, conditioning on additional data sources in this way may exacerbate problems with latching onto a very small part of the prior.

Forecast accuracy can potentially be improved by a better model of wILI measurements. Modeling the inflation in wILI levels around holidays due to sharp drops in the number of non-ILI visits may improve forecasts by preventing "latching" of late-peaking seasons onto early ones, or seasons with a single real peak onto ones with secondary peaks like 2006–2007. As additional reports from ILINet providers are received and processed, wILI values for a particular week can be revised; revisions of wILI values after a few weeks tend to be slightly higher than the initial values, and are more stable. A model of the revision process can be incorporated into the weighting step of the framework and reduce errors in conditioning due to bias or increased noise in recent wILI measurements. Adjusting our wILI noise model may also yield improvements in the historical curve-fitting and importance sampling weighting steps. For example, one choice that incorporates the fact that wILI lies between 0% and 100% without requiring additional domain information would be to use beta-

distributed noise (with some constraints on the relationship of the parameters to make fitting and smoothing procedures well-defined, such as fixed dispersion). Considering various Box-Cox transformations [15] would add additional flexibility to the Gaussian noise model while maintaining generality. Tailoring a model to wILI specifically may also be an option, but is complicated by the fact that (a) ILINet providers can start or stop reporting on a weekly basis, differ in size and type, and potentially encourage or discourage visits for ILI (e.g., in phone calls before a visit is scheduled) in a way that can change from week to week and based on current patient load; (b) ILI visits can have trends based on the day of the week [21] and time of year; and (c) wILI is a weighted average of the ILI visit proportions for the states [33], and these proportions are not publicly available for all states for all seasons (nor is the data from the individual providers, nor is daily-level data for ILINet). plots the trend-filtering residuals, which do not look exactly normally distributed; deviations from normality may be due to holiday effects, autocorrelation between residuals, and/or non-normality of wILI measurements.
